# Reliability, Device Agreement and Validity of Load–Velocity Profiles: A Systematic Review with Meta-analysis

**DOI:** 10.1186/s40798-026-01102-0

**Published:** 2026-09-03

**Authors:** Nina Claassen, Stanislav Dimitri Siegel, Mareike Sproll, Niklas Lebelt, Alyssa Virginia Bargende, Anne Marieke Fasold, Konstantin Warneke

**Affiliations:** 1 Institute of Human Movement Science and Exercise Physiology, University Osnabrück, Osnabrück, Germany; 2 Institute of Human Movement Science and Exercise Physiology, Friedrich Schiller University Jena, Jena, Germany; 3 Institute of Human Movement Science and Exercise Physiology, University of Hamburg, Hamburg, Germany; 4 Institute of Sustainability Psychology, Leuphana University Lüneburg, Lüneburg, Germany; 5 Experimental Orthopaedics, University Hospital Jena, Campus Eisenberg, Waldkliniken Eisenberg, Friedrich-Schiller-Universität Jena, Jena, Germany; 6 Orthopaedics, University Hospital Jena, Campus Eisenberg, Waldkliniken Eisenberg, Friedrich-Schiller-University Jena, Jena, Germany

**Keywords:** Velocity-based training, Load–velocity relationship, One-repetition maximum prediction, Movement velocity, Resistance training, Measurement validity, Measurement reliability, Wearable sensors, Meta-analysis

## Abstract

**Background:**

For a valid one-repetition maximum (1RM) prediction via load–velocity (LV) relationships, high reliability and accuracy must be assumed.

**Objective:**

Since individual study results indicate ambivalent prediction, this systematic review and meta-analysis was designed to provide a updated and comprehensive overview, extending knowledge about the validity and reliability of commercially available velocity sensors in Part I and the validity and reliability of velocity-based 1RM prediction models in Part II.

**Methods:**

A systematic literature search was conducted in PubMed/MEDLINE, Web of Science, and Scopus. Validity and/or reliability studies or velocity-based 1RM prediction evaluations were included. Methodological quality was assessed using adapted COSMIN. The analysis was performed for intraclass correlation coefficient (ICC), Lin’s concordance correlation coefficient (CCC), and Pearson’s correlation coefficient (*r*). The review was preregistered in PROSPERO (CRD42025634595).

**Results:**

Sixty-three studies were included for sensor validity and reliability and 38 for 1RM prediction models. Part I: Velocity sensors demonstrated good-to-excellent pooled validity and device agreement (ICC = 0.91–0.92 [0.83–0.97]; *k* = 55 and 439, respectively); intra- and inter-day reliability were classified as good to excellent with ICC = 0.90–0.91 [0.85–0.95] (*k* = 228 and 608, respectively), with sensor technology moderating the results. However, substantial heterogeneity and wide ranges of study-level estimates indicated considerable variability across moderators, linear position transducer (LPT) generally showing more consistent performance than inertial measurement units (IMU). Part II: Velocity-based 1RM prediction showed ICCs = 0.90 [0.83–0.94] (*k* = 124) and ICC = 0.91 [0.72–0.98] (*k* = 9); for reliability and validity, respectively.

**Discussion:**

Commercial velocity sensors generally provide high relative validity and reliability. Results varied depending on exercise complexity, intensity, sensor technology, and modeling approach. While velocity-based 1RM prediction demonstrated high average validity, large heterogeneity in lower body exercises significantly biased the results. Furthermore, the dearth of measurement error and agreement analyses prohibits final conclusions.

**Conclusion:**

Therefore, velocity-based monitoring and 1RM prediction require cautious interpretation, as sensor- and exercise-specific evidence remains limited.

**Supplementary Information:**

The online version contains supplementary material available at https://doi.org/10.1186/s40798-026-01102-0.

## Key Points

- For a valid one-repetition maximum (1RM) prediction via load-velocity relationships, high reliability and accuracy must be assumed.
- This systematic review included 63 studies that revealed good to excellent overall validity and device agreement as well as reliability.
- Exercise type, velocity sensor, and intensity, among others, moderated results.
- A dearth of systematic and random error evaluations calls for appropriate statistical calculations.

**Supplementary Information:**

The online version contains supplementary material available at https://doi.org/10.1186/s40798-026-01102-0.

## Introduction

The precise determination of maximal strength plays a vital role when designing resistance training programs. Herein, training intensity has been described to significantly moderate morphological and functional adaptations and is commonly calculated as a percentage of the one-repetition maximum (1RM). To achieve muscle hypertrophy, 60–85% of the 1RM [1] were sufficient, while higher intensities of  > 85% up to 100% were recommended for maximal strength training [2].

Training control on a microdosing level should consider daily performance variations potentially induced via life stress, training load-induced fatigue or recovery, among others [3, 4]. Since daily maximal strength testing before each training session is considered impractical, load–velocity profiles were frequently highlighted in the literature as a practical and valid alternative [5, 6]. In this approach, the bar velocity is measured when performing submaximal repetitions with, e.g., 50% and 70% of the 1RM to approximate the maximal achievable load by extrapolating the 1RM by assuming a minimal velocity threshold based on a hypothesized inverse linear relationship between load (in kg or %1RM) and movement velocity (mean, peak, or mean propulsive velocity) [7, 8]. Recent practical recommendations suggest that individualized load–velocity relationships can be established using as few as two submaximal loads, provided that one load is relatively heavy (> 75% 1RM) and that the selected loads produce a sufficiently large velocity difference of approximately 0.50 m s⁻^1^ to ensure a stable regression slope [6]. Such protocols can be implemented into warm-up routines, potentially reducing the need for periodic and time-consuming maximal strength testing [5]. Individual studies indicated strong correlations (*r* > 0.9) with deviations < 10 kg from the measured 1RM, rationalizing sufficient construct validity assumptions [7, 8].

Despite frequent recommendations for practical application [9–13], evidence supporting the valid estimation of maximal strength is ambivalent in quality and results, with studies reporting high inter-individual variability in movement velocity contradicting these recommendations [14, 15]. For instance, serious overestimations of the maximal strength from velocity profiles built in warm-up sets were reported in several studies, with systematic overestimations of up to 30 kg [7]. Although the predictability when using high loads near to the 1RM (e.g., 90% 1RM) and slow velocities improved [7, 16], practically relevant measurement errors called the approach into question [17]. Mean errors of up to 20% [18] were found when compared with the actual 1RM for lower [7, 16, 19–21] and upper body strength tests [19]. Numerous sensor types exist. These include linear position transducers (LPT), e.g., GymAware or Tendo, or inertial measurement units (IMUs) like PUSH or EnodePro, with differences in validity [22, 23], especially during complex, multi-joint movements such as squats or Olympic lifts [24, 25]. Numerous sources of variance stem from chosen exercise [23, 26], sensor placement [27], applied velocity threshold choice (minimal versus 0-velocity assumption in maximal strength test) [17], or the number and range of loads used to construct the load–velocity relationship [15] (two- or multipoint method [18, 25]) that affected the accuracy of the maximal strength prediction.

Beyond these influences causing systematic bias, sensor as well as test reliability and validity are of decisive importance in empirical data collections [28–30] and are crucial to allow reasonable conclusions on prediction errors in velocity-based 1RM estimations. Reliability describes the extent to which measurements can consistently distinguish between individuals or remain stable across repeated trials, whereas validity refers to the extent to which a sensor or prediction model corresponds to an appropriate reference criterion. Agreement, in contrast, describes the absolute closeness between repeated measurements or between sensors and is therefore crucial when evaluating sensor interchangeability. If criteria such as intra- and inter-day reliability and repeatability, objectivity, and validity are not fulfilled, internal data validity as the prerequisite for external and construct validity is violated [29]. If a data collection performed several times in a row results in different outcomes (repeatability problem), or the test outcome depends on the investigator/device collecting the data (objectivity problem), the results cannot be valid, per se [30]. However, detailed measurement error analyses in reliability and validity analyses accounting for different measurement error sources are scarce.

Although previous reviews have addressed commercially available velocity sensors [31] and LV-based 1RM prediction methods [11], neither the measurement quality of the underlying sensors nor device-to-device agreement was quantitatively synthesized in relation to 1RM prediction. The present review extends this work by first evaluating the validity, reliability, and agreement of commercially available velocity sensors and then examining the reliability and validity of velocity-based 1RM prediction models. In addition, updated evidence and moderator analyses were included for sensor technology, exercise type, intensity, velocity metric, and prediction method.

## Methods

The study protocol adhered to the PRISMA (Preferred Reporting Items for Systematic Reviews and Meta-Analyses) guidelines (see checklist in Supplemental Material S21) [32], and the study was conducted under consideration of ethical publishing standards for systematic reviews [33] and was registered in the PROSPERO database (CRD42025634595).

Part I addresses the validity, agreement, and reliability of velocity measurements obtained using commercially available velocity sensors, while Part II evaluates the reliability and validity of various approaches *to predict the 1RM.* Part-specific literature searches, eligibility screening, data extraction, and statistical analysis were performed.

### Literature Search

The “Participants Intervention Comparison Outcome” (PICO) framework [34] has limited validity for search term production. The search was performed in the scientific databases PubMed/MEDLINE, Web of Science (Core collection), and Scopus. Four investigators (NL, AB, AF, NC) independently performed the systematic literature search based on the following eligibility criteria for study inclusion:

Participants: healthy participants (no restriction for age, training level, type of sport) all available velocity sensors were included.

Outcome: reliability and validity metrics commonly used in the literature, including correlation coefficients (r) and intraclass correlation coefficients.

Study types: reliability and validity studies.

Studies were excluded if missing specific velocity parameters or lacked reliability metrics.

Specific search terms were created based on these criteria. The following search terms were used for PubMed/MEDLINE:

**Part I:**

*((“validity” OR “agreement” OR “reliability” OR "Intraclass correlation coefficient" OR "ICC" OR "test-retest reliability" OR "measurement reliability" OR "consistency" OR "repeatability")*

*AND*

*("velocity-based" OR "force-velocity" OR "load-velocity" OR "speed measurement" OR "velocity measurement" OR "velocity sensor" OR "load-velocity profiling" OR*

*tracking" OR “motion capture” OR “inertial sensor” OR “displacement transducer” OR “linear position transducer”))*

This search term was developed on 15 November 2024 and subsequently updated in April 2025.

**Part II:**

("load velocity" OR "velocity profile*" OR "velocity-based profile*" OR "velocity profiling" OR "load-velocity profiling" OR "velocity-load relationship".

*AND*

*("1RM" OR "one repetition maximum" OR "one rep max" OR "one-rep max" OR "maximal strength" OR "strength prediction" OR "strength estimation").*

*AND*

*("validity" OR "reliability" OR "agreement" OR "measurement" OR "accuracy" OR "consistency" OR "Intraclass correlation coefficient" OR "ICC" OR "test–retest").*

For this Part, the term was developed in June 2025. Further terms are provided in the Supplemental Material (S1 Search terms).

### Quality Assessment

Methodological study quality was evaluated via the COSMIN Risk of Bias Checklist for measurement instrument evaluations [35], using domains for *reliability* (Box 6) and *measurement error* (Box 7). Relevant design requirements and statistical considerations were simplified into five items. This approach was adopted from previous systematic reviews with similar research questions (e.g., [36]).

**Part I:**

- I1: Participant Selection: representativeness, comparability, and a sample size of  ≥ 30 were considered a minimum threshold to avoid an inadequate rating according to COSMIN’s general guidance for measurement reliability and validity analyses.
- I2: Measurement Protocol: clarity and standardization of sensor placement and test procedures. A study was scored "low" if essential details on sensor positioning, test environment, and movement execution were missing.
- I3: Reliability: specification of ICC or coefficient of variation (CV), with 95% confidence intervals, and appropriate test–retest design (minimum 1-day separation, same protocol conditions). Absence of reliability metrics or use of non-validated metrics (e.g., Pearson’s* r* only) downgraded.
- I4: Validity: presence of comparison with a gold standard (e.g., force plate, 3D motion capture), and application of at least one accepted metric such as Bland–Altman Limits of Agreement (LoA), mean absolute error (MAE), mean absolute percentage error (MAPE), standard error of the mean (SEM), or root mean square error (RMSE). If no comparison or inappropriate reference (e.g., self-report) [27] was used, the item was scored “low”.
- I5: Statistical Rigor: absence of major statistical flaws, such as missing units, inappropriate test selection (e.g., use of t-test for ICC), or lack of error margins. Ambiguities or partially reported results led to a downgrade.

**Part II:** For studies evaluating 1RM prediction models, definitions were adjusted:

- I2: Measurement Protocol: emphasized the clarity of procedures used to generate predictor variables (e.g., barbell velocity, submaximal loads) and the standardization of movement execution across conditions.
- I3: Reliability: focused on the test–retest consistency of model predictions and underlying input variables.
- I4: Validity: required a direct comparison between predicted and actual 1RM values, with appropriate statistical analysis (e.g., Bland–Altman (BA) analysis, standard error of estimate, or R^2^ values) and use of a true 1RM test as reference.
- I5: Statistical Rigor: additionally considered modeling quality (e.g., overfitting, validation methods, appropriate regression analysis) and reporting completeness (e.g., confidence intervals, error margins).

Each assessment item was rated on a three-point scale, with a maximum possible score of 15 points. Higher scores indicated better methodological quality. The quality assessment was conducted independently by two authors (AB and NL). Disagreements were solved by consulting a third person (NC).

### Data Processing and Statistics

The reliability- or/and validity metrics extraction process was conducted independently by four investigators (AF, AB, NL and NC). Missing information was requested from the corresponding author of the respective study via e-mail or ResearchGate. No response after the second reminder led to study exclusion.

Statistical analyses were conducted using RStudio (version 2024.09.1). The analysis leveraged several specialized R packages, including metafor, mixtools, ggplot2, and dplyr [37], and followed methodological approaches from previous high-quality analyses [38]. For reliability studies reporting intraclass correlation coefficients, the ICC variance was computed analytically following the method proposed by Donner [39], defined as:

$$ Var\,\left( {ICC} \right)\, = \,\frac{{2\,\left( {1\, - \,ICC} \right)^{2} \,\left( {1\, + \,\left( {n\, - \,1} \right)\,ICC} \right)^{2} }}{{k\, * \,n\,\left( {n\, - \,1} \right)}}, $$

where Var = variance, ICC = intraclass correlation coefficient, *k* = number of repeated measurements, *n* = number of participants. This estimator was originally derived for ICC(1,1) but was also applied to ICC(2,1) and ICC(3,1), as primary studies rarely reported the within-subject variance component required for estimating the sampling variance of these ICC formulations [40]. ICCs were analyzed on the logit scale and back-transformed for interpretation.

For validity studies reporting correlation coefficients, Fisher’s z-transformation was applied to stabilize the variance [41], and were back-transformed to *r* for interpretation. The corresponding sampling variance was calculated as:

$$ Var\,\left( z \right)\, = \,\frac{1}{n\, - \,3}, $$

where *n* = number of participants, *z* = fishers z transformed Pearson correlation coefficient.

To ensure consistency across included studies, only correlation coefficients (e.g., ICC, Pearson’s *r*, or Lin’s CCC) within the valid range of −1 to +1 and with a sample size greater than three were considered for meta-analytic modeling. The reliability-classification by Koo and Li [42] was applied, defining values ICC < 0.50 = poor, ICC = 0.50–0.75 = moderate, ICC = 0.75–0.90 = good, ICC > 0.90 = excellent.

A multilevel random-effects meta-analysis was conducted using restricted maximum likelihood (REML) estimation, with study entered as a random intercept to account for the non-independence of effect sizes within studies and between-study heterogeneity. Between-study heterogeneity was assessed using Cochran’s Q statistic [43] following Chi-square distributions. A statistically significant *Q*(df) value indicates the presence of substantial heterogeneity among included effects, suggesting that effect sizes vary across studies due to methodological or contextual differences rather than chance.

To assess the risk of publication bias, funnel plots were visually inspected for asymmetry across all outcome domains [44]. In addition, regression-based tests for funnel plot asymmetry (Egger-type tests) were applied to identify potential small-study effects [45].

To explore sources of heterogeneity, meta-regressions were fitted with the following variables as moderators:

1. Exercise category (accounting for movement complexity/stability (e.g., free-weight vs. machine-based exercises) [7, 27].
2. Measured variable (e.g., mean velocity, peak velocity) [5].
3. Training intensity (e.g., %1RM or categorical levels: low [< 50%], moderate [50–80%], high [> 80%] [2] as a continuous and categorical moderator [46]. The categorical model allows for a more intuitive interpretation of intensity levels.
4. Sensor type (LPTs versus IMUs) [47, 48].
5. Gold standard comparator (e.g., 3D motion capture vs. force plate) [49].

## Results

### Results Part I: Reliability and Validity of Different Velocity Sensors

#### Risk of Publication Bias

Funnel plots for all primary meta-analyses can be reviewed in the Supplementary Figures S2–S11. Overall, the risk of publication bias was ambivalent, with some visual inspection indicating asymmetry, while others indicated symmetrical distribution.

For device agreement (S2–S4), Egger’s test revealed significant funnel plot asymmetry in ICC [*t*(437) = −4.01, *p* < 0.001] and Lin’s CCC analyses [*t*(62) = −2.16, *p* = 0.035], suggesting the presence of small-study effects. In contrast, no statistically significant evidence of funnel plot asymmetry was found for Pearson-based agreement estimates (*p* = 0.0937), validity (S5-S6) via ICC (*p* = 0.118) or Pearson-based analyses (*p* = 0.611).

For interday reliability estimates, the funnel plot (S7-S9) suggested slight asymmetry, which was supported by a statistically significant Egger’s test [*t*(606) = 3.50, *p* < 0.001 for ICC-based analysis and *t*(42) =  7.34, *p* < 0.001 for Pearson-based analysis], indicating the presence of small-study effects. Asymmetry assessments with a study number of *n* < 10 (e.g., interday reliability analysis with Lin’s CCC and intraday reliability with Pearson’s *r*) require cautious interpretation [50].

For intraday reliability via ICCs (Figure S10), the funnel plot showed a symmetrical distribution of smaller studies, although clustering of effects was evident [*t*(226) = −4.23, *p* < 0.001].

#### Search Results

**Fig. 1:**
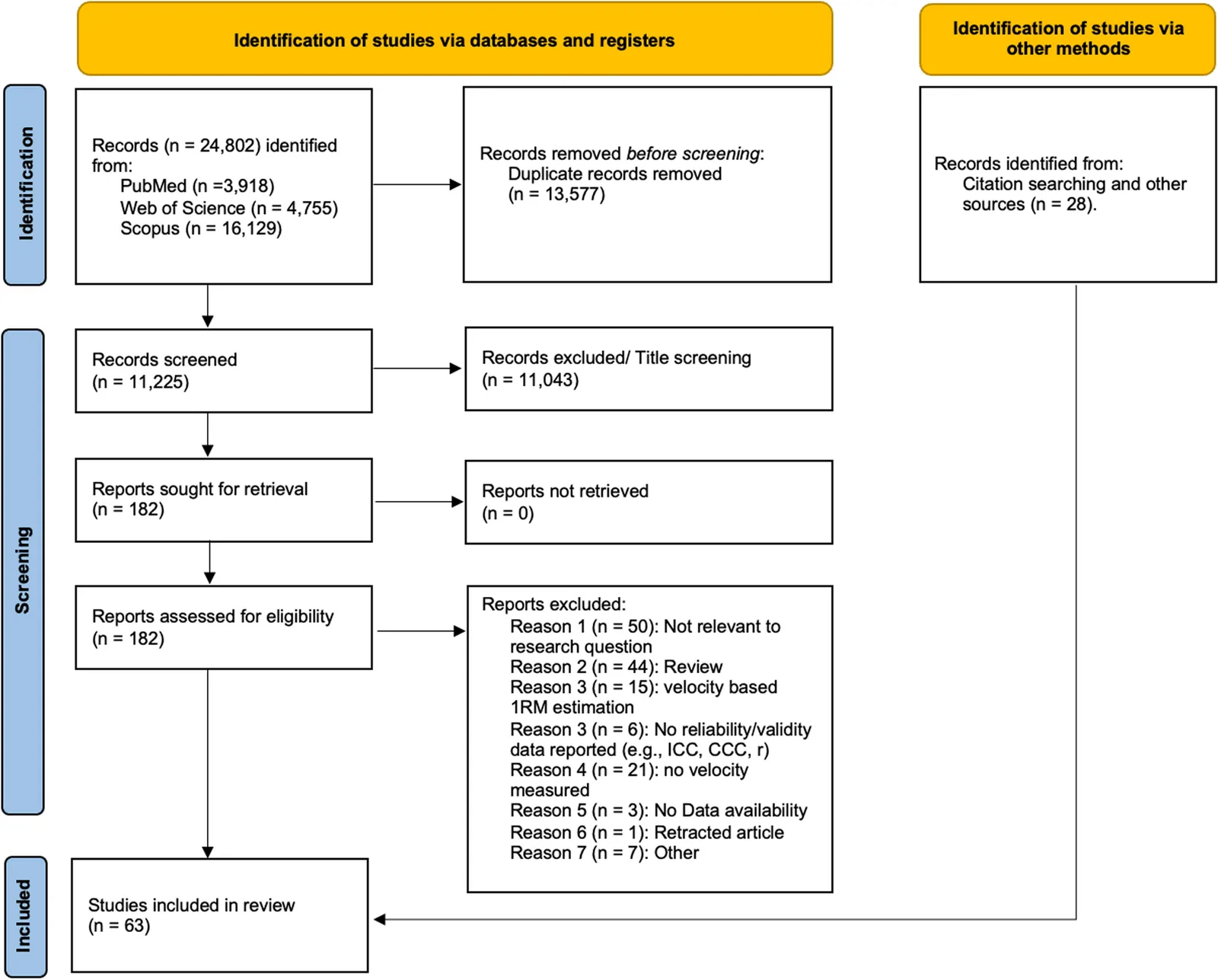
PRISMA flowchart of the literature search and study selection process for Part I: validity, reliability, and device agreement of commercially available velocity sensors. *1RM* one-repetition-maximum, *ICC* intraclass correlation coefficient, *CCC* Lin’s concordance correlation coefficient, *r* Pearson’s correlation coefficient

The searches in the three databases returned 24,802 hits from which 63 studies were included in the analysis (Fig. 1). Among these, 0 studies included only females, 41 focused exclusively on male participants [22, 23, 26, 27, 46, 47, 49, 51–81] and the remaining 22 studies [24, 25, 82–102] included both sexes (Supplementary Table S15).

LPTs were used in 52 studies, including sensors such as SmartCoach [52], GymAware [22, 25, 46, 47, 49, 55, 59, 66, 69, 74, 78, 82, 85, 88, 90], T-force [22, 23, 53, 58, 61, 64, 66, 67, 71, 72, 81, 84, 87, 90, 94, 99, 102], Chronojump [58, 73, 89, 102], Humac [86, 101], Tendo [22, 62, 63, 80, 92, 95, 97], Vitruve [64, 76, 81], Speed4Lift [65, 87, 94], MuscleLab [79, 94], and 1080 Quantum System [85]. Additionally, 28 studies used IMUs, including Gyko [52], PUSH [25, 26, 47, 53, 57, 58, 71, 79, 85], Myotest [54, 56], Bar Sensei [82], EnodePro [24, 25, 65, 74, 83–85, 94], WIMU [61, 99], Jueying [75], IMS [68], Ergonauta [103] and PowerTool [82]. Six studies validated sensors via 3D motion capture systems, e.g., Vicon [51, 74, 84], Qualisys [49], Velowin [58] and OptiTrack [73]. Systems with combined measurements (e.g., LPTs with integrated force measurement, such as the 1080 Quantum System) were classified under the LPT category.

Most studies focused on lower-body exercises such as:

1. Squats [22, 24, 25, 46, 49, 51, 53, 56, 58, 59, 61–68, 70, 71, 73–76, 79, 80, 82, 84–90, 92, 94, 97, 99],
2. Deadlifts [47, 57, 88],
3. Hip thrusts [65] or
4. Jumps [54, 72, 85, 91, 103].

Twenty-three studies were performed for upper body exercises:

1. Bench presses [23, 24, 27, 46, 52, 58, 62, 64, 71, 74, 81, 87, 92, 94–96, 101, 102],
2. Bench press throw [27],
3. Complex exercises such as snatches [83, 85], landmine punch throws [69] and high pulls [77, 78].

#### Methodological Quality, Risk of Bias and Quality of Evidence

The studies achieved a mean score of 12.06 ± 1.27 points, suggesting an overall acceptable to good methodological quality [35] with the highest adapted COSMIN score of 14 [24, 25, 62, 64, 84, 92–94, 101, 103] and the lowest score of 9 [77] (Supplementary Table S17).

#### Validity of Different Velocity Sensors

##### Overall Effects

Device validity (*N* = 11) was evaluated by comparing the velocity sensor against a gold-standard system (Motion capture). Device agreement (*N* = 27) describes the agreement between the velocity sensor and a criterion-reference system without gold-standard status (e.g., LPT, IMU). Validity based on ICC (*n* = 5 studies) [49, 63, 70, 74, 96] demonstrated high validity (ICC = 0.91 [0.87–0.94]) ranging from ICC = 0.65 [74]–0.98 [49]. Additionally, linear association was assessed via Pearson’s *r* in 8 studies [22, 51, 70, 74, 84, 85, 100, 103] (*r* = 0.95 [0.82–0.98]) ranging from 0.55 [22] to 0.99 [85], providing supplementary information on correlation but not on absolute agreement.

Since analytical heterogeneity was evident [*Q*(54)=689.26, *p* < 0.001], subgroup analyses were performed to identify meaningful moderators.

##### Moderator Analysis

Exercise category (*p* = 0.196–0.684), type of gold standard reference (*p* = 0.130–0.143), as well as intensity (*p* = 0.121) did not reach the level of significance.

Velocity variables significantly moderated validity in correlation statistics (*p* < 0.001). However, the beta coefficients for neither peak velocity nor mean velocity were significant (*p* = 0.201–0.538).

Sensor type regression (*p* < 0.001) and close individual study examination indicated differences between IMU and LPT. Although Quantum, as a bar-mounted hybrid sensor, is the only sensor with a significant beta value (*β* = 2.99, *p* = 0.002) and correlations of *r* = 0.97–0.99, a wide range of validity outcomes was reported with 7 out of 8 IMU studies [22, 51, 70, 74, 84, 85, 96, 100] providing a range of *r* = 0.40–0.99 [22, 51, 70, 74, 84, 85, 100] and ICC = 0.65–0.96 [70, 74, 96]. Most studies indicated moderate to good validity with *r* = 0.55–0.61 [22] in the ballistic squat velocity for the Myotest IMU or *r* = 0.40–0.83 for the EnodePro [84]. In contrast, LPTs showed validity values with *r* = 0.77–0.99 and ICC = 0.87–0.98 [22, 49, 63, 85, 96, 103].

#### Device Agreement

##### Overall Effects

Across 439 effect sizes (*N* = 10 [24, 62, 64, 73, 79, 80, 82, 86, 92, 101]) device agreement showed good-to-excellent pooled ICC = 0.92 [0.83–0.97] between sensors, individual study estimates ranged from poor to excellent (ICC = 0.19 [52]–0.99 [24, 58, 64, 87, 92, 94]). Lin's CCC was reported in 3 studies [64, 67, 80] with CCC = 0.90 [0.52–0.98]. The lowest CCC was found with CCC = 0.36 [80] and the highest CCC = 0.99 [67]. As Pearson’s *r* reflects association rather than agreement, it was treated as a secondary metric. Pearson correlations were reported in 19 studies [24, 47, 52, 53, 57, 61, 65, 68, 71, 74–77, 79, 81, 84, 89, 92, 104] and indicated a pooled strong linear association (*r* = 0.91 [0.84–0.95]). However, individual studies ranged from *r* = 0.2 [52] to *r* = 0.99 [24, 77, 81, 92].

**Fig. 2:**
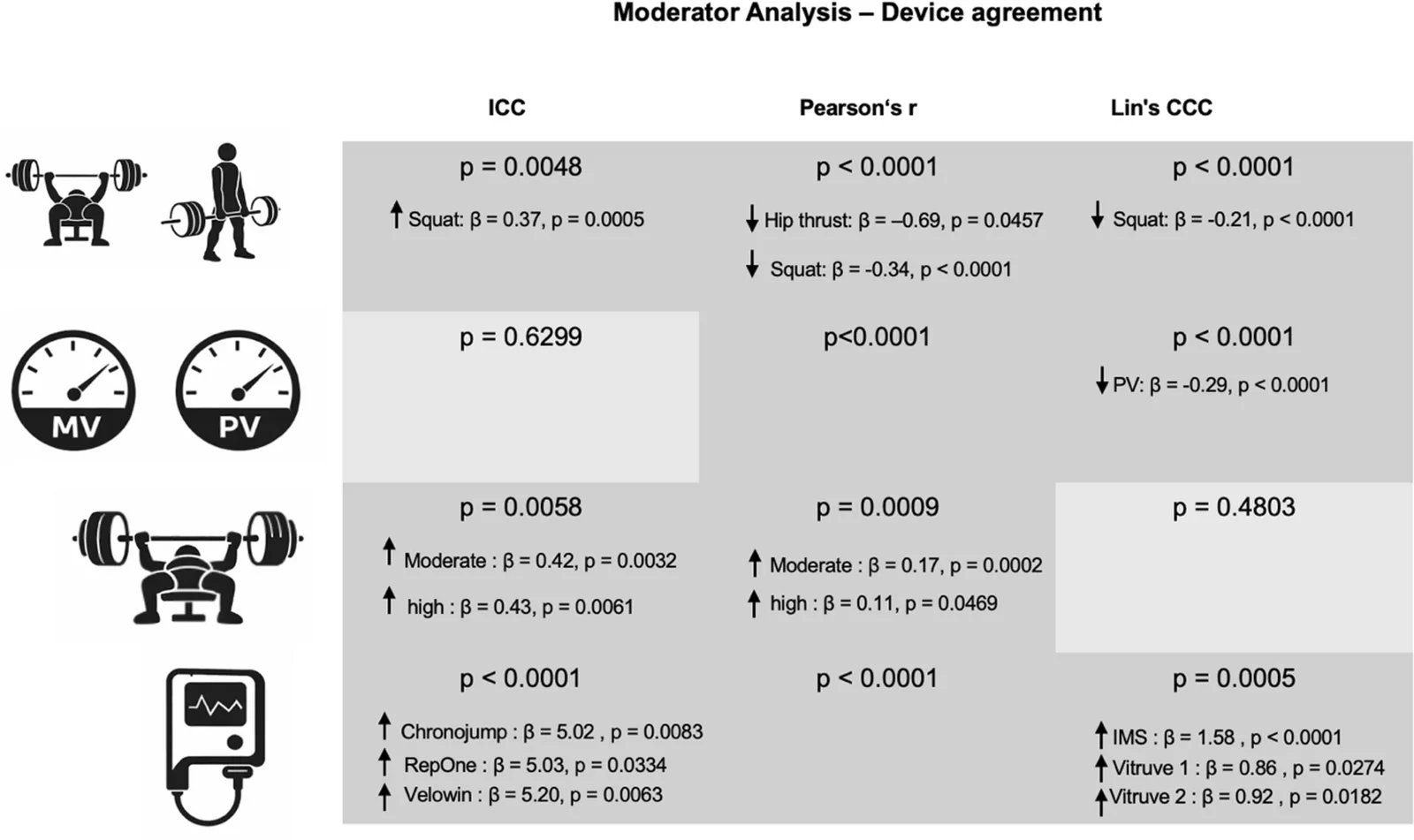
Moderator analyses for device agreement. Results are shown separately for ICC-based, Pearson’s *r*-based and Lin’s CCC-based agreement analyses. Rows represent the investigated moderators (exercise type, velocity variable, training intensity, and sensor type). Shaded cells indicate statistically significant moderator effects based on the QM test (*p* < 0.05). Specific moderator levels are displayed only when the analysis reached statistical significance. Directional arrows summarize the direction of significant effects. *ICC* intraclass correlation coefficient, *r* Pearson’s correlation coefficient, *Lin’s CCC* Lin’s concordance correlation coefficient, *p* corresponding significance level of the regression coefficient, *β* estimated meta-regression coefficient: positive *β* values indicate higher ICC, *r* or CCC values relative to the reference category, whereas negative *β* values indicate lower values

Since analytical heterogeneity was evident [ICC: *Q*(438) = 1317.53, CCC: *Q*(63) = 1083.51, *r*: *Q*(226) = 1550.48, all *p* < 0.001], subgroup analyses were performed to identify meaningful moderators (Fig. 2).

##### Moderator Analysis

Velocity variable (analyzed with ICCs; *p* = 0.630) and intensity (analyzed with Lin’s CCC, *p* = 0.480) did not reach the level of significance.

In contrast, in the ICC-based analysis, squat-based assessments were associated with higher agreement estimates (*β* = 0.37, *p* < 0.001, *n* = 13) with excellent ICC values for the T-Force-Tendo LPT comparison (ICC = 0.90) [62], and the RepOne sensor vs Tendo LPT (ICCs = 0.83–0.99). However, Lin’s CCC indicated lower concordance for squat movements (*β* = −0.21, *p* < 0.001, *n* = 3), and Pearson-based analyses showed a similar reduction in linear association (*β* = −0.34, *p* < 0.001, *n* = 6).

Additionally, the variable is a significant moderator in Lin’s CCC and Pearson analyses. Peak velocity (*β* = − 0.29, *p* < 0.001, *n* = 3) showed reduced concordance with CCC = 0.59–0.99 when IMU compared to T-force [64, 67], while mean velocity did not reach statistical significance. The Pearson-based device agreement model reinforced this pattern, with all velocity variables tending toward lower correlations, although none of the individual beta coefficients reached statistical significance.

Training intensity significantly moderated device agreement in both the ICC and Pearson-based analyses. The analysis revealed that agreement was significantly higher at moderate (ICC: *n* = 10; *r*: *n* = 6) and high (ICC: *n* = 4; *r*: *n* = 2) intensities compared with low intensities. Good to excellent correlations were found at moderate intensities (ICC = 0.87–0.99, *r* = 0.81–0.99) between RepOne and Tendo [92], indicating the best correlation at 60%1RM with *r* and ICC = 0.99. At high intensities, correlations remained high between these sensors [92] (ICC = 0.87–0.99), while for low intensities agreement was lower (ICC = 0.79–0.99) [92].

Sensor type significantly moderated device agreement in ICC- and Lin’s CCC-based analyses. Certain LPTs [58, 92] demonstrated significantly higher agreement with T-force or Tendo (ICC = 0.82–0.99). In Lin’s CCC-based analysis, IMS and Vitruve showed moderate-to-high concordance with T-force IMU (CCCs = 0.58–0.99) [64, 67]. Pearson-based regression revealed Gyko IMU (*β* = − 1.43, *p* = 0.0366, *n* = 1) with significantly lower agreement with smartcoach LPT; however, this finding is based on a single study. For detailed moderator analysis, see Fig. 2.

#### Reliability of Velocity Measurements Obtained Using Different Velocity Sensors

##### Overall Effects

For interday reliability, 29 studies reported ICCs [25, 27, 46, 54–56, 58–60, 62, 65, 67, 69, 72, 75, 78, 80, 82–84, 86, 88, 90, 92, 95, 97–99, 101]. The pooled estimate indicated good-to-excellent reliability with ICC = 0.90 [0.85–0.93]; however, individual study estimates ranged widely from poor to excellent (ICC = 0.14 [56]–0.99 [25, 58, 98]. One study reported Lin´s CCC [25] with CCC = 0.99. For interday reliability, Pearson correlations were reported in *N* = 3 studies [65, 69, 98] and were treated as supplementary association metrics rather than primary reliability evidence (*r* = 0.91 [0.38–0.99]) with a range of *r* = 0.55 [65] to 0.99 [98].

Thirteen intraday studies [23, 26, 53, 65, 66, 69, 83, 84, 91, 93, 101–103] indicated good-to-excellent reliability (ICC = 0.91 [0.87–0.95]) with individual studies ranging from 0.29 [23] to 0.99 [103]. One study [65] additionally reported Pearson’s correlation coefficient (*r* = 0.89 [0.83–0.93]).

Since analytical heterogeneity was evident for Interday reliability [ICC: *Q*(607) = 950.13, CCC: *Q*(5) = 30.61], [Pearson’s *r* *Q*(43) = 232.07); all *p* < 0.001], as well as for intraday ICCs [*Q*(227) = 268.22, *p* = 0.032], subgroup analyses were performed to identify meaningful moderators.

##### Moderator Analyses

*Intraday:*

In the intraday analysis, no moderator effect could be found for exercise, velocity variable, or intensity (*p* = 0.059–0.684). Only sensor type significantly moderated intraday reliability (Table 1) when assessed with ICC (*p* = 0.001). The analysis revealed that only the Beast sensor (*β* = − 2.13, *p* = 0.004, *n* = 1), demonstrated significantly lower reliability, whereas no other sensor showed statistically significant differences.

**Table 1:** Sensor-specific range of velocity sensor reliability across interday and intraday analyses with significant beta values in italics

Sensor	Interday_ICC	Interday_*r*	Intraday_ICC
LPT			
Tendo	0.56–0.99	–	0.65–0.97
GymAware	0.54–0.99	*0.60–0.92*	0.61–0.92
Quantum	0.58–0.97	–	–
Chronojump	0.98–0.99	–	0.72–0.97
HUMAC	0.45–0.99	–	0.91–0.97
Speed4Lifts	0.56–0.75	*0.56–0.75*	0.81–0.97
PowerLift	–	–	0.74–0.87
ADR	0.94–0.99	0.90–0.99	0.75–0.96
Velowin	0.98–0.99	–	0.68–0.83
IMU			
PUSH	0.38–0.99	–	0.46–0.98
Beast	–	–	*0.29–0.64*
Myotest	0.14–0.96	–	–
WIMU	0.91–0.98	–	–
Bar Sensei	0.17–0.45	–	–
PowerTool	0.65–0.79	–	–
EnodePro	0.55–0.99	*0.55–0.81*	0.58–0.94
Jueying	0.79–0.95	–	–
ReoOne	0.59–0.98	–	–
Other			
Vicon (MoCap)	0.69–0.91	–	0.61–0.99
T-force (LVT)	0.7–0.99	–	0.77–0.99

*ICC* intraclass correlation coefficient, *r* Pearson’s correlation coefficient, *LPT* linear position transducer, *IMU* inertial measurement unit, *LVT* linear velocity transducer

*Interday:*

Intensity (ICC: *p* = 0.455) did not reach the level of significance and could not be conducted for Lin’s CCC and Pearson’s *r* analyses.

For interday reliability assessed using Lin’s CCC, the velocity variable showed a significant moderating effect (*p* = 0.0002), with peak velocity being significantly lower (*β* = −0.44, *p* < 0.001, *n* = 1) for measurement with GymAware, PUSH, and EnodePro [25]*.*

When Pearson’s *r* was considered as a supplementary association metric for interday reliability, exercise category showed a significant moderating effect (*p* < 0.001). Lower between-day correlations were observed for hip thrust (*β* = −2.02, *p* < 0.001, *n* = 1), landmine punch throw (*β* = −1.58, *p* < 0.001, *n* = 1), and squat (*β* = −1.59, *p* < 0.001, *n* = 1). For the squat, correlations were moderate for Speed4Lifts (*r* = 0.75) and EnodePro (*r* = 0.81) [65]. Lower associations were observed for the hip thrust (Speed4Lifts: *r* = 0.56; EnodePro: *r* = 0.55) [65], and landmine punch throw for GymAware (*r* = 0.60–0.92) [69].

Sensor type significantly moderated reliability outcomes in all analyses, except for the intraday analysis based on Pearson’s *r* (*p* = 0.1153), with PUSH (*β* = − 0.51, *p* < 0.001, *n* = 1), EnodePro (*β* = − 1.77, *p* < 0.001, *n* = 1), and GymAware (*β* = − 1.58, *p* < 0.001, *n* = 1) being sensors with the poorest reliability. As each estimate was derived from a single study, these findings should be regarded as exploratory. Twenty-one studies were conducted using LPTs ([25, 46, 55, 58–60, 62, 65, 67, 69, 78, 80, 83, 86, 88, 90, 92, 95, 97, 98, 101] and 13 studies [25, 54, 56, 58, 75, 78, 80, 82–84, 92, 99] on IMUs. A linear velocity transducer (LVT) is represented in all studies by the T-Force sensor [27, 58, 67, 72, 84, 95, 99]. Table 1 summarizes all sensors and reports the range of study-level effect sizes for each analysis across studies, exercises, and intensities.

### Results Part II: 1RM Prediction Models

#### Risk of Publication Bias

Funnel plots for all primary meta-analyses can be reviewed in the Supplementary Figures S12–S14.

#### Search Results

**Fig. 3:**
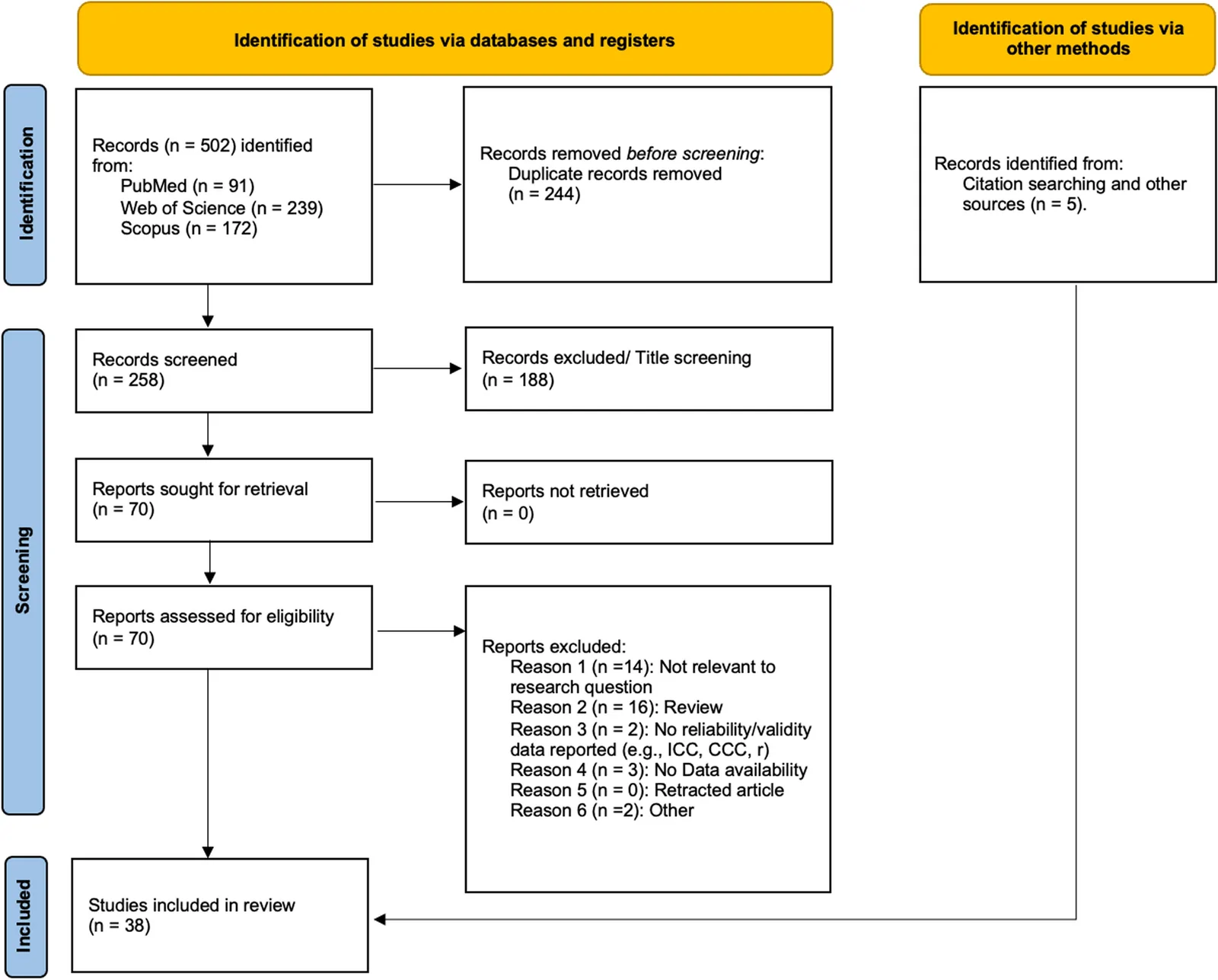
PRISMA flowchart of the literature search and study selection process for Part II: reliability and validity of velocity-based 1RM prediction models. *ICC* intraclass correlation coefficient, *CCC* Lin’s concordance correlation coefficient, *r* Pearson’s correlation coefficient

Figure 3 displays the flowchart of the literature search and selection process for the 1RM predictions that returned 502 hits from which 38 studies were included.

Among these, 2 studies included only females [15, 105], 25 focused exclusively on male participants [7, 8, 11, 14, 16, 18–21, 106–121] and the remaining 10 studies included both sexes [118, 122–130] (Supplementary Table S15). One study [131] did not explicitly report the sex of the participants.

The majority of studies (*N* = 37) applied linear load–velocity (LV) regression [7, 8, 14, 15, 18–21, 23, 57, 105–116, 118–132], five studies used polynomial regression [11, 20, 111–113], one study used quadratic regression [8], and one study employed multilinear regression [14]. One study applied regularized regression methods [14] and another study [14] implemented artificial neural networks. Across the studies included, a variety of exercises were used for 1RM prediction. Lower-body exercises were used in 25 studies:

1. Squats [7, 8, 15, 18, 19, 21, 109, 112, 122, 124, 127, 129, 132],
2. Deadlift [11, 25, 57, 105, 107, 114, 116, 132],
3. Snatch pull [128]
4. Knee extension [106, 129],
5. Ankle dorsiflexion, seated ankle plantarflexion, standing ankle plantarflexion, hip abduction, hip adduction, hip extension, hip flexion, and knee flexion [129].

Upper-body exercises were used in 22 studies:

1. Bench press throw [131],
2. Power clean [108, 121],
3. Prone bench pull [110, 125],
4. Bench press [14, 15, 18, 19, 111, 113, 115, 117–120, 123, 126, 127],
5. Horizontal pull [18] and lat pull [118],
6. Seated cable row [118]
7. Overhead press [130].

#### Methodological Quality, Risk of Bias and Quality of Evidence

The studies achieved a mean adapted COSMIN score of 12.24 ± 1.38 points, with a maximum of 15 points [27, 107] and the lowest score of 10 [8, 19, 105, 106, 116, 133]. A detailed table of methodological quality and study characteristics is provided in the supplementary materials (Table S16).

#### Reliability of Prediction Models

##### Overall Effects

**Fig. 4:**
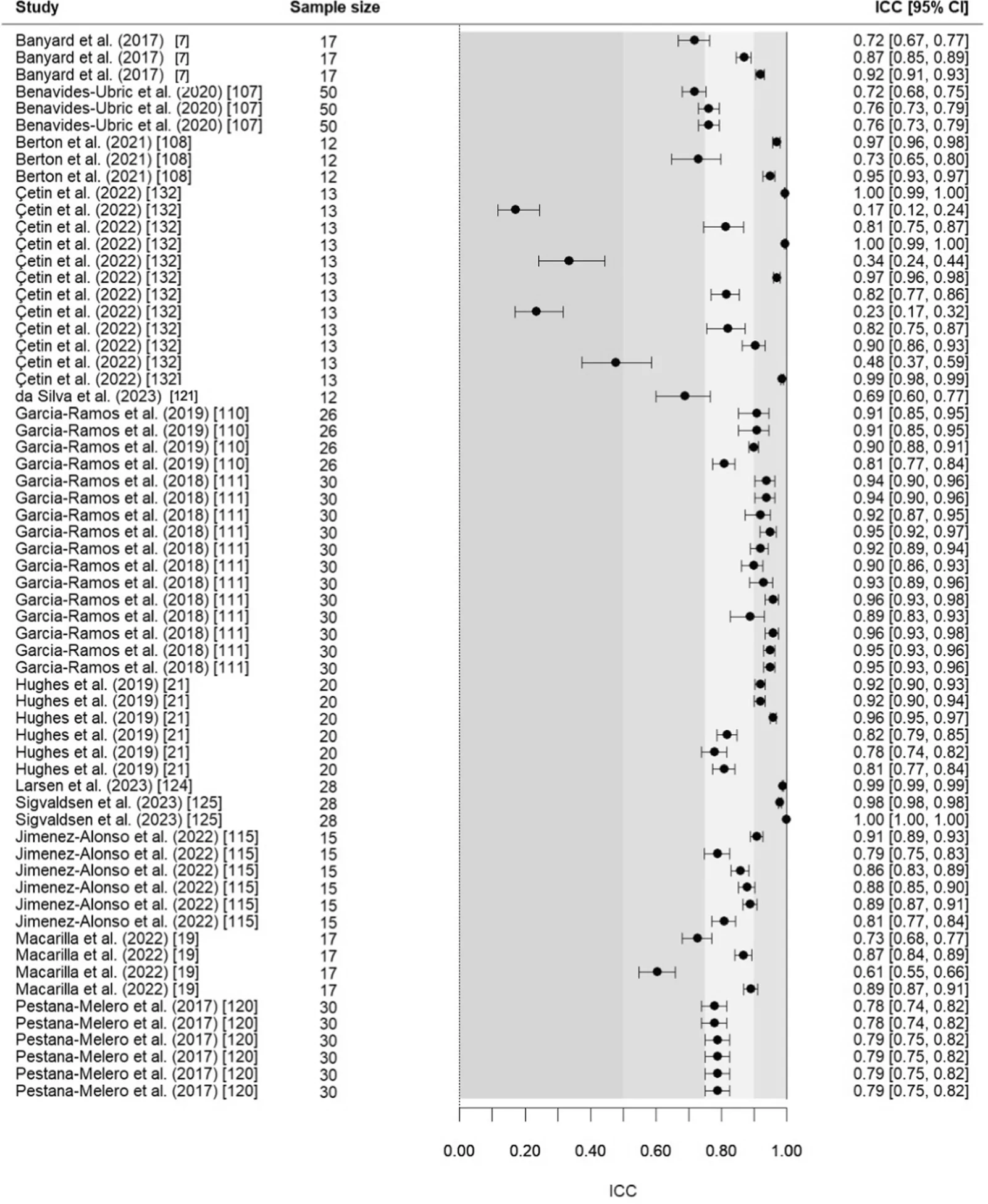

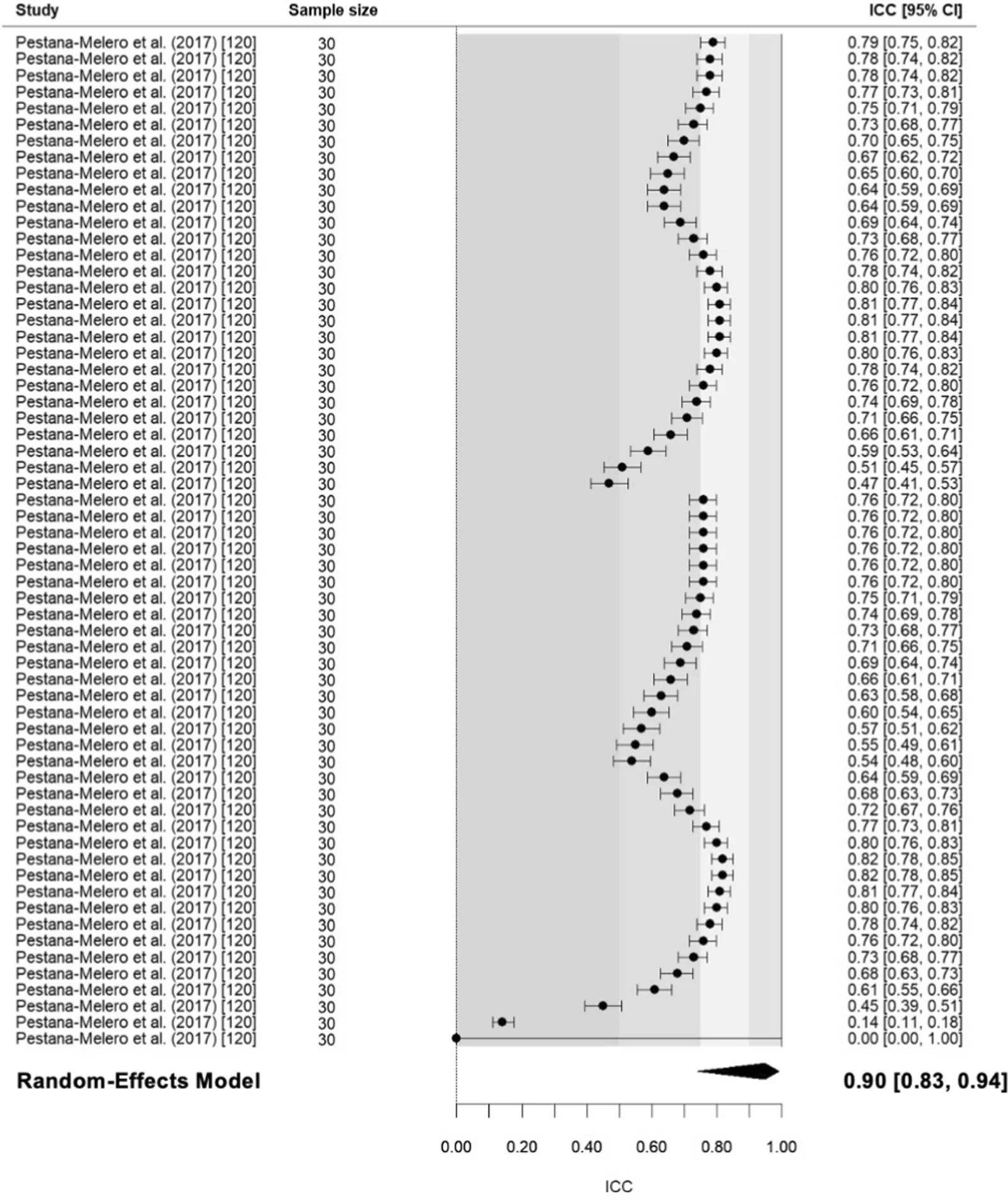
Forest plot of interday reliability estimates for velocity-based 1RM prediction models. Each point represents an individual study-level ICC estimate, with horizontal lines indicating the 95% confidence interval. Values closer to 1.00 indicate higher relative reliability, whereas lower values indicate poorer test–retest stability. The pooled random-effects estimate summarizes the overall interday reliability across included effects but should be interpreted in light of the wide dispersion of individual study estimates and substantial heterogeneity. *ICC* intraclass correlation coefficient, *CI* confidence interval

Thirteen studies [7, 19, 21, 107, 108, 111, 115, 120, 121, 124, 125, 132, 134] reported good-to-excellent interday reliability with pooled ICC = 0.90 [0.83–0.94], while individual study ICCs ranged from 0.14 [120] to 0.99 [124, 132] (Fig. 4).

Since analytical heterogeneity was evident [*Q*(123) = 7136.71; *p* < 0.001], subgroup analyses were performed to identify meaningful moderators.

##### Moderator Analysis

Moderator analyses did not identify any statistically significant influence of prediction method (*p* = 0.750) or used velocity (*p* = 0.205).

In contrast, exercise type substantially influenced prediction reliability (*p* < 0.001), with squat predictions showing lower reliability (*β* = −1.28, *p* < 0.001, *n* = 5) when using the LV method to predict 1RM. ICCs ranged between 0.61 [7, 19] and 0.96 [21] for multiple load points and ICC = 0.24–0.99 [132] for the two-point prediction model [129]. In contrast, other exercises did not show statistically significant deviations (all *p* = 0.079–0.768). Bench press–based predictions demonstrated moderate-to-excellent interday reliability for single-point approaches (ICCs = 0.64 [120]–0.91) [115] and ICC = 0.92–0.95 [111] and ICC = 0.89–0.96, respectively, for two-point or polynomial models [111, 120].

#### Validity of Prediction Models

##### Overall Effects

Three studies [105, 122, 131] used the ICC and yielded moderate-to-excellent relative reliabilities for agreement (ICC = 0.91 [0.72–0.98]) ranging between ICC = 0.68 [131] and 0.98 [105]. Pearson correlations were used in 31 studies [7, 9, 11, 14–16, 18, 19, 25, 107, 109, 111–120, 123–130, 135, 136] with an overall excellent pooled correlation (*r* = 0.96 [0.94–0.97]); however individual studies ranged between 0.44 [109] and 0.99 [8, 14, 113, 117, 118, 126, 127, 133].

Since analytical heterogeneity was evident [ICC: *Q*(8) = 914.14; *r*: *Q*(187) = 1472.50, *p* < 0.001], subgroup analyses were performed to identify meaningful moderators.

##### Moderator Analysis

In the validity analysis, no moderator effect was evident for the velocity variable (mean vs peak velocity) (*p* = 0.486).

In contrast, exercise type significantly influenced 1RM prediction in both analyses (*p* < 0.001), with squat-based predictions (*β* = −0.98, *p* < 0.001, *n* = 9) [7, 8, 15, 18, 19, 109, 112, 127, 129] and deadlift predictions (*β* = −0.94, *p* < 0.001, *n* = 7) [11, 16, 20, 107, 114, 116, 126] demonstrating lower validity. Some upper-body pulling exercises were conducted in 1 study [118] and exhibited stronger associations, including the lat pull (*β* = 0.51, *p* = 0.003, *n* = 1) and cable row (*β* = 0.47, *p* = 0.005, *n* = 1). Effect sizes for each exercise (if *n* > 2) are shown in Table 2 with significant β values in italics.

**Table 2:** Exercise-specific range of validity across 1RM prediction analyses with significant beta values in italics

Exercise	ICC	r
Squat	–	*0.37–0.99*
Deadlift	*0.97–0.98*	*0.60–0.98*
Bench press throw	0.68–0.97	–
Knee extension	–	0.84–0.96
Bench press	–	0.50–0.99

*ICC* intraclass correlation coefficient, *r* Pearson’s correlation coefficient

Additionally, the prediction method significantly moderated the validity estimates based on Pearson’s *r* (*p* < 0.001), with polynomial regression performing significantly worse than other methods (*β* = −0.3941; *p* < 0.001, *n* = 5) [11, 20, 111–113] and showing moderate-to-high correlations of *r* = 0.85–0.98 [111] (one-point) and *r* = 0.78 [112]–0.98 [113] (multipoint). Most studies (*n* = 21) applied multipoint LV regression models, which yielded a wide range of correlations (*r* = 0.40 [109]–0.99 [14, 113]). Two-point models (*n* = 13) demonstrated moderate-to-excellent correlations (*r* = 0.50–0.99 [113]), while single-point (*n* = 3) approaches generally showed moderate-to-excellent correlations (*r* = 0.60 [116]–0.95 [124]). Quadratic regression models (*r* = 0.98–0.99 [8]) were included in fewer studies (*n* = 4) but demonstrated consistently excellent validity.

## Discussion

This systematic review synthesized evidence from 63 articles to meta-analyze the currently available validity and reliability of commercially available velocity-based measurement systems (Part I) and the accuracy of velocity-based 1RM prediction approaches applied in resistance training exercises (Part II).

In part I, 11 studies indicated high to excellent validity (ICCs = 0.91 [0.87–0.94], *r* = 0.95 [0.82–0.98]. More studies (*n* = 27) compared velocity tracking with other sensors, showing ICC = 0.92 [0.83–0.97], Lin’s CCC = 0.90 [0.52–0.98]. Pearson’s *r* was additionally reported (*r* = 0.91 [0.84–0.95]) as a supplementary association metric, which does not assess agreement. Forty-seven articles (*N* = 47) evaluated reliability facets, indicating good-to-excellent measurement consistency in 13 intraday reliability studies (ICC = 0.91 [0.87–0.95], *r* = 0.89 [0.83–0.93] and 29 studies on interday reliability metrics with ICC = 0.90 [0.85–0.93], LIN’s CCC = 0.99 [0.99–0.99] and Pearson’s *r* = 0.91 [0.38–0.99]. Most studies reported relative reliability, while only 12 studies quantified systematic and/or random measurement errors [23, 25, 46, 53, 62, 65, 66, 91, 93, 102, 103].

Part II comprised 38 studies evaluating the accuracy of the 1RM prediction via different methods (e.g., LV regression). Between-day reliability (*N* = 13) showed good-to-excellent ICCs = 0.90 [0.83–0.94], with 8 studies providing detailed measurement error analyses [19, 107, 110, 115, 120, 124, 125, 132]. Validity was quantified via ICCs and Pearson’s *r*, showing ICC = 0.91 [0.72–0.98] (*N* = 3) and *r* = 0.96 [0.94–0.97] (*N* = 31), also focusing on relative reliabilities.

### Validity of Velocity-Based Measurement Systems (Part I)

Despite high pooled validity estimates (ICC = 0.91, *r* = 0.95), substantial heterogeneity was observed across studies. The wide range of correlation coefficients and analysis heterogeneity within IMU-based systems (ICC = 0.65–0.96, *r* = 0.40–0.99) indicates that validity is strongly sensor-dependent rather than uniformly high across technologies. In contrast, LPT-based systems demonstrated more consistent validity estimates with lower variability (ICC = 0.87–0.98, *r* = 0.77–0.99). It can be speculated whether the lower validity of IMU-based systems was attributable to indirect acceleration integration, which is more sensitive to signal noise, drift, and calibration assumptions. LPTs calculate speed directly from displacement over time, which likely contributes to their more consistent validity across different studies [24].

Relative reliability metrics should be interpreted cautiously in sensor-validation studies because they reflect rank-order consistency within a sample rather than measurement agreement with the reference device [137]. Validity assessment therefore requires quantifying differences between the new sensor and the reference standard using agreement analyses [138].

#### Measurement Error Analysis

Relative validity coefficients quantify the strength of association between devices rather than the magnitude of measurement error. Consequently, high ICC or Pearson's *r* values do not necessarily imply that two sensors can be used interchangeably in practice. For this purpose, agreement analyses and absolute measurement error metrics are required. Few authors included agreement analyses in their validity evaluations [22, 49, 51, 63, 70, 74, 84, 85, 96, 100, 103], and many of those who did provided insufficient or erroneous interpretations. Agreement between sensors should be interpreted by relating the device-to-device differences to the corresponding paired mean velocities [138]. Accordingly, a systematic bias between the gold standard measure and the new device can be interpreted as an over- or underestimation of velocity, that could be attributed to different underlying calculation models. Several studies provided limited agreement information. Two studies presented Bland–Altman plots only qualitatively without numerical agreement indices [51, 70], while Olaya-Cuartero et al. [100] interpreted the finding that “most of the data are between the upper and lower LoA” as evidence of agreement. However, this is not an adequate interpretation of Bland–Altman analyses, as the LoA are constructed to include approximately 95% of observed differences [30]. Two studies [84, 96] reported numerical LoA incompletely, as these were not described in reference to the respective means, complicating the interpretation of the analysis.

Although several studies concluded that the sensors were valid, high correlation or ICC values alone do not demonstrate acceptable agreement. When interpreted relative to mean velocity, the reported LoA indicated potentially meaningful device-to-device differences, ranging from −0.32 to 0.41 m s⁻^1^ at 1.03 ± 0.10 m s⁻^1^ [84] and from 0.022 to 0.410 m s⁻^1^ at 0.510 ± 0.244 m s⁻^1^ [96]. Only Askow et al. [49] reported relative LoA (− 11.6 to 7.1%) for GymAware versus Qualisys, allowing scale-independent interpretation [80]. LoA appeared narrower for LPTs than for IMUs, although this comparison remains descriptive because protocols and reference systems differed. Tendo showed LoA of −0.141 to 0.099 m·s⁻^1^ versus 3D motion capture [96] and −0.15 to −0.07 m·s⁻^1^ versus Optotrak [63], whereas Gheller et al. [103] reported mean bias only for EnodePro versus Vicon. The LoAs quantified a random scattering from unsystematic influences such as inter-individual standardization limitations (e.g., inappropriate sensor placement or unsystematic mathematical model problems). Appropriate reporting and interpretations of agreement analyses are urgently needed for a comprehensive practical recommendation.

Future validation studies should therefore complement relative validity analyses with standardized agreement and measurement error reporting to improve the practical interpretation of sensor performance.

### Device Agreement Between Velocity Measurement Systems (Part I)

It is noteworthy that some authors considered LPT sensors such as GymAware and T-Force as the gold standard [52, 61, 74, 81, 94, 104]: *“LPT systems are considered the gold standard for measuring barbell velocity… [.]”* (p.6) [61]. Definitions and terms, as well as awareness of the difference between validity and device objectivity, were blurred and must be reconsidered. In the presented analysis, these studies were in the device agreement section, as they compared different velocity sensors against each other.

Twenty-three studies revealed good-to-excellent correlation metrics (*r* = 0.91 [0.84–0.95], ICCs = 0.92 [0.83–0.97]. Only 3 studies [64, 67, 80] evaluated agreement via CCC = 0.90 [0.52–0.98]. Increased device agreement variability (*Q* > 1050, *p* < 0.001) might be attributable to the lack of gold standard measurements, with both sensors showing individual and specific random and systematic errors. Sensor type, used exercise, intensity or velocity profile influenced results. Comparisons between LPT-based systems and LPT to T-force LVT generally demonstrated moderate-to-excellent agreement (ICC = 0.79–0.99) [92], with sensors such as Chronojump and Velowin showing significantly higher agreement with T-force [58]. IMU to LPT comparisons yielded *r* = − 0.20–0.99 [24, 47, 57, 65, 74–76, 79, 92], ICC = 0.19–0.99 [24, 65, 74, 79, 80, 92], and CCC = 0.36–0.80 [80], while IMU to LVT showed correlations with *r* = 0.41–0.97 [53, 61, 84, 104], ICC = 0.00–0.98 [58, 64, 87, 94] and CCC = 0.61–0.99 [64, 67]. IMU-to-IMU comparisons yielded ICC = 0.30–0.56 [82], questioning the interchangeability of sensors. However, these findings should be regarded as exploratory rather than interpreted as evidence of superior measurement performance of these sensors, as the comparisons partly stem from a single study. In contrast, comparisons between EnodePro and Tendo reported lower CCCs [76], further highlighting that these effects likely reflect study-specific characteristics rather than generalizable differences in sensor quality. Analyses of squat exercises [24, 75, 76, 89] support this finding: whereas RepOne showed a relatively narrow ICC range (0.83–0.99), EnodePro exhibited a substantially wider range (0.28–0.99), reflecting pronounced inconsistency across measurement conditions. This could suggest that high ICC values observed for the squat may be driven more by sensor-specific characteristics than by uniformly high agreement across systems. In contrast, upper-body exercises such as the bench press demonstrate considerably high correlation values (*r* = 0.84–0.99), as reported for EnodePro [24] and RepOne [92], which introduces uncertainty regarding the sources of measurement error. Given the large number of potential influencing variables, it remains difficult to determine whether poorer device agreement is primarily attributable to sensor characteristics, exercise-specific factors, or their interaction. Exercise-specific effects are supported by Behrmann et al. [24], who reported a significantly smaller error range for bench press velocity measurements (MAPE = 3.29–8.79%) compared with squat measurements (MAPE = 8.28–29.90%).

Also, intensity or movement velocity moderated results, with agreement being significantly higher at moderate (50–80%1RM) and high intensities (> 80%) compared with low intensities (< 50%). Especially for IMU-based systems compared with T-force or LPTs, the results show a wide range with partly very low for low intensities (*r* = 0.19–0.99, ICC = 0.37–0.99) [24, 52, 57, 61, 71, 75, 76, 80, 82, 84]. Additionally, Behrmann et al. [24] reported LoA for bench press velocity at 70%1RM (−0.043 to 0.095 m s⁻^1^, mean velocity 0.50 ± 0.14 m·s⁻^1^), whereas markedly wider LoA were observed at 30%1RM (−0.109 to 0.246 m s⁻^1^, mean velocity 0.94 ± 0.19 m·s⁻^1^). Similarly, Warneke et al. [80] reported MAPE between IMU and LPTs ranging from 9 to 11% at low squat intensities and 5.6–7.3% at moderate-to-high intensities.

#### Measurement Error Analysis

In contrast to correlation-based validity measures, agreement analyses directly quantify whether different devices provide interchangeable measurements by accounting for systematic bias and absolute differences between sensors. Consequently, agreement metrics such as Lin's CCC and Bland–Altman analyses are particularly relevant for practitioners intending to replace or combine measurement systems. However, comparatively few studies reported these analyses, limiting firm conclusions regarding device interchangeability. As for validity, some studies determined agreement via LoA or bias values, but without providing corresponding mean velocities or relative error metrics and without stratifying results by exercise or intensity [52, 58, 61, 65, 71, 72, 74, 76, 79, 81, 84, 87, 89, 92]. Most studies that used agreement analyses for reliability evaluations failed to report systematic bias and numerical LoA [57, 77, 82, 94] with one study even failing to report the mean velocity [74]. Only a few studies evaluated the systematic bias. González-Galán et al. [64] reported mean biases ranging from 2.8 to 13.4% when comparing Vitruve LPT with T-Force. Pérez-Castilla et al. [71] observed small absolute biases of 0.06–0.07 m s⁻^1^ across mean velocities of 0.48–1.28 m s⁻^1^, while Martínez-Cava et al. [87] reported biases between −0.02 and 0.03 m s⁻^1^ across velocities of 0.7–1.15 m s⁻^1^. In studies directly comparing LPT sensors, only two reported BA analyses with sufficient detail: Illera-Domínguez et al. [89] identified a substantial negative mean bias of −0.119 m s⁻^1^ at relatively low velocities (0.38–0.55 m·s⁻^1^), whereas Lawson et al. [92] reported positive biases ranging from 0.039 to 0.110 m s⁻^1^ across a broader velocity range (0.42–1.52 m s⁻^1^). An appropriate random error analysis was only found/rarely reported in 2 studies [80, 93].

### Reliability (Part I)

#### Interday

Overall interday reliability revealed ICCs = 0.90 [0.85–0.93] (*N* = 29) and *r* = 0.91 [0.38–0.99] (*N* = 3) with a range between ICC = 0.14 and 0.99. This wide range of ICCs might be attributable to different methodological constrains. Koo and Li [42] recommended *n* ≥ 30 as a rule of thumb to adequately interpret ICCs. While this recommendation is based on robustness of analyses of variance effects with sample sizes exceeding *n* = 30, larger sample sizes of *n* > 150 were required for stable ICC estimates [30]. Reliability analyses with *n* < 50 were considered “pilot data” [29]. Out of the included studies, only four studies met the criterion from Koo and Li [54, 62, 67, 97], and only one study reached a sample size of *n* > 50 [25], showing that instable reliability metrics could be attributable to comparatively small sample sizes.

A significant moderator for interday reliability was the sensor type (*p* < 0.001), with ICC = 0.44–0.99 for LPTs, ICC = 0.70–0.99 for T-force LVT and ICC = 0.14–0.99 for IMUs. Although the ICC-based meta-regression did not yield statistically significant *β* coefficients for individual sensors, several individual studies reported very low ICC values for specific IMU-based systems, including the Myotest (ICC = 0.14), Bar Sensei (ICC = 0.17), and the PUSH sensor (ICC = 0.38). The performed regression analysis showed EnodePro (*β* = − 1.77) and GymAware (*β* = − 1.58) to be sensors with the poorest reliability when Pearson ‘*r *was calculated. Since regression analyses are impacted by sample size per potential moderator, the comparatively small study number might bias the regression analysis, and individual study results should be considered carefully.

#### Intraday

Intraday reliability reflects the consistency of velocity measurements obtained within a single testing session and therefore represents the lowest threshold for protocol reliability. Despite good-to-excellent reliability metrics (ICC = 0.91 [0.87–0.95], *r* = 0.89 [0.83–0.93]), values ranged widely from poor to excellent (ICC = 0.29–0.99) [23, 26, 53, 65, 66, 69, 83, 84, 91, 93, 101–103]. To explain the observed variability, meta-regression identified only sensor type as a significant moderator (*p* = 0.001), with the Beast IMU sensor (*β* = − 2.13, *p* = 0.004) exhibiting significantly lower reliability, with ICC = 0.29–0.64 [23]. Studies employing LPTs (e.g., GymAware, Chronojump) [23, 65, 66, 69, 93, 101, 102] or T-force LVT [9, 23, 66, 84, 93, 102] demonstrated ICC = 0.61–0.99, indicating LPTs or LVTs to be more reliable than IMU sensors.

#### Measurement Error Analysis

Only eight studies reported CV (1.22–54.9%) [23, 53, 66, 91, 93, 102], TE (1.02–2.46) [103] or MAE (0.03–0.64) [93]. From a practical perspective, coaches and practitioners are often more interested in the expected measurement error than in relative reliability alone, particularly when monitoring small performance changes over time. Therefore, reliability studies should also complement ICC-based analyses with absolute reliability measures and standardized measurement error reporting to facilitate practical interpretation [139]. When interpreting reliability and repeatability quantifications, it should be noted that not sensor reliability, but rather a combination of sensor reliability limitations and biological variability could cause outlined random errors. A pure sensor evaluation would exclude participants and use (for instance) a machine that moved the sensor with a prescribed velocity. Since all studies evaluated reliability in exercise performed by humans, repeatability errors were caused by the training individual and/or sensor limitations. Therefore, in contrast to systematic sensor over- or underestimations quantified in measurement analyses for validity or device agreement, in test–retest reliability, systematic errors could also be attributed to learning or fatiguing effects of the exercising individual. Reliability estimates from repeated exercise trials may reflect both device error and biological variability and should not be interpreted as device reliability alone [30]. Exercise-specific differences may also reflect habituation demands, particularly in complex multi-joint tasks and untrained participants [140, 141].

### 1RM Predictions (Part II)

#### Validity of 1RM Predictions

Based on 38 studies, velocity-based 1RM prediction showed high relative validity estimates with Pearson's *r* = 0.96 [0.94–0.97] (*N* = 32) [7, 9, 11, 14–16, 18, 19, 25, 107, 109, 111–120, 123–130, 134–136]. High heterogeneity [ICC: *Q*(8) = 914.14; *r*: *Q*(197) = 1492.73] could be explained by exercise (*p* < 0.001), variable (*p* = 0.046), and regression model (*p* < 0.001) being significant moderators. Squat and deadlift predictions demonstrated lower validity, with correlation coefficients ranging from *r* = 0.37–0.99 for squats [7, 8, 15, 18, 19, 109, 112, 127, 129] and *r* = 0.60–0.98 for deadlifts [11, 16, 20, 107, 114, 116, 126]. Besides this high variability, Warneke et al. [80] observed mean random over- and underestimations during free-weight squats with MAPE up to 71%, although relative quantifications (ICC/*r*) indicated good agreement. Additionally, the prediction method significantly moderated validity estimates, with polynomial regression models showing comparatively lower validity despite moderate-to-high correlations (one-point: *r* = 0.85–0.98 [111]; multipoint: *r* = 0.78–0.98 [112, 113]). LV regression models yielded a wide range of correlations (*r* = 0.40–0.99) [14, 109, 113], with both two-point and single-point models showing similarly broad validity estimates [112, 116, 124]. Although current practical recommendations suggest that two carefully selected loads may be sufficient [6], heterogeneous loading schemes and limited reporting preclude conclusions regarding the superiority of two-point over multipoint approaches. The apparently narrower validity range of single-point methods should likewise be interpreted cautiously because few studies used this approach. Increasing load points improved prediction accuracy in one study (three points: *r* = 0.78; five points: *r* = 0.93), [7] but this likely reflects broader sampling of the load–velocity spectrum rather than the number of loads alone. Accordingly, load location and distribution may be more important than the absolute number of tested loads [109, 142]. Recent evidence suggests that the location and distribution of tested loads are more important than the absolute number of loads.

Quadratic regression models (*r* = 0.98–0.99) [8] showed high validity but were evaluated in fewer studies. Prediction accuracy also appears to benefit from individualization: individualized regression equations (*r* = 0.96–0.98) outperformed generalized equations (*r* = 0.88–0.94) [15, 20, 107], and individualized or optimized minimum velocity thresholds may reduce bias and absolute prediction error, particularly in lower-body exercises [15, 109, 143]. This may partly explain the lower validity observed for squat and deadlift predictions. However, high group-level validity can obscure substantial individual error. For example, Ruf et al. [16] confirmed consistent group-level overestimations (~ 5–10 kg) in conventional deadlift, with high inter-individual variability (− 14–29 kg).

#### Reliability of 1RM Predictions

Reliability of 1 RM prediction was evaluated in 13 studies showing good-to-excellent ICC = 0.90 [0.83–0.94]. Despite a wide range of ICC = 0.14–0.99, the exercise type emerged as the only significant moderator (*p* < 0.001), with squat-based predictions demonstrating lower reliability (*β* = − 1.28, *p* < 0.001) when the LV method was applied. Within the squat exercise, however, prediction stability strongly depended on the number of measured loads (Multipoint: ICC = 0.61–0.96 [7, 19, 21], Two-point method: ICC = 0.24–0.99 [132]. Importantly, even when reliability was reduced for squat-based predictions, Bazuelo-Ruiz et al. [122] and Perez-Castilla et al. [119, 135] reported absolute mean differences between predicted and measured 1RM values of< 5 kg when two-point and multiple-point methods were applied in the bench press. However, recent evidence indicates that back squat prediction accuracy can be substantially improved when optimized MVTs are used [109, 143], suggesting that lower reliability in squat-based predictions may partly reflect suboptimal threshold selection rather than exercise type alone. Nevertheless, biomechanical factors may also contribute. Lower-body movements involve greater displacement and more complex bar paths compared to upper-body exercises, which typically follow a more vertical and linear trajectory and may therefore reduce the predictability and stability of the LV relationship in complex lower-body tasks, resulting in a less predictable relationship [144].

## Limitations

Despite the comprehensive scope of this meta-analysis, several limitations must be considered when interpreting these findings. First, substantial between-study heterogeneity was observed across most analyses, reflecting large methodological differences in study design, participant characteristics, exercise selection, sensor technology, and statistical reporting. While moderator analyses were conducted to explore these sources, several moderator levels were represented by only a small number of studies (e.g., Pearson-based sensor type regression). Consequently, some statistically significant moderator effects may reflect study-specific characteristics rather than robust, generalizable patterns. Second, the majority of included studies used small sample sizes (*n* < 30) [42] and male-dominated cohorts, which may reduce the generalizability of findings across populations, training levels, and sex. In addition, incomplete reporting of Bland–Altman analyses and error margins in several studies further constrained the evaluation of systematic and random measurement error. A further limitation is that different ICC formulations may have been pooled, as several studies did not report whether ICCs were based on consistency or absolute agreement models. Since these ICC types are not directly interchangeable, this may have contributed to the observed heterogeneity. These limitations, although originating from the primary studies, may have biased the analysis and affected the interpretation of the meta-analytical results, calling for further evaluation.

## Conclusion

Overall, commercially available velocity sensors demonstrated good-to-excellent pooled relative validity and reliability, although these findings were mainly supported by LPT-based systems. CCC-based evidence for agreement was limited but generally favorable where available. However, substantial heterogeneity across studies highlights that measurement quality is strongly influenced by contextual factors such as exercise complexity, sensor technology, and movement intensity. LPT-based systems appeared more consistent than IMU-based systems, while prediction accuracy was generally more robust for upper-body exercises and less consistent for complex lower-body movements such as the squat and deadlift, although device-specific performance should be considered. Also, velocity-based 1RM prediction models showed high average validity and reliability, supporting their potential use as a practical alternative to maximal strength testing, but their practical use depends on acceptable absolute prediction errors. Although recent evidence suggests that optimized MVT selection can substantially improve prediction accuracy in these exercises, prediction accuracy varied considerably between exercises, with complex lower-body movements such as the squat and deadlift demonstrating reduced stability and validity. These findings suggest that velocity-based prediction should be interpreted cautiously and practitioners should preferentially rely on sensor- and exercise-specific evidence rather than pooled estimates alone when implementing velocity-based monitoring or 1RM prediction. However, some subgroup-specific recommendations remain preliminary because they are based on a limited number of studies. Consequently, conclusions regarding the practical precision of velocity-based monitoring and prediction remain limited.

## Supplementary Information

Supplementary material 1.

## Data Availability

The datasets and code used and/or analyzed during the current study are available from the corresponding author on reasonable request.
