# Supplementary material for: Reliability, Device Agreement and Validity of Load–Velocity Profiles: A Systematic Review with Meta-analysis
Source: Sports Med Open. 2026 Sep 3;12:131. doi: 10.1186/s40798-026-01102-0 (PMC13542044; doi:10.1186/s40798-026-01102-0)
Supplement: Supplementary file 1 [file 40798_2026_1102_MOESM1_ESM.docx]

**Supplementary Materials**

**S1: Search Terms**

Part I: velocity devices
Pubmed: ((“validity” “agreement” OR “reliability” OR "Intraclass correlation coefficient" OR "ICC" OR "test-retest reliability" OR "measurement reliability" OR "consistency" OR "repeatability") AND ("velocity-based" OR "force-velocity" OR "load-velocity" OR "speed measurement" OR "velocity measurement" OR "velocity sensor" OR "load-velocity profiling" “tracking" OR “motion capture” OR “inertial sensor” OR “displacement transducer” OR “linear position transducer”))

Web of Science: TS=("validity” OR “agreement” OR “reliability” OR "Intraclass correlation coefficient" OR ICC OR "test-retest reliability" OR "measurement reliability" OR “consistency” OR “repeatability”) AND TS=("velocity-based" OR "force-velocity" OR "load-velocity" OR "speed measurement" OR "velocity measurement" OR "velocity sensor" OR "load-velocity profiling" OR "motion tracking" OR "motion capture" OR "inertial sensor" OR "displacement transducer" OR "linear position transducer")

Scopus: TITLE-ABS-KEY ((validity OR agreement OR reliability OR "Intraclass correlation coefficient" OR ICC OR "test-retest reliability" OR "measurement reliability" OR consistency OR repeatability) AND ("velocity-based" OR "force-velocity" OR "load-velocity" OR "speed measurement" OR "velocity measurement" OR "velocity sensor" OR "load-velocity profiling" OR "motion tracking" OR "motion capture" OR "inertial sensor" OR "displacement transducer" OR "linear position transducer"))

Part II: velocity-based 1RM prediction

Pubmed: ("load velocity" OR "velocity profile*" OR "velocity-based profile*" OR "velocity profiling" OR "load-velocity profiling" OR "velocity-load relationship" AND ("1RM" OR "one repetition maximum" OR "one rep max" OR "one-rep max" OR "maximal strength" OR "strength prediction" OR "strength estimation") AND ("validity" OR "reliability" OR "agreement" OR "measurement" OR "accuracy" OR "consistency" OR "Intraclass correlation coefficient" OR "ICC" OR "test-retest") à 91

Web of Science: TS=(("load velocity" OR "velocity profil*" OR "velocity-based training" OR "load-velocity relationship" OR "force-velocity" OR "velocity-based profile*") AND ("1RM" OR "one repetition maximum" OR "one rep max" OR "one-rep max" OR "strength prediction" OR "strength estimation" OR "maximal strength") AND (validity OR reliability OR agreement OR consistency OR repeatability OR accuracy OR "test-retest" OR "Intraclass correlation coefficient" OR ICC)) à 239

Scopus: TITLE-ABS-KEY ( ( "load velocity" OR "velocity profil" OR "velocity-based training" OR "load-velocity relationship" OR "force-velocity" OR "velocity-based profile*" ) AND ( "1RM" OR "one repetition maximum" OR "one rep max" OR "one-rep max" OR "strength prediction" OR "strength estimation" OR "maximal strength" ) AND ( "validity" OR "reliability" OR "agreement" OR "consistency" OR "repeatability" OR "accuracy" OR "test-retest" OR "Intraclass correlation coefficient" OR "ICC" ) ) à 172

**S2-S12: Funnel plots**

Part I:

S2-S4: Device Agreement

S5-S6: Device Validity

S7-S9: Interday Reliability

S10-S11: Intraday Reliability

Part II:

S12-S13: Validity

S14: Interday Reliability

**S15: Study characteristics part I**

| **Study** | **Procedure** | **Muscle group/**  **Exercise** | **Results** | **clinical recommendation** |
| --- | --- | --- | --- | --- |
| Gant et al. (2023) | The study evaluated the validity and reliability of the Humac360 LPT compared to the Tendo Weightlifting Analyzer in measuring velocity, displacement, and duration during barbell back squats. Seventeen recreationally active participants completed three visits: Visit 1 – Participants underwent a 1RM-test to determine maximal strength. Visits 2 & 3 – Participants performed two sets of three repetitions at 30%, 50%, 60%, and 70% of 1RM, while movement data was recorded by both devices.The study analyzed mean concentric velocity (MCV), peak velocity (PV), displacement (D), and duration (T) using ICCs to assess reliability across repetitions, sets, and sessions. Statistical methods, including paired t-tests and SEM, were applied to compare measurement accuracy between devices. | barbell back squat | *Validity*  30% 1RM: ICC=0.673; p<0.001; MV HUMAC=(0.76±0.23)m/s MV TENDO=(0.92±0.19)m/s  50% 1RM: ICC=0.553; p<0.001; MV HUMAC=(0.68±0.17)m/s  MV TENDO=(0.80±0.12)m/s  60% 1RM: ICC=0.537; p<0.001; MV HUMAC=(0.63±0.15)m/s  MV TENDO=(0.74±0.11)m/s  70% 1RM: ICC=0.610; p<0.001; MV HUMAC=(0.55±0.14)m/s  MV TENDO=(0.64±0.10)m/s  30% 1RM: ICC=0.862; p<0.001;PV HUMAC=(1.33±0.32)m/s  PV TENDO=(1.46±0.31)m/s  50% 1RM: ICC=0.860; p<0.001; PV HUMAC=(1.19±0.23)m/s  PV TENDO=(1.29±0.23)m/s  60% 1RM: ICC=0.810; p<0.001; PV HUMAC=(1.13±0.24)m/s  PV TENDO=(1.24±0.21)m/s  70% 1RM: ICC=0.784; p<0.001; PV HUMAC=(1.01±0.25)m/s  PV TENDO=(1.13±0.19)m/s  *Inter-set reliability for HUMAC*  30% 1RM: ICC=0.995; p=0.686; MV: Set1=(0.75±0.24)m/s; Set2=(0.76±0.24)m/s  50% 1RM: ICC=0.982; p=0.851; MV: Set1=(0.68±0.17)m/s; Set2=(0.68±0.19)m/s  60% 1RM: ICC=0.988; p=0.638; MV: Set1=(0.63±0.15)m/s; Set2=(0.63±0.15)m/s  70% 1RM: ICC=0.548; p=0.363; MV: Set1=(0.55±0.14)m/s; Set2=(0.62±0.33)m/s  30% 1RM: ICC=0.990; p=0.851; PV: Set1=(1.32±0.33)m/s; Set2=(1.34±0.32)m/s  50% 1RM: ICC=0.964; p=0.448; PV: Set1=(1.19±0.24)m/s; Set2=(1.17±0.30)m/s  60% 1RM: ICC=0.994; p=0.239; PV: Set1=(1.12±0.24)m/s; Set2=(1.10±0.24)m/s  70% 1RM: ICC=0.816; p=0.380; PV: Set1=(1.01±0.24)m/s; Set 2=(1.06±0.37)m/s  *Inter-session reliability for HUMAC*  30% 1RM: ICC=0.827; p=0.349; MV Day 1=(0.72±0.24)m/s Day 2=(0.80±0.20)m/s  50% 1RM: ICC=0.833; p=0.386; MV Day 1=(0.70±0.18)m/s Day 2=(0.72±0.11)m/s  60% 1RM: ICC=0.742; p=0.603; MV Day 1=(0.64±0.15)m/s Day 2=(0.66±0.08)m/s  70% 1RM: ICC=0.448; p=0.396; MV Day 1=(0.63±0.32)m/s Day 2=(0.57±0.07)m/s  30% 1RM: ICC=0.945; p=0.749; PV Day 1=(1.37±0.32)m/s Day 2=(1.38±0.38)m/s  50% 1RM: ICC=0.924; p=0.043; PV Day 1=(1.21±0.25)m/s Day 2=(1.27±0.22)m/s  60% 1RM: ICC=0.908; p=0.191; PV Day 1=(1.12±0.24)m/s Day 2=(1.16±0.20)m/s  70% 1RM: ICC=0.759; p=0.816; PV Day 1=(1.08±0.36)m/s Day 2=(1.07±0.19)m/s | Criterion validity of the HUMAC is questioned in comparison to the TENDO. Construct validity, however, is suggested due to reliable measurements of MCV, PV, D, and T between sets and sessions. |
| Caruso et al. (2012) | The study examined the reproducibility of front squat performance data collected using a triple-axis accelerometer in American college football players (n = 18) across two training sessions spaced one week apart. Participants performed four sets of front squats at 55%, 65%, 75%, and 80-83% of their 1RM, with 3-6 repetitions per set. A triple-axis accelerometer was attached to the barbell to measure peak force, velocity, and power for each lift. Test-retest reliability was assessed using ICCs, CVs, and SEM to evaluate data consistency between sessions. | front squat | *Data reproducibility Peak Velocity*  55% 1RM: ICC=0.14; PV=(260±115) cm/s  65% 1RM: ICC=0.89; PV=(201±121) cm/s  75% 1RM: ICC=0.86; PV=(191±136) cm/s  80 - 83% 1RM: ICC=0.96; PV=(147±119) cm/s | Attention is admired when monitoring the resistive exercise performance or progress of athletes with accelerometry over time. Values collected at lighter barbell loads inherently yield less data reproducibility and should thus pay attention to exercise repetition movement rates. |
| Lorenzetti et al. (2017) | The study evaluated the validity and reliability of four simple measurement devices for tracking barbell velocity during squats, comparing them against 3D motion capture (Vicon) as the gold standard. Nine participants performed two sets of five repetitions of traditional squats (70% 1RM) and ballistic squats (25 kg) in a randomized order. Simultaneously, velocity data were collected using three linear position transducers (T-FORCE, Tendo Power, GymAware) and one accelerometer-based device (Myotest). Validity and reliability were assessed by calculating RMSE and Pearson correlation coefficients between each device and the gold standard. | traditional squat & ballistic squats | *Traditional Squat 70% 1RM Mean Velocity*  MV=(0.75±0.13) m/s; T-force: r=0.970; p<0.01; Tendo: r=0.963; p < 0.01; GymAware: r=0.958; p<0.01  *Traditional Squat 70% 1RM Peak Velocity*  T-force: r=0.933; p < 0.01: Tendo: r=0.932; p<0.01; GymAware: r=0.957; p<0.01  *Ballistic Squat 70% 1RM Mean Velocity*  MV=(1.56±0.20) m/s; T-force: r=0.724; p<0.01; Tendo: r=0.770; p<0.01; GymAware: r=0.783; p<0.01; Myotest: r=0.610; p<0.01  *Ballistic Squat 70% 1RM Peak Velocity*  T-force: r=0.810; p<0.01; Tendo: r=0.860; p<0.01; GymAware: r=0.852; p<0.01; Myotest: r=0.552; p<0.01 | The study concluded that LPTs such as T-Force, Tendo Power, and GymAware provide reliable measurements for Vmean, Vmax, and tVmax during traditional squats. These devices can be effectively used for monitoring training performance, estimating 1RM, and assessing fatigue levels. For ballistic squats, LPTs also showed moderate to high reliability, but the accelerometer-based Myotest device was less accurate. Coaches and practitioners should prioritize LPTs for velocity-based training and use Vmean and tVmax as primary indicators for guiding strength training and optimizing load adjustments. |
| Janicijevic et al. (2021) | The study compared the reliability and agreement of two commonly used velocity monitoring devices (GymAware vs. T-Force) in measuring mean velocity (MV) and maximum velocity (Vmax) during the free-weight barbell back squat. Twenty resistance-trained males completed two testing sessions, with the first session used to determine 1RM and the second session consisting of two blocks of 16 repetitions at 45%, 65%, and 85% 1RM. The GymAware and T-Force devices were attached to opposite sides of the barbell and measured MV and Vmax simultaneously. Reliability was assessed using CV, ICC, and Bland-Altman plots to evaluate systematic bias and measurement agreement. | parallel free weight barbell back squat | MV 45% 1RM T-Force Left: ICC=0.77; Block1=(0.91±0.06) m/s Block 2=(0.90±0.06) m/s  MV 45% 1RM T-Force Right: ICC=0.84; Block 1=(0.91±0.08) m/s Block 2=(0.90±0.07) m/s  MV 45% 1RM GymAware Left: ICC=0.78; Block 1=(0.96±0.08) m/s Block 2=(0.95±0.07) m/s  MV 45% 1RM GymAware Right: ICC=0.69; Block 1=(0.93±0.06) m/s Block 2=(0.93±0.05) m/s  MV 65% 1RM T-Force Left: ICC=0.82; Block 1=(0.71±0.06) m/s Block 2=(0.67±0.07) m/s  MV 65% 1RM T-Force Right: ICC=0.78; Block 1=(0.71±0.07) m/s Block 2=(0.69±0.07) m/s  MV 65% 1RM GymAware Left: ICC=0.77; Block 1=(0.74±0.08) m/s Block 2=(0.73±0.07) m/s  MV 65% 1RM GymAware Right: ICC=0.82; Block 1=(0.73±0.07) m/s Block 2=(0.71±0.10) m/s  MV 85% 1RM T-Force Left: ICC=0.73; Block 1=(0.51±0.08) m/s Block 2=(0.49±0.10) m/s  MV 85% 1RM T-Force Right: ICC=0.74; Block 1=(0.50±0.09) m/s Block 2=(0.50±0.09) m/s  MV 85% 1RM GymAware Left: ICC=0.76; Block 1=(0.53±0.09) m/s Block 2=(0.53±0.10) m/s  MV 85% 1RM GymAware Right: ICC=0.76; Block 1=(0.54±0.09) m/s Block 2=(0.51±0.11) m/s  PV 45% 1RM T-Force Left: ICC=0.81; Block 1=(1.52±0.12) m/s Block 2=(1.51±0.10) m/s  PV 45% 1RM T-Force Right: ICC=0.83; Block 1=(1.52±0.11) m/s Block 2=(1.52±0.11) m/s  PV 45% 1RM GymAware Left: ICC=0.75; Block 1=(1.52±0.12) m/s Block 2=(1.54±0.12) m/s  PV 45% 1RM GymAware Right: ICC=0.58; Block 1=(1.51±0.13) m/s Block 2=(1.50±0.09) m/s  PV 65% 1RM T-Force Left: ICC=0.84; Block 1=(1.26±0.11) m/s Block 2=(1.23±0.14) m/s  PV 65% 1RM T-Force Right: ICC=0.79; Block 1=(1.29±0.11) m/s Block 2=(1.28±0.10) m/s  PV 65% 1RM GymAware Left: ICC=0.80; Block 1=(1.31±0.11) m/s Block 2=(1.29±0.11) m/s  PV 65% 1RM GymAware Right: ICC=0.81; Block 1=(1.25±0.11) m/s Block 2=(1.24±0.15) m/s  PV 85% 1RM T-Force Left: ICC=0.70; Block 1=(1.03±0.13) m/s Block 2=(1.02±0.15) m/s  PV 85% 1RM T-Force Right: ICC=0.73; Block 1=(1.04±0.11) m/s Block 2=(1.05±0.12) m/s  PV 85% 1RM GymAware Left: ICC=0.54; Block 1=(1.04±0.14) m/s Block 2=(1.06±0.12) m/s  PV 85% 1RM GymAware Right: ICC=0.79; Block 1=(1.04±0.13) m/s Block 2=(1.03±0.15) m/s | These results suggest that both the GymAware and T-Force can be used as gold-standards in studies designed to validate other velocity monitoring devices. The MV should not be used interchangeably because the T-Force rather constantly provided a MV ≈0.031 m s^−1^ higher than the GymAware. Practitioners can expect the same V_max_ values regardless of whether they use GymAware or T-Force. |
| Oleksy et al. (2023) | The study assessed the intra-rater and test-retest reliability of the GymAware linear transducer in measuring barbell force, velocity, and power during the Landmine Punch Throw (LPT) test. Twenty-five physically active male students performed the LPT test across two visits, separated by a 2-day interval. Each session included four sets of LPT with progressively increasing loads (20, 25, 30, and 35 kg) and 5-minute rest intervals between sets. Reliability was evaluated using ICC, SEM and Pearson´s r for force, power, velocity, and barbell height across repeated trials. | Landmine Punch | *Intra-rater reliability, Not throw (NT), Throw (T)*  NT MV 20kg Right: ICC=0.73; r=0.62; p<0.05; Day 1=(1.44±0.2) m/s; Day 2=(1.46±0.24) m/s  NT MV 20kg Left: ICC=0.90; r=0.82; p<0.05; Day 1=(1.28±0.26) m/s; Day 2=(1.31±0.30) m/s  NT MV 25kg Right: ICC=0.88; r=0.79; p<0.05; Day 1=(1.27±0.27) m/s; Day 2=(1.31±0.27) m/s  NT MV 25kg Left: ICC=0.87; r=0.77; p<0.05; Day 1=(1.17±0.20) m/s; Day 2=(1.21±0.19) m/s  NT MV 30kg Right: ICC=0.89; r=0.75; p<0.05; Day 1=(1.14±0.22) m/s Day 2=(1.14±0.23) m/s  NT MV 30kg Left: ICC=0.88; r=0.79; p<0.05; Day 1=(1.20±0.22) m/s Day 2=(1.12±0.21) m/s  NT MV 35kg Right: ICC=0.74; r=0.58; p<0.05; Day 1=(1.10±0.26) m/s Day 2=(1.06±0.27) m/s  NT MV 35kg Left: ICC=0.87; r=0.79; p<0.05; Day 1=(1.04±0.20) m/s Day 2=(1.03±0.19) m/s  NT PV 20kg Right: ICC=0.70; r=0.66; p<0.05; Day 1=(2.23±0.35) m/s Day 2=(2.30±0.31) m/s  NT PV 20kg Left: ICC=0.92; r=0.86; p<0.05; Day 1=(2.01±0.36) m/s Day 2=(2.04±0.39) m/s  NT PV 25kg Right: ICC=0.90; r=0.82; p<0.05; Day 1=(1.93±0.42) m/s Day 2=(2.03±0.39) m/s  NT PV 25kg Left: ICC=0.85; r=0.75; p<0.05; Day 1=(1.88±0.21) m/s Day 2=(1.96±0.29) m/s  NT PV 30kg Right: ICC=0.81; r=0.68; p<0.05; Day 1=(1.80±0.36) m/s Day 2=(1.76±0.36) m/s  NT PV 30kg Left: ICC=0.91; r=0.84; p<0.05; Day 1=(1.61±0.23) m/s Day 2=(1.68±0.29) m/s  NT PV 35kg Right: ICC=0.80; r=0.66; p<0.05; Day 1=(1.63±0.40) m/s Day 2=(1.61±0.42) m/s  NT PV 35kg Left: ICC=0.78; r=0.73; p<0.05; Day 1=(1.33±0.30) m/s Day 2=(1.62±0.34) m/s  T MV 20kg Right: ICC=0.61; r=0.46; p<0.05; Day 1=(1.32±0.21) m/s Day 2=(1.42±0.28) m/s  T MV 20kg Left: ICC=0.93; r=0.87; p<0.05; Day 1=(1.21±0.28) m/s Day 2=(1.28±0.29) m/s  T MV 25kg Right: ICC=0.82; r=0.69; p<0.05; Day 1=(1.18±0.29) m/s Day 2=(1.26±0.24) m/s  T MV 25kg Left: ICC=0.91; r=0.85; p<0.05; Day 1=(1.22±0.19) m/s Day 2=(1.23±0.20) m/s  T MV 30kg Right: ICC=0.86; r=0.75; p<0.05; Day 1=(1.04±0.19) m/s Day 2=(1.07±0.20) m/s  T MV 30kg Left: ICC=0.91; r=0.84; p<0.05; Day 1=(0.92±0.16) m/s Day 2=(0.99±0.16) m/s  T MV 35kg Right: ICC=0.81; r=0.75; p<0.05; Day 1=(0.96±0.20) m/s Day 2=(0.98±0.23) m/s  T MV 35kg Left: ICC=0.82; r=0.76; p<0.05; Day 1=(0.82±0.11) m/s Day 2=(0.94±0.17) m/s  T PV 20kg Right: ICC=0.73; r=0.57; p<0.05; Day 1=(2.41±0.38) m/s Day 2=(2.54±0.44) m/s  T PV 20kg Left: ICC=0.92; r=0.77; p<0.05; Day 1=(2.20±0.45) m/s Day 2=(2.26±0.49) m/s  T PV 25kg Right: ICC=0.90; r=0.81; p<0.05; Day 1=(2.12±0.51) m/s Day 2=(2.26±0.41) m/s  T PV 25kg Left: ICC=0.81; r=0.78; p<0.05; Day 1=(1.88±0.36) m/s Day 2=(1.78±0.37) m/s  T PV 30kg Right: ICC=0.90; r=0.83; p<0.05; Day 1=(1.92±0.38) m/s Day 2=(1.97±0.36) m/s  T PV 30kg Left: ICC=0.81; r=0.73; p<0.05; Day 1=(1.70±0.30) m/s Day 2=(1.89±0.31) m/s  T PV 35kg Right: ICC=0.81; r=0.85; p<0.05; Day 1=(1.72±0.37) m/s Day 2=(1.75±0.41) m/s  T PV 35kg Left: ICC=0.83; r=0.74; p<0.05; Day 1=(1.62±0.23) m/s Day 2=(1.52±0.34) m/s  *Test-retest reliability*  NT MV 20kg Right: ICC=0.85; r=0.79; p<0.05; Day 1=(1.44±0.21) m/s Day 2=(1.41±0.29) m/s  NT MV 20kg Left: ICC=0.77; r=0.63; p<0.05; Day 1=(1.28±0.26) m/s Day 2=(1.19±0.30) m/s  NT MV 25kg Right: ICC=0.92; r=0.85; p<0.05; Day 1=(1.27±0.27) m/s Day 2=(1.31±0.34) m/s  NT MV 25kg Left: ICC=0.91; r=0.85; p<0.05; Day 1=(1.17±0.20) m/s Day 2=(1.14±0.24) m/s  NT MV 30kg Right: ICC=0.80; r=0.85; p<0.05; Day 1=(1.14±0.22) m/s Day 2=(1.09±0.33) m/s  NT MV 30kg Left: ICC=0.80; r=0.81; p<0.05; Day 1=(1.20±0.22) m/s Day 2=(1.17±0.18) m/s  NT MV 35kg Right: ICC=0.78; r=0.63; p<0.05; Day 1=(1.10±0.26) m/s Day 2=(1.21±0.18) m/s  NT MV 35kg Left: ICC=0.86; r=0.74; p<0.05; Day 1=(1.04±0.20) m/s Day 2=(0.96±0.19) m/s  NT PV 20kg Right: ICC=0.89; r=0.76; p<0.05; Day 1=(2.23±0.35) m/s Day 2=(2.28±0.50) m/s  NT PV 20kg Left: ICC=0.76; r=0.66; p<0.05; Day 1=(2.01±0.36) m/s Day 2=(1.81±0.35) m/s  NT PV 25kg Right: ICC=0.92; r=0.87; p<0.05; Day 1=(1.93±0.42) m/s Day 2=(1.98±0.50) m/s  NT PV 25kg Left: ICC=0.94; r=0.91; p<0.05; Day 1=(1.88±0.21) m/s Day 2=(1.78±0.19) m/s  NT PV 30kg Right: ICC=0.83; r=0.74; p<0.05; Day 1=(1.80±0.36) m/s Day 2=(1.73±0.33) m/s  NT PV 30kg Left: ICC=0.85; r=0.78; p<0.05; Day 1=(1.61±0.23) m/s Day 2=(1.48±0.19) m/s  NT PV 35kg Right: ICC=0.92; r=0.85; p<0.05; Day 1=(1.63±0.40) m/s Day 2=(1.74±0.39) m/s  NT PV 35kg Left: ICC=0.82; r=0.72; p<0.05; Day 1=(1.33±0.30) m/s Day 2=(1.43±0.24) m/s  T MV 20kg Right: ICC=0.80; r=0.72; p<0.05; Day 1=(1.32±0.21) m/s Day 2=(1.35±0.23) m/s  T MV 20kg Left: ICC=0.88; r=0.79; p<0.05; Day 1=(1.21±0.28) m/s Day 2=(1.15±0.25) m/s  T MV 25kg Right: ICC=0.92; r=0.87; p<0.05; Day 1=(1.18±0.29) m/s Day 2=(1.19±0.25) m/s  T MV 25kg Left: ICC=0.83; r=0.75; p<0.05; Day 1=(1.22±0.19) m/s Day 2=(1.12±0.21) m/s  T MV 30kg Right: ICC=0.74; r=0.60; p<0.05; Day 1=(1.04±0.19) m/s Day 2=(0.98±0.22) m/s  T MV 30kg Left: ICC=0.83; r=0.70; p<0.05; Day 1=(0.92±0.16) m/s Day 2=(0.87±0.19) m/s  T MV 35kg Right: ICC=0.75; r=0.66; p<0.05; Day 1=(0.96±0.20) m/s Day 2=(1.08±0.16) m/s  T MV 35kg Left: ICC=0.73; r=0.73; p<0.05; Day 1=(0.82±0.11) m/s Day 2=(0.86±0.23) m/s  T PV 20kg Right: ICC=0.76; r=0.65; p<0.05; Day 1=(2.41±0.38) m/s Day 2=(2.36±0.43) m/s  T PV 20kg Left: ICC=0.92; r=0.77; p<0.05; Day 1=(2.20±0.45) m/s Day 2=(2.07±0.40) m/s  T PV 25kg Right: ICC=0.95; r=0.92; p<0.05; Day 1=(2.12±0.51) m/s Day 2=(2.11±0.52) m/s  T PV 25kg Left: ICC=0.90; r=0.83; p<0.05; Day 1=(1.88±0.36) m/s Day 2=(1.84±0.32) m/s  T PV 30kg Right: ICC=0.84; r=0.89; p<0.05; Day 1=(1.92±0.38) m/s Day 2=(1.87±0.42) m/s  T PV 30kg Left: ICC=0.85; r=0.73; p<0.05; Day 1=(1.70±0.30) m/s Day 2=(1.65±0.38) m/s  T PV 35kg Right: ICC=0.90; r=0.85; p<0.05; Day 1=(1.72±0.37) m/s Day 2=(1.85±0.34) m/s  T PV 35kg Left: ICC=0.87; r=0.81; p<0.05; Day 1=(1.62±0.23) m/s Day 2=(1.52±0.22) m/s | The GymAware linear transducer is recommended for monitoring force and power parameters during the Landmine Punch Throw test, particularly at lower to moderate loads (20–25 kg), where the highest reliability was observed. Caution is advised with higher loads (30–35 kg) and barbell throws due to reduced reliability, making it ideal for experienced users and tailored strength and conditioning programs. |
| Behrmann et al. (2025) | The study evaluated the validity and reliability of the EnodePro® velocity sensor in measuring bar velocity during the bench press and barbell back squat, comparing it to a high-resolution linear displacement sensor. Fifty-three moderately trained participants (16 females, 37 males) performed bench press and squat movements with loads corresponding to 30%, 50%, and 70% of their predetermined 1RM. Velocity data were simultaneously recorded by the EnodePro® sensor and a Micro-Epsilon displacement sensor, with agreement assessed using ICC, Bland–Altman analysis, MAE, and MAPE. | Bench press  deep squat | *Bench press Mean Velocities*  30% 1RM: ICC=0.904; r=0.882; Enode=(0.94±0.19)m/s; WPS=(0.88±0.18)m/s  50% 1RM: ICC=0.957; r=0.971; Enode=(0.73±0.15)m/s; WPS=0.68±0.14m/s  70% 1RM: ICC=0.972; r=0.969; Enode=(0.50±0.14)m/s WPS=(0.48±0.12)m/s  *Bench press Peak Velocities*  30% 1RM: ICC=0.956; r=0.926; Enode=(1.63±0.35)m/s WPS=(1.59±0.32)m/s  50% 1RM: ICC=0.987; r=0.979; Enode=(1.18±0.27)m/s WPS=(1.16±0.25)m/s  70% 1RM: ICC=0.970; r=0.955; Enode=(0.78±0.23)m/s WPS=(0.77±0.19)m/s  *Squat Mean Velocities*  30% 1RM: ICC=0.580; r=0.445; Enode=(0.84±0.16)m/s; WPS=0.76±0.19m/s  50% 1RM: ICC=0.835; r=0.788; Enode=(0.73±0.14)m/s WPS=(0.68±0.11)m/s  70% 1RM: ICC=0.604; r=0.511; Enode=(0.59±0.16)m/s WPS=(0.54±0.10)m/s  *Squat Peak Velocities*  30% 1RM: ICC=0.900; r=0.941; Enode=(1.49±0.24)m/s; WPS=(1.36±0.23)m/s  50% 1RM: ICC=0.822; r=0.918; Enode=(1.31±0.20)m/s; WPS=1.16±0.18m/s  70% 1RM: ICC=0.772; r=0.895; Enode=(1.14±0.19)m/s; WPS=0.97±0.18m/s | The results raise doubt about the precision and accuracy of the EnodePro, especially for complex movements. Practitioners are advised to prohibit the use of these devices until novel techniques with optimized (in this case, maximized) validity are available, as accurate training control is hindered by precision limitations. |
| Lopez-Torres et al. (2022) | The study assessed the validity and reliability of the ADR linear position transducer (LPT) for measuring mean propulsive velocity (MPV) during the bench press and compared it with the T-Force (gold standard) and Speed4Lifts (S4L) devices. Seventeen trained participants (14 men, 3 women) performed a 1RM bench press test followed by three repetitions at 45%, 55%, 65%, 75%, and 85% 1RM at maximal concentric velocity. Barbell displacement and MPV were simultaneously measured using the ADR, T-Force, and S4L devices to assess agreement. Validity and reliability were analyzed using ICC, CV, Bland-Altman plots, and standardized typical error (STE). | Bench press | *Intra-session agreement*  T-force:  45% 1RM: MV=(0.82±0.10)m/s; ICC=0.93 55% 1RM: MV=(0.69±0.09)m/s; ICC=0.95  65% 1RM: MV=(0.53±0.05)m/s; ICC=0.90  75% 1RM: MV=(0.43±0.05)m/s; ICC=0.95  85% 1RM: MV=(0.30±0.04)m/s; ICC=0.92  ADR:  45% 1RM: MV=(0.78±0.08)m/s; ICC=0.95 55% 1RM: MV=(0.66±0.07)m/s; ICC=0.96  65% 1RM: MV=(0.51±0.03)m/s; ICC=0.75  75% 1RM: MV=(0.40±0.04)m/s; ICC=0.91  85% 1RM: MV=(0.28±0.03)m/s; ICC=0.85  Speed4Lift:  45% 1RM: MV=(0.82±0.10)m/s; ICC=0.87 55% 1RM: MV=(0.68±0.10)m/s; ICC=0.97  65% 1RM: MV=(0.53±0.04)m/s; ICC=0.86  75% 1RM: MV=(0.43±0.05)m/s; ICC=0.83  85% 1RM: MV=(0.30±0.04)m/s; ICC=0.91 | The ADR is recommended as a low-cost and valid option for monitoring mean propulsive velocity during velocity-based training, especially for submaximal loads used in routine resistance training. However, it may require further calibration for higher loads to ensure optimal precision in professional athletic settings. |
| Lake et al. (2019) | The study evaluated the validity and reliability of the PUSH Band™ 2.0 in measuring peak and mean velocity during the bench press at moderate (60% 1RM) and heavy (90% 1RM) loads, comparing it to 3D motion capture (gold standard). Fourteen resistance-trained men performed three sets of three repetitions at 60% 1RM and three sets of one repetition at 90% 1RM. Barbell velocity was simultaneously recorded by the PUSH Band™ 2.0 (accelerometer-based) and 3D motion capture, with ordinary least products regression used to assess reliability and agreement. | Bench press | *Motion Capture*  60% 1RM: MV=(0.543±0.086)m/s; ICC=0.985; PV=(0.786±0.153)m/s; ICC=0.984  90% 1RM: MV=(0.297±0.067)m/s; ICC=0.988; PV=(0.441±0.132)m/s; ICC=0.985  *PUSH Band*  60% 1RM: MV=(0.608±0.108)m/s; ICC=0.937; PV=(0.825±0.168)m/s; ICC=0.947  90% 1RM: MV=(0.329±0.086)m/s; ICC=0.973; PV=(0.471±0.135)m/s; ICC=0.957 | The results show that during bench press the PUSH Band 2.0 provides reliable peak and mean velocity data. It also provides valid peak velocity data. However, it does not provide valid mean velocity data during bench press exercise. Therefore, we recommend that researchers and strength and conditioning practitioners can use bench press peak velocity data from the PUSH BandTM 2.0 confidently but should avoid considering mean velocity data from this version of the device |
| Moreno-Villanueva et al. (2022) | The study assessed the validity and reliability of the ADR linear position transducer (LPT) in measuring mean propulsive velocity (MPV) during the bench press exercise on a Smith machine, comparing it with the T-Force (gold standard). Eleven male participants performed bench press repetitions across a wide load spectrum (5 kg to 1RM) under two conditions: with (PP) and without (N-PP) an isometric phase before the concentric contraction. MPV was simultaneously recorded using the ADR encoder and T-Force, and agreement was analyzed using ICC, SEM, and Bland-Altman plots. | Benchpress (on Smith Machine) | *Inter-device reliability ADR Pause-protocol*  0%-40% 1RM: ICC=0.940; r=0.899  40%-60% 1RM: ICC=0.968: r=0.946  60%-80% 1RM: iCC=0.998; r=0.997  80%-100% 1RM: iCC=0.999; r=0.997  *Inter-device reliability ADR Non-pause-protocol*  0%-40% 1RM: ICC=0.963; r=0.946  40%-60% 1RM: ICC=0.997: r=0.994  60%-80% 1RM: iCC=0.999; r=0.998  80%-100% 1RM: iCC=0.999; r=0.997  *Agreement between ADR & T-force Pause-protocol*  0%-40% 1RM: ADR1-T-force1: SEM=0.0204m/s; ADR1-T-force2: SEM=0.0186m/s; ADR2-T-force1: SEM=0.0137m/s; ADR2-T-force2: SEM=0.0133m/s  40%-60% 1RM: ADR1-T-force1: SEM=0.0090m/s; ADR1-T-force2: SEM=0.0084m/s; ADR2-T-force1: SEM=0.0097m/s; ADR2-T-force2: SEM=0.0105m/s  60%-80% 1RM: ADR1-T-force1: SEM=0.0031m/s; ADR1-T-force2: SEM=0.0029m/s; ADR2-T-force1: SEM=0.0042m/s; ADR2-T-force2: SEM=0.0042m/s  80%-100% 1RM: ADR1-T-force1: SEM=0.0024m/s; ADR1-T-force2: SEM=0.0029m/s; ADR2-T-force1: SEM=0.0034m/s; ADR2-T-force2: SEM=0.0040m/s  *Agreement between ADR & T-force Non-pause-protocol*  0%-40% 1RM: ADR1-T-force1: SEM=0.0288m/s; ADR1-T-force2: SEM=0.0166m/s; ADR2-T-force1: SEM=0.0262m/s; ADR2-T-force2: SEM=0.0125m/s  40%-60% 1RM: ADR1-T-force1: SEM=0.0173m/s; ADR1-T-force2: SEM=0.0095m/s; ADR2-T-force1: SEM=0.0170m/s; ADR2-T-force2: SEM=0.0089m/s  60%-80% 1RM: ADR1-T-force1: SEM= -; ADR1-T-force2: SEM=0.0063m/s; ADR2-T-force1: SEM=-m/s; ADR2-T-force2: SEM=0.0060m/s  80%-100% 1RM: ADR1-T-force1: SEM=-; ADR1-T-force2: SEM=0.0038m/s; ADR2-T-force1: SEM=-; ADR2-T-force2: SEM=0.0034m/s | ADR encoder offers high reliability for the measurement of MPV in bench press exercise performed on Smith machine regardless of their execution mode, in the entire range of intensities.However, this device is not interchangeable with T- Force since it considerably underestimates the MPV values, especially at low loads (0%–40%). The data should be interpreted with caution. |
| García-Pinillos et al. (2019) | The study investigated the validity and reliability of the WIMU® system, a wireless inertial measurement unit (IMU), in tracking barbell velocity during the half-squat exercise, comparing it to the T-Force linear position transducer (LPT) as the gold standard. Nineteen competitive male powerlifters performed an incremental half-squat loading test, progressively increasing weight until reaching their maximal effort. Barbell velocity parameters—including mean velocity (MV), mean propulsive velocity (MPV), and maximum velocity (Vmax)—were measured simultaneously using WIMU® and T-Force. The study assessed reliability using CV and SEM and validity using Pearson correlation analysis, Bland–Altman plots, and linear regression models to compare velocity outputs between devices. | half-squat | *Pearson Correlation between LPT & WIMU for MV*  10% 1RM: r=0.865; 20% 1RM: r=0.520; 30% 1RM: r=0.696; 40% 1RM: r=0.877; 50% 1RM: r=0.760; 60% 1RM: r=0.646; 70% 1RM: r=0.419; 80% 1RM: r=0.662; 90% 1RM: r=0.739; 100% 1RM: r=0.687  *Pearson Correlation between LPT & WIMU for MPV*  10% 1RM: r=0.898; 20% 1RM: r=0.398; 30% 1RM: r=0.813; 40% 1RM: r=0.882; 50% 1RM: r=0.823; 60% 1RM: r=0.645; 70% 1RM: r=0.628; 80% 1RM: r=0.632; 90% 1RM: r=0.717; 100% 1RM: r=0.685  *Pearson Correlation between LPT & WIMU for Vmax*  10% 1RM: r=0.971; 20% 1RM: r=0.773; 30% 1RM: r=0.196; 40% 1RM: r=0.842; 50% 1RM: r=0.908; 60% 1RM: r=0.729; 70% 1RM: r=0.819; 80% 1RM: r=0.498; 90% 1RM: r=0.742; 100% 1RM: r=0.861 | Results indicate that the WIMU system is a reliable tool for tracking barbell velocity in the half squat, but these data also reveal some limitations regarding its concurrent validity as compared to the gold standard, with velocity measures slightly underestimated in the tested conditions |
| Muyor et al. (2017) | The study examined the validity and reliability of the WIMU® accelerometer system for measuring mean velocity during resistance exercises, specifically the back squat in a Smith machine, by comparing it to a T-Force linear encoder (gold standard). Twenty-three trained male participants performed 10 repetitions at 40% 1RM and 8 repetitions at 80% 1RM, executing the back squat at maximum concentric velocity. Velocity data were simultaneously recorded using the WIMU® accelerometer and the T-Force linear encoder, with measurements taken during both concentric and eccentric phases. Reliability was analyzed using ICC, SEM, and CV, while validity was assessed through regression analysis, Bland-Altman plots, and systematic bias evaluation. | back squat  smith machine | *Test-retest reliability T-force for Mean Velocities*  40% 1RM concentric: T-force: S1=(0.77±0.12)m/s; S2=(0.78±0.11)m/s; ICC=0.979  40% 1RM eccentric: T-force: S1=(-0.69±0.13)m/s; S2=(-0.71±0.13)m/s; ICC=0.970  80% 1RM concentric: T-force: S1=(0.68±0.09)m/s; S2=(0.66±0.07)m/s; ICC=0.855  80% 1RM eccentric: T-force: S1=(-0.63±0.10)m/s; S2=(-0.62±0.10)m/s; ICC=0.924  *Test-retest reliability WIMU for Mean Velocities*  40% 1RM concentric: Wimu: S1=(0.74±0.12)m/s; S2=(0.74±0.12)m/s; ICC=0.976  40% 1RM eccentric: Wimu: S1=(-0.67±0.14)m/s; S2=(-0.66±0.15)m/s; ICC=0.955  80% 1RM concentric: Wimu: S1=(0.65±0.09)m/s; S2=(0.65±0.08)m/s; ICC=0.905  80% 1RM eccentric: Wimu: S1=(-0.60±0.09)m/s; S2=(-0.59±0.08)m/s; ICC=0.924  *Concurrent validity T-force and WIMU for Mean Velocities*  40% 1RM concentric: ICC=0.970  40% 1RM eccentric: ICC=0.971  80% 1RM concentric: ICC=0.976 80% 1RM eccentric: ICC=0.953 | In conclusion, the findings of this study suggest that the Wimu system is a reliable and valid tool for the assessment of mean velocity during the back squat exercise using the Smith machine. |
| Pérez-Castilla et al. (2019) | The study evaluated the validity and reliability of seven commercially available devices for measuring barbell velocity during the bench press exercise, comparing them to a 3D motion capture system (Trio-OptiTrack) as the gold standard. Fourteen resistance-trained men completed two testing sessions, with the first session determining 1RM and the second session including three repetitions at 45%, 55%, 65%, 75%, and 85% of 1RM at maximal concentric velocity. Barbell velocity was measured simultaneously using one linear velocity transducer (T-Force), two linear position transducers (Chronojump, Speed4Lift), one camera-based system (Velowin), one smartphone app (PowerLift), and two inertial measurement units (IMUs: PUSH Band and Beast Sensor). Reliability was assessed using ICC and CV, while validity was analyzed via Pearson correlation coefficients, Bland-Altman plots, and systematic bias evaluation. | Bench press | *Trio-OptiTrack:*  45% 1RM: MV=(0.84±0.05)m/s; ICC=0.73  55% 1RM: MV=(0.71±0.05)m/s; ICC=0.93  65% 1RM: MV=(0.58±0.05)m/s; ICC=0.84  75% 1RM: MV=(0.46±0.05)m/s; ICC=0.83  85% 1RM: MV=(0.35±0.05)m/s; ICC=0.88  *T-force:*  45% 1RM: MV=(0.83±0.06)m/s; ICC=0.90  55% 1RM: MV=(0.70±0.05)m/s; ICC=0.95  65% 1RM: MV=(0.59±0.05)m/s; ICC=0.78  75% 1RM: MV=(0.49±0.05)m/s; ICC=0.77  85% 1RM: MV=(0.37±0.05)m/s; ICC=0.87  *Chronojump:*  45% 1RM: MV=(0.90±0.05)m/s; ICC=0.87  55% 1RM: MV=(0.76±0.05)m/s; ICC=0.90  65% 1RM: MV=(0.60±0.07)m/s; ICC=0.72  75% 1RM: MV=(0.47±0.06)m/s; ICC=0.85  85% 1RM: MV=(0.34±0.05)m/s; ICC=0.86  *Speed4Lift:*  45% 1RM: MV=(0.88±0.06)m/s; ICC=0.87  55% 1RM: MV=(0.75±0.04)m/s; ICC=0.84  65% 1RM: MV=(0.63±0.05)m/s; ICC=0.93  75% 1RM: MV=(0.51±0.05)m/s; ICC=0.81  85% 1RM: MV=(0.38±0.05)m/s; ICC=0.94  *PowerLift:*  45% 1RM: MV=(0.79±0.04)m/s; ICC=0.84  55% 1RM: MV=(0.70±0.06)m/s; ICC=0.85  65% 1RM: MV=(0.58±0.05)m/s; ICC=0.74  75% 1RM: MV=(0.51±0.05)m/s; ICC=0.87  85% 1RM: MV=(0.40±0.04)m/s; ICC=0.85  *Velowin:*  45% 1RM: MV=(0.91±0.06)m/s; ICC=0.83  55% 1RM: MV=(0.77±0.05)m/s; ICC=0.79  65% 1RM: MV=(0.64±0.06)m/s; ICC=0.83  75% 1RM: MV=(0.51±0.05)m/s; ICC=0.68  85% 1RM: MV=(0.38±0.06)m/s; ICC=0.69  *PUSH band:*  45% 1RM: MV=(0.79±0.07)m/s; ICC=0.69  55% 1RM: MV=(0.63±0.07)m/s; ICC=0.46  65% 1RM: MV=(0.46±0.08)m/s; ICC=0.78  75% 1RM: MV=(0.31±0.06)m/s; ICC=0.50  85% 1RM: MV=(0.24±0.06)m/s; ICC=0.47  *Beast sensor:*  45% 1RM: MV=(0.82±0.32)m/s; ICC=0.29  55% 1RM: MV=(0.70±0.27)m/s; ICC=0.64  65% 1RM: MV=(0.51±0.21)m/s; ICC=0.30  75% 1RM: MV=(0.34±0.16)m/s; ICC=0.31  85% 1RM: MV=(0.23±0.15)m/s; ICC=0.27 | These results suggest that linear velocity/position transducers, camera based optoelectronic systems, and the smartphone application could be used to obtain accurate velocity measurements for restricted linear movements, whereas the IMUs used in this study were less reliable and valid. |
| Askow et al. (2018) | The study assessed the validity and reliability of a commercially available LPT for measuring velocity, power, and force during the barbell back squat, comparing it to a 3D motion capture system (Qualysis) and dual force platforms as the gold standard. Nine resistance-trained men performed four squat sessions, completing a single set to volitional failure at 75% or 90% 1RM, with kinetic and kinematic data recorded simultaneously. Barbell velocity, peak force, and power were measured using the LPT and gold standard systems, with agreement assessed through ICC, Bland-Altman plots, and SEM. The study analyzed differences in measurement accuracy between high and low velocity trials to determine whether the LPT remained valid and reliable across different force-velocity conditions. | back squat | *Overall Validity and Reliability between GymAware & Qualisis*  Peak Velocity:  Below Median: ICC=0.975  Above Median:ICC= 0.953  All Values: ICC=0.982  Mean Velocity:  Below Median: ICC= 0.914  Above Median:ICC= 0.894  All Values: ICC=0.966 | Together, these data indicate that the commercially-available LPT is a valid and reliable measure for kinetic and kinematic variables of interest with the exception of high peak velocities. |
| Balsalobre-Fernández et al. (2016) | The study investigated the validity and reliability of the PUSH wearable device for measuring movement velocity during the back squat exercise, comparing it with a T-Force linear transducer (gold standard). Ten recreationally active men performed three repetitions of the back squat on a Smith machine at five different loads ranging from 25% to 85% of 1RM. Movement velocity was simultaneously recorded using the PUSH wearable device (an accelerometer worn on the forearm) and the T-Force linear transducer (attached to the barbell). The study analyzed mean velocity and peak velocity, assessing reliability through ICC, SEE, and Bland-Altman plots to determine systematic bias. | back squat | *Agreement between T-force and PUSH band for PV*  T-force=(1.55±0.27)m/s; PUSH=(1.47±0.33)m/s; r=0.91; ICC=0.944  *Test-Retest Reliability for PV*  T-force: ICC=0.988; r=0.975  PUSH: ICC=0.981; r=0.952  *Agreement between T-force and PUSH band for MV*  T-force=(0.77±0.17)m/s; PUSH=(0.88±0.22)m/s; r=0.86; ICC=0.907  *Test-Retest Reliability for MV*  T-force: ICC=0.989; r=0.980  PUSH: ICC=0.978; r=0.956 | In conclusion, these results support the use of an affordable wearable device to track velocity during back squat training. |
| Chavda et al. (2024) | The study assessed the validity and reliability of the Enode inertial measurement unit (IMU) for measuring barbell kinematics and kinetics during the snatch exercise, comparing it to an 11-camera 3D motion capture system (gold standard). Thirteen competitive weightlifters performed two snatch repetitions at 85% of their 1RM across two separate testing sessions to evaluate within- and between-session reliability. The Enode sensor was attached to the barbell and simultaneously recorded barbell trajectory, velocity, acceleration, force, and power, with data compared against the 3D motion capture system. Passing–Bablok regression, ICC, SEM, and Bland-Altman plots were used to assess agreement, reliability, and systematic bias between the two measurement methods | snatch | *Session 1 Within-session reliability Enode*  1st pull_v1: ICC= 0.940;1st pull_x1: iCC=0.667; 1st pull_y1: ICC=0.871; Transition_vT: ICC=0.727; Transition_xT: ICC=0.837; Transition_yT: ICC=0.772; 2nd Pull_v2: ICC=0.928; 2nd Pull_x2: ICC=0.901; 2nd Pull_y2: ICC=0.705; Turnover_xPBH: ICC=0.756; Turnover_yPBH: ICC=0.766; Receive_xR: iCC=0.629; Receive_yR: ICC=0.728; Catch_D1: ICC=0.841; xCatch: ICC=0.519; yCatch: ICC=0.696; Catch_D2: ICC=0.877; Catch_D3: ICC=0.925; xLoop: ICC=0.822; yLoop: ICC=0.584; Loop: ICC=0.850  *Session 2 Within-session reliability Enode*  1st pull_v1: ICC= 0.835;1st pull_x1: iCC=0.829; 1st pull_y1: ICC=0.707; Transition_vT: ICC=0.738; Transition_xT: ICC=0.836; Transition_yT: ICC=0.833; 2nd Pull_v2: ICC=0.950; 2nd Pull_x2: ICC=0.831; 2nd Pull_y2: ICC=0.861; Turnover_xPBH: ICC=0.649; Turnover_yPBH: ICC=0.893; Receive_xR: iCC=0.630; Receive_yR: ICC=0.760; Catch_D1: ICC=0.504; xCatch: ICC=0.613; yCatch: ICC=0.798; Catch_D2: ICC=0.870; Catch_D3: ICC=0.962; xLoop: ICC=0.754; yLoop: ICC=0.835; Loop: ICC=0.702  *Between-session reliability Enode*  1st pull_v1: ICC= 0.917;1st pull_x1: iCC=0.732; 1st pull_y1: ICC=0.881; Transition_vT: ICC=0.942; Transition_xT: ICC=0.919; Transition_yT: ICC=0.966; 2nd Pull_v2: ICC=0.954; 2nd Pull_x2: ICC=0.937; 2nd Pull_y2: ICC=0.943; Turnover_xPBH: ICC=0.834; Turnover_yPBH: ICC=0.930; Receive_xR: iCC=0.818; Receive_yR: ICC=0.785; Catch_D1: ICC=0.825; xCatch: ICC=0.698; yCatch: ICC=826; Catch_D2: ICC=0.897; Catch_D3: ICC=0.966; xLoop: ICC=0.862; yLoop: ICC=0.894; Loop: ICC=0.787 | Overall the Enode offers a practical and affordable option for coaches seeking to monitor weightlifting technique in training environments. |
| Elsworthy et al. (2021) | The study investigated the validity and reliability of using load-velocity relationship (LVR) profiles to determine the 1 m·s⁻¹ load (V1Load) in the back squat, aiming to assess its effectiveness in monitoring strength and neuromuscular fatigue. Twenty-seven resistance-trained male rugby league players completed two load-velocity testing sessions separated by one week, performing 3 repetitions at 30%, 40%, and 60% 1RM, and 1 repetition at 80% 1RM, with bar velocity tracked using a GymAware linear position transducer. A subgroup of 18 players performed two additional sessions (separated by 24 hours) in which they completed three back squat repetitions at their predicted V1Load, allowing assessment of test-retest reliability and criterion validity. The study analyzed ICC, CV, and SEM to evaluate the reliability and accuracy of the predicted V1Load and its effectiveness in assessing neuromuscular status. | back squat | *Mean concentric velocity GymAware*  30% 1RM: S1=(1.21±0.09)m/s; S2=(1.19±0.09)m/s; ICC=0.72  40% 1RM: S1=(1.10±0.08)m/s; S2=(1.10±0.06)m/s; ICC=0.74  60% 1RM: S1=(0.87±0.07)m/s; S2=(0.87±0.06)m/s; ICC=0.80  80% 1RM: S1=(0.66±0.07)m/s; S2=(0.65±0.09)m/s; ICC=0.82 | The study supports the use of load-velocity profiling to monitor performance and neuromuscular readiness in athletes. Practitioners can use V1Load testing as part of warm-up routines to identify fatigue or inform training adjustments based on velocity deviations. This method is particularly useful in team sports with time constraints, offering a practical approach to individualize load prescription and monitor training adaptations. |
| Zhang et al. (2024) | The study developed and evaluated a novel IMU-based method for measuring barbell velocity, displacement, and motion counting in resistance training exercises, comparing it to the Enode Pro sensor, GymAware linear position transducer (LPT), and a 3D motion capture system (VICON) as the gold standard. Five resistance-trained male participants performed back squats and bench presses at 60% of their 1RM while barbell velocity, displacement, and movement counts were simultaneously recorded using the proposed IMU system, Enode Pro, GymAware, and VICON. The proposed IMU method employed integration with high-pass filtering to improve the accuracy of mean velocity (MV), peak velocity (PV), and displacement calculations. Validity and reliability were assessed using Pearson’s r, ICC, mean absolute deviation (MAD), SEE, and Bland-Altman plots to compare the IMU system with Enode Pro, GymAware, and VICON. | back squat,  benchpress | *Back Squat Mean Velocity*  Enode vs. GymAware: r=0.85; ICC=0.86  Enode vs. VICON: r=0.70; ICC=0.65  *Back Squat Peak Velocity*  Enode vs. GymAware: r=0.92; ICC=0.92  Enode vs. VICON: r=0.95; ICC=0.95  *Bench press Mean Velocity*  Enode vs. GymAware: r=0.82; ICC=0.82  Enode vs. VICON: r=0.81; ICC=0.80  *Bench press Peak Velocity*  Enode vs. GymAware: r=0.92; ICC=0.91  Enode vs. VICON: r=0.95; ICC=0.95 | The study demonstrated that the proposed IMU-based method for measuring barbell velocity and displacement is a cost-effective and reliable alternative to traditional motion capture systems and LPTs. The results showed high validity and reliability, especially for MV and PV in back squat and bench press exercises.Strength and conditioning professionals can confidently use IMU-based devices for VBT, as they offer a portable and user-friendly solution with strong agreement to gold-standard methods like VICON and GymAware. However, careful calibration is required to minimize measurement errors, and further research is needed to validate its use across a broader range of loads and populations |
| Jukic et al. (2023) | The study investigated the reproducibility and sensitivity of three velocity monitoring devices—GymAware, PUSH2, and VmaxPro—during resistance training. Fifty-one trained participants completed five lab sessions, including familiarization, 1RM testing, and repetitions-to-failure (RTF) tests at different intensities (70%, 80%, and 90% of 1RM). During all trials, mean velocity (MV) and peak velocity (PV) were recorded simultaneously using two devices of each brand. Statistical analyses, including reliability estimations and sensitivity assessments, were performed to determine each device's accuracy in detecting meaningful changes in resistance training performance. | back squat | *GymAware*  MV: ICC=0.995; CCC=0.995 PV: ICC=0.989; CCC=0.989  *VmaxPro (Enode)*  MV: ICC=0.997; CCC=0.997 PV: ICC=0.987; CCC=0.988  *PUSH2*  MV: ICC=0.987; CCC=0.987 PV: ICC=0.979; CCC=0.979 | Based on the findings, GymAware demonstrated the highest reproducibility and sensitivity for detecting small changes in resistance training performance, making it the most reliable tool for velocity-based training. VmaxPro is a viable, cost-effective alternative to GymAware, but only when using the MV metric. PUSH2, however, showed greater measurement errors and lower sensitivity, making it less suitable for precise training monitoring. Coaches and practitioners should prioritize GymAware or VmaxPro (MV only) for resistance training monitoring, while PUSH2 should be used cautiously due to its potential for inaccurate performance assessments. |
| Jovanovic & Jukic (2020) | The study involved twelve strength-trained men performing hexagonal barbell deadlifts (HBD) to assess the reliability of two velocity measurement devices: GymAware (GYM) and PUSH2. Participants attended three laboratory sessions, including a familiarization session, a 1RM test, and a repetitions-to-failure (RTF) assessment with 80% and 90% of their 1RM. Movement velocity (mean and peak) was recorded simultaneously with two GYM and two PUSH2 devices. Statistical analysis, including regression models and error calculations, was conducted to evaluate the devices' within-unit reliability and agreement. | hexagonal barbell deadlift | *Within-Units-Reliability*  GymAware MV: r=0.996753; PV: r=0.998592 PUSH2 MV: r=0.888172; PV: r=0.894115  *Between-Units Agreement*  Right MV: r=0.915482; Left MV: r=0.935972  Right PV: r=0.939805; Left PV: r=0.947874 | The findings of the present study suggest that sport professionals should not use PUSH2 devices when the aim is to accurately monitor velocity variables during HBD exercise because low within-unit agreement and high fixed and proportional bias and RSE compared with GYM devices may compromise the utility of the collected data. |
| Bampouras et al. (2013) | The current study assessed the validity and reliability of the Myotest Pro wireless accelerometer (ACC). Thirty physically active males performed two squat jumps, on two separate sessions. The jump was recorded simultaneously by a force platform and ACC, which was attached to a barbell resting on the subjects’ shoulders. Validity was determined using Pearson correlation coefficient (r) and t-test between the maximum force platform (FFP) and ACC (FACC) force. Between session reliability of FACC, power (PACC) and velocity (VACC) from the ACC were assessed witht-test, intraclass correlation coefficient (ICC), and coefficient of variation (CV). | squat jump with barbell resting on shoulders | *Velocity for jump with the highest force value*  S1=(2.68±0.4)m/s; S2=(2.65±0.5)m/s  ICC=0.84 | ACC is a valid and reliable tool to use for assessing barbell movement, but caution in power data interpretation is needed. |
| Perez-Castillia et al. (2021) | After an initial assessment of the 1RM for the Smith machine squat, 24 resistance-trained males participated in the study. The participants completed one or two testing sessions separated by **7 days.** In one session, the squat was performed with free weights (n = 22). In another session, the squat was performed using the Smith machine (n = 16). Each testing session consisted of two blocks with a total of eight repetitions, distributed across different load levels: 3 repetitions at 45% and 65 % of 1RM. 2 repetitions at 85% of 1RM. During the concentric phase (lifting movement), the mean velocity of the barbell was simultaneously recorded using two measurement systems (PUSH Band™ 2.0 and T-Force® System). The collected data were analyzed to assess test-retest reliability and concurrent validity (comparison with the gold standard). | squat (free), squat (smith machine) | squat free:  45 % 1RM: MCV: B1=(0.99 ± 0.10) m/s; B2=(1.03 ± 0.13) m/s; r=0.75  65 % 1RM: MCV: B1=(0.81 ± 0.13) m/s; B2=(0.80 ± 0.09) m/s; r=0.63  85 % 1RM: MCV: B1=(0.59 ± 0.08) m/s; B2=(0.58 ± 0.13) m/s; r=0.66  squat smith machine:  45 % 1RM: MCV: B1=(0.95 ± 0.07) m/s; B2=(0.93 ± 0.07) m/s r=0.58  65 % 1RM: MCV: B1=(0.77 ± 0.10) m/s; B2=(0.74 ± 0.09) m/s r=0.82  85 % 1RM: MCV: B1=(0.57 ± 0.11) m/s; B2=(0.57 ± 0.07) m/s r=0.82 | Pushband 2 shows reliable values between the measurements, but the variability is rather low in comparison with T-Force, which is why this must be clearly taken into account when evaluating the dumbbell speed |
| Maroto-Izquierdo et al. (2023) | The study investigates the validity and reliability of an Inertial Measurement System (IMS) integrated into a secondary pulley for measuring linear movement velocity (T-force) during flywheel squat exercises. Thirty-one experienced participants performed squats with three incremental loads, and their mean velocity (MV), mean propulsive velocity (MPV), and maximum velocity (Vmax) were recorded using both a validated linear encoder (gold standard) and the IMS. During testing session participants performed 3 sets of 4 rep. at three different intensities. | Flywheel Squat | *Load 0.02 kg*m^2*  *MV:*  T-force=(0.998±0.152)m/s; ICC=0.90; IMS=(0.977±0.148)m/s; ICC=0.90  *Vmax:*  T-force=(1.560±0.274)m/s; ICC=0.95; IMS=(1.596±0.265)m/s; ICC=0.95  *MPV:*  T-force=(1.072±0.168)m/s; ICC=0.87; IMS=(1.050±0.169)m/s; ICC=0.88  *Load 0.05 kg*m^2*  *MV:*  T-force=(0.905±0.140)m/s; ICC=0.94; IMS=(0.895±0.134)m/s; ICC=0.89  *Vmax:*  T-force=(1.464±0.208)m/s; ICC=0.94; IMS=(1.491±0.210)m/s; ICC=0.91  *MPV:*  T-force=(0.971±0.160)m/s; ICC=0.95; IMS=(0.962±0.155)m/s; ICC=0.92  *Load 0.11 kg*m^2*  *MV:*  T-force=(0.689±0.119)m/s; ICC=0.92; IMS=(0.713±0.119)m/S; ICC=0.93  *Vmax:*  T-force=(1.301±0.191)m/s; ICC=0.95; IMS=(1.363±0.201)m/s; ICC=0.95  *MPV:*  T-force=(0.764±0.129)m/s; ICC=0.93; IMS=(0.764±0.127)m/s; ICC=0.90 | The study recommends using the inertial measurement system (IMS) for velocity-based training (VBT) and strength assessments due to its high validity and reliability in measuring movement velocity. It suggests that the IMS can accurately differentiate between concentric and eccentric phases, making it suitable for monitoring training loads and optimizing resistance training programs. Additionally, its practical applicability allows coaches and practitioners to track performance with minimal measurement error, improving training prescription and reducing injury risk. |
| Ruiz-Alias et al. (2024) | The study examined the validity and between-day reliability of the Vitruve linear position transducer (LPT) for measuring mean velocity (MV), peak velocity (PV), mean power (MP), and peak power (PP) during free-weight and Smith machine back squats, using GymAware LPT as a reference. Fourteen male sports science students performed six experimental sessions over three weeks, with two sessions per week and 72-hour rest intervals. The first two sessions determined 1RM for free-weight and Smith machine back squats, while the remaining four sessions involved incremental load tests (20, 40, 60, 80, and 90% 1RM) where Vitruve and GymAwaresimultaneously recorded bar velocity and power outputs. Validity and reliability were assessed using Pearson correlation (r), CV, effect size (ES), and Bland-Altman plots to compare measurement agreement and systematic bias. |  | *free weight Mean Velocity GymAware(GA) & Vitruve(V)*  20% 1RM: GA=1.08m/s; V=1.03m/s; r=0.663  40% 1RM: GA=0.92m/s; V=0.86m/s; r=0.692  60% 1RM: GA=0.73m/s; V=0.67m/s; r=0.666  80% 1RM: GA=0.49m/s; V=0.47m/s; r=0.599  90% 1RM: GA=0.41m/s; V=0.39m/s; r=0.800  *free weight Peak Velocity GymAware(GA) & Vitruve(V)*  20% 1RM: GA=1.61m/s; V=1.62m/s; r=0.791  40% 1RM: GA=1.41m/s; V=1.39m/s; r=0.939  60% 1RM: GA=1.17m/s; V=1.17m/s; r=0.813  80% 1RM: GA=1.01m/s; V=0.995m/s; r=0.929  90% 1RM: GA=0.91m/s; V=0.90m/s; r=0.969  *smith machine Mean Velocity GymAware(GA) & Vitruve(V)*  20% 1RM: GA=1.12m/s; V=1.04m/s; r=0.854  40% 1RM: GA=0.93m/s; V=0.84m/s; r=0.953  60% 1RM: GA=0.71m/s; V=0.65m/s; r=0.951  80% 1RM: GA=0.50m/s; V=0.46m/s; r=0.915  90% 1RM: GA=0.38m/s; V=0.35m/s; r=0.866 *smith machine Peak Velocity GymAware(GA) & Vitruve(V)*  20% 1RM: GA=1.67m/s; V=1.65m/s; r=0.918  40% 1RM: GA=1.42m/s; V=1.41m/s; r=0.957  60% 1RM: GA=1.19m/s; V=1.18m/s; r=0.960  80% 1RM: GA=1.05m/s; V=1.04m/s; r=0.959  90% 1RM: GA=0.92m/s; V=0.91m/s; r=0.892 | The study findings suggest that Vitruve should be used with caution for velocity and power tracking in resistance training due to inconsistent validity but acceptable reliability. The authors prefer Smith Machine Over Free-Weight Squats – Vitruve exhibited greater reliability in the Smith machine squat compared to the free-weight squat, likely due to reduced horizontal barbell movement. Coaches may consider Smith machine testing for more consistent results when using Vitruve. While Interpretting MV and MP with Caution – The device systematically underestimated MV and MP, suggesting that velocity-based training prescriptions based on these values may require individual calibration to avoid misjudging training intensity. Strength coaches should adjust their interpretation of results based on the specific load range used in testing. |
| Warneke et al. (2024) | The study examined the validity and reliability of two commonly used velocity sensors (vMaxPro and Tendo) for estimating 1RM in the back squat, analyzing their measurement errors and practical applicability. Twenty-five elite youth soccer players performed 1RM back squat testing followed by two trials of submaximal squats at prescribed intensities while velocity data were collected from both sensors. Velocity-based 1RM predictions were calculated using minimal velocity thresholds and the velocity = 0 m/s method, then compared to directly measured 1RM values. Agreement between devices and predictive models was assessed using ICC, MAE, MAPE, and Bland–Altman plots to determine systematic and random biases | parallel back squat | 20% VmaxPro: T1=(1.14±0.08)m/s; T2=(1.17±0.09)m/s; ICC=0.68, MAE=0.06m/s, MAPE=5.3%  40% VmaxPro: T1=(0.98±0.07)m/s; T2=(1.01±0.08)m/s; ICC=0.78; MAE=0.04m/s; MAPE=3.8%  60% VmaxPro: T1=(0.83±0.07)m/s; T2=(0.85±0.06)m/s; ICC=0.79; MAE=0.03m/s; MAPE=4.1%  80% VmaxPro: T1=(0.67±0.05)m/s; T2=(0.68±0.04)m/s; ICC=0.80; MAE=0.22; MAPE=3.5%  90% VmaxPro: T1=(0.61±0.07)m/s; T2=(0.60±0.07)m/s; ICC=0.81; MAE=0.03; MAPE=5.3%  20% Tendo: T1=(1.23±0.13)m/s; T2=(1.28±0.11)m/s; ICC=0.66; MAE=0.07m/s, MAPE=6.5%  40% Tendo: T1=(1.06±0.10)m/s; T2=(1.08±0.09)m/s; ICC=0.74; MAE=0.04m/s; MAPE=4.2%  60% Tendo: T1=(0.90±0.08)m/s; T2=(0.89±0.06)m/s; ICC=0.70; MAE=0.04m/s; MAPE=4.4%  80% Tendo: T1=(0.70±0.06)m/s; T2=(0.70±0.06)m/s; ICC=0.77; MAE=0.03; MAPE=4.7%  90% Tendo: T1=(0.64±0.07)m/s; T2=(0.62±0.08)m/s; ICC=0.74; MAE=0.05; MAPE=7.5% | The study raises concerns about the practical relevance of using velocity sensors (vMaxPro and Tendo) for 1RM prediction due to systematic and random measurement errors. Caution in Velocity-Based 1RM Estimations – Due to high measurement variability (up to 71.6%), velocity-based predictions should not replace direct 1RM testing. The discrepancies between vMaxPro and Tendo indicate that these devices cannot be used interchangeably, as they systematically report different velocities for the same movement. |

| Grgic et al. (2020) | The study investigated the test-retest reliability of velocity and power measurements in deadlifts and squats using the GymAware PowerTool system. Sixteen resistance-trained participants attended four sessions, including a 1RM test and three testing sessions where velocity and power were recorded at different loads (30-90% 1RM). The reliability of measurements was assessed by comparing results across sessions with and without a familiarization phase. | deadlift, squat | *Deadlift*  30% 1RM:  PV1(1.67±0.23)m/s; PV2=(1.69±0.19)m/s; PV3=(1.67± 0.23)m/s; ICC=0.84; MV1=(0.98±0.11)m/s; MV2=(0.99 0.10)m/s; MV3=(0.98±0.12)m/s; ICC=0.81  45% 1RM:  PV1=(1.47 ±0.18)m/s; PV2=(1.47±0.17)m/s; PV3=(1.46±0.19)m/s; ICC= 0.84; MV1=(0.86±0.09)m/s; PV2=(0.86±0,09)m/s; PV3=(0.85±0.10)m/s; ICC=0.82  60% 1RM:  PV1=(1.26±0.15)m/s; PV2=(1.24±0.14)m/s; PV3=(1.23±0.13)m/s; ICC=0.95; MV1=(0.73±0.07)m/s; MV2=(0.73±0.09)m/s; MV3=(0.73±0.07)m/s; ICC=0.83  75% 1RM:  PV1=(0.99±0.14)m/s; PV2=(0.98±0.13)m/s; PV3=(1.00±0.11)m/s; ICC=0.91; MV1=(0.59±0.07)m/s; MV2=(0.57±0.10)m/s; MV3=(0.60±0.06)m/s; ICC=0.63  90% 1RM:  PV1=(0.76±0.18)m/s; PV2=(0.74±0.14)m/s; PV3=(0.77±0.16)m/s; ICC=0.79; MV1=(0.43±0.09)m/s; MV2=(0.42±0.08)m/s; MV3=(0.44±0.08)m/s; ICC=0.66  *Squat*  30% 1RM:  PV1=(1.63±0.19)m/s; PV2=(1.64±0.20)m/s; PV3=(1.60±0.19)m/s; ICC=0.82; MV1=(1.00±0.13)m/s; MV2=(0.99±0.13)m/s; MV3=(0.97±0.13)m/s; ICC=0.80  45% 1RM:  PV1=(1.49±0.18)m/s; PV2=(1.49±0.17)m/s; PV3=(1.48±0.15)m/s; ICC=0.92; MV1=(0.88±0.12)m/s; MV2=(0.87±0.10)m/s; MV3=(0.86±0.10)m/s; ICC=0.87  60% 1RM:  PV1=(1.35±0.13)m/s; PV2=(1.33±0.18)m/s; PV3=(1.34±0.16)m/s; ICC=0.86; MV1=(0.75±0.10)m/s; MV2=( 0.74±0.10)m/s; MV3=(0.74±0.10)m/s; ICC=0.92  75% 1RM:  PV1=(1.23±0.13)m/s; PV2=(1.19±0.16)m/s; PV3=(1.21±0.15)m/s; ICC=0.89; MV1=(0.62±0.08)m/s; MV2=(0.61±0.08)m/s; MV3=(0.61±0.10)m/s; ICC=0.89  90% 1RM:  PV1=(1.15±0.18)m/s; PV2=(1.14±0.14); PV3=(1.11±0.16)m/s; ICC=0.83; MV1=(0.51±0.08)m/s; MV2=(0.53±0.08)m/s; MV3=(0.51±0.10)m/s; ICC=  0.78 | The study found that the GymAware system provides moderate-to-excellent reliability for deadlifts and good-to-excellent reliability for squats. Familiarization did not significantly improve reliability, except for velocity measurements at lighter loads in squats. Coaches and practitioners using velocity-based training can rely on the GymAware system for consistent performance tracking, but they should ensure proper technique and consider individual variability when interpreting data. |
| --- | --- | --- | --- | --- |
| Orange et al. (2020) | This study examined the test-retest reliability of the GymAware PowerTool (GYM) for measuring velocity and power in the back squat and bench press. Twenty-nine academy rugby league players participated and completed two test-retest sessions for each exercise at 20, 40, 60, 80, and 90% of their 1RM. The GymAware device measured mean velocity (MV), peak velocity (PV), mean power (MP), and peak power (PP) for each repetition. The study assessed the reliability of the device usingICC, SEM, and smallest worthwhile change (SWC). | back squat, bench press | *Mean velocities back squat*  20% 1RM: ICC=0.72; 40% 1RM: ICC= 0.77; 60% 1RM: ICC=0.83;  80% 1RM: ICC=0.83; 90% 1RM: ICC=0.79  *Peak velocities back squat*  20% 1RM: ICC=0.77; 40% 1RM: ICC=0.78; 60% 1RM: ICC=0.79;  80% 1RM: ICC=0.68; 90% 1RM: ICC=0.65  *Mean velocities bench press*  20% 1RM: ICC=0.64; 40% 1RM: ICC=0.71: 60% 1RM: ICC=0.7  80% 1RM: ICC=0.78; 90% 1RM: ICC=0.87  *Peak velocities bench press*  20% 1RM: ICC=0.70; 40% 1RM: ICC=0.82; 60% 1RM: ICC=0.81  80% 1RM: ICC=0.77; 90% 1RM: ICC: 0.68 | The GymAware PowerTool demonstrated good reliability for velocity and power measurements at 40–90% of 1RM in both back squats and bench presses, making it a useful tool for velocity-based training (VBT). Strength and conditioning coaches can use mean velocity (MV) as a reliable metric for load monitoring, particularly at heavy loads. However, caution is advised when using peak velocity (PV) at high loads (>80% 1RM), as its reliability is lower. Additionally, GYM should not be used at very light loads (20% 1RM) due to high measurement variability. |
| Olaya-Cuartero, et al. (2022) | The study involved two laboratory test sessions conducted two weeks apart. In the first session, participants performed a standardized 1RM test for back squats, progressively increasing weights until their maximum load was determined. In the second session, mean velocity values were measured at different percentages (75%, 85%, 90%, and 95%) of the 1RM to assess the reliability and validity of the VmaxPro device compared to a 3D Motion Capture System. Data were collected using both the VmaxPro sensor and the motion capture system, and statistical analyses were performed to evaluate measurement reliability and errors. | back squat | ICC=0.986; r=0.992 | The findings suggest that the IMU-based VmaxPro device is a reliable and valid tool for monitoring execution velocity and range of displacement in strength training. Coaches and athletes can confidently use it for tracking and optimizing training loads. However, minor measurement errors indicate that it should be used cautiously in scenarios requiring high precision. Further research is recommended to assess its application across different exercises and athlete. populations. |
| Lake et al. (2018) | Twenty-two volunteers (m=18, f= 4) performed 3 CMJ on a force platform (with 60s of rest), while a belt-worn Push Band and a motion capture system (a marker affixed to the Push Band) simultaneously recorded data that enabled peak and mean velocity and power to be calculated and then compared using ordinary least products regression. | countermovement jump | PUSH Band and force plate:  r=0.826  Velocity ICC (r):  MC (motion capturing) vs. PB (PUSH Band) Mean: 0.770  MC (motion capturing) vs. PB (PUSH Band) Peak: 0.946  FP (force plate) vs. PB Mean: 0.704  FP (force plate) vs. PB Peak: 0.826 | The results of this study show that while the Push Band overestimates peak (9–17%) and mean (23–27%) velocity, it does not demonstrate fixed or proportional bias. Therefore, if these differences are considered, the Push Band can be used to reliably record CMJ peak and mean velocity. However, while the Push Band and motion capture peak powers agree, the Push Band and force plate peak powers do not. |
| Beckham et al. (2019) | The purpose of this study was to evaluate the reliability and validity of the Assess2Perform Bar Sensei during the back squat exercise, using the GymAware PowerTool as the criterion device. Eleven male and five female subjects were recruited for this study. Subjects were tested for their back squat one repetition maximum (1RM). Then, on two separate days, subjects performed two sets of three repetitions at loads of 45%, 60% and 75% 1RM. The GymAware PowerTool and Bar Sensei were attached to the barbell in similar locations for concurrent collection of mean concentric velocity (MCV) and peak concentric velocity (PCV). Agreement between devices was assessed using ICC. | back squat | Mixed-effect model to assess the degree of agreement between the two devices:  MCV (45% 1RM): Between device ICC (CI=0.482, p<0.001), Mean Difference (CI=-0.106, p<0.001), Ratio of SDs (CI=2.888, p<0.001)  MCV(60% 1RM): Between device ICC (CI=0.303, p<0.001), Mean Difference (CI=-0.094, p<0.001), Ratio of SDs (CI=3.558, p<0.001)  MCV(75% 1RM): Between device ICC (CI=0.329, p<0.001), Mean Difference (CI=-0.081, p<0.001), Ratio of SDs (CI=3.319, p<0.001)  PCV (45% 1RM): Between device ICC (CI=0.555, p<0.001), Mean Difference (CI=0.009, p=0.564), Ratio of SDs (CI=2.272, p<0.001)  PCV (60% 1RM): Between device ICC (CI=0.362, p<0.001), Mean Difference (CI=-0.037, p=0.028), Ratio of SDs (CI=3.123, p<0.001)  PCV (75% 1RM): Between device ICC (CI=0.361, p<0.001), Mean Difference (CI=-0.099, p<0.001), Ratio of SDs (CI=3.425, p<0.001)  Individual Reliability of the PowerTool and Bar Sensei:  PowerTool ICC:  MCV(45% 1RM): CI=0.774, p<0.001  MCV(60% 1RM): CI=0.752, p<0.001  MCV(75% 1RM): CI=0.651, p<0.001  PCV (45% 1RM): CI=0.793, p<0.001  PCV (60% 1RM): CI=0.775, p<0.001  PCV (75% 1RM): CI=0.761, p<0.001  Bar Sensei ICC:  MCV(45% 1RM): CI=0.419, p<0.001  MCV(60% 1RM): CI=0.171, p=0.010  MCV(75% 1RM): CI=0.295, p<0.001  PCV (45% 1RM): CI=0.451, p<0.001  PCV (60% 1RM): CI=0.273, p<0.001  PCV (75% 1RM): CI=0.349, p<0.001 | Data from the present study suggest that the version of the Bar Sensei and iOS App used for this study is neither a reliable nor valid tool to measure velocity data while performing barbell back squats. Overall reliability of the Bar Sensei across the loads tested was generally poor. Data across all trials show that the validity of the Bar Sensei, in comparison to the PowerTool, is fair to poor. Reliability and validity were contingent on 1RM intensity. In comparison with the PowerTool, the Bar Sensei appears to underestimate MCV on average in our study population. When we closely look at the data via graphic, the Bar Sensei tends to underestimate both MCV and PCV when measuring low velocities and overestimate when measuring high velocities. |
| Courel-Ibanez et al. (2019) | This study aimed to analyze the agreement between five bar velocity monitoring devices, currently used in resistance training, to determine the most reliable device based on reproducibility (between-device agreement for a given trial) and repeatability (between-trial variation for each device). Seventeen resistance-trained men (age 26.0 ± 3.6 years) performed duplicate trials against seven increasing loads (20-30-40-50-60-70-80 kg) while obtaining mean, mean propulsive and peak velocity outcomes in the bench press, full squat and prone bench pull exercises. Measurements were simultaneously registered by two linear velocity transducers (LVT: T-Force Dynamic Measurement System), two linear position transducers (LPT: Chronojump), two optoelectronic camera-based systems (OEC: Velowin), two smartphone video-based systems (VBS: PowerLift) and one accelerometer (ACC: Push Band). | bench press, squat and bench pull | Bench press inter-device agreement:  MV (LTV 1 vs. LPT 1): ICC=0.992, CCC=0.983  MV (LTV 1 vs. OEC 1): ICC=0.998, CCC=0.995  MV (LTV 1 vs. VBS 1): ICC= 0.966, CCC=0.934  MV (LTV 1 vs. ACC 1): ICC= 0.928, CCC=0.870  Bench press inter-device agreement:  MPV (LTV 1 vs. LPT 1): ICC=0.997, CCC=0.981  MPV (LTV 1 vs. OEC 1): ICC=0.997, CCC=0.995  Bench press inter-device agreement:  PV (LTV 1 vs. LPT 1): ICC=0.998, CCC=0.997  PV (LTV 1 vs. OEC 1): ICC=0.999, CCC=0.999  MV (LTV 1 vs. ACC 1): ICC= 0.937, CCC=0.881  Full squat inter-device agreement:  MV (LTV 1 vs. LPT 1): ICC=0.984, CCC=0.969  MV (LTV 1 vs. OEC 1): ICC=0.992, CCC=0.985  MV (LTV 1 vs. VBS 1): ICC= 0.955, CCC=0.913  MV (LTV 1 vs. ACC 1): ICC= 0.941, CCC=0.887  Full squat inter-device agreement:  MPV (LTV 1 vs. LPT 1): ICC=0.986, CCC=0.972  MPV (LTV 1 vs. OEC 1): ICC=0.992, CCC=0.984  Full squat inter-device agreement:  PV (LTV 1 vs. LPT 1): ICC=0.989, CCC=0.979  PV (LTV 1 vs. OEC 1): ICC=0.993, CCC=0.986  PV (LTV 1 vs. ACC 1): ICC= 0.952, CCC=0.909  Prone bench pull:  MV (LTV 1 vs. LPT 1): ICC=0.993, CCC=0.986  MV (LTV 1 vs. OEC 1): ICC=0.967, CCC=0.937  Prone bench pull:  MPV (LTV 1 vs. LPT 1): ICC=0.994, CCC=0.990  MPV (LTV 1 vs. OEC 1): ICC=0.967, CCC=0.938  Prone bench pull:  PV (LTV 1 vs. LPT 1): ICC=0.997, CCC=0.994  PV (LTV 1 vs. OEC 1): ICC=0.999, CCC=0.997  Between-trial variation (repeatability):  MV:  Bench Press vs. LTV: ICC=0.999  Bench Press vs. LPT: ICC=0.997  Bench Press vs. OEC: ICC=0.997  Bench Press vs. VBS: ICC=0.988  Bench Press vs. ACC: ICC=0.974  Full squat vs. LTV: ICC=0.995  Full squat vs. LPT: ICC=0.990  Full squat vs. OEC: ICC=0.988  Full squat vs. VBS: ICC=0.986  Full squat vs. ACC: ICC=0.979  Prone bench pull vs. LTV: ICC=0.995  Prone bench pull vs. LPT: ICC=0.990  Prone bench pull vs. OEC: ICC=0.994  MPV:  Bench Press vs. LTV: ICC=0.999  Bench Press vs. LPT: ICC=0.997  Bench Press vs. OEC: ICC=0.998  Full squat vs. LTV: ICC=0.996  Full squat vs. LPT: ICC=0.991  Full squat vs. OEC: ICC=0.987  Prone bench pull vs. LTV: ICC=0.995  Prone bench pull vs. LPT: ICC=0.987  Prone bench pull vs. OEC: ICC=0.994  PV:  Bench Press vs. LTV: ICC=0.999  Bench Press vs. LPT: ICC=0.999  Bench Press vs. OEC: ICC=0.998  Bench Press vs. ACC: ICC=0.962  Full squat vs. LTV: ICC=0.989  Full squat vs. LPT: ICC=0.985  Full squat vs. OEC: ICC=0.983  Full squat vs. ACC: ICC=0.944  Prone bench pull vs. LTV: ICC=0.999  Prone bench pull vs. LPT: ICC=0.998  Prone bench pull vs. OEC: ICC=0.998 | The LVT is the most reliable technology for measuring bar velocity in resistance training exercises, and the only one recommended as a reference for comparing emerging technologies. The OEC and LVT are valid alternatives, considering the particular margins of error for each exercise and velocity outcome. ACC and VBS are not recommended as monitoring tools for VBRT purposes given their substantial errors and uncertainty of the outcomes. For practical reasons, future studies assessing velocity monitoring technolo gies should report errors both in absolute velocity units (m s21) and their equivalent relative load units (% 1RM) |
| Ferro et al. (2019) | The aim of this study was to compare Vmax during loaded CMJ (CMJloaded) using three different technologies to show a criterion for selecting the more appropriate depending on its use. Nine recreationally active men performed a CMJloaded test. Five jumps were made in each of 6 series with a 20- kg barbell +0, + 5, +10, + 15, + 20 and + 25 kg, with 2 seconds rest between the jumps and 5 minutes rest between the series to explore a wide range of speeds. Vmax was obtained from force platform, inertial device and linear encoder technologies. Bland-Altman plots and mean differences were used to compare devices. Reproducibility was tested using the intraclass correlation coefficient (ICC) for single measures and typical error (TE). | CMJ loaded jump | Linear encoder:  OVmax (m*s-1)= 2.11 ± 0.24, JVmax 1 (m*s-1)= 2.12±0.25, JVmax 2 = 2.12±0.23, JVmax 3 (m*s-1)= 2.11±0.24, ICC= 0.944  Accelerometer:  OVmax (m*s-1)= 2.11 ± 0.26, JVmax 1 (m*s-1)= 2.12±0.26, JVmax 2 = 2.11±0.24, JVmax 3 (m*s-1)= 2.11±0.27, ICC= 0.851  Force platform:  OVmax (m*s-1)= 2.12 ± 0.24, JVmax 1 (m*s-1)= 2.12±0.24, JVmax 2 = 2.13±0.24, JVmax 3 (m*s-1)= 2.11±0.24, ICC= 0.924 | The main findings of this study confirmed our first hypothesis. No difference was found in the Vmax calculated among the three devices. In addition, the three devices were shown to be consistent when calculating Vmax with high levels of reproducibility. How ever, wide LoA were found in all comparison. |
| Abbott et al. (2020) | The aim of this study was to evaluate the level of agreement in measuring back squat kinematics between an inertial measurement unit (IMU) and a 3D motion capture system (3DMOCAP). Kinematic variables included concentric peak velocity (CPV), concentric mean velocity (CMV), eccentric peak velocity (EPV), eccentric mean velocity (EMV), mean propulsive velocity (MPV), and POP-100: a proprietary variable. Sixteen resistance-trained males (25.9 ± 5.2 years) performed an incrementally loaded one repetition maximum (1RM) squat protocol.The protocol consisted of two repetitions at each 10% load increment between 20 and 70% of self-reported 1RM and one repetition at each 5% load increment from 75% 1RM until technical or actual failure. A series of Pearson correlations, 2 x 4 RM ANOVA, Cohen’s d effect size differences, coeffcient of variation (CV), and standard error of the estimate (SEE) were calculated. A large relationship existed for all variables between devices (r = 0.78–0.95). Between-device agreement for CPV worsened beyond 60% 1RM. The remaining variables were in agreement between devices with trivial e ect size differences and similar CV magnitudes. | squat | IMU vs. 3D Motion analysis: → Pearson  EMV: r=0.92  EPV: r=0.95  CMV: r=0.90  MPV: r=0.93  CPV: r=0.78 | With the current model of this IMU, practitioners need to be aware that there is an obvious error from the IMU considering the ability to capture measurements and the results of the measurements. The results of the current investigation suggest that measurements of CON peak velocity agree between IMU and 3DMOCAP below 60% 1RM. The current study suggests that the ECCportion of the task is reasonably valid as compared to the 3D motion capture system; however, the CON portion must be calculated more precisely. |
| McGrath et al. (2018) | The purpose of this investigation was to determine the concurrent validity of two devices used in velocity-based monitoring of resistance training. The TendoTM Fitrodyne is a linear position transducer (LPT) which attaches to the barbell to measure velocity of movement. PUSHTM is a wearable inertial sensor (WIS) that is worn on the athlete’s forearm and utilizes an integrated gyroscope and accelerometer to provide a value for movement velocity. Both devices were compared with 3D motion capture data. The validation of such devices is paramount as they are being more frequently used in a strength and conditioning and biomechanics setting. Ten subjects (23.4 ± 6.8 years) completed 2 sets of 6 repetitions on the barbell bench press at loads of 40% and 80% of 1RM. Velocity differences were analysed using a one-way ANOVA with post-hoc LSD. Correlations between LPT and 3D motion capture, and WIS and motion capture were analyzed using linear regression. Bland-Altmann limits of agreement analysis was employed to scrutinise results for both portable devices to detect systematic bias and random error. | bench press | Tendo vs. 3D Motion capture:  ICC=0.977  CI=0.966-0.984  PUSH vs. 3D Motion capture:  ICC=0.923  CI=0.889-0.946 | The results of the current investigation suggest that the LPT provides valid measures for barbell velocity in the bench press exercises. Practitioners should be cognisant of systematic bias and random differences by the WIS through 20% underestimation of mean concentric velocity. |
| Arede et al. (2019) | The aim of this study was to examine the validity and reliability of the novel inertial measurement unit (named: Gyko Sport) for the measurement of barbell concentric velocity on the bench-press exercise. Ten under-16 (u16) basketball players (15.1 ± 1.0 years) performed 2 repetitions of the bench press exercise with 6 different loads (40, 50, 60, 70, 80 and 90% of one repetition maximum [1-rM]; N.=120 repetitions). Mean velocity for each repetition was simultaneously recorded using the Gyko Sport and Smartcoach linear transducer. Gyko’s accuracy was tested using paired samples t-test and Bland-altman plots. The Pearson’s product-moment correlation coefficient and bootstrapping (N.=1000) were used to validate the inertial sensor system. | bench press | 40%: r=0.637, CI= 0.193-0.882, ICC= 0.605, p=0.240  50%: r=0.682, CI= 0.533-0.846, ICC= 0.594, p=0.364  60%: r=0.495, CI= -0.023-0.842, ICC= 0.495, p=0.714  70%: r=0.410, CI= 0.162-0.715, ICC= 0.371, p=0.120  80%: r=0.207, CI= -0.291-0.632, ICC= 0.193, p=0.032  90%: r=0.025, CI= -0.107-0.596, ICC= 0.241, p=0.593 | The results of this study confirmed that Gyko Sport sys tem is acceptable for measuring barbell mean velocity on bench press exercise. However, practitioners using this device should be aware about the overestimation and error associated with respect to a criterion measure. practitioners must decide whether the magnitude of measurement error is acceptable based on the specific needs of their athletes. The advantage of the Gyko Sport is the portability and affordability (mag netic pocket). Thus, the use of Gyko Sport device may be used to develop individual performance indicators aiming to optimize training adaptations and performance |
| Sato et al. (2009) | The purpose of this study was to validate measures obtained by a commercially available accelerometer in comparison with kinematic data derived from video. The hypothesis was that there would be a high positive relationship between accelerometer data and acceleration measures derived from video records of a barbell high-pull movement. Accelerometer values and kinematic data from high-speed video were obtained from 7 male volunteers (24.29 ± 2.98 years) performing 2 trials each of a barbell high-pull. The results showed that the accelerometer measures were highly correlated with derived acceleration data from video (r = 0.94–0.99). | barbell high-pull | Pearson product-moment correlation coefficients between raw acceleration data and acceleration data obtained from kinematic analysis:  High-Pull trial 1:  r= 0.98, r = 0.99, r = 0.99, r = 0.98, r = 0.97, r = 0.95, r = 0.97  High-Pull trial 2:  r = 0.94, r = 0.99, r = 0.99 r = 0.98 r = 0.99 r = 0.99 r = 0.96 | Because of its capability for capturing data and real-time display, this commercial accelerometer could be a valuable tool for field application. Because the unit collects data that reflect force production for lifting during a training session, is easily attached, leaves the athlete unrestrained by cables, and is not cost-prohibitive, it seems worthwhile to study its potential for improving lifting performance. |
| Feuerbacher et al. (2023) | We aimed at assessing the validity and test-retest reliability of the inertial measurement unit-based Vmaxpro sensor compared with a Vicon 3D motion capture system and the T-Force sensor during an incremental 1-repetition maximum (1RM) test and at submaximal loads. Nineteen strength-trained subjects (24.6 ± 2.6 years) reported to the laboratory for the 1RM test sessions, whereas 15 subjects carried out another 3 sessions consisting of 3 repetitions with 4 different intensities (30, 50, 70, and 90% of1RM) to determine the intra-and interday reliability. Agreement between the differences of the index and the criterion (index 2 criterion) and the criterion was assessed by Bland-Altman analysis. | deep squat | Intraday reliability:  Vicon (MV with SD (m*s-1):  30 %: Repetition 1= 0.94 ± 0.12 , Repetition 2= 0.97 ± 0.11, Repetition 3= 0.98 ± 0.11  50 %: Repetition 1= 0.83 ± 0.08 , Repetition 2= 0.83 ± 0.09, Repetition 3= 0.82 ± 0.07  70 %: Repetition 1= 0.67 ± 0.09 , Repetition 2= 0.67 ± 0.06, Repetition 3= 0.47 ± 0.08  90 %: Repetition 1= 0.48 ± 0.09 , Repetition 2= 0.48 ± 0.11, Repetition 3= 0.98 ± 0.11  Vicon:  30 %: ICC= 0.713-0.868, p<0.001  50 %: ICC= 0.865-0.919, p<0.002  70 %: ICC= 0.614-0.842, p<0.016  90 %: ICC= 0.880-0.953, p<0.001  Vmaxpro (MV with SD (m*s-1):  30 %: Repetition 1= 1.0 ± 0.12 , Repetition 2= 1.05 ± 0.13, Repetition 3= 1.05 ± 0.14  50 %: Repetition 1= 0.89 ± 0.09, Repetition 2= 0.89 ± 0.08, Repetition 3= 0.88 ± 0.08  70 %: Repetition 1= 0.70 ± 0.09 , Repetition 2= 0.71 ± 0.07, Repetition 3= 0.72 ± 0.10  90 %: Repetition 1= 0.51 ± 0.09 , Repetition 2= 0.53 ± 0.11, Repetition 3= 0.51 ± 0.10  Vmaxpro:  30 %: ICC= 0.729-0.901, p<0.001  50 %: ICC= 0.708-0.911, p<0.001  70 %: ICC= 0.662-0.859, p<0.001  90 %: ICC= 0.704-0.938, p<0.001  T-Force (MV with SD (m*s-1):  30 %: Repetition 1= 0.97 ± 0.09 , Repetition 2= 0.98 ± 0.09, Repetition 3= 0.98 ± 0.09  50 %: Repetition 1= 0.82 ± 0.08, Repetition 2= 0.83 ± 0.08, Repetition 3= 0.82 ± 0.07  70 %: Repetition 1= 0.66 ± 0.06, Repetition 2= 0.6 ± 0.06, Repetition 3= 0.67 ± 0.06  90 %: Repetition 1= 0.48 ± 0.08 , Repetition 2= 0.48 ± 0.08, Repetition 3= 0.47 ± 0.07  T-Force:  30 %: ICC= 0.832-0.937, p<0.001  50 %: ICC= 0.864-0.934, p<0.001  70 %: ICC= 0.874-0.955, p<0.001  90 %: ICC= 0.868-0.956, p<0.001  Interday reliability:  Vicon (MV with SD (m*s-1):  30 %: Session 3= 0.95 ± 0.12 , Session 4= 0.98 ± 0.08, Session 5= 0.67 ± 0.05  50 %: Session 3= 0.82 ± 0.08, Session 4= 0.83 ± 0.06, Session 5= 0.82 ± 0.06  70 %: Session 3= 0.66 ± 0.08, Session 4= 0.68 ± 0.05, Session 5= 0.67 ± 0.05  90 %: Session 3= 0.46 ± 0.06 , Session 4= 0.49 ± 0.07, Session 5= 0.49 ± 0.07  Vicon:  30 %: ICC= 0.820, p<0.001  50 %: ICC= 0.804, p<0.001  70 %: ICC= 0.696, p=0.004  90 %: ICC= 0.908, p<0.001  Vmaxpro (MV with SD (m*s-1):  30 %: Session 3= 1.05 ± 0.10 , Session 4= 1.03 ± 0.07, Session 5= 1.03 ± 0.16  50 %: Session 3= 0.88 ± 0.08, Session 4= 0.89 ± 0.07, Session 5= 0.89 ± 0.06  70 %: Session 3= 0.69 ± 0.08, Session 4= 0.73 ± 0.06, Session 5= 0.71 ± 0.09  90 %: Session 3= 0.50 ± 0.09, Session 4= 0.52 ± 0.08, Session 5= 0.53 ± 0.07  Vicon:  30 %: ICC= 0.623, p=0.017  50 %: ICC= 0.623, p=0.016  70 %: ICC= 0.568, p=0.033  90 %: ICC= 0.837, p<0.001  T-Foce (MV with SD (m*s-1):  30 %: Session 3= 0.97 ± 0.11 , Session 4= 0.98 ± 0.07, Session 5= 0.99 ± 0.07  50 %: Session 3= 0.81 ± 0.09, Session 4= 0.84 ± 0.06, Session 5= 0.82 ± 0.07  70 %: Session 3= 0.65 ± 0.06, Session 4= 0.68 ± 0.06, Session 5= 0.66 ± 0.05  90 %: Session 3= 0.45 ± 0.08, Session 4= 0.48 ± 0.07, Session 5= 0.49 ± 0.08  T-Force:  30 %: ICC= 0.889, p<0.001  50 %: ICC= 0.820, p<0.001  70 %: ICC= 0.792, p<0.001  90 %: ICC= 0.901, p<0.001 | According to our data, the intraday reliability of the Vmaxpro was moderate to high (ICC: 0.662–0.938), hinting toward a somewhat weaker reliability compared with other IMUs, such as the PUSH werable device(ICC:0.981).In particular, the wide range of ICCs of the Vmaxpro sensor demonstrate inferior interday reliability, which was especially true for the velocities at the loads of 30 and 50%. The Vmaxpro sensor seems to be a valid measurement tool that can be used to monitor movement velocities within a training session, to determine the magnitude of neuromuscular fatigue when it is placed according to the manufacturer’s instructions (i.e., on the bar). However,practitioners should be cautious when assessing movement velocities on separate days because of the moderate interday reliability |
| Goldsmith et al. (2018) | The aim of this study was to examine the validity of two linear position transducers, the Tendo Weightlifting Analyzer System (TWAS) and Open Barbell System (OBS), compared to a criterion device, the Optotrak Certus Three-Dimensional Motion Capture System (OC3D). Twenty-five males (age: 25 ± 3 years) with ≥2yrs of resistance training experience completed a back 1RM and one set to failure at 70% of 1RM. Average (ACV) and peak (PCV) concentric velocity were recorded by all three devices during the final warm-up set, all 1RM International Journal of Sports Physiology and Performance attempts, and every repetition during the 70% set. Bland-Altman plots, Mountain plots, a one-way ANOVA, standard error of measurement, and intraclass correlation coefficients were used to analyze validity. | back squat | OC3D (Average Concentric Velocity =ACV):  Mean ± SD (m*s-1): 0.4706 ± 0.1450  OBS (ACV):  Mean ± SD (m*s-1): 0.4886 ± 0.1320  TWAS (ACV):  Mean ± SD (m*s-1): 0.4822 ± 0.1312  OC3D (PCV):  Mean ± SD (m*s-1): 0.8399 ± 0.1941  OBS (PCV):  Mean ± SD (m*s-1): 0.8243 ± 0.1941  TWAS (PCV):  Mean ± SD (m*s-1): 0.7801 ± 0.2076 | Both LPTs were valid measures of ACV, however all statistical models demonstrated greater accuracy and consistency of OBS versus TWAS for ACV. Ultimately, the OBS was a valid measure of PCV, while TWAS was not. |
| Illera-Dominguez et al. (2022) | The purpose of the study was to investigate the validity of a low-cost friction encoder against a criterion measure (strain gauge combined with a linear encoder) for assessing velocity, force and power in flywheel exercise devices. Ten young and physically active volunteers performed two sets of 14 maximal squats on a flywheel inertal device (YoYo Technology, Stockholm, Sweden) with five minutes rest between each set. Two different resistances were used (0.075 kg·m2 for the first set; 0.025 kg·m2 for the second). Mean velocity (Vrep), force (Frep) and power (Prep) for each repetition were assessed simultaneously via a friction encoder (Chronojump, Barcelona, Spain), and with a strain gauge combined with a linear encoder (MuscleLab 6000, Ergotest Technology, Porsgrunn, Norway). | squat | ICC:  High load Vrep= 0.97  Low load Vrep= 0.98  Total load Vrep= 0.98 | Based on these findings, the friction encoder provides valid measures of velocity, force and power in flywheel exercise devices. However, as error did exist between measures, the same testing protocol should be used when assessing changes in these parameters over time, or when aiming to perform inter-individual comparisons. |
| Martinez-Cava et al. (2020) | This study examined the inter- and intra-device agreement of four new devices for barbell velocity measurement. Mean, mean propulsive, and peak velocities were recorded for bench press and squat exercises across the entire load-velocity spectrum. Measurements were simultaneously taken using two linear velocity transducers (T-Force), two linear position transducers (Speed4Lifts), two smartphone-based video systems (My Lift), and a 3D motion analysis system (STT). Calculations included (ICC), Bland-Altman LoA, SEM, SDC, and MaxError. Fifteen male participants (27.0 ± 3.8 years) completed two sessions for bench press and squat exercises, separated by 48 hours. Each session involved one repetition with eight increasing loads (25–95 kg, 10-kg increments, 5 min rest). | bench press, full squat | Intra-device agreement:  Bench press  T-Force: PV: ICC= 1.000, CCC= 1.000, r= 0.9998; MPV: ICC= 1.000, CCC= 1.000, r= 0.9999; MV: ICC= 1.000, CCC= 1.000, r=0.9999  Speed4Lifts: PV: ICC= 1.000, CCC= 0.999; MPV: ICC= 0.999, CCC= 0.999; MV: -  MyLift: PV: ICC= 0.993, CCC= 0.986; MPV: - ; MV: -  Full Squat  T-Force: PV: ICC= 1.000, CCC= 1.000, r=0.9997; MPV: ICC= 1.000, CCC= 1.000, r=0.9998; MV: ICC= 1.000, CCC= 1.000, r=0.9998  Speed4Lifts: PV: ICC= 0.999, CCC= 0.998; MPV: ICC= 0.999, CCC= 0.999; MV: -  MyLift: PV: ICC= 0.972, CCC= 0.945; MPV: - ; MV: -  Inter-device agreement:  Bench Press  Speed4Lifts: PV: ICC= 0.995, CCC= 0.991; MPV: ICC= 0.999, CCC= 0.997, r=0.9985; MV: -  MyLift: PV: ICC= 0.991, CCC= 0.981, r=0.9921; MPV: - ; MV: -  STT: PV: ICC= 0.994, CCC=0.988; MPV: -; MV: ICC= 0.998, CCC= 0.995, r=0.9990  Full Squat  Speed4Lifts: PV: ICC= 0.997, CCC= 0.994; MPV: ICC= 0.995, CCC= 0.991, r=0.9936; MV: -  MyLift: PV: ICC= 0.937, CCC= 0.890, r=0.9627; MPV: - ; MV: -  STT: PV: ICC= 0.981, CCC= 0.963; MPV: - ; MV: ICC= 0.999, CCC= 0.995, r=0.9983 | T-Force stands as the preferable option to assess barbell velocity and to identify technical errors of measurement for emerging monitoring technologies. Both the Speed4Lifts and STT are fine alternatives to T-Force for measuring velocity against high-medium loads, while the excessive errors of the newly updated My Lift app advise against the use of this tool for velocity-based resistance training. |
| Pelka et al. (2023) | The purpose of this investigation was to compare concentric movement velocity (CMV) measured with the PUSH Band (v2.0) and a Vicon motion capture system (MC) during the back squat (SQ) and the bench press (BP) (RE). Twelve resistance-trained males (26.0 ± 5.5 years; 175.6 ± 4.9 cm; 96.3 ± 15.8 kg) completed ten repetitions at 50% of one-repetition maximum (1RM), and six repetitions at 75% 1RM for both BP and SQ. Four PUSH devices were utilized and attached to the subject’s right forearm, the center barbell, left and right sides of the barbell. | bench press, squat | Bench Press  50% - Arm: ICC=0.961, r=0.949  50% - CB: ICC=0.922, r=0.871  50% - LB: ICC=0.809, r=0.722  50% - RB: ICC=0.828, r=0.773  75% - Arm: ICC=0.936, r=0.880  75% - CB: ICC=0.890, r=0.847  75% - LB: ICC=0.910, r=0.839  75% - RB: ICC=0.867, r=0.878  Squat  50% - Arm: ICC=0.931, r=0.870  50% - CB: ICC=0.955, r=0.947  50% - LB: ICC=0.889, r=0.815  50% - RB: ICC=0.894, r=0.912  75% - Arm: ICC=0.852, r=0.742  75% - CB: ICC=0.835, r=0.831  75% - LB: ICC=0.835, r=0.863  75% - RB: ICC=0.671, r=0.826 | The measurement of CMV between MC and PUSH compares favorably during moderate (i.e., 50%) and high (75%) intensity SQ and BP RE. These data indicate individuals can use the PUSH band v2.0 to accurately monitor CMV within a RE set for SQ and BP RE. |
| Stock et al. (2011) | The purpose of this study was to calculate test–retest reliability statistics for peak barbell velocity during the free weight bench-press exercise for loads corresponding to 10 - 90% of the 1-repetition maximum (1RM). Twenty-one healthy, resistance-trained men (mean 6 SD age = 23.5 ± 2.7 years; body mass = 90.5 ± 14.6 kg; 1RM bench press = 125.4 ± 18.4 kg) volunteered for this study. A minimum of 48 hours after a maximal strength testing and familiarization session, the subjects performed single repetitions of the free-weight bench press exercise at each tenth percentile (10–90%) of the 1RM on 2 separate occasions. | bench press | 10%: ICC=0.717, p-Value=0.690  20%: ICC=0.572, p-Value=0.378  30%: ICC=0.805, p-Value=0.423  40%: ICC=0.669, p-Value=0.216  50%: ICC=0.790, p-Value=0.678  60%: ICC=0.785, p-Value=0.644  70%: ICC=0.811, p-Value=0.109  80%: ICC=0.714, p-Value=0.618  90%: ICC=0.564, p-Value=0.315 | There were no mean differences between the barbell velocity values from trials 1 and 2. These results indicated moderate to high test–retest reliability for barbell velocity from 10 to 70% 1RM but decreased consistency at 80 and 90% 1RM. When examining barbell velocity during the free-weight bench-press exercise, greater measurement error must be overcome at 80 and 90% 1RM to be confident that an observed change is meaningful. |
| Boehringer, S. & Whyte D. G. (2019) | This study assessed validity and reliability of the 1080 Quantum (1080Q) during the bench press exercise. Twenty-seven resistance-trained men (28 ± 4 years; body mass 88.9 ± 12.8 kg; 1 repetition maximum [1RM] bench press 94.8 ± 10.7 kg) completed 2 test-retest sessions, separated by 1 week. In each session, subjects performed single repetitions at 30, 40, 50, 60, 70,and 80% of their bench press 1RM. Mean Velocity (Vmean), peak velocity (Vpeak), mean force (Fmean), peak force (Fpeak), mean power (Pmean), and peak power (Ppeak) were simultaneously assessed using the 1080Q Synchro and a linear position transducer (GymAware). | bench press | MV:  1080Q (m*s^-1^) vs GXM(m*s^-1^): r=1.00  PV:  1080Q (m*s^-1^) vs GXM(m*s^-1^): r=1.00  MV:  Overall: 0.97  30%: ICC=0.64  40%: ICC=0.82  50%: ICC=0.53  60%: ICC=0.45  70%: ICC=0.63  80%: ICC=0.69  PV:  Overall: 0.97  30%: ICC=0.61  40%: ICC=0.84  50%: ICC=0.68  60%: ICC=0.75  70%: ICC=0.58  80%: ICC=0.80 | The high validity observed for Vmean and Vpeak means coaches and athletes can confidently use the 1080Qtomonitorvelocityonarep-by-rep basis, as part of a velocity-based training program. The high reliability of measures obtained with the 1080Q also means it has the capability to identify small but important improve ments in force production across a range of velocities, making the 1080Q a useful tool for testing the efficacy of a RT program. However, strength and conditioning practitioners should be cautious when comparing force and power data from the 1080Q with that from other testing equipment, as systematic and proportional biases exist. |
| Qu et al. (2024) | This study evaluated the validity and test-retest reliability of a resistance training device Jueying (Beijing, China) for Smith machine back squat exercise. Twelve male participants completed two test sessions with an interval of one week. In each test session, participants completed 30%, 45%, 60%, and 75% of 1RM back squats on a Smith machine equipped with Jueying and a linear position transducer GymAware (Canberra, Australia), which measured the velocity and power during the movement simultaneously. | back squat | Validity assessment of individual loads for Jueying:  Vpeak (m/s):  30% 1 RM: r= 0.999  45% 1 RM: r= 0.998  60% 1 RM: r= 0.991  75% 1 RM: r= 0.989  Ppeak (w):  30% 1 RM: r= 0.993  45% 1 RM: r= 0.996  60% 1 RM: r= 0.990  75% 1 RM: r= 0.976  Vmean (m/s):  30% 1 RM: r= 0.986  45% 1 RM: r= 0.974  60% 1 RM: r= 0.896  75% 1 RM: r= 0.925  Pmean (w):  30% 1 RM: r= 0.986  45% 1 RM: r= 0.973  60% 1 RM: r= 0.927  75% 1 RM: r= 0.911  Test-retest reliability assessment of individual loads for Jueying:  Vpeak (m/s):  30% 1 RM: ICC= 0.89  45% 1 RM: ICC= 0.95  60% 1 RM: ICC= 0.91  75% 1 RM: ICC= 0.79  Ppeak (w):  30% 1 RM: ICC= 0.91  45% 1 RM: ICC= 0.95  60% 1 RM: ICC= 0.91  75% 1 RM: ICC= 0.84  Vmean (m/s):  30% 1 RM: ICC= 0.83  45% 1 RM: ICC= 0.89  60% 1 RM: ICC= 0.82  75% 1 RM: ICC= 0.88  Pmean (w):  30% 1 RM: ICC= 0.82  45% 1 RM: ICC= 0.92  60% 1 RM: ICC= 0.89  75% 1 RM: ICC= 0.90 | Jueying provides a function of measuring velocity and power during resistance training. As far as Smith machine back squat training is concerned, its validity and reliability have been verified. The device can be applied to sports practice, and it can provide effective and reliable data to provide a reference for the arrangement of training. |
| Suchomel et a. (2023) | This study examined the reliability, potential bias, and practical differences between the GymAwarePowertool (GA), Tendo Power Analyzer (TENDO), and Push Band 2.0 (PUSH) during the jump shrug (JS) and hang high pull (HHP) performed across a spectrum of loads. Fifteen resistance trained men (age: 25.5 ± 4.5years) performed JS and HHP repetitions with 20, 40, 60, 80, and 100% of their 1RM hang power clean, and mean (MBV) and peak barbell velocity (PBV) were determined by each velocity measurement device. Least-products regression and Bland–Altman plots were used to examine instances of proportional, fixed, and systematic bias between the TENDO and PUSH compared to the GA. Hedge’s g effect sizes were also calculated to determine any meaningful differences between devices. | Jump Shrug & Hang high pull | Test-retest reliability for the GA, TENDO, PUSH  MBV = mean barbell velocity  PBV= peak barbell velocity  Jump Shrug:  MBV vs. GA:  20%: ICC= 0.91, CI=0.78-0.97  40%: ICC= 0.96, CI= 0.91-0.99  60%: ICC=0.96, CI= 0.89-0.98  80%: ICC= 0.95, CI= 0.87-0.98  100%: ICC= 0.97, CI= 0.93-0.99  PBV vs. GA:  20%: ICC= 0.97, CI= 0.93-0.99  40%: ICC= 0.97, CI= 0.93-0.99  60%: ICC=0.97, CI= 0.92-0.99  80%: ICC= 0.96, CI= 0.90-0.99  100%: ICC= 0.96, CI= 0.89-0.98  MBV vs. TENDO:  20%: ICC= 0.94, CI= 0.85-0.98  40%: ICC= 0.95, CI= 0.87-0.98  60%: ICC=0.96, CI= 0.90-0.98  80%: ICC= 0.97, CI= 0.93-0.99  100%: ICC= 0.98, CI= 0.97-0.99  PBV vs. TENDO:  20%: ICC= 0.98, CI= 0.94-0.99  40%: ICC= 0.97, CI= 0.94-0.99  60%: ICC=0.93, CI= 0.84-0.98  80%: ICC= 0.96, CI= 0.90-0.98  100%: ICC= 0.95, CI= 0.88-0.98  MBV vs. PUSH:  20%: ICC= 0.91, CI= 0.77-0.97  40%: ICC= 0.90, CI= 0.77-0.96  60%: ICC=0.89, CI= 0.73-0.96  80%: ICC= 0.94, CI= 0.86-0.98  100%: ICC= 0.79, CI= 0.49-0.92  PBV vs. PUSH.  20%: ICC= 0.96, CI= 0.91-0.99  40%: ICC= 0.96, CI= 0.91-0.99  60%: ICC=0.89, CI= 0.75-0.96  80%: ICC= 0.96, CI= 0.91-0.99  100%: ICC= 0.69, CI= 0.26-0.89  Hang High Pull:  MBV vs. GA:  20%: ICC= 0.95, CI= 0.89-0.99  40%: ICC= 0.97, CI= 0.93-0.99  60%: ICC=0.96, CI= 0.90-0.98  80%: ICC= 0.98, CI= 0.94-0.99  100%: ICC= 0.98, CI= 0.95-0.99  PBV vs. GA:  20%: ICC= 0.96, CI= 0.92-0.99  40%: ICC= 0.95, CI= 0.87-0.98  60%: ICC=0.95, CI= 0.89-0.98  80%: ICC= 0.98, CI= 0.96-0.99  100%: ICC= 0.98, CI= 0.95-0.99  MBV vs. TENDO:  20%: ICC= 0.97, CI= 0.92-0.99  40%: ICC= 0.97, CI= 0.93-0.99  60%: ICC=0.96, CI= 0.90-0.99  80%: ICC= 0.96, CI= 0.92-0.99  100%: ICC= 0.97, CI= 0.92-0.99  PBV vs. TENDO:  20%: ICC= 0.92, CI= 0.82-0.97  40%: ICC= 0.93, CI= 0.83-0.97  60%: ICC=0.94, CI= 0.86-0.98  80%: ICC= 0.97, CI= 0.93-0.99  100%: ICC= 0.96, CI= 0.90-0.99  MBV vs. PUSH:  20%: ICC= 0.90, CI= 0.75-0.96  40%: ICC= 0.88, CI= 0.73-0.96  60%: ICC=0.94, CI= 0.85-0.98  80%: ICC= 0.94, CI= 0.84-0.98  100%: ICC= 0.94, CI= 0.86-0.98  PBV vs. PUSH:  20%: ICC= 0.65, CI= 0.14-0.88  40%: ICC= 0.38, CI= -0.47-0.78  60%: ICC=0.90, CI= 0.76-0.96  80%: ICC= 0.88, CI= 0.70-0.96  100%: ICC= 0.92, CI= 0.81-0.97 | The GA and TENDO displayed excellent reliability and acceptable variability during the JS and HHP while the PUSH showed instances of poor–moderate reliability and unacceptable variability at various loads. While the TENDO and PUSH showed instances of various bias, the TENDO device demonstrated greater validity when compared to the GA. The results also indicate that the PUSH device may underestimate MBV and PBV compared to the GA during the HHP, and in some instances provide inaccurate velocity metrics at higher loads. From a practical standpoint, it appears that both the GA and TENDO may be used to reliably assess MBVandPBVduringboththe JS and HHPacross an entire loading spectrum. |
| Chéry & Ruf (2019) | This study investigated the reliability between load and mean velocity, mean propulsive velocity, peak velocity, mean power, and peak power in the deadlift. Also, we looked at the validity of an inertial sensor (PUSH) and a linear-position transducer (Tendo) to measure velocity variables. Ten strength-trained men (23.4 ± 1.3 years) were involved in three 1 repetition maximum (1RM) testing sessions, separated by at least 72 hours. The protocol used 6 different lifting intensities, comprising 20, 40, 60, 80, 90, and 100% of 1RM. Reliability of the load-velocity and load power relationship was assessed by comparing velocity and power measurements from session 2 and 3 for each relative %1RM. The validity of tested devices was analyzed regarding to each relative intensity by comparing results from each instrument to a reference instrument (GymAware). To analyze PUSH and Tendo validity, the raw difference between GymAware and tested device, as well as the magnitude of Pearson product-moment correlation r was determined. | Deadlift | Validity of MV and PV for the Tendo and PUSH compared with GymAware:  Correlation (95% CL)  PUSH vs. GymAware:  MV:  20%: r=0.47  40%: r=0.50  60%: r=0.30  80%: r=0.29  90%: r=0.66  100%: r=-0.20  All: r=0.90  PUSH vs. GymAware:  PV:  20%: r=0.67  40%: r=0.68  60%: r=0.63  80%: r=0.64  90%: r=0.89  100%: r=0.20  All: r=0.92  Tendo vs. GymAware:  MV:  20%: r=0.91  40%: r=0.89  60%: r=0.78  80%: r=0.94  90%: r=0.94  100%: r=-0.97  All: r=1.0  Tendo vs. GymAware:  PV:  20%: r=0.98  40%: r=0.95  60%: r=0.98  80%: r=0.99  90%: r=1.0  100%: r=0.99  All: r=1.0 | A Tendo weightlifting analyzer and a GymAware. Because, as both devices have their own validation studies, we cannot draw any conclusions on the validity of one over the other. However, when all 120 repetitions were combined for analysis, no statistical differences were found between these devices. Nevertheless, the analysis of each relative intensity revealed disparity in measurements at 80 and 90% of 1RM for MV and at 20, 40, and 60% of 1RM for PV combined to a moderate to large ES. They do not recommend the PUSHed with GymAware, Tendo measurements were significantly different at 80 and 90% of 1RM for MV and at 20, 40, and 60% of 1RM for PV. |
| Fritschi et al. (2021) | This study assessed the validity of several mobile and one stationary VBT device for measuring mean and peak concentric barbell velocity over a range of velocities and exercises, including low- and high-velocity, ballistic and non-ballistic, and plyometric and non-plyometric movements, and to quantify the isolated effect of device attachment point on measurement validity. Eleven men (28 ± 5 years old) and three women (24 ± 2 years old) took part in the study. | back squat | GymAware: MV: r=0.99; PV: r=0.99  Vmaxpro: MV: r=0.99; PV: r=0.99  Quantum: MV: r=0.97; PV: r=1.0  Flex: MV: r=0.96; PV: r=0.96  Push: MV: r=0.97; PV: r=98 | This study reconfirmed the high validity of the GymAware, expanding findings to previously unexplored ballistic and plyometric exercises. The stationary 1080 Quantum system also displayed excellent validity characteristics for the wide range of free-weight exercises and velocities, and these two missed a repetition least frequently among the devices tested. With this in mind, it was somewhat surprising that the second-best mobile device in the current study, only slightly behind GymAware, was also the cheapest: Vmaxpro. The Vmaxpro is also an IMU and the smallest of the tested devices, making it quicker to install and more versatile than the others. |
| Held et al. (2021) | The validity and reliability indicators of an easy-to-use inertial measurement unit (VmaxPro®) were examined. Nineteen trained males (23.1 ± 3.2 years) performed squats and hip thrusts (3–5 sets, 30 repetitions total, 75% 1RM) on two separate days. The MCV and displacement were simultaneously measured using VmaxPro® and a linear position transducer (Speed4Lift®). | squat & hip thrusts | Validity of MCV between S4L and VMP:  Squat:  MCV (m*s-1): ICC=0.94, CI=0.92-0.95; CC=0.96, CI=0.95-0.96  Hip Thrust:  MCV (m*s-1): ICC= 0.85, CI= 0.70-0.92; CC= 0.91, CI= 0.91-0.92  Within- and between-day reliability for S4L and VMP sensors for squat (SQ) and hip thrust (HT) exercises:  S4L:  Within-day MVC (m*s-1): ICC=0.91, CI=0.78-0.97; CC=0.92, CI=0.80-0.97  Between-day MVC (m*s-1): ICC= 0.75, CI=0.56-0.87; CC=0.75, CI=0.85-0.87  HT:  Within-day MVC (m*s-1): ICC=0.93, CI=0.83-0.97; CC=0.93, CI=0.83-0.97  Between-day MVC (m*s-1): ICC= 0.56, CI=0.22-0.74; CC=0.56, CI=0.26-0.76  VMP:  SQ:  Within-day MVC (m*s-1): ICC=0.88, CI=0.71-0.95; CC=0.87, CI=0.69-0.95  Between-day MVC (m*s-1): ICC= 0.82, CI=0.67-0.90; CC=0.81, CI=0.66-0.90  HT:  Within-day MVC (m*s-1): ICC=0.80, CI=0.56-0.92; CC=0.81, CI=0.57-0.93  Between-day MVC (m*s-1): ICC= 0.55, CI=0.25-0.75; CC=0.55, CI=0.24-0.75 | The IMU-based VmaxPro® sensor showed good to excellent validity with relatively low limits of agreements for the mean concentric velocity during squats and hip thrusts; there was a comparable (within- and between-day) reliability with previously validated linear position transducers (Speed4Lift®) for the mean concentric velocity during squats and hip thrusts. The commercially available IMU-based VmaxPro® sensor can be considered to be a valid and reliable measurement tool for the mean concentric velocity. For the barbell displacement measurements, the high limits of agreement seriously restrict the practical use of the IMU-based VmaxPro® sensor. |
| González-Galán et al. (2024) | This study aimed to analyze the intra-device agreement of a new linear position transducer (Vitruve, VT) and the inter-device agreement with a previously validated linear velocity transducer (T-Force System, TF) in different range of velocities. A group of 50 healthy, physically active men (25 ±3.3 years) performed a progressive loading test during a bench press (BP) and full-squat (SQ) exercise with a simultaneous recording of two VT and one TF devices. The established velocity ranges were as follows: >1.20 m·s−1; 1.20–0.95 m·s−1; 0.95–0.70 m·s−1; 0.70–0.45 m·s−1; ≤0.45 m·s−1 for BP; and >1.50 m·s−1; 1.50–1.25 m·s−1; 1.25–1.00 m·s−1; 1.00–0.75 m·s−1; and ≤0.75 m·s−1 for SQ. | bench press & squat | Between-device agreement (reproducibility) for the maximal velocity:  pooled:  TF-VT1: ICC=0.990, CI=0.988-0.993  TF-VT2: ICC=0.995, CI=0.993-0.996  VT1-VT2: ICC=0.998, CI=0.998-0.999  >1.20 m*s-1:  TF-VT1: ICC=0.754, CI=0.551-0.865  TF-VT2: ICC=0.847, CI=0.718-0.917  VT1-VT2: ICC=0.972, CI=0.949-0.985  1.20 m*s-1 - 0.95 m*s-1:  TF-VT1: ICC=0.832, CI=0.672-0.914  TF-VT2: ICC=0.919, CI=0.840-0.959  VT1-VT2: ICC=0.970, CI=0.941-0.985  0.95 m*s-1 - 0.70 m*s-1:  TF-VT1: ICC=0.941, CI=0.904-0.964  TF-VT2: ICC=0.977, CI=0.963-0.986  VT1-VT2: ICC=0.976, CI=0.961-0.985  0.70 m*s-1 - 0.45 m*s-1:  TF-VT1: ICC=0.978, CI=0.962-0.987  TF-VT2: ICC=0.986, CI=0.976-0.992  VT1-VT2: ICC=0.988, CI=0.980-0.993  <0.45 m*s-1:  TF-VT1: ICC=0.995, CI=0.992-0.997  TF-VT2: ICC=0.996, CI=0.993-0.998  VT1-VT2: ICC=0.991, CI=0.984-0.995  Between-device agreement (reproducibility) for the mean propulsive velocity in the bench press:  pooled:  TF-VT1: ICC=0.998, CI=0.997-0.998  TF-VT2: ICC=0.995, CI=0.994-0.996  VT1-VT2: ICC=0.999, CI=0.999-0.999  >1.20 m*s-1:  TF-VT1: ICC=0.963, CI=0.933-0.980  TF-VT2: ICC=0.918, CI=0.851-0.955  VT1-VT2: ICC=0.980, CI=0.964-0.989  1.20 m*s-1 - 0.95 m*s-1:  TF-VT1: ICC=0.873, CI=0.753-0.935  TF-VT2: ICC=0.790, CI=0.590-0.892  VT1-VT2: ICC=0.950, CI=0.902-0.975  0.95 m*s-1 - 0.70 m*s-1:  TF-VT1: ICC=0.930, CI=0.884-0.957  TF-VT2: ICC=0.883, CI=0.807-0.929  VT1-VT2: ICC=0.977, CI=0.961-0.986  0.70 m*s-1 - 0.45 m*s-1:  TF-VT1: ICC=0.963, CI=0.940-0.977  TF-VT2: ICC=0.948, CI=0.911-0.970  VT1-VT2: ICC=0.973, CI=0.953-0.985  <0.45 m*s-1:  TF-VT1: ICC=0.992, CI=0.987-0.995  TF-VT2: ICC=0.986, CI=0.976-0.992  VT1-VT2: ICC=0.995, CI=0.992-0.997  Between-device agreement (reproducibility) for the mean propulsive velocity in the full squat:  pooled:  TF-VT1: ICC=0.998, CI=0.997-0.998  TF-VT2: ICC=0.999, CI=0.999-0.999  VT1-VT2: ICC=0.999, CI=0.999-0.999  >1.50 m*s-1:  TF-VT1: ICC=0.946, CI=0.912-0.967  TF-VT2: ICC=0.926, CI=0.874-0.956  VT1-VT2: ICC=0.916, CI=0.856-0.951  1.50 m*s-1 - 1.25 m*s-1:  TF-VT1: ICC=0.909, CI=0.860-0.941  TF-VT2: ICC=0.940, CI=0.906-0.962  VT1-VT2: ICC=0.961, CI=0.939-0.975  1.25 m*s-1 - 1.00 m*s-1:  TF-VT1: ICC=0.964, CI=0.945-0.976  TF-VT2: ICC=0.981, CI=0.972-0.988  VT1-VT2: ICC=0.968, CI=0.952-0.979  1.00 m*s-1 - 0.75 m*s-1:  TF-VT1: ICC=0.966, CI=0.949-0.978  TF-VT2: ICC=0.974, CI=0.961-0.983  VT1-VT2: ICC=0.980, CI=0.970-0.987  <0.75 m*s-1:  TF-VT1: ICC=0.992, CI=0.988-0.995  TF-VT2: ICC=0.996, CI=0.994-0.998  VT1-VT2: ICC=0.996, CI=0.993-0.997  Between-device agreement (reproducibility) for the maximal velocity in the full squat:  pooled:  TF-VT1: ICC=0.966, CI=0.958-0.972  TF-VT2: ICC=0.970, CI=0.963-0.975  VT1-VT2: ICC=0.986, CI=0.982-0.988  >1.50 m*s-1:  TF-VT1: ICC=0.695, CI=0.516-0.809  TF-VT2: ICC=0.696, CI=0.506-0.813  VT1-VT2: ICC=0.849, CI=0.756-0.907  1.50 m*s-1 - 1.25 m*s-1:  TF-VT1: ICC=0.812, CI=0.710-0.878  TF-VT2: ICC=0.866, CI=0.790-0.914  VT1-VT2: ICC=0.882, CI=0.816-0.925  1.25 m*s-1 - 1.00 m*s-1:  TF-VT1: ICC=0.885, CI=0.827-0.923  TF-VT2: ICC=0.923, CI=0.884-0.949  VT1-VT2: ICC=0.986, CI=0.978-0.990  1.00 m*s-1 - 0.75 m*s-1:  TF-VT1: ICC=0.903, CI=0.851-0.937  TF-VT2: ICC=0.958, CI=0.935-0.973  VT1-VT2: ICC=0.977, CI=0.964-0.985  <0.75 m*s-1:  TF-VT1: ICC=0.920, CI=0.871-0.950  TF-VT2: ICC=0.970, CI=0.951-0.981  VT1-VT2: ICC=0.971, CI=0.954-0.983 | The results suggest that the VT system should only be used at MPVs below 0.45 m·s−1 for BP and 0.75 m·s−1 for SQ in order to obtain an accurate and reliable measurement, preferably using the MPV variable instead of the PV. Therefore, it appears that the VT system may not be appropriate for objectively monitoring resistance training and assessing strength performance along the entire spectrum of load-velocity curve. |
| Pueo et al. (2021) | A custom image-processing algorithm allows for the detection of reference points of a multipower machine to autocalibrate and automatically track barbell markers to give real-time kinematic-derived parameters. Validity and reliability were studied by comparing the simultaneous measurement of 160 repetitions of back squat lifts executed by 20 athletes (23.6 4.1 y) with the proposed instrument and a validated linear position transducer (LPT), used as a criterion. | back squat | Reliability of video and LPT:  ICC (2,1):  Range: 0.996 (0.881-0.999)  Velocity: 0.988 (0.542-0.997)  Force: 0.978 (0.370-0.994)  Power:0.979 (0.296-0.995)  Range LPT vs. Range video:  r= 0.999  Velocity LPT (m/s) vs Velocity Video (m/s):  r=0.997  Force LPT (N):  r=0.996  Power LPT (W) vs. Power Video (W):  r=0.996 | Our results suggest that the developed video system is a valid, reliable, and trustworthy instrument for measuring velocity and derived variables accurately with practical implications for use by coaches and practitioners. |
| Garnacho-Castano et al. (2015) | The objectives of the study were to determine the validity and reliability of peak velocity (PV), average velocity (AV), peak power (PP) and average power (AP) measurements were made using a linear position transducer. Validity was assessed by comparing measurements simultaneously obtained using the Tendo Weightlifting Analyzer System and T-Force Dynamic Measurement System (Ergotech, Murcia, Spain) during two resistance exercises, bench press (BP) and full back squat (BS), performed by 71 trained male subjects (21.6 ± 2.1 years). For the reliability study, a further 32 men (20.7 ± 3.4 years) completed both lifts using the Tendo Weight-lifting Analyzer System in two identical testing sessions one week apart (session 1 vs. session 2). | bench press & full back squat | Concurrent validity (Tendo Weight-lifting (TWAS) vs. T-Force Dynamic measurement System (TFDMS):  Back squat  AV (m*s-1): ICC=0.985  PV (m*s-1): ICC=0.963  Bench press  AV (m*s-1): ICC=0.989  PV (m*s-1): ICC=0.984  Test-retest reliability of the TWAS. Average values of AV (average velocity) and PV (peak velocity):  Back squat  AV (m*s-1): ICC=0.982  PV (m*s-1): ICC=0.966  Bench press  AV (m*s-1): ICC=0.977  PV (m*s-1): ICC=0.988 | The Tendo Weightlifting Analyzer System emerged as a reliable system for measuring movement velocity and estimating power in resistance exercises. The low biases and random errors observed here (mainly AV, AP) make this device a useful tool for monitoring resistance training. |
| Sousa et al. (2024) | The current study aimed to analyze the validity and reliability of the T-Force and Chronojump systems to measure the movement velocity in the leg press (LP) and chest press (CP) exercises in older people. Eighteen older adults (6 men and 12 women, 79.9 ± 8.5 years) performed a set of procedures over three weeks: (i) the first week was to familiarize participants with the testing procedures, (ii) the second was to perform a progressive loading test until reaching one-repetition maximum (1RM) in the LP and CP, and (iii) in the third week, participants performed three repetitions against five loads (40, 50, 60, 70, and 80% of 1RM). The mean velocity of each repetition was recorded simultaneously through the T-Force and Chronojump devices. | chest press & leg press | inter-device reliability in measuring mean velocity:  chest press:  40% : ICC=0.97, CI=0.92-0.99  50% : ICC=0.96, CI=0.90-0.98  60% : ICC=0.96, CI=0.89-0.98  70% : ICC=0.95, CI=0.86-0.98  80%: ICC=0.95, CI=0.86-0.98  r²=0.972  leg press:  40% : ICC=0.97, CI=0.91-0.99  50% : ICC=0.98, CI=0.94-0.99  60% : ICC=0.98, CI=0.95-0.99  70% : ICC=0.98, CI=0.94-0.99  80%: ICC=0.97, CI=0.93-0.99  r²=0.978  intra-device reliability in measuring mean velocity:  chest press  chronojump:  40% : ICC=0.96, CI=0.90-0.99  50% : ICC=0.94, CI=0.85-0.98  60% : ICC=0.92, CI=0.78-0.97  70% : ICC=0.96, CI=0.89-0.99  80%: ICC=0.94, CI=0.85-0.98  T-Force:  40% : ICC=0.95, CI=0.88-0.98  50% : ICC=0.94, CI=0.83-0.98  60% : ICC=0.90, CI=0.74-0.96  70% : ICC=0.96, CI=0.89-0.99  80%: ICC=0.96, CI=0.89-0.98  leg press  chronojump:  40% : ICC=0.95, CI=0.88-0.98  50% : ICC=0.97, CI=0.91-0.99  60% : ICC=0.94, CI=0.84-0.98  70% : ICC=0.94, CI=0.85-0.98  80%: ICC=0.95, CI=0.86-0.98  T-Force:  40% : ICC=0.97, CI=0.92-0.99  50% : ICC=0.97, CI=0.93-0.99  60% : ICC=0.94, CI=0.83-0.98  70% : ICC=0.95, CI=0.88-0.98  80%: ICC=0.95, CI=0.87-0.98 | This study shows that the T-Force and Chronojump devices are highly valid and reliable for measuring movement velocity in the leg and chest press exercises in older adults. The Chronojump LPT can be a valid and reliable alternative to the T-Force LVT, considering the low measurement errors and similar mean velocity values observed during the execution of the leg and chest press exercises in older adults, especially for relative loads between 50 and 80% 1RM. |
| Garcia Ramos et al. (2018) | This study aimed to examine the reliability of different power and velocity variables during the Smith machine bench press (BP) and bench press throw (BPT) exercises. Twenty-two healthy (age 21.7 ± 3.0 years) men conducted four testing sessions after a preliminary BP one-repetition maximum (1RM) test. In a counterbalanced order, participants performed two sessions of BP in one week and two sessions of BPT in another week. Mean propulsive power, peak power, mean propulsive velocity, and peak velocity at each tenth percentile (20–70% of 1RM) were recorded by a linear transducer. | bench press & bench press throw | test-retest reliability of barbell’s MPV in BP and BPT:  BP20: ICC= 0.791, CI (90%)=0.57-0.90  BP30: ICC= 0.843, CI=0.67-0.93  BP40: ICC= 0.920, CI=0.84-0.96  BP50: ICC= 0.915, CI=0.82-0.96  BP60: ICC= 0.894, CI=0.78-0.95  BP70: ICC= 0.780, CI=0.49-0.90  BPT20: ICC= 0.896, CI=0.78-0.95  BPT30: ICC= 0.867, CI=0.72-0.94  BPT40: ICC= 0.836, CI=0.66-0.92  BPT50: ICC= 0.909, CI=0.81-0.96  BPT60: ICC= 0.855, CI=0.70-0.93  BPT70: ICC= 0.853, CI=0.36-0.95  test-retest reliability of barbell’s PV in BP and BPT:  BP20: ICC= 0.827, CI (90%)=0.64-0.92  BP30: ICC= 0.921, CI=0.84-0.96  BP40: ICC= 0.840, CI=0.65-0.92  BP50: ICC= 0.928, CI=0.84-0.97  BP60: ICC= 0.894, CI=0.77-0.95  BP70: ICC= 0.860, CI=0.62-0.94  BPT20: ICC= 0.962, CI=0.92-0.98  BPT30: ICC= 0.923, CI=0.84-0.96  BPT40: ICC= 0.863, CI=0.72-0.93  BPT50: ICC= 0.946, CI=0.89-0.97  BPT60: ICC= 0.895, CI=0.78-0.95  BPT70: ICC= 0.942, CI=0.82-0.98 | The four variables examined in the present study met the criteria for acceptable reliability, suggesting that all of them can be employed for monitoring an athlete’s performance during the Smith machine BP and BPT exercises. (1) velocity variables seem to be more reliable than power variables; (2) peak values seem to be more reliable than mean propulsive values; and (3) the BPT seem to be slightly more reliable than the BP, especially with the lighter loads. |
| Perez-Castilla et al. (2018) | This study aimed to compare the between-session reliability of three typically measured velocity variables (mean velocity [MV], mean propulsive velocity [MPV], and maximum velocity [Vmax]) to assess vertical jump performance. Totally, 23 men had their squat jump (SJ) and countermovement jump (CMJ) tested against five different loading conditions (17, 30, 45, 60 and 75 kg) during two consecutive weeks. The two sessions of each jump type were performed within the same week separated by 48–72 h. | loaded squat jump & countermovement jump | Test-retest reliability of different velocity variables during Squat Jump (SJ) and countermovement jump (CMJ) exercises:  MV in SJ:  17 kg: ICC= 0.77 (0.53-0.90)  30 kg: ICC= 0.83 (0.63-0.92)  45 kg: ICC= 0.92 (0.82-0.97)  60 kg: ICC= 0.89 (0.76-0.95)  75 kg: ICC= 0.90 (0.77-0.95)  MPV in SJ:  17 kg: ICC= 0.77 (0.52-0.94)  30 kg: ICC= 0.84 (0.66-0.93)  45 kg: ICC= 0.88 (0.74-0.95)  60 kg: ICC= 0.87 (0.72-0.94)  75 kg: ICC= 0.89 (0.75-0.95)  Vmax in SJ:  17 kg: ICC= 0.91 (0.80-0.96)  30 kg: ICC= 0.94 (0.87-0.97)  45 kg: ICC= 0.95 (0.88-0.98)  60 kg: ICC= 0.95 (0.88-0.98)  75 kg: ICC= 0.93 (0.85-0.97)  MV in CMJ:  17 kg: ICC= 0.91(0.81-0.96)  30 kg: ICC= 0.87(0.71-0.94)  45 kg: ICC= 0.93(0.83-0.97)  60 kg: ICC= 0.95(0.88-0.98)  75 kg: ICC= 0.90(0.77-0.95)  MPV in CMJ:  17 kg: ICC= 0.94(0.86-0.97)  30 kg: ICC= 0.89(0.76-0.95)  45 kg: ICC= 0.92(0.83-0.97)  60 kg: ICC= 0.93(0.85-0.97)  75 kg: ICC= 0.91(0.80-0.96)  Vmax in CMJ:  17 kg: ICC= 0.94(0.87-0.98)  30 kg: ICC= 0.92(0.81-0.96)  45 kg: ICC= 0.94(0.87-0.97)  60 kg: ICC= 0.95(0.88-0.98)  75 kg: ICC= 0.92(0.81-0.96) | The three velocity variables examined in the present study (MV, MPV, and Vmax) met the criteria for acceptable reliability regardless of the vertical jump type and loading condition examined, suggesting that all of these variables could be used for the assessment of lower body ballistic performance. However, the results of this study suggest that the most reliable testing procedure would consist on the evaluation of the Vmax during both vertical jump types. It should also be noted that while no meaningful differences in reliability between the three velocity variables were observed for the CMJ exercise, the reliability of the mean velocity variables (MV and MPV) is compromised during the SJ exercise. |
| San Augustin et al. (2020) | This study aimed to evaluate the validity of the Inertial Measurement System (IMS) to measure the velocity, force, and power during the performance of different hamstring exercises on a flywheel resistance training device. Fifteen males (average age: 22.4 ± 2.5 years; years of strength training: 4.3 ± 2.2 years) performed the bilateral stiff-leg deadlift (SDL), 45 hip extension (HE), and unilateral straight knee bridge (SKB) in two sessions (familiarization and evaluation) with a 1-week interval between them. The velocity, force, and power (average and peak values) in the concentric and eccentric phases for each of the exercises were recorded simultaneously with Inertial Measure System (IMS) and MuscleLab. | stiff-leg deadlift, straight knee bridge & hip extension | Validity between IMS and MuscleLab in the SLD:  concentric:  PV (m/s): r=0.964  AV (m/s): r=0.910  eccentric:  PV (m/s): r=0.965  AV (m/s): r=0.960  Validity between IMS and MuscleLab in the 45° hip extension:  concentric:  PV (m/s): r=0.971  AV (m/s): r=0.866  eccentric:  PV (m/s): r=0.915  AV (m/s): r=0.895  Validity between IMS and MuscleLab in the unilateral SKB:  concentric:  PV (m/s): r=0.957  AV (m/s): r=0.922  eccentric:  PV (m/s): r=0.947  AV (m/s): r=0.854 | The findings prove that the IMS is valid to measure speed and force in the exercises studied and power in the SDL, but contrary to that, various power parameters in HE and SKB does not achieve the minimum validity. Therefore, IMS could have potential practical applications for any professional or athlete who wants to monitor inertial hamstring exercises. |
| van den Tillaar & Ball (2019) | The aim of this study was to compare the validity and reliability of a PUSH band device with a linear encoder to measure movement velocity with different loads during the push-up and bench press exercises. Twenty resistance-trained athletes performed push-up and bench press exercises with four different loads: without weight vest, 10-20-30 kg weight vest, bench press: 50–82% of their assumed 1 repetition maximum (1 RM) in steps of 10 kg. A linear encoder (Musclelab) and the PUSH band measured mean and peak velocity during both exercises. Several statistical analyses were used to investigate the validity and reliability of the PUSH band with the linear encoder. | bench press & push-ups | Push-Up:  linear encoder:  PV: ICC= 0.98 (0.97-0.99), r=0.95  MV: ICC= 0.98 (0.97-0.99), r= 0.95  PUSH band:  PV: ICC= 0.98 (0.97-0.99), r=0.94  MV: ICC= 0.98 (0.97-0.99), r= 0.95  Bench Press:  linear encoder:  PV: ICC= 0.98 (0.98-0.99), r=0.96  MV: ICC= 0.98 (0.97-0.99), r= 0.96  PUSH band:  PV: ICC= 0.92 (0.89-0.95), r=0.81  MV: ICC= 0.95 (0.93-0.97), r= 0.87  Correlation between PUSH Band and linear encoder:  Push-up:  AV linear encoder (m/s) vs. Velocity Push band: r= 0.70  PV linear encoder (m/s) vs. Velocity Push band: r= 0.47  Bench Press:  AV linear encoder (m/s) vs. Velocity Push band: r= 0.62  PV linear encoder (m/s) vs. Velocity Push band: r= 0.49  Bland-Altman Plots between PUSH band and linear encoder measured PV and MV in bench press and push-up:  Push up (AV): r=0.57  Push up (PV): r= 0.46  Bench press (AV): r=-0.07  Bench press (PV): r=0.27 | The PUSH band will show decreased reliability from velocity measures in a bench press exercise and underestimate load-velocity based 1 RM predictions. For training, the PUSH band can be used during push-ups, however caution is suggested when using the device for the purposes of feedback in bench press at increasing loads. |
| Wagner et al. (2025) | This study explores relative reliability values arising from test-retest data collection and additionally provides values for the systematic and random error. 49 youth elite athletes (17.71±2.07 years) were recruited and performed a 1RM test followed by a load velocity profiling test using 30%, 50% and 70% of the 1RM in the bench press and bench pull, respectively. Reliability analysis, ICCs and the coefficient of variability, were calculated and supplemented by an agreement analysis including the mean absolute error (MAE) and mean absolute percentage error (MAPE) to provide the resulting measurement error. Furthermore, validity analyses between the measured 1RM and different calculation models to estimate 1RM were performed. | bench press & bench pull | Intraday Test-Retest reliability of velocity:  Bench Pull:  AV (30%1RM): ICC= 0.845 (0.740–0.909)  PV (30%1RM): ICC= 0.850 (0.749–0.913)  AV (50%1RM): ICC= 0.906 (0.838–0.946)  PV (50%1RM): ICC= 0.974 (0.954–0.985)  AV (70%1RM): ICC= 0.822 (0.703–0.896)  PV (70%1RM): ICC= 0.957 (0.925–0.976)  Bench Press  AV (30%1RM): ICC= 0.734 (0.570–0.841)  PV (30%1RM): ICC= 0.650 (0.453–0.787)  AV (50%1RM): ICC= 0.851 (0.749–0.913)  PV (50%1RM): ICC= 0.853 (0.754–0.915)  AV (70%1RM): ICC= 0.835 (0.726–0.903)  PV (70%1RM): ICC= 0.788 (0.654–0.874) | Considering the observed measurement error associated with LVP-based methods, it can be posited that their utility as a programming strategy is limited. The lack of accuracy required to discriminate between small but significant changes in performance and error, coupled with the potential risks of under- and overestimating 1RM, can result in insufficient stimulus or increased injury risk, respectively. This further diminishes the practicality of these methods, particularly in elite sports settings. |
| Pinzone et al. (2024) | This study evaluated validity and reliability of the HUMAC360 linear position transducer (LPT) compared to the Tendo Sport Weightlifting Analyzer (TENDO) for measuring mean velocity (MV), peak velocity (PV), and displacement (D) during the bench press. Seventeen (n = 12 males/5 females; 24 ± 4 years) recreationally active individuals completed three visits. During visit one, participants were assessed for their one repetition maximum (1RM) bench press. During subsequent visits, participants completed two sets of three repetitions of bench press at 30, 50, 60, and 70% 1RM. | bench press | Validity data:  30% MV (m/s)  ICC:0.556; SEM: 0.268  30% PV (m/s)  ICC:0.764 ; SEM: 0.219  50% MV (m/s)  ICC:0.443 ; SEM: 0.157  50% PV (m/s)  ICC:0.536; SEM:0.179  60% MV (m/s)  ICC:0.353 ; SEM: 0.136  60% PV (m/s)  ICC:0.499; SEM: 0.163  70% MV (m/s)  ICC:0.446; SEM:0.103  70% PV (m/s)  ICC:0.611; SEM: 0.126  Interset-Reliability:  30% MV (m/s)  ICC:0.950; SEM: 0.049  30% PV (m/s)  ICC:0.959 ; SEM: 0.075  50% MV (m/s)  ICC:0.971; SEM: 0.025  50% PV (m/s)  ICC:0.972; SEM:0.035  60% MV (m/s)  ICC:0.965; SEM:0.019  60% PV (m/s)  ICC:0.919; SEM: 0.041  70% MV (m/s)  ICC:0.931; SEM:0.021  70% PV (m/s)  ICC:0.945; SEM: 0.030  Inter-day Reliability:  30% MV (m/s)  ICC:0.0.946; SEM: 0.046  30% PV (m/s)  ICC:0.953 ; SEM: 0.078  50% MV (m/s)  ICC:0.922; SEM: 0.035  50% PV (m/s)  ICC:0.834; SEM:0.078  60% MV (m/s)  ICC:0.547; SEM:0.060  60% PV (m/s)  ICC:0.466; SEM:0.093  70% MV (m/s)  ICC:0.549; SEM:0.043  70% PV (m/s)  ICC:0.627; SEM: 0.070 | Due to consistent differences observed between both devices, strength coaches should consistently utilize the same device for all measurements if using the HUMAC or TENDO as an autoregulatory tool for velocity-based training. The ability of the HUMAC to reliably assess MV, PV, D, and T from set to set and day to day up to 60% 1RM suggests that the device can successfully monitor changes in velocity driven by fatigue or daily fluctuations in performance. In conclusion, the HUMAC reliably measures MV, PV, D, and T from set to set and from day to day at lower intensities (30 and 50% 1RM), though was not valid based on comparisons to TENDO. |
| Gheller et al. (2023) | This study aimed to test the reliability and criterion validity of the *Ergonauta* encoder to assess countermovement jump (CMJ) performance, considering jump height and mean propulsive velocity metrics. Twenty-three recreationally active men participated in this study. The participants were positioned on a force plate with the *Ergonauta* individually connected through a belt. Two CMJs were performed, and the jump height and mean propulsive velocity metrics were analyzed. | countermovement jump | Test, retest data reliability of CMJ metrics obtained by the force plate and Ergonauta encoder:  Force plate (MPV): ICC= 0.95 (0.89-0.98), TE=2.46 (1.90–3.80)  Ergonauta (MPV): ICC= 0.99 (0.98-1.00), TE=2.14 (1.22–9.88) | In addition, the use of the *Ergonauta* encoder can represent a practical tool particularly for use outside the laboratory environment, as it is portable and does not need to be connected to an electrical current. We concluded that the *Ergonauta* encoder is reliable and valid to measure CMJ performance variables (jump height and mean propulsive velocity). Thus, the *Ergonauta* encoder can be used to assess and monitor changes in CMJ performance over time; however, caution should be employed when comparing the metrics with other measuring devices |
| Justo-Álvarez et al. (2025) | This study evaluated the concurrent validity of the Vitruve linear encoder compared to the T-Force device for measuring mean propulsive velocity (MPV) and peak velocity (PV) during the free-weight bench press exercise. Thirteen resistance-trained men (23.4 ± 1.9 years) participated in three sessions, during which MPV and PV were recorded simultaneously by both devices. | bench press |  |  |

**S16: Study characteristics part II**

| **Study** | **Procedure** | **Muscle group/ exercise** | **Results** | **clinical recommendations** |
| --- | --- | --- | --- | --- |
| Balsalobre-Fernández (2019) | Fifteen resistance trained males performed an incremental test (incremented at a rate of 5-10kg until they were unable to complete another lifting) in the bilateral and unilateral leg extension exercise up to the 1-RM in two separate occasions (48h apart).  Mean velocity during the bilateral leg extension exercise was measured for each repetition by using the validated *My Lift* v.8.1 iOS app [[12](https://pmc.ncbi.nlm.nih.gov/articles/PMC6746367/#pone.0222632.ref012)], which was installed on an iPhone 8 with the iOS 12.2 operative system (Apple Inc., USA).  The following parameters from the load-velocity relationships were computed in order to compare bilateral and unilateral tests: R2, the standard error of the estimate (SEE), the slope and the intercept with the Y-axis of the regression line, and the theoretical velocity at 40, 70 and 100% of the 1-RM. | bilateral and unilateral leg extension | Percentage of the 1-RM (%) vs. Mean Velocity (m/s)  generalized load-velocity relationship in the bilateral leg extension exercise:  r=-0.762  95% CI: -1.000, -0.674  typical load-velocity relationship in the bilateral leg extension exercise:  r=-0.991  95% CI: -1.000, -0.956  Correlation between actual and estimated 1RM:  bilateral: r= 0.96  dominant leg: r=0.96  non-dominant leg: r=0.88 | This proof of concept proved that load-velocity relationships can be measured in a machine-based, single-joint exercise by video-analyzing the vertical velocity of the weight plates using a smartphone application. These results could help strength and conditioning researchers and coaches who wish to analyze load-velocity relationship in other common machine-based exercises. |
| Balsalobre-Fernández & Kipp (2021) | Smith machine bench-press tests were performed in an eccentric–concentric (n = 192) and a concentric-only manner (n = 176) while mean concentric velocity was registered using a linear position transducer. Load–velocity profiles were derived from incremental submaximal load (40–80% 1RM) tests. Five different methods were used to calculate 1RM using the slope, intercept, and velocity at 1RM (minimum velocity threshold—MVT) from the load–velocity profiles: calculation with individual MVT, calculation with group average MVT, multilinear regression without MVT, regularized regression without MVT, and an artificial neural network without MVT | bench press | correlation between actual 1RM and estimated 1RM data eccentric-concentric and concentric-only bench-press with five different methods:  Ecc-Conc:  Ind (individual MVT): r=0.970, MAE=3.4 ± 4.4  Grp MVT (group average MVT): r=0.971, MAE=3.4 ± 4.4  OLS (ordinary least squares regression): r=0.979, MAE=2.8 ± 3.7  LASSO (least absolute shrinkage and selection operator regression): r= 0.979, MAE=2.8 ± 3.7  ANN (artificial neural network): r=0.980, MAE=2.9 ± 3.8  Conc-only:  Ind (individual MVT): r=0.987, MAE=2.9 ± 3.6  Grp MVT (group average MVT): r=0.988, MAE=3.1 ± 3.7  OLS (ordinary least squares regression): r=0.988, MAE=2.7 ± 3.5  LASSO (least absolute shrinkage and selection operator regression): r= 0.988, MAE=2.7 ± 3.5  ANN (artificial neural network): r=0.987, MAE=3.1 ± 3.8 | Results showed that multilinear regression can be used to estimate bench-press 1RM. The findings and resulting equations should be helpful for strength and conditioning coaches as they would help estimating 1RM without MVT data. |
| Banyard, Nosaka & Haff (2017) | Seventeen strength trained males performed three 1RM assessments on 3 separate days. All repetitions were performed to full depth with maximal concentric effort. Predicted 1RMs were calculated by entering the mean concentric velocity of the 1RM (V1RM) into an individualized linear regression equation, which was derived from the load–velocity relationship of 3 (20, 40, 60% of 1RM),4 (20,40,60, 80% of 1RM),or 5 (20,40,60, 80, 90% of 1RM) incremental warm-up sets. All subjects performed four 1RM assessments with each trial separated by 48 hours. Barbell displacement and mean concentric velocity were monitored by 4 fixed position transducers (Celesco PT5A 250; Chatsworth, CA, USA), which were mounted to the top of the power cage and attached to the side of the barbell. | back squat | 1RM (ICC = 0.99)  90% (ICC = 0.92)  80% (ICC = 0.87)  60% (ICC = 0.72)  Pearson correlations for the predicted 1RMs  up to 90% (r = 0.93)  80% (r = 0.87)  60% (r = 0.78) | For programming purposes, strength-trained individuals are recommended to periodize relative loads from a periodic 1RM assessment and account for daily readiness or envisaged fatigue by systematically modifying volume or intensity in accordance with period ization strategies and athlete monitoring programs |
| Benavides-Ubric et al. (2020) | Fifty men (age = 23.8 ± 3.6 years, body mass = 78.2 ± 8.3 kg, height = 1.78 ± 0.06 m) performed a first evaluation (T1) consisting of a one-repetition maximum (1RM) test. Forty-two subjects performed a second evaluation (T2) after 6 weeks. Mean (MV), mean propulsive (MPV) and peak (PV) velocity measures of the concentric phase were analyzed. Load-velocity relation ships were studied by fitting first order equations to the data using loads from 30-100% of 1RM.  The initial load was set at 20 kg and was gradually increased, initially in 20 kg increments until an MPV of 0.8 mꞏs-1 was reached, carrying out 3 repetitions with each load. Two repetitions were performed when the MPV was between 0.8 and 0.6 mꞏs-1 (10 kg increments), and only one repetition for higher loads. Increments of 5 kg were used when the MPV ranged from 0.6 to 0.5 mꞏs-1 and 2.5 kg increments were used when the MPV was less than 0.5 mꞏs 1 to 1RM. The heaviest load that each subject could properly lift while completing a full range of movement and without any external help was considered to be his 1RM. Inter-set rests were 3 min. | Deadlift | Agreement between actual and predicted 1RM from different methods:  General MV: r=0.88, ES (90%)=0.19 (0.07; 0.31)  General MPV: r=0.90, ES (90%)=0.09 (-0.01; 0.20)  General PV: r=0.91, ES (90%)=0.21 (0.11; 0.31)  Individual MV: r=0.97, ES (90%)=0.14 (0.07; 0.20)  Individual MPV: r=0.97, ES (90%)=0.04 (-0.02; 0.10)  Individual PV: r=0.98, ES (90%)=0.19 (0.13; 0.24)  The repeatability analysis resulted in following stability values:  MV: ICC: 0.870 (95%CI = 0.758 - 0.930)  MPV: ICC: 0.865 (95%CI = 0.748 - 0.927)  PV: ICC: 0.828 (95%CI = 0.681 - 0.908)  Velocity at 1RM showed the following values:  MV & MPV: ICC: 0.763 (95%CI = 0.565 - 0.871)  PV: ICC: 0.718 (95%CI = 0.483 - 0.847) | This study suggests that by monitoring repetition velocity during the DL exercise, it is possible to determine whether the proposed load (kg) for a given training session actually represents the effort (%1RM) that was intended. Individual load-velocity relationships should be recommended to prescribe the RT intensity on an individual basis with appropriate accuracy |
| Bazuelo-Ruiz et al. (2015) | One hundred and five physically active young subjects (87 men and 18 women) performed a submaximal and a maximal load test during half-squat exercises on a Smith machine. In the submaximal test, subjects completed 3 repetitions with a load equivalent to body weight. The velocity and power of barbell displacement were recorded during the upward movement from 908 of knee flexion. All repetitions were performed at maximum velocity. In a subsequent 1–2RM test, the 1RM for the exercise was calculated. The variables’ load and mean velocity (Vmean) were used to construct an adjusted 1RM prediction model, which was capable of estimating the 1RM with an accuracy of 58%. | squat | comparison between the actual vs. estimated RM:  ICC = 0.793; 95% confidence interval [CI]: 0.32–0.93, p<0.01 | According to this model, for the same BW, a 0.1 m*s-1 improvement in the barbell displacement velocity would indicate an increase of 9–15 kg in 1RM. Thus, 1RM or a change in 1RM can be predicted with an accuracy of 58% in young physically active adults using the prediction equation. From a practical point of view, a mixed sample model simplifies the estimation process, provides a quick and safe method to estimate 1RM values in untrained subjects starting a resistance training program, and serves as guidance in determining power-load profiles |
| Berton et al. (2021) | For each exercise, 12 participants performed two 1RM sessions tests and two sessions to measure the barbell’s load-velocity relationship at 30, 45, 60, 75, and 90% of 1RM. The velocity recorded at each load was used to establish the linear regression equation and, consequently, to predict 1RM value. Participants visited the laboratory on 12 occasions; four sessions per exercise. For a given exercise, the first two sessions were dedicated to the 1RM direct tests, while the test to predict the 1RM based on the load-velocity relationship was carried out in the last two sessions. A battery of five loads (30, 45, 60, 75 and 90% of 1RM) was employed through the load-velocity continuum to establish a linear regression equation to predict the 100% of 1RM. Consistent intervals of 72 or 96 hours for each participant were granted between the sessions.  Video recordings of all the repetitions were obtained via an iPhone 5s camera (Apple Inc., USA) and were filmed with 1,280 x 720 pixel resolution at 120 frames per second (FPS) (Garhammer & Newton, 2013). Throughout all video recordings, the iPhone’s camera was placed on a tripod 1.30 m above the ground, in the sagittal plane (participants' left side) at 5 m from the area in which the exercises were performed.  Barbell trajectory auto-tracking analyses were carried out by the Kinovea software (Experimental Version 0.8.25-x64) (Daehlin, Krosshaug, & Chiu, 2017; Garhammer & Newton, 2013). | power clean from the knee (PCK), high pull from the knee (HPK) and mid-thigh clean pull (MTCP) | Validity between the best 1RM direct test vs. predicted 1RM – session 1, and best 1RM direct test vs. predicted 1RM  PCK:  Best 1RM direct test vs. predicted 1RM – Session 1:ES= -0.76 (-1.27, -0.31)  Best 1RM direct test vs. predicted 1RM – Session 2: ES=-0.58 (-1.06, -0.15)  HPK:  Best 1RM direct test vs. predicted 1RM – Session 1:ES= -0.40 (-0.53, 0.35)  Best 1RM direct test vs. predicted 1RM – Session 2: ES=-0.39 (-0.53, 0.34)  MTCP:  Best 1RM direct test vs. predicted 1RM – Session 1:ES= -0.29 (-0.53, 0.39)  Best 1RM direct test vs. predicted 1RM – Session 2: ES=-0.19 (-0.51, 0.41)  Reliability between 1RM direct test and predicted 1RM based on the load-velocity relationship:  PCK: 1RM direct test: ICC= 0.97 (0.89 - 0.99), ES=-0.02(-0.44 - 0.39)  predicted 1RM: ICC= 0.97 (0.83 - 0.99), ES=0.13(-0.29 - 0.55)  HPK: 1RM direct test: ICC= 0.91 (0.64 - 0.98), ES=0.17(-0.27 - 0.61)  predicted 1RM: ICC= 0.73 (0.25 - 0.93), ES=-0.06(-0.51 - 0.37)  MTCP: 1RM direct test: ICC= 0.98 (0.95 - 0.99), ES=0.04(-0.40 - 0.48)  predicted 1RM: ICC= 0.95 (0.73 - 0.99), ES=0.12(-0.34 - 0.59) | The load-velocity relationship was not able to predict 1RM values with high accuracy in the PCK, HPK, and MTCP. The 1RM direct test was the most reliable for PCK, HPK and MTCP. |
| Bosquet et al. (2010) | Twenty seven physical education students and teachers (5 women and 22 men) with a heterogeneous history of strength training participated in this study. They performed a 1 RM test and a force - velocity test using a Bench press lifting task in a random order. Mean 1 RM was 61.8 ± 15.3 kg (range: 34 to 100 kg), while 1 RM estimated by the Musclelab’s software from the force-velocity relationship was 56.4 ± 14.0 kg (range: 33 to 91 kg). Load incrementation was then adjusted to this subjective assessment (RPE). Three minutes of passive recovery were given between each attempt, and load was increased until task failure. | bench press | Actual and estimated 1RM were very highly correlated r = 0.93, p=<0.001, ES= 1.37  Relative reliability, which represents the degree to which individuals maintain their position in a sample with repeated day-to-day measurements: r = 0.97 | Athletes and coaches should therefore consider this measure as a good one for monitoring training induced adaptations, since any increase in estimated 1 RM should reflect a real improvement of this component of muscle strength, but not for prescribing training intensities, the error of prediction being too large to determine precisely training loads. |
| Caven et al. (2020) | Seventeen trained females (age 17.8 ± 1.3 years) performed an incremental loading test to 1RM on bench press and squat with the mean velocity being recorded. The 1RM was estimated from the load–velocity relationship using the multiple- (8 loads) and two-point (2 loads) methods and group and individual minimum velocity thresholds (MVT). Participants attended the strength and conditioning gym on two occasions (one per exercise with 48 h between) during the competitive season.  The data of eight loads (~40, 45, 55, 60, 70, 80, 85 and 90% 1RM for bench press and ~20, 30, 45, 55, 65, 75, 85 and 90% 1RM for squat; i.e., the multiple-point method) and only the two distant loads (~40 and 90% 1RM for bench press and 20 and 90% 1RM for squat; i.e., the two-point method) were used for the modelling of the individual load–velocity relationships by applying linear regression models.  GymAware was employed to measure mean velocity from time and displacement. | bench press & squat | MVT Bench press:  Group (Multiple-point): r=0.94, ES= 0.10  Group (Two-point): r=0.84, ES=0.32  Individualised (Multiple-point): r=0.97, ES=0.11  Individualised (Two-point): r=0.89, ES=0.33  MVT squat:  Group (Multiple-point): r=0.86, ES=0.19  Group (Two-point): r=0.76, ES=0.29  Individualised (Multiple-point): r=0.95, ES=0.16  Individualised (Two-point): r=0.93, ES=0.23 | From the practical point of view, it is more time-efficient and less fatiguing to use the two-point method. Readers should also be aware that low-cost phone applications (e.g., iLOAD, PowerLift), which can accurately measure velocity, are available to use if the most expensive equipment used in our study is not available. |
| Çetin et al. (2022) | Thirteen resistance-trained men performed six sessions that consisted of two incremental loading tests (multi-point method: 20-40-60-80-90% and two-point method variations: 40-60%, 40-80%, 40-90%,60-80%, 60-90%) followed by 1RM tests. Squat assessments were done on the first, the second, and the third day, whereas deadlift assessments were done on the fourth, the fifth, and the sixth day. On the first day, and the fourth day, familiarization sessions were conducted for each exercise.The testing sessions were separated by 48 h each other. Barbell velocity data was collected by means of a linear position transducer GymAware. | deadlift & back squat | Inter-session reliability from the load-velocity profile in the deadlift:  Deadlift multiple method: ICC=0.996  Deadlift 40-60%: ICC= 0.171  Deadlift 40-80%: ICC= 0.815  Deadlift 40-90%: ICC= 0.996  Deadlift 60-80%: ICC= 0.335  Deadlift 60-90%: ICC= 0.972  Inter-session reliability from the load-velocity profile in the squat:  Squat multiple method: ICC=0.817  Squat 40-60%: ICC= 0.235  Squat 40-80%: ICC= 0.822  Squat 40-90%: ICC= 0.905  Squat 60-80%: ICC= 0.479  Squat 60-90%: ICC= 0.988  Relationship of the load velocity profile in deadlift exercise between methods:  Deadlift multiple method vs. Deadlift Real RM: r= 0.929  Deadlift 40-60% vs. Deadlift Real RM: r= 0.851  Deadlift 40-80% vs. Deadlift Real RM: r= 0.903  Deadlift 40-90% vs. Deadlift Real RM: r= 0.972  Deadlift 60-80% vs. Deadlift Real RM: r= 0.856  Deadlift 60-90% vs. Deadlift Real RM: r= 0.956  Squat multiple method vs. Squat Real RM: r= 0.879  Squat 40-60% vs. Squat Real RM: r= 0.030  Squat 40-80% vs. Squat Real RM: r= 0.610  Squat 40-90% vs. Squat Real RM: r= 0.839  Squat 60-80% vs. Squat Real RM: r= 0.062  Squat 60-90% vs. Squat Real RM: r= 0.872 | They recommend using the multi-point method and especially two-point methods variations including higher relative loads in estimating 1RM. |
| da Silva et al. (2023) | Initially, 12 healthy young males, with no PC experience, underwent eight sessions to learn the PC technique. After the learning period, the participants visited the laboratory on four more occasions with intervals between the visits from 72 to 96 h. The first two sessions were dedicated to the actual 1RM tests, while the last two sessions were performed to measure the barbell’s load-velocity relationship at 30%, 45%, 60%, 75%, and 90% of PC 1RM. The highest peak velocity recorded at each load was used to establish the linear regression equation and, consequently, to predict 1RM values. The video recordings of all PC repetitions were obtained via an iPhone 5S camera (Apple Inc., USA) and were filmed with 1,280 x 720-pixel resolution at 120 fps (Garhammer & Newton, 2013).  Barbell trajectory auto-tracking analysis was carried out using Kinovea software (Experimental Version 0.8.25-x64) (Daehlin, Krosshaug, & Chiu, 2017; Marriner, Cronin, Macadam, & Storey, 2017). | power clean from the knee (PC) | Validity analyses between the highest actual 1RM test value vs. predicted 1RM—sessions 1 and 2.  Highest 1RM test vs. predicted 1RM—Session 1:ES=-1.00 (-1.56, -0.52)  Highest 1RM test vs. predicted 1RM—Session 2:ES=-1.23 (-1.84, -0.71)  Reliability analyses between 1RM tests and predicted 1RM based on the load-velocity relationship:  1RM test: ICC= 0.94 (0.81-0.98), ES= 0.38 (-0.04 - 0.83)  Predicted 1RM: ICC= 0.69 (0.19-0.87), ES= -0.34 (-0.78 - 0.08) | From a practical standpoint, few strength and conditioning coaches have access to this type of equipment. Thus, the data collected by a smartphone and the use of free software may favor implementation and usability. Second, only two prediction 1RM sessions were performed, further improving applicability. Specifically, including more sessions could induce a tech nical improvement in the PC and, consequently, provide a better predicted 1RM value. However, due to high time demand, several 1RM testing sessions may be impractical in training programs.These results collectively support the use of the 1RM test instead of the load-velocity relationship to measure the 1RM value in the PC exercise. |
| dos Santos et al. (2023) | Twenty-two National Collegiate Athletic Association Division I male ice hockey players (age = 21.0 ± 1.5 years, height = 182.9 ± 7.3 cm, and body mass = 86.2 ± 7.3 kg) completed a progressive loading test using the HBD at maximum intended velocity to determine their 1RM. The mean concentric velocity was measured for each load through a linear position transducer (GymAware). Each subject refrained from strenuous activity for 24 hours before the testing.  After a standardized dynamic warm-up protocol, initial load was set at 65% and increased to 75, 80, 90, and 95% of estimated 1RM. Three repetitions were performed for intensities ≤80% 1RM, 2 for 90% 1RM, and 1 for 95% 1RM. After that, increments of 2–5 kg were made until the subjects were unable to complete a single lift throughout the entire range of motion with proper form (i.e., technical failure). A rest interval of 3 minutes after each set was provided. | Hexagonal Barbell Deadlift | R(95%)= 0.93 (0.84-0.97), p= 0.90, ES (95% CI)= -0.08 (-0.16 to 0.14) | With HBD velocity measurements at hand, coaches may have real-time performance feedback allowing them to determine exercise intensity and monitor neuromuscular fa tigue. These data can also assist coaches when implementing velocity-based training as part of the training program. |
| Fernandes et al. (2021) | Two days after maximal strength testing, 20 young (age 21.0 ± 1.6 years) and 20 middle-aged (age 42.6 ± 6.7 years) resistance-trained males completed three repetitions of bench press, back squat, and bent-over-row at loads corresponding to 20–80% 1RM. Using reference minimum velocity threshold (MVT) values, the 1RM was estimated from the load-velocity relationships through multiple (20, 30, 40, 50, 60, 70, and 80% 1RM), two-point (20 and 80% 1RM), high-load (60 and 80% 1RM) and low-load (20 and 40% 1RM) methods for each group.  Session 1:Bench press and bent-over-row maximal strength were directly assessed using a standard 1RM protocol. The 1RM back squat was predicted from a 5RM protocol and equation detailed previously.  Session 2: Mean velocity was assessed during the three exercises at loads corresponding to 20, 30, 40, 50, 60, 70, and 80% 1RM. Loads were applied randomly, and the FitroDyne rotary encoder (Fitronic, Bratislava, Slovakia) was attached directly under and to the most lateral aspect of the Smith machine bar.Three repetitions of each exercise were performed at each load with self-selected rest intervals that were capped at 90 s, but ranged from 30 to 90 s. Rest times were self-selected as lighter loads did not require the same recovery time. | bench press, back squat & bent-over-row | bench press:  whole:  multiple-point: r= 0.85, p= 0.815, ES=−0.02  Distant two-point: r= 0.87, p= 0.531, ES=0.05  Low-load: r= 0.58, p= 0.220, ES=0.21  High-load: r= 0.87, p= 0.324, ES=0.08  young:  multiple-point: r= 0.79, p=0.438, ES=−0.12  Distant two-point: r= 0.80, p=0.743, ES=−0.05  Low-load: r= 0.63, p=0.870, ES=−0.03  High-load: r= 0.81, p=0.902, ES=−0.02  Middle-aged:  multiple-point: r= 0.86, p=0.504, ES=0.08  Distant two-point: r= 0.90, p=0.098, ES=0.17  Low-load: r=0.61, p=0.069, ES=0.56  High-load: r= 0.90, p=0.056, ES=0.20  back squat:  whole:  multiple-point: r= 0.92, p=0.570, ES=−0.04  Distant two-point: r= 0.91, p=0.463, ES=−0.05  Low-load: r= 0.74, p=0.814, ES=−0.03  High-load: r= 0.93, p=0.071, ES=−0.13  young:  multiple-point: r= 0.95, p=0.689, ES=0.04  Distant two-point: r= 0.96, p=0.965, ES=0.00  Low-load: r= 0.71, p=0.927, ES=−0.02  High-load: r= 0.95, p=0.755, ES=−0.03  Middle-aged:  multiple-point: r= 0.78, p=0.306, ES=−0.16  Distant two-point: r= 0.73, p=0.382, ES=−0.16  Low-load: r=0.62, p=0.802, ES=−0.07  High-load: r= 0.81, p=0.037 *, ES=−0.32  bent-over-row:  whole:  multiple-point: r= 0.77, p=0.011 * , ES=0.34  Distant two-point: r= 0.74, p<0.001 *, ES=0.56  Low-load: r= 0.72, p=0.403, ES=−0.14  High-load: r= 0.77, p=0.002 *, ES=0.50  young:  multiple-point: r= 0.71, p=0.046 *, ES=0.43  Distant two-point: r= 0.71, p=0.014 *, ES=0.54  Low-load: r= 0.68, p=0.964, ES=0.01  High-load: r= 0.72, p=0.023 *, ES=0.52  Middle-aged:  multiple-point: r= 0.78, p=0.110, ES=0.29  Distant two-point: r= 0.70, p=0.004 *, ES=0.69  Low-load: r=0.55, p=0.080, ES=−0.56  High-load: r= 0.81, p=0.035 *, ES=0.50 | They do not recommend using reference MVT values to predict 1RM in these populations. Instead, if practitioners want to implement a 1RM mean velocity prediction value, it should be specific to their population, training situation, and device. |
| Fitas et al. (2024) | Individual load-velocity profiles (LVP) were obtained in 18 athletes enrolled in recreational soccer. Reliability analyses were computed for all LVP-derived variables. Estimations of 1RM were made based on general (0.3 m.s− 1), pre-individual (mean velocity at 1RM obtained in a preliminary session) and optimal MVT (velocity that eliminates the difference between actual and predicted 1RM, determined in a preliminary session). The accuracy of 1RM predictions was examined using absolute-percent error and Bland-Altman plots. | back squat | Between-day reliability of 1RM, slope, load at zero velocity (LD0), individual and optimal minimum velocity threshold (MVT)  1RM: ICC= 0.99 [0.990–0.999], p= 0.564  relative 1RM:ICC=0.99 [0.975–997], p= 0.563  slope:ICC= 0.96 [0.900–0.986], p= 0.917  LD0:ICC= 0.99 [0.975–0.996], p= 0.594  Individual MVT:ICC= 0.93 [0.810–0.973], p= 0.798  optimal MVT: ICC= 0.92 [0.788–0.970], p= 0.737  Validation of 1RM predictions based on the general, pre-individual and optimal minimal velocity threshold (MVT):  General MVT: r= 0.396  Pre-individual MVT: r= 0.743*  Optimal MVT: r= 0.437 | This suggests that the optimal MVT can be used to estimate an ath lete’s readiness state and maximal neuromuscular capacities during, at least, a reduced period (i. e. microcycle).It is recommended that practitioners evaluate their optimal MVT from time to-time because its long-term stability remains largely unknown. |
| Gantois et al. (2022) | Participants (n=27) performed a progressive load test up to the one-repetition maximum (1RM) load for determining the individual load–velocity relationship in the hexagonal bar deadlift exercise. Bar velocity was measured in every repetition through a linear encoder. In The Progressive Loading Test,a hexagonal bar(barmass=13kg) was coupled with a linear position transducer (Speed4lifts).The cable was attached to the right side the bar using a velcro strap. The initial external load of the test was 33 kg, which was increased by 10 kg until the bar velocity reached 0.80 m/s, then increments of 10–5 kg until reaching a bar velocity of 0.5 m/s, and then increments of 5–1 kg were performed until the 1RM was determined. For light loads (>0.80 m/s), three repetitions were performed, two for moderate (0.80–0.50 m/s) and one for heavy (<0.50 m/s) loads. The interset rest period was 2 min for the light, 3 min for moderate, and 4 min for the heavy loads. Only the fastest repetition recorded by the mean propulsive velocity (MPV) at each load was retained for the subsequent analysis | hexagonal bar deadlift | actual vs. predicted 1RM for general load-velocity relationship: ICC= 0.97; CI= 0.90, 0.99  actual vs. predicted 1RM for individual load-velocity relationship: ICC= 0.98; CI= 0.91, 0.99 | These findings suggest that strength and conditioning coaches can estimate the 1RM from the MPV recorded during a single repetition performed at maximum effort in the HBD exercise, providing a quick and safe approach to determine the % 1RM in this exercise in resistance trained women. Strength and conditioning coaches and sports scientists may use these reference values for prescribing HBD exercise load |
| Garcia-Ramos et al. (2019) | Twenty-six men (22 rowers and four weightlifters) performed an incremental loading test until reaching their 1RM, followed by a set of repetitions-to-failure. Eighteen participants were re-tested to conduct the reliability analysis. The 1RM was estimated through the lifts-to-failure equations proposed by Lombardi and O’Connor, general load velocity (L-V) relationships proposed by Sánchez-Medina and Loturco and the individual L-V relationships modelled using four (multiple-point method) or only two loads (two-point method).  Participants were asked to attend two testing sessions separated by 72–96 hours. The first testing session was used for validity analyses, while 18 participants returned for reliability analyses. Both testing sessions were identical and consisted of an incremental loading test until reaching the external load that could be completed for one repetition (1RM). Following 10 minutes of recovery, a single set to failure with a load that was 75–90% of 1RM was completed  The external load was increased in 10 kg until the mean velocity (MV) of the barbell was lower than 0.80 ms−1 (≈ 70% 1RM) and afterwards from 5 to 1 kg until the 1RM load was reached. Three repetitions were performed with light loads (MV >1.10m·s−1), two with medium loads (1.10 m·s−1 ≤ MV ≤ 0.80 m·s−1) and one with heavy loads (MV < 0.80 m·s−1). Intra set rest between repetitions was 10 seconds and inter-set rest was 5 minutes. The closest load of the incremental loading test to a MV of 0.70 m·s−1 (L4) was used to estimate the 1RM through the general L-V relationships | bench pull | Reliability of the 1RM obtained from different methods during bench pull:  maximal single lift (direct): ICC = 0.97(0.93,0.99)  Lifts-to-failure (Libardi): ICC= 0.94(0.86,0.98)  Lifts-to-failure (O’Connor et al.): 0.86(0.66,0.94)  General L-V relationship (Sánchez-Medina et al.): ICC= 0.91(0.77,0.96)  General L-V relationship (Loturco et al.): ICC= 0.91(0.77,0.96)  Individual L-V relationship (Multiple-point): ICC= 0.90(0.75,0.96)  Individual L-V relationship (Two-point): ICC= 0.81(0.56,0.93)  Association between the actual 1RM and the predicted 1RM:  Lifts-to-failure (Libardi): r = 0.966 (0.930,0.986)  Lifts-to-failure (O’Connor et al.): r= 0.927 (0.880,0.960  General L-V relationship (Sánchez-Medina et al.): r= 0.933 (0.858, 0.971)  General L-V relationship (Loturco et al.): r= 0.933 (0.861, 0,971)  Individual L-V relationship (Multiple-point): r= 0.926 (0.832,0.974)  Individual L-V relationship (Two-point): r= 0.926 (0.845,0,967) | They specifically recommend the individual L-V relationship over the lifts-to-failure and general L-V relationship equations. The determination of the individual L-V relationship is less time consuming and less prone to fatigue than the repetitions-to-failure procedure (especially if determined by the two-point method), while the accuracy of the general L-V relationship could be affected by slight variations of the testing procedure such as the use of a Smith machine or a different velocity measurement device. |
| García-Ramos et al. (2018) | Thirty men (BP 1RM relative to body mass: 1.08 [0.18] kg·kg−1) performed 2 incremental loading tests in the concentric-only BP exercise and another 2 in the eccentric–concentric BP exercise to assess their actual 1RM and load–velocity relationships.  A high velocity (≈1 m·s−1) and a low velocity (≈0.5 m·s−1) were selected from their load–velocity relationships to estimate the 1RM from generalized group equations and through an individual linear model obtained from the 2 velocities. The initial external load of the incremental loading test was set at 17 kg and was progressively increased in 10 kg increments until the attained MPV was lower than 0.50 m·s-1. Thereafter, the load was progressively increased in steps of 5 to 1 kg until the actual 1RM was directly determined with the completion of a single maximal lift. Three attempts were executed at each lighter load (MPV > 1.00 m·s−1), two for the medium (0.65 m·s−1 ≤ MPV ≤ 1.00 m·s−1), and only one for the heavier loads (MPV < 0.65 m·s−1). The average number of sets performed with the heavier loads was 4.2 1.1 for the concentric-only BP and 3.6 0.9 for the eccentric-concentric BP. Intra-set rest was 10 s and inter-set rest was fixed to 5 min.  A Smith machine (Technogym, Barcelona, Spain) coupled with a linear velocity transducer (T-Force System; Ergotech, Murcia, Spain) which sampled the velocity of the barbell at a frequency of 1,000 Hz was used for all testing sessions. The MV and MPV of all repetitions were recorded. The start of the concentric phase for both BP variants was defined as the onset of positive velocity. The acceleration of the barbell was obtained as a time derivate of the velocity data. MV was calculated as the average velocity from the start of the concentric phase (i.e., onset of positive velocity) until the barbell reaches maximum height (i.e, zero velocity). MPV was calculated as the average velocity from the start of the concentric phase until the acceleration of the barbell dropped below the gravity (-9.81 ms-2).  Two different procedures were used to estimate the 1RM from barbell velocity: Generalized group equations & Two-point method. | bench press | Validity of the predicted 1RMs:  correlation obtained for the high velocities: r = 0.847-0.876  correlation obtained for the low velocities: r = 0.960-0.968  correlation obtained for the two-point method: r= 0.956-0.977  load-velocity relationships of a representative subject in the concentric-only BP and eccentric-concentric BP + mean velocity and mean propulsive velocity:  MV in concentric-only: r =0.996  MV in eccentric-concentric BP: r=0.995  MPV in concentric-only: r=0.997  MPV in eccentric-concentric: r=0.988  the absolute differences between the actual 1RM and predicted 1RM:  actual 1RM :  1.0 MV (concentric-only BP): r= 0.860, ES=-0.81  0.5 MV (concentric-only BP): r= 0.966, ES=-0.41  Two-Point MV (concentric-only BP): r= 0.976, ES=-0.17  1.0 MPV (eccentric-concentric BP): r= 0.876, ES=-0.98  0.5 MPV (eccentric-concentric BP): r= 0.968, ES=-0.36  Two-point MPV (eccentric-concentric BP): r= 0.977, ES=-0.03  predicted 1RM (kg):  1.0 MV (concentric-only BP): r= 0.847, ES=0.94  0.5 MV (concentric-only BP): r= 0.961, ES=0.15  Two-Point MV (concentric-only BP): r= 0.957, ES=0.02  1.0 MPV (eccentric-concentric BP): r= 0.872, ES=0.88  0.5 MPV (eccentric-concentric BP): r= 0.960, ES=0.15  Two-point MPV (eccentric-concentric BP): r= 0.956, ES=0.04  reliability of the 1RM obtained from different methods:  direct method: ICC= 0.98  comparison of the reliability of the 1RM value between different methods for each bench press variant:  concentric-only:  1.0 MV: ICC= 0.94 (0.87, 0.97), p=0.666, ES=0.03  0.5 MV: ICC= 0.94 (0.88, 0.97), p=0.490, ES=-0.05  1.0 MPV: ICC=0.92 (0.84, 0.96), p=0.501, ES=-0.05  0.5 MPV: ICC= 0.95 (0.89, 0.98), p=0.308, ES=-0.07  Two-Point MV: ICC= 0.92 (0.84, 0.96), p=0.373, ES=-0.07  Two-point MPV: ICC= 0.90 (0.79, 0.95), p=0.735, ES=-0.03  direct: ICC= 0.99 (0.97, 0.99), p=0.538, ES=-0.02  eccentric-concentric:  1.0 MV: ICC= 0.93 (0.86, 0.97), p=0.229, ES=0.09  0.5 MV: ICC= 0.96 (0.91, 0.98), p=0.790, ES=-0.02  1.0 MPV: ICC=0.89 (0.78, 0.95), p=0.975, ES=0.0  0.5 MPV: ICC= 0.96 (0.91, 0.98), p=0.599, ES=-0.03  Two-Point MV: ICC=0.95 (0.89, 0.97), p=0.529, ES=-0.05  Two-point MPV: ICC= 0.95 (0.89, 0.98), p=0.215, ES=-0.08  direct: ICC= 0.98 (0.96, 0.99), p=0.396, ES=0.03 | The 1RM of the concentric-only and eccentric-concentric BP exercises can be estimated with high reliability and validity through the two-point method. The application on a daily basis of the two-point method would require: (1) set the velocity of the 1RM for a particular subject with the device used in training to monitor velocity (this step could be avoided when the velocity of the 1RM is known), (2) record the mean velocity against two different external loads representing approximately 50% and 80% of self-reported 1RM, and (3) model the individual load-velocity relationship and determine the 1RM as the load associated with the velocity of the 1RM previously established. |
| Hughes et al. (2019) | Twenty strength-trained men (age: 24.3 ± 2.9 years, height: 180.1 ± 5.9 cm, and body mass: 84.2 ± 10.5 kg) were recruited and visited the laboratory on 3 occasions. The load-velocity relationship was developed using the mean concentric velocity of repetitions performed at loads between 20 and 90% 1RM. Predicted 1RM was calculated using 3 different methods discussed in existing research: minimal velocity threshold 1RM (1RMMVT), load at zero velocity 1RM (1RMLD0), and force-velocity 1RM methods (1RMFV). The reliability of 1RM predictions was examined using intraclass correlation coefficient (ICC) and coefficient of variation (CV).  During each lift, the displacement of the bar and time between data points were recorded using a cable linear position transducer sampling at up to 50 Hz (GymAware Powertool).  The 1RMFV was calculated using a custom Matlab (Math-Works; Natick, MA, USA) script with all calculations based on the supplementary material provided by Picerno et al.. | squat | Change in ICC of variation for test-retest assessments of the velocity of different loads:  Mean velocity 20% 1RM: ICC (CI)= 0.38 (-0.17 to 0.68)  Mean velocity 40% 1RM: ICC (CI)= 0.74 (0.39 to 0.87)  Mean velocity 60% 1RM: ICC (CI)= 0.62 (0.15 to 0.81)  Mean velocity 80% 1RM: ICC (CI)= 0.54 (0.00 to 0.77)  Mean velocity 90% 1RM: ICC (CI)= 0.25 (-0.29 to 0.63)  Mean velocity 100% 1RM: ICC (CI)= 0.45 (0.32 to 0.56)  Change in ICC of variation for test and rest assessment of 1RM predicted from the load-velocity relationship:  1RM_MVT_:  20–90% (kg): ICC=0.92 (0.82 to 0.97)  20–80% (kg): ICC= 0.92 (0.84 to 0.97)  40–90% (kg): ICC=0.96 (0.91 to 0.98)  1RM_LD0_  20–90% (kg): ICC= 0.82 (0.63 to 0.90)  20–80% (kg): ICC= 0.78 (0.57 to 0.9)  40–90% (kg): ICC=0.81 (0.62 to 0.91)  1RM_FV_  20–90% (kg): ICC= 0.00 (-0.39 to 0.39)  20–80% (kg): ICC= -0.28 (-0.6 to 0.12)  40–90% (kg): ICC= -0.11 (-0.48 to 0.29)  Difference in measured 1RM and the 1RM MVT calculated using different loads against the mean 1RM values across both methods:  20-90% 1RM:  Trial 1: r= -0.421, p= 0.082  Trial 2: r= -0.439, p= 0.068  20-80% 1RM  Trial 1: r= -0.536, p= 0.022  Trial 2: r= -0.543, p= 0.020  40-90% 1RM  Trial 1: r= -0.316, p= 0.201  Trial 2: r= -0.337, p= 0.171  Difference in measured 1RM and the 1RM_LD0_ calculated using different loads against the mean 1RM values across both methods:  20-90% 1RM:  Trial 1: r= -0.257, p= 0.303  Trial 2: r= -0.276, p= 0.279  20-80% 1RM  Trial 1: r= -0.364, p= 0.138  Trial 2: r= -0.214, p= 0.394  40-90% 1RM  Trial 1: r= -0.216, p= 0.388  Trial 2: r= -0.357, p= 0.146  Difference in measured 1RM and the 1RM_FV_ calculated using different loads against the mean 1RM values across both methods:  20-90% 1RM:  Trial 1: r= -0.797, p<0.001  Trial 2: r= -0.926, p<0.001  20-80% 1RM  Trial 1: r= -0.399, p= 0.101  Trial 2: r= -0.843, p<0.001  40-90% 1RM  Trial 1: r= -0.955, p<0.001  Trial 2: r= -0.872, p<0.001 | This indicates that load-velocity profiles constructed with >4 incremental weights between 40 and 80% 1RM may be most accurate. The 1RMMVT models provide the most reliable methods currently available to predict 1RM from load-velocity profiles for free-weight squat exercise. The results emphasize that caution should be used when predicting 1RM values from load-velocity profiles, particularly if it is the practitioner’s intention to use these values to adjust training load. |
| Janicijevic et al. (2021) | A total of 86 men performed 2 identical sessions (incremental loading test until reaching the 1RM followed by a set to failure) in a randomized order during the paused and touch-and-go bench press exercises. Individualized load-velocity relationships were modeled by linear and polynomial regression models considering 4 loads (45%-60%-75%-90% of 1RM) (multiple-point methods) and considering only 2 loads (45%-90% of 1RM) by a linear regression (2-point method). Three minimal velocity thresholds were used: the general velocity of 0.17 m·s-1 (general velocity of the 1RM [V1RM]), the velocity obtained when lifting the 1RM load (individual V1RM), and the velocity obtained during the last repetition of a set to failure. The initial load was set to 20 kg, and it was increased in 10 kg increments when the MCV of the barbell was >0.50 m·s−1 and in 1 to 5 kg increments when the MCV was lower than 0.50 m·s−1 until the 1RM load was achieved. Two repetitions were performed when the MCV was higher than 0.50 m·s−1, and one repetition when the MCV was lower than 0.50 m·s−1. The rest between sets ranged from 3 to 5 minutes. A set of repetitions to failure with approximately 85% 1RM load was performed 10 minutes after the 1RM assessment.  A validated linear velocity transducer (T-Force System; Ergotech, Murcia, Spain) was attached to the bar of the Smith machine and sampled the velocity–time data at a frequency of 1000 Hz.21 The data of 4 loads (approximately 45%, 60%, 75%, and 90% of 1RM; ie, multiple-point methods) or only 2 loads (approximately 45% and 90% of 1RM; ie, 2-point method) were used for the modeling of the individualized L–V relationships. Three minimal velocity thresholds were used to estimate the 1RM for each regression model: a general V1RM of 0.17 m·s−1, the individual V1RM obtained when lifting the 1RM load, and the velocity obtained during the last repetition of a set to failure (Vlast). | bench press | Relationship between actual 1RM and the 1RM estimated by the nine 1RM prediction methods during the paused BP:  Multiple-point linear; General V_1RM_: r= 0.99, SEE= 2,8 kg, p<0.001, ES=0.09  Multiple-point linear; Individual V_1RM_: r= 0.98, SEE= 3,1 kg, p<0.001, ES=0.09  Multiple-point linear; V_1RM_: r= 0.99, SEE= 2, kg, p<0.001, ES=0.12  Two-point linear; General V_1RM_: r= 0.99, SEE= 2,9 kg, p<0.001, ES=0.08  Two-point linear; Individual V_1RM_: r= 0.99, SEE= 3,0 kg, p<0.001, ES=0.08  Two-point linear; V_1RM_: r= 0.99, SEE= 3,0 kg, p<0.001, ES=0.12  Multiple-point polynomial; General V_1RM_: r= 0.96, SEE= 4,7 kg, p=0.041, ES=0.06  Multiple-point polynomial; Individual V_1RM_: r= 0.97, SEE= 4,6 kg, p=0.059, ES=0.096  Multiple-point polynomial; V_1RM_: r= 0.96, SEE= 4,9 kg, p=0.003, ES=0.10  Relationship between the 1RM and the 1RM estimated by the nine prediction methods during the touch-and-go BP exercise:  Multiple-point linear; General V_1RM_: r= 0.98, SEE= 3,4 kg. p<0.001, ES=0.15  Multiple-point linear; Individual V_1RM_: r= 0.98, SEE= 3,2 kg, p<0.001, ES=0.15  Multiple-point linear; V_1RM_: r= 0.98, SEE= 3,6 kg, p<0.001, ES=0.17  Two-point linear; General V_1RM_: r= 0.98, SEE= 3,5 kg, p<0.001, ES=0.14  Two-point linear; Individual V_1RM_: r= 0.98, SEE= 3,3 kg, p<0.001, ES=0.14  Two-point linear; V_1RM_: r= 0.98, SEE= 3,7 kg, p<0.001, ES=0.16  Multiple-point polynomial; General V_1RM_: r= 0.97, SEE= 4,3 kg, p=0.009, ES=0.07  Multiple-point polynomial; Individual V_1RM_: r= 0.98, SEE= 4,0 kg, p=0.009, ES=0.06  Multiple-point polynomial; V_1RM_: r= 0.97, SEE= 4,4 kg, p=0.002, ES=0.08  Relationship of the raw errors in the prediction of the 1RM between the paused and touch-and-go BP exercises for the nine 1RM prediction:  Multiple-point linear; General V_1RM_: r= 0.243, p=0.024  Multiple-point linear; Individual V_1RM_: r= 0.292, p=0.006  Multiple-point linear; V_1RM_: r= 0.182, p=0.093  Two-point linear; General V_1RM_: r= 0.173, p=0.111  Two-point linear; Individual V_1RM_: r= 0.252, p=0.019  Two-point linear; V_1RM_: r= 0.103, p=0.345  Multiple-point polynomial; General V_1RM_: r= -0.014, p=0.897  Multiple-point polynomial; Individual V_1RM_: r= -0.024, p=0.826  Multiple-point polynomial; V_1RM_: r= 0.012, p=0.911 | Practitioners are encouraged to use linear regression models and a general minimal velocity threshold of 0.17 m·s−1 with relatively novice individuals if they are willing to accept approximately 4.5% error in the estimation of the 1RM. |
| Janicijevic, et al. (2024) | The 1RMs of 27 resistance-trained males were predicted using three types of MVT: (i) General MVT: averaged across subjects’ velocity of the 1RM trial (0.25 m · s-1), (ii) individual MVT: velocity attained during the 1RM trial, and (iii) Optimal MVT: MVT that eliminated the differences between the actual and predicted 1RM. Two individual load-velocity relationships were modelled considering five (30-50-70-80 %1RM) or six (30-50-70-80-90 %1RM) loading conditions.  Participants attended the laboratory on three occasions separated by 48-96 hours. The first session was utilized to further familiarize the participants with performing the HBD exercise at the maximum intended velocity and to determine their 1RM for the HBD exercise. The second and third testing sessions, identical in format, comprised an incremental loading test (30 %, 50 %, 70 %, 80 %, and 90 % of the 1RM obtained in the first session, culminating in 1RM trials) during the HBD exercise. A validated linear position transducer (GymAware) was affixed to the right side of the barbell. This apparatus served the purpose of recording the MV of all repetitions. | hexagonal deadlift | 90% 1RM general MVT: r= 0.94; ES= 0.02; p= 0.743  80% 1RM general MVT: r= 0.93; ES= -0.04; p= 0.592  90% 1RM individual MVT: r= 0.94; ES= -0.03; p= 0.657  80% 1RM individual MVT: r= 0.94; ES= -0.10; p= 0.191  90% 1RM optimal MVT: r= 0.95; ES= -0.07; p= 0.296  80% 1RM optimal MVT: r= 0.94; ES= -0.17; p= 0.030 | These results suggest that the individual load-velocity relationship offers an accurate estimation of the HBD 1RM in resistance-trained males, and these estimates could maintain similar levels of precision across different types of MVT (general, individual, and optimal) and final tested loads (80 %1RM and 90 %1RM). |
| Jukic et al. (2022) | 18 men performed 2 sessions that consisted of an incremental loading test (20-40-60-80-90% of 1RM) followed by 1RM attempts during the DLn (Deadlift without lifting straps) and DLw (with lifting straps). Predicted 1RMs were calculated by entering both group and individualized mean concentric velocity of the 1RM (V1RM) into an individualized linear and polynomial regression equations, which were derived from the load–velocity relationship of 5 ([20-40-60-80-90% of 1RM], i.e., multiple-point method) or 2 ([40 and 90% of 1RM] i.e., 2 point method) incremental warm-up sets. The MV of all repetitions was collected with a validated linear position transducer (LPT) (GymAware). | deadlift | Deadlift without straps:  Multiple-point polynomial General V1RM: r= 0.92; SEE (kg)= 10.9; ES= 0.08; p= 0.412  Multiple-point polynomial Individual V1RM: r= 0.98; SEE= 5.0; ES= 0.07; p= 0.344  Multiple-point linear General V1RM: r= 0.93; SEE= 10.1; ES= 0.00; p= 0.979  Multiple-point linear Individual V1RM: r= 0.96; SEE= 7.5; ES= 0.02; p= 0.787  Two-point General V1RM: r= 0.92; SEE= 10.7; ES= 0.12; p= 0.235  Two-point Individual V1RM: r= 0.98; SEE= 5.7; ES= 0.11; p= 0.086  Deadlift with straps:  Multiple-point polynomial General V1RM: r= 0.80; SEE= 18.5; ES= 0.01; p= 0.928  Multiple-point polynomial Individual V1RM: r= 0.83; SEE= 17.0; ES= 0.01; p= 0.956  Multiple-point linear General V1RM: r= 0.90; SEE= 13.2; ES= 0.37; p= 0.002  Multiple-point linear Individual V1RM: r= 0.86; SEE= 15.9; ES= 0.40; p= 0.008  Two-point General V1RM: r= 0.93; SEE= 11.6; ES= 0.36; p= 0.001  Two-point Individual V1RM: r= 0.89; SEE= 14.3; ES= 0.40; p= 0.005 | Therefore, these results suggest that the 1RM for both DLn and DLw should not be estimated through the recording of movement velocity if sport professionals are not willing to accept more than 5 kg of absolute errors. |
| Lake et al. (2017) | 12 active men volunteered to participate. Participant deadlift 1RM was assessed at least seven days before load-velocity testing. Instructions emphasised the use of an explosive upward phase and a controlled downward phase. A maximum of five attempts were allowed with the heavier loads. After a minimum of 48 h of rest, participants underwent load-velocity testing. They were required to perform deadlifts with 65%, 70%, 75%, 80%, 85%, and 90% of their 1RM. Three repetitions were performed with the first three loads, while two repetitions were performed with the last three loads. After a minimum of another 48 h of rest, participants performed maximum effort sets to failure with 70% 1RM and 80% 1RM. Bar velocity was recorded during all tests at 1000 Hz using a linear position transducer (Chronojump) attached to the barbell. Barbell MPV and MAV were then obtained from the last repetition of the sets to volitional fatigue with 70% and 80% 1RM and were used as a cut-off value to locate the predicted 1RM from each participant’s load-velocity profile. | deadlift | MPV 70: r= 0.73; 95% LoA (kg)= 30.4; MD (kg)= 16.3 (9.8-22.8)*; g= -0.77 (-1.58-0.08)  MAV 70: r= 0.60; 95% LoA= 35.9; MD= 25.5 (17.8-33.1)**;* g= -1.20 (-2.06- -0.33) MPV 80: r= 0.84; 95% LoA= 23.8; MD= 23.8 (18.8-28.8)*; g= -1.10 (-1.96- -0.24)  MAV 80: r= 0.91; 95% LoA= 18.5; MD= 27.8 (23.8-31.7)*; g= -1.24 (-2.12- -0.37) | Practitioners should not use this method in combination with the application of MVT obtained from the last repetition of sets to volitional fatigue. |
| Larsen et al. (2023) | 28 recreationally active subjects performed load-velocity (L-V) relationship, 1RM, isometric mid thigh pull (IMTP), and maximal repetitions to failure (MRF) tests on 3 separated sessions. Predicted 1RMs for theL-Vrelationship were estimated from a linear regression equation, correlating movement velocity and relative loads. The number of repetitions from the MRF tests (at loads relative to bodyweight) and peak force from the IMTP tests were used in regression equations to predict 1RM. On test day 1, LV-relationship, 1 RM leg press test and the IMTP was done. Approximately 20 minutes after the 1RM test, the IMTP was executed on FDMax force platforms (VALD Performance) in a custom-built cage with a fixed barbell.On test day 2, they performed the MRF. | leg press | L-V vs. 1RM: r= 0.95 (0.89, 0.98); CV= 7.4 (5.5, 9.3); SEE (kg)= 20.4; ES= 0.08 (-0.29, 0.45)  IMTP vs. 1RM: r= 0.67 (0.40, 0.83); CV= 16.1 (11.9, 20.3); SEE= 48,4; ES= 0.00 (-0.37, 0.37)  Max rep * resistance vs. 1RM: r= 0.82 (0.64, 0.91); CV= 12.2 (9.0, 15.4); SEE= 36,7; ES= 0.00 (-0.37, 0.37) | In conclusion, the tests used in this study cannot be used interchangeably, but they represent a good alternative in training settings where 1RM testing is not feasible. |
| Lawson et al. (2024) | The aim of this study was to determine the validity, reliability, and sensitivity of a new linear position transducer (LPT) device (RepOne) to a previously validated LPT (Tendo) during the barbell back squat and bench press exercises. Fourteen recreationally resistance trained individuals (7 males (23.3y) and 7 females (21.9y)) performed three repetitions for the back squat and bench press at loads ranging from 30–90% 1RM. Both devices recorded average (ACV) and peak (PCV) concentric velocities concurrently for every repetition at each load. | back squat & bench press | RepOne vs. Tendo:  Day 1:  Back squat:  PCV:  30%: r=0.90***, R2= 0.77, SEE= 0.10  40%: r= 0.99***, R2= 0.99, SEE= 0.02  50%: r= 0.99***, R2= 0.98, SEE= 0.03  60%: r= 0.99***, R2= 0.99, SEE= 0.01  70%: r=0.98***, R2= 0.96, SEE= 0.03  80%: r= 0.98***, R2= 0.96, SEE= 0.03  90%: r=0.98***, R2= 0.96, SEE= 0.03  ACV:  30%: r= 0.84***, R2= 0.70, SEE= 0.07  40%: r= 0.97***, R2= 0.94, SEE= 0.03  50%: r= 0.98***, R2= 0.96, SEE= 0.02  60%: r= 0.99***, R2= 0.99, SEE= 0.01  70%: r=0.98***, R2= 0.97, SEE= 0.02  80%: r= 0.99***, R2= 0.99, SEE= 0.01  90%: r=0.99***, R2 0.99, SEE= 0.01  Bench press:  PCV:  30%: r=0.99***, R2= 0.97, SEE= 0.04  40%: r= 0.98***, R2= 0.97, SEE= 0.03  50%: r= 0.88***, R2= 0.77, SEE= 0.07  60%: r= 0.99***, R2= 0.99, SEE= 0.02  70%: r=0.98***, R2= 0.95, SEE= 0.02  80%: r= 0.97***, R2= 0.95, SEE= 0.02  90%: r=0.74***, R2= 0.55, SEE= 0.06  ACV:  30%: r= 0.96***, R2= 0.93, SEE= 0.05  40%: r= 0.96***, R2= 0.93, SEE= 0.04  50%: r= 0.81***, R2= 0.66, SEE= 0.07  60%: r= 0.97***, R2= 0.95, SEE= 0.02  70%: r=0.94**, R2= 0.88, SEE= 0.03  80%: r= 0.98***, R2= 0.97, SEE= 0.01  90%: r=0.96***, R2 0.91, SEE= 0.02  Day 2:  Back squat:  PCV:  30%: r=0.96***, R2= 0.93, SEE= 0.06  40%: r= 0.99***, R2= 0.99, SEE= 0.01  50%: r= 0.99***, R2= 0.98, SEE= 0.03  60%: r= 0.99***, R2= 0.99, SEE= 0.01  70%: r=0.99***, R2= 0.99, SEE= 0.01  80%: r= 0.99***, R2= 0.99, SEE= 0.02  90%: r=0.99***, R2= 0.99, SEE= 0.01  ACV:  30%: r= 0.95***, R2= 0.90, SEE= 0.15  40%: r= 0.96***, R2= 0.93, SEE= 0.03  50%: r= 0.99***, R2= 0.98, SEE= 0.02  60%: r= 0.95***, R2= 0.91, SEE= 0.03  70%: r=0.99***, R2= 0.99, SEE= 0.01  80%: r= 0.99***, R2= 0.99, SEE= 0.01  90%: r=0.99***, R2 0.98, SEE= 0.01  Bench press:  PCV:  30%: r=0.93***, R2= 0.86, SEE= 0.10  40%: r= 0.95***, R2= 0.9, SEE= 0.06  50%: r= 0.97***, R2= 0.94, SEE= 0.04  60%: r= 0.98***, R2= 0.96, SEE= 0.03  70%: r=0.99***, R2= 0.99, SEE= 0.01  80%: r= 0.99***, R2= 0.98, SEE= 0.01  90%: r=0.98**, R2= 0.96, SEE= 0.02  ACV:  30%: r= 0.84***, R2= 0.70, SEE= 0.10  40%: r= 0.90***, R2= 0.82, SEE= 0.06  50%: r= 0.97***, R2= 0.93, SEE= 0.03  60%: r= 0.99***, R2= 0.98, SEE= 0.02  70%: r=0.99***, R2= 0.97, SEE= 0.02  80%: r= 0.99***, R2= 0.99, SEE= 0.01  90%: r=0.99***, R2 0.97, SEE= 0.01  RepOne vs. Tendo ICC and 95% CI for PCV and ACV at each %1RM and Rep for Day 1 and Day 2 during back squat:  PCV:  Day 1:  30%: Rep 1= 0.854 (0.545- 0.953)***, Rep 2= 0.994 (0.980- 0.998)***, Rep 3= 0.994 (0.982- 0.998)***  40%: Rep 1= 0.996 (0.987- 0.999)***, Rep 2= 0.997 (0.991- 0.999)***, Rep 3= 0.995 (0.983- 0.998)***  50%: Rep 1= 0.992 (0.975- 0.997)***, Rep 2= 0.994 (0.980- 0.998)***, Rep 3= 0.983 (0.948- 0.995)***  60%: Rep 1= 0.997 (0.991- 0.999)***, Rep 2= 0.997 (0.991- 0.999)***, Rep 3= 0.995 (0.985- 0.998)***  70%: Rep 1= 0.977 (0.928- 0.993)***, Rep 2= 0.997 (0.989- 0.999)***, Rep 3= 0.995 (0.985- 0.998)***  80%: Rep 1= 0.968 (0.901- 0.990)***, Rep 2= 0.996 (0.989- 0.999)***, Rep 3= 0.994 (0.982- 0.998)***  90%: Rep 1=0.996 (0.989- 0.999)***, Rep 2= 0.993 (0.978- 0.998)***, Rep 3= 0.996 (0.985- 0.999)***  Day 2:  30%: Rep 1=0.935 (0.788- 0.980)***, Rep 2= 0.944 (0.816- 0.983)***, Rep 3= 0.993 (0.978- 0.998)***  40%: Rep 1=0.995 (0.984- 0.999)***, Rep 2= 0.995 (0.985- 0.999)***, Rep 3= 0.997 (0.989- 0.999)***  50%: Rep 1= 0.995 (0.984- 0.998)***, Rep 2= 0.993 (0.976- 0.998)***, Rep 3= 0.990 (0.967- 0.997)***  60%: Rep 1= 0.996 (0.985- 0.999)***, Rep 2= 0.997 (0.990- 0.999)***, Rep 3= 0.997 (0.989- 0.999)***  70%: Rep 1= 0.997 (0.990- 0.999)***, Rep 2= 0.997 (0.990- 0.999)***, Rep 3= 0.996 (0.987- 0.999)***  80%:  Rep 1=0.996 (0.988- 0.999)***, Rep 2= 0.997 (0.990- 0.999)***, Rep 3= 0.991 (0.972- 0.997)***  90%: Rep 1=0.997 (0.991- 0.999)***, Rep 2= 0.996 (0.986- 0.999)***, Rep 3= 0.997 (0.990- 0.999)***  ACV  Day 1:  30%: Rep 1=0.826 (0.458- 0.944)**, Rep 2= 0.974 (0.918- 0.992)***, Rep 3= 0.955 (0.860- 0.986)***  40%: Rep 1=0.994 (0.982- 0.998)***, Rep 2= 0.970 (0.907- 0.990)***, Rep 3= 0.989 (0.964- 0.997)***  50%: Rep 1= 0.995 (0.984- 0.998)***, Rep 2= 0.995 (0.985- 0.998)***, Rep 3= 0.983 (0.946- 0.994)***  60%: Rep 1= 0.994 (0.980 -0.998)***, Rep 2= 0.998 (0.993 -0.999)***, Rep 3= 0.996 (0.989- 0.999)***  70%: Rep 1= 0.999 (0.996- 1.000)***, Rep 2= 0.997 (0.992- 0.999)***, Rep 3= 0.979 (0.936- 0.993)***  80%: Rep 1= 0.993 (0.978- 0.998)***, Rep 2= 0.998 (0.994- 0.999)***, Rep 3= 0.997 (0.990- 0.999)***  90%: Rep 1=0.998 (0.994- 0.999)***, Rep 2= 0.999 (0.995- 1.000)***, Rep 3= 0.998 (0.995- 1.000)***  Day 2:  30%: Rep 1=0.993 (0.977- 0.998)***, Rep 2= 0.971 (0.906- 0.991)***, Rep 3= 0.946 (0.812 -0.984)***  40%: Rep 1=0.999 (0.997- 1.000)***, Rep 2=0.996 (0.988 -0.999)*, Rep 3=0.958 (0.863- 0.987)***  50%: Rep 1= 0.989 (0.965- 0.997)***, Rep 2=0.997 (0.990- 0.999)*, Rep 3=0.995 (0.985- 0.999)***  60%: Rep 1= 0.934 (0.784- 0.980)***, Rep 2=0.990 (0.967- 0.997)*, Rep 3=0.997 (0.991- 0.999)***  70%: Rep 1= 0.999 (0.995- 1.000)***, Rep 2=0.996 (0.988- 0.999)*, Rep 3=0.998 (0.994- 0.999)***  80%: Rep 1= 0.997 (0.990- 0.999)***, Rep 2=0.998 (0.995- 1.000)***, Rep 3=0.999 (0.997- 1.000)***  90%: Rep 1=0.980 (0.934- 0.994)***, Rep 2= 0.999 (0.997- 1.000)*, Rep 3= 0.999 (0.997- 1.000)***    Rep One vs. Tendo ICCs (95% CI) for PCV and ACV at each relative load (%1RM) and repetition (Rep) for Day 1 and Day 2 testing during bench press:  PCV:  Day 1:  30%: Rep 1= 0.993 (0.979- 0.998)***, Rep 2= 0.991 (0.970- 0.997)***, Rep 3= 0.989 (0.965- 0.996)***  40%:Rep 1=0.989 (0.966- 0.997)*** , Rep 2= 0.989 (0.966- 0.996)*** , Rep 3= 0.990 (0.966- 0.997)***  50%: Rep 1=0.929 (0.778- 0.977)***, Rep 2=0.935 (0.799- 0.979)***, Rep 3= 0.907 (0.711- 0.970)***  60%:Rep 1=0.998 (0.994- 0.999)*** , Rep 2=0.995 (0.985- 0.998)*** , Rep 3= 0.988 (0.963- 0.996)***  70%: Rep 1=0.989 (0.965- 0.996)***, Rep 2= 0.978 (0.930- 0.993)***, Rep 3= 0.984 (0.947- 0.995)***  80%: Rep 1= 0.995 (0.985- 0.998)***, Rep 2=0.967 (0.897- 0.989)*** , Rep 3= 0.994 (0.983- 0.998)***  90%: Rep 1=0.871 (0.598- 0.959)***, Rep 2= 0.986 (0.956 -0.996)*** , Rep 3= 0.905 (0.689- 0.971)***  Day 2:  30%: Rep 1= 0.961 (0.873 -0.988)*** , Rep 2= 0.941 (0.805- 0.982)***, Rep 3= 0.944 (0.804- 0.984)***  40%:Rep 1=0.988 (0.960- 0.996)*** , Rep 2= 0.938 (0.797- 0.981)***  , Rep 3= 0.993 (0.976- 0.998)***  50%: Rep 1=0.988 (0.960- 0.996)*** , Rep 2=0.986 (0.954- 0.996)***,  Rep 3=0.979 (0.933- 0.994)***  60%:Rep 1=0.985 (0.951- 0.995)***  , Rep 2=0.994 (0.980- 0.998)*** , Rep 3= 0.985 (0.951- 0.995)***  70%: Rep 1=0.995 (0.983- 0.998)*** , Rep 2=0.995 (0.984 -0.998)*** , Rep 3= 0.998 (0.992- 0.999)***  80%: Rep 1=0.995 (0.983- 0.998)*** , Rep 2=0.991 (0.970 -0.997)*** , Rep 3=0.994 (0.981 0.998)***  90%: Rep 1=0.989 (0.963- 0.997)*** , Rep 2=0.979 (0.927- 0.994)***  , Rep 3= 0.996 (0.987 -0.999)***  Bench press:  Day 1:  ACV:  30%: Rep 1= 0.994 (0.980- 0.998)*** , Rep 2=0.966 (0.896 -0.989)*** , Rep 3= 0.799 (0.341- 0.939)***  40%:Rep 1=0.993 (0.977- 0.998)*** , Rep 2= 0.881 (0.628 -0.962)***, Rep 3= 0.967 (0.884- 0.990)***  50%: Rep 1=0.868 (0.589-0.958)***  , Rep 2=0.986 (0.954 -0.996)***,  Rep 3=0.875 (0.610 -0.960)***  60%:Rep 1=0.982 (0.941- 0.995)*** , Rep 2=0.983 (0.944 -0.995)***  , Rep 3= 0.986 (0.954- 0.996)***  70%: Rep 1=0.991 (0.972- 0.997)*** , Rep 2=0.944 (0.825- 0.982)*** , Rep 3= 0.950 (0.843- 0.984)***  80%: Rep 1=0.993 (0.979- 0.998)***, Rep 2=0.987 (0.960 -0.996)*** , Rep 3=0.989 (0.965 -0.996)***  90%: Rep 1=0.992 (0.976- 0.998)*** , Rep 2=0.945 (0.829- 0.982)***, Rep 3= 0.973 (0.911- 0.992)***  Day 2:  ACV:  30%: Rep 1= 0.899 (0.669 -0.969)*** , Rep 2=0.833 (0.451 -0.949)**  , Rep 3= 0.991 (0.970- 0.998)***  40%:Rep 1=0.989 (0.965- 0.997)***, Rep 2= 0.987 (0.959 -0.996)*** , Rep 3=0.979 (0.921- 0.994)***  50%: Rep 1=0.990 (0.967- 0.997)***  , Rep 2=0.984 (0.947 -0.995)*** ,  Rep 3=0.976 (0.922- 0.993)***  60%:Rep 1=0.998 (0.995- 0.999)*** , Rep 2=0.986 (0.953- 0.996)*** , Rep 3= 0.995 (0.984- 0.999)***  70%: Rep 1=0.988 (0.960 -0.996)*** , Rep 2=0.997 (0.989 -0.999)*** , Rep 3= 0.990 (0.968 -0.997)***  80%: Rep 1=0.997 (0.989- 0.999)*** , Rep 2=0.992 (0.975 -0.998)***, Rep 3=0.997 (0.992- 0.999)***  90%: Rep 1=0.990 (0.969- 0.997)*** , Rep 2=0.988 (0.959 -0.997)***, Rep 3= 0.996 (0.985- 0.999)***  RepOne intra-rater reliability between Day 1 and Day 2 testing: ICC (95% CI) for PCV and ACV at reach relative load (%) and Rep during back squat and bench press:  Back squat (Day 1 & 2):  RepOne PCV:  30%: Rep 1=0.890 (0.619- 0.968)*** , Rep 2=0.850 (0.508- 0.954)***, Rep 3=0.862 (0.547- 0.958)***  40%: Rep 1=0.959 (0.866- 0.988)*** , Rep 2=0.882 (0.613- 0.964)***, Rep 3= 0.927 (0.746- 0.979)***  50%: Rep 1=0.874 (0.588- 0.962)***  , Rep 2=0.891 (0.643- 0.967)***, Rep 3=0.849 (0.507- 0.954)***  60%: Rep 1=0.946 (0.823- 0.983)***  , Rep 2=0.901 (0.676- 0.970)*** , Rep 3=0.855 (0.525- 0.956)***  70%: Rep 1=0.942 (0.810- 0.982)** , Rep 2=0.869 (0.569- 0.960)***, Rep 3=0.900 (0.672- 0.969)***  80%: Rep 1=0.909 (0.700- 0.972)***  , Rep 2=0.930 (0.770- 0.979)*** , Rep 3=0.896 (0.660- 0.968)***  90%: Rep 1=0.883 (0.616- 0.964)*** , Rep 2=0.954 (0.839- 0.987)*** , Rep 3=0.958 (0.843- 0.989)***  RepOne ACV:  30%:Rep 1=0.936 (0.792- 0.981)***, Rep 2= 0.785 (0.296- 0.934)**, Rep 3= 0.902 (0.680- 0.970)***  40%:Rep 1=0.957 (0.860- 0.987)***, Rep 2=0.910 (0.706- 0.973)***, Rep 3=0.909 (0.683- 0.974)***  50%:Rep 1=0.919 (0.735- 0.975)***, Rep 2=0.928 (0.763- 0.978)***, Rep 3=0.929 (0.768- 0.978)***  60%:Rep 1=0.857 (0.531- 0.956)***, Rep 2=0.806 (0.364- 0.941)** , Rep 3=0.911 (0.708- 0.973)***  70%:Rep 1=0.966 (0.888- 0.990)***, Rep 2= 0.875 (0.591- 0.962)***, Rep 3= 0.929 (0.767- 0.978)***  80%:Rep 1=0.892 (0.647- 0.967)***, Rep 2=0.968 (0.894- 0.990)***, Rep 3= 0.905 (0.689- 0.971)***  90%:Rep 1=0.856 (0.528- 0.956)***, Rep 2=0.925 (0.741- 0.979)***, Rep 3= 0.968 (0.881- 0.991)***  Bench press (Day 1 & 2):  RepOne PCV:  30%:Rep 1=0.914 (0.719- 0.974)***, Rep 2=0.866 (0.561- 0.959)***, Rep 3=0.926 (0.743- 0.979)***  40%:Rep 1=0.955 (0.851- 0.986)***, Rep 2=0.882 (0.614- 0.964)***, Rep 3= 0.894 (0.633- 0.970)***  50%:Rep 1=0.787 (0.302- 0.935)***, Rep 2=0.885 (0.623- 0.965)***, Rep 3=0.931 (0.773- 0.979)***  60%:Rep 1=0.945 (0.821- 0.983)***, Rep 2=0.880 (0.608- -0.963)***, Rep 3=0.960 (0.868- 0.988)***  70%:Rep 1=0.878 (0.600- 0.963)***, Rep 2=0.866 (0.561- 0.959)***, Rep 3= 0.925 (0.738- 0.978)***  80%:Rep 1=0.929 (0.767- 0.978)***, Rep 2=0.915 (0.721- 0.974)***, Rep 3=0.844 (0.488- 0.952)***  90%:Rep 1=0.786 (0.297- 0.935)***, Rep 2=0.981 (0.928- 0.995)***, Rep 3=0.763 (0.120- 0.936)*  RepOne ACV:  30%:Rep 1=0.815 (0.393- 0.944)**, Rep 2=0.766 (0.234- 0.929)**, Rep 3=0.895 (0.637- 0.970)***  40%:Rep 1=0.935 (0.787- 0.980)***, Rep 2=0.854 (0.523- 0.956)***, Rep 3= 0.908 (0.657- 0.975)***  50%:Rep 1=0.883 (0.617- 0.964)***, Rep 2=0.768 (0.240- 0.929)**, Rep 3=0.839 (0.472- 0.951)**  60%:Rep 1=0.970 (0.901- 0.991)***, Rep 2=0.919 (0.734- 0.975)***, Rep 3=0.911 (0.707- 0.973)***  70%:Rep 1=0.885 (0.625- 0.965)***, Rep 2=0.810 (0.378- 0.942)**, Rep 3=0.830 (0.444- 0.948)**  80%:Rep 1=0.955 (0.853- 0.986)***, Rep 2=0.937 (0.793- 0.981)**, Rep 3=0.785 (0.295- 0.934)*  90%:Rep 1=0.873 (0.585- 0.961)***, Rep 2=0.868 (0.543- 0.962)**, Rep 3=0.586 (−0.539- 0.889)  Tendo intra-rater reliability between Day 1 & 2: ICCs (95% CI) for PCV and ACV at each relative load (%1RM) and Rep during abc squat and bench press:  PCV:  30%:Rep 1=0.938 (0.796- 0.981)***, Rep 2= 0.885 (0.622- 0.965)***, Rep 3=0.855 (0.524- 0.956)***  40%:Rep 1=0.952 (0.844- 0.985)*** , Rep 2=0.888 (0.632- 0.966)***, Rep 3=0.942 (0.809- 0.982)***  50%:Rep 1=0.887 (0.630- 0.966)***, Rep 2=0.905 (0.689- 0.971)***, Rep 3=0.900 (0.672- 0.969)***  60%:Rep 1=0.949 (0.834- 0.985)*** , Rep 2=0.912 (0.712- 0.973)***, Rep 3= 0.867 (0.563- 0.959)***  70%:Rep 1=0.949 (0.832- 0.984)***, Rep 2=0.890 (0.641- 0.967)***, Rep 3=0.907 (0.696- 0.972)***  80%:Rep 1=0.893 (0.651- 0.967)*** , Rep 2=0.929 (0.767- 0.978)***, Rep 3=0.903 (0.682- 0.970)***  90%:Rep 1=0.886 (0.626- 0.965)***, Rep 2=0.953 (0.836- 0.986)***, Rep 3=0.954 (0.82-7 0.987)***  ACV:  30%:Rep 1=0.948 (0.831- 0.984)*** , Rep 2=0.868 (0.568- 0.960)***, Rep 3=0.897 (0.644- 0.970)***  40%:Rep 1=0.949 (0.834- 0.985)***, Rep 2=0.944 (0.815- 0.983)***, Rep 3=0.958 (0.863- 0.987)***  50%:Rep 1=0.928 (0.764- 0.978)*** , Rep 2=0.950 (0.837- 0.985)***, Rep 3=0.931 (0.77-4 0.979)***  60%:Rep 1=0.915 (0.720- 0.974)*** , Rep 2=0.835 (0.461- 0.950)***, Rep 3=0.910 (0.706- 0.973)***  70%:Rep 1=0.971 (0.906- 0.991)***, Rep 2=0.898 (0.665- 0.969)***, Rep 3=0.917 (0.728- 0.975)***  80%:Rep 1=0.875 (0.592- 0.962)*** , Rep 2=0.966 (0.889- 0.990)***, Rep 3=0.907 (0.694- 0.971)***  90%:Rep 1=0.866 (0.562- 0.959)*** , Rep 2=0.925 (0.739- 0.978)***, Rep 3=0.970 (0.889- 0.992)***  Bench press:  PCV:  30%:Rep 1=0.939 (0.801- 0.981)***, Rep 2=0.934 (0.784- 0.980)***, Rep 3=0.966 (0.882 0.990)***  40%:Rep 1=0.970 (0.901- 0.991)***, Rep 2=0.972 (0.908- 0.991)***, Rep 3=0.928 (0.765 0.978)***  50%:Rep 1=0.938 (0.796- 0.981)***, Rep 2=0.886 (0.626- 0.965)***, Rep 3=0.931 (0.773 0.979)***  60%:Rep 1=0.954 (0.848- 0.986)**, Rep 2=0.902 (0.680- 0.970)***, Rep 3=0.975 (0.917 0.992)***  70%:Rep 1=0.897 (0.663- 0.969)***, Rep 2=0.845 (0.493- 0.953)***, Rep 3=0.883 (0.593 0.966)***  80%:Rep 1=0.913 (0.716- 0.974)**, Rep 2=0.884 (0.621- 0.965)***, Rep 3=0.910 (0.705 0.973)***  90%:Rep 1=0.716 (0.068- 0.913)*, Rep 2=0.925 (0.741- 0.979)***, Rep 3=0.714 (−0.150 0.926)*  ACV:  30%:Rep 1=0.926 (0.756- 0.977)***, Rep 2=0.859 (0.537- 0.957)***, Rep 3= 0.809 (0.374- 0.942)**  40%:Rep 1=0.952 (0.843- 0.985)***, Rep 2=0.935 (0.788- 0.980)***, Rep 3=0.892 (0.647- 0.967)***  50%:Rep 1=0.939 (0.799- 0.981)***, Rep 2=0.919 (0.733- 0.975)***, Rep 3= 0.907 (0.696- 0.972)***  60%:Rep 1=0.960 (0.861- 0.988)***, Rep 2=0.901 (0.654- 0.971)***, Rep 3=0.932 (0.765- 0.981)***  70%:Rep 1=0.913 (0.714- 0.973)***, Rep 2=0.924 (0.752- 0.977)***, Rep 3= 0.900 (0.673- 0.970)***  80%:Rep 1=0.956 (0.857- 0.987)***, Rep 2=0.924 (0.752- 0.977)***, Rep 3=0.773 (0.256- 0.931)**  90%:Rep 1=0.845 (0.492- 0.953)***, Rep 2=0.877 (0.503- 0.969)**, Rep 3=0.740 (−0.046- 0.935)* | The authors suggest not using these devices interchangeably. If switching from one device to another, it's recommended to retest athletes or clients if loading parameters are referenced from a load-velocity profile using a specific device or when interpreting data to determine athlete readiness. Furthermore, practitioners are encouraged to conduct in-house calculations of measurement error using SEM or SDC, and caution is warranted when interpreting relatively small velocity changes at very heavy (> 80%) or very light (< 50%) loads. |
| Oberhofer et al. (2021) | Thirty physically healthy, recreationally trained athletes performed, on one test day, four consecutive sets of barbell deadlift, barbell bench press, and barbell back squat exercises with increasing loads from 60% to 80% of their estimated 1RM. The loading regime was chosen to enable the indirect prediction of 1RM via the L-V relationship. The acceleration of the left wrist and the velocity of the barbell were simultaneously measured during all repetitions for all exercises using the Apple Watch Sport on the participant’s left wrist and an LPT (GymAware) strapped around the bar.  For the 1RM Prediction, three different equations to estimate the 1RMs of each subject for the barbell bench press, the barbell back squat, and the barbell deadlift were adopted. One equation was based on the repetition to failure method, and two of the equations were based on well-established linear regression techniques to derive the 1RM based on the L-V relationship. Thereby, the L-V equation proposed in Sayers, Schlaeppi, Hitz and Lorenzetti is based on findings that the peak vertical bar velocity yields more accurate predictions of Smith Machine bench press 1RM than mean bar velocity. | deadlift, bench press, back squat | Bench press:  Mean: r= 0.99; $R^{2}$= 0.9799; RMSE= 4.63; p<0.01  Peak: r= 0.99; $R^{2}$= 0.9791; RMSE= 4.00; p= 0.04  Peak, Mean: r= 0.99; $R^{2}$= 0.9846; RMSE= 5.10; p<0.01  Back squat:  Mean: r= 0.90; $R^{2}$= 0.8129; RMSE= 13.86; p<0.01  Peak: r= 0.89; $R^{2}$= 0.7919; RMSE= 19.76; p<0.01  Peak, Mean: r= 0.96; $R^{2}$= 0.9163; RMSE= 11.41; p<0.01  Deadlift:  Mean: r= 0.89; $R^{2}$= 0.6540; RMSE= 22.71; p<0.01  Peak: r= 0.84; $R^{2}$= 0.7078; RMSE= 21.82; p= 0.01  Peak, Mean: r= 0.90; $R^{2}$= 0.8094; RMSE= 16.90; p= 0.68 | power athletes may be more willing and experienced with direct 1RM testing, and should be considered for further validation of 1RM predictions from wearable and smartphone-based technology in future work |
| Pérez-Castilla et al. (2019) | 11 men performed an incremental test on Smith machine against 5 loads, followed by 1RM attempts. The mean velocity was simultaneously measured by 1 linear velocity transducer (T-Force), 2 linear position transducers (Chronojump and Speed4Lift), 1 camera-based optoelectronic system (Velowin), 2 inertial measurement units (PUSH band and Beast sensor), and 1 smartphone application (PowerLift). The velocity recorded at the 5 loads (45–55–65–75–85%1RM) or only at the 2 most distant loads (45-85%1RM) were considered for the multiple- and 2-point methods, respectively.  Subjects performed 2 testing sessions. The first session was used for anthropometric measures and to determine the bench press 1RM. The second session began with the same warm-up described above. Subjects then performed 3 repetitions against 5 incremental loads (45–55–65–75–85%1RM), followed by 1RM attempts (a maximum of 3 attempts were allowed). | bench press | Multiple-point method:  T-Force: r= 0.97 (0.91, 0.99); SEE= 3.16 (2.30, 5.20); ES= 0.35 (-1.19, 0.49); p= 0.001  Chronojump: r= 0.95 (0.84, 0.98); SEE= 4.11 (3.00, 6.76); ES= -0.13 (-0.71, 0.97); p= 0.0195  Speed4Lift: r= 0.97 (0.91, 0.99); SEE= 3.02 (2.21, 4.98); ES= 0.30 (-1.14, 0.54); p= 0.002  Velwoin: r= 0.97 (0.91, 0.99); SEE= 3.15 (2.30, 5.18); ES= 0.18 (-1.02, 0.66); p= 0.045  PUSH band: r= 0.94 (0.82, 0.98); SEE= 4.45 (3.25, 7.32); ES= -0.83 (-0.04, 1.70); p<0.001 Beast sensor: r= 0.68 (0.24, 0.89); SEE= 9.44 (6.89, 15.5); ES= -0.84 (-0.04, 1.71); p= 0.005  PowerLift: r= 0.94 (0.82, 0.98); SEE= 4.46 (3.25, 7.33); ES= 0.37 (-1.22, 0.47); p= 0.005  2-point Method:  T-Force: r= 0.97 (0.91, 0.99); SEE= 3.12 (2.27, 5.13); ES= 0.35 (-1.19, 0.49); p= 0.001  Chronojump: r= 0.96 (0.87, 0.99); SEE= 3.68 (2.68, 6.05); ES= -0.08 (-0.76, 0.91); p= 0.372  Speed4Lift: r= 0.97 (0.91, 0.99); SEE= 3.13 (2.28, 5.15); ES= 0.31 (-1.15, 0.53); p= 0.001  Velwoin: r= 0.97 (0.90, 0.99); SEE= 3.27 (2.39, 5.38); ES= 0.24 (-1.08, 0.59); p= 0.013  PUSH band: r= 0.93 (0.79, 0.98); SEE= 4.80 (3.50, 7.90); ES= -0.70 (-0.16, 1.57); p<0.001 Beast sensor: r= 0.50 (-0.03, 0.81); SEE= 11.2 (8.15, 18.4); ES= 0.36 (-1.20, 0.49); p= 0.258  PowerLift: r= 0.95 (0.84, 0.98); SEE= 4.13 (3.02, 6.80); ES= 0.40 (-1.24, 0.45); p= 0.002 | These results highlight that the accuracy of 1RM prediction methods based on movement velocity are device-dependent with the inertial measurement units providing the least accurate estimate of the 1RM. |
| Pérez-Castilla et al. (2021) (PUSH Band) | The aim of this study was to examine the test-retest reliability and concurrent validity of the PUSH Band 2.0 to measure barbell’s velocity during unconstrained (free-weights) and constrained (Smith machine) squat exercises. After an initial assessment of the Smith machine squat one-repetition maximum (1RM), 24 resistance-trained males (age=23.1+-3.4years (range: 20-33 years) completed one or two testing sessions separated by 7days. In one session, the squat was performed with free-weights (n=22), while in another session, the Smith machine was used (n=16). Both testing sessions consisted of two blocks of eight repetitions (three repetitions at 45%1RM, three repetitions at 65%1RM, and two repetitions at 85%1RM). | Free-weight squat & Smith machine squat | Reliability of mean concentric velocity recorded by the PUSH Band 2.9 and the T-Force System at different relative loads during free-weight and Smith machine squat exercise:  free-weight squat:  PUSH Band 2.0:  45%: p= 0.062, ES: 0.37, SEM: 0.07(0.05,0.10), CV:7.06(5.43,10.09)  65%: p= 0.392, ES:-0.17, SEM:0.07(0.05,0.10), CV:8.78(6.76,12.55)  85%: p= 0.954, ES:-0.01, SEM:0.08(0.06,0.11), CV:13.14 (10.11,18.77)  T-Force System:  45%: p= 0.323, ES:-0.14, SEM:0.04(0.03,0.05), CV:4.14(3.19,5.92)  65%: p=0.079, ES:-0.23, SEM:0.03(0.03,0.05), CV: 4.53(3.49,6.48)  85%: p= 0.331, ES:-0.12, SEM:0.04(0.03,0.06), CV: 7.86(6.05,11.24)  Smith machine:  PUSH Band 2.0  45%: p=0.194, ES:-0.38, SEM:0.05(0.04,0.08), CV: 5.81(4.29,8.99)  65%: p= 0.084, ES:-0.31, SEM:0.04(0.03,0.07), CV:5.97(4.41,9.25)  85%: p= 0.975, ES:-0.01, SEM:0.05(0.04,0.08), CV: 9.57(7.07,14.81)  T-Force System:  45%: p= 0.884, ES:-0.03, SEM:0.03(0.02,0.04), CV:2.95(2.18,4.56)  65%: p= 0.001, ES:-0.65, SEM:0.03(0.02,0.05), CV:4.43(3.27,6.85)  85%: p= 0.375, ES:-0.15, SEM:0.04(0.03,0.06), CV: 7.45(5.50,11.52)  Validity of the PUSH Band 2.0 with respect to the T-Force System for the recording of mean concentric velocity at different relative loads during the free-weight and Smith machine squat:  Free-weight squat:  45%: p=0.295, ES:0.18 (20.17, 0.52), r= 0.75 (0.49, 0.89)  65%: p= 0.044, ES: 0.44 (0.01, 0.86), r=0.63 (0.28, 0.83)  85%: p=0.002, ES:0.4 (0.27, 1.01), r=0.66 (0.32, 0.84)  Smith machine:  45%: p=0.248, ES:0.33 (-0.25, 0.91), r= 0.58 (0.11, 0.83)  65%: p=0.212, ES:0.26 (-0.16, 0.68), r= 0.82 (0.54, 0.93)  85%: p=0.001, ES:0.61 (0.28, 0.94), r=0.82 (0.55, 0.94) | The PUSHBand 2.0 is a reliable, but not valid, IMU based wearable technology to measure the barbell velocity during the free-weight and Smith machine squat exercises. Therefore, unless velocity monitoring is done with the same PUSH Band 2.0 device, the practical applications of VBT may be limited |
| Pérez-Castilla et al. (2021) (iLOAD) | This study aimed to evaluate the reliability and validity of a smartphone application (iLOAD) for the monitoring of mean concentric velocity (MV) during resistance training sets. Twenty males (age=23.0 +- 2.6 years [range: 18–30 years]) completed 2 identical sessions consisting of one set of 10 repetitions against 4 loads (25, 40, 55, 70% of the one repetition maximum [1RM]) during the back squat and bench press exercises. The MV of the 5 initial repetitions and for the whole set were determined simultaneously with the iLOAD application and a linear velocity transducer (LVT). Two independent researchers operated the iLOAD application during the experimental sessions to evaluate the interrater agreement for the assessment of MV. | back squat & bench press | Mean concentric velocity recorded with the iLOAD app by 2 independent raters during the back squat in the first 5 repetitions of the set and for the whole set:  1st -5th:  rater 1 vs rater 2: r=0.98  Difference between raters vs. Mean value of both raters: r2= 0.01; ES=-0.03  1st-10th:  rater 1 vs rater 2: r=1.00  Difference between raters vs. Mean value of both raters: r2= 0.03; ES=-0.03  Mean concentric velocity recorded with the iLOAD app by 2 independent raters during the bench press in the first 5 repetitions of the set and for the whole set:  1st -5th:  rater 1 vs rater 2: r=0.98  Difference between raters vs. Mean value of both raters: r2= 0.01; ES=0.02  1st-10th:  rater 1 vs rater 2: r=0.99  Difference between raters vs. Mean value of both raters: r2= 0.03; ES=0.05  Between-session reliability of the mean concentric velocity recorded by the iLOAD app and the linear velocity transducer (LVT) at different intensities during the back squat exercise:  iLOAD:  25%:  1st -5th: ES(95%CI): -0.25(20.88to0.37), SEM(95%CI): 0.10(0.08–0.15), CV(95%CI): 9.71(7.38–14.2)  1st-10th: ES(95%CI): -0.25(20.87to0.37), SEM(95%CI): 0.10(0.08–0.15), CV(95%CI): 9.79(7.45–14.3)  40%:  1st -5th: ES(95%CI): 0.00(20.62to0.62), SEM(95%CI): 0.06(0.05–0.09), CV(95%CI): 6.72(5.11–9.82)  1st-10th: ES(95%CI):-0.11(20.73to0.51), SEM(95%CI): 0.05(0.04–0.07), CV(95%CI): 5.61(4.27–8.20)  55%:  1st -5th: ES(95%CI):-0.29(20.92to0.33), SEM(95%CI): 0.07(0.06–0.11), CV(95%CI): 9.45(7.19–13.8)  1st-10th: ES(95%CI):-0.22(20.85to0.39), SEM(95%CI): 0.06(0.05–0.09), CV(95%CI): 8.63(6.56–12.6)  70%:  1st -5th: ES(95%CI): -0.37(21.00to0.25), SEM(95%CI): 0.09(0.07–0.14), CV(95%CI): 16.1(12.2–23.5)  1st-10th: ES(95%CI):-0.39(21.02to0.24), SEM(95%CI): 0.09(0.07–0.13), CV(95%CI): 17.4(13.2–25.3)  LVT:  25%:  1st -5th: ES(95%CI): 0.00(20.62to0.62), SEM(95%CI): 0.07(0.05–0.10), CV(95%CI): 7.02(5.34–10.3)  1st-10th: ES(95%CI): 0.00(20.62to0.62), SEM(95%CI): 0.08(0.06–0.12), CV(95%CI): 8.18(6.22–11.9)  40%:  1st -5th: ES(95%CI):0.15(20.47to0.77), SEM(95%CI): 0.04(0.03–0.06), CV(95%CI): 4.94(3.76–7.21)‡  1st-10th: ES(95%CI):0.15(20.47to0.77), SEM(95%CI): 0.04(0.03–0.06), CV(95%CI): 4.94(3.76–7.21)‡  55%:  1st -5th: ES(95%CI): -0.16(20.78to0.46), SEM(95%CI): 0.04(0.03–0.06), CV(95%CI): 5.64(4.29–8.24)‡  1st-10th: ES(95%CI):-0.16(20.78to0.46), SEM(95%CI): 0.05(0.04–0.07), CV(95%CI): 6.69(5.09–9.77)  70%:  1st -5th: ES(95%CI):-0.28(20.90to0.35), SEM(95%CI): 0.07(0.05–0.10), CV(95%CI): 11.3(8.59–16.5)  1st-10th: ES(95%CI):-0.40(21.03to0.22), SEM(95%CI): 0.07(0.05–0.10), CV(95%CI): 12.8(9.70–18.6)  Between-session reliability of the mean concentric velocity recorded by the iLOAD app and the linear velocity transducer (LVT) at different intensities during the bench press exercise:  iLOAD:  25%:  1st -5th:ES(95%CI): -0.25(20.87to0.37), SEM(95%CI): 0.11(0.09–0.17), CV(95%CI): 9.05(6.88–13.2)  1st-10th: ES(95%CI):-0.25(20.88to0.37), SEM(95%CI): 0.11(0.08–0.16), CV(95%CI): 8.66(6.59–12.7)  40%:  1st -5th:ES(95%CI):-0.08(20.70to0.54), SEM(95%CI): 0.09(0.07–0.13), CV(95%CI): 8.17(6.22–11.9)  1st-10th: ES(95%CI):-0.09(20.71to0.53), SEM(95%CI): 0.08(0.06–0.11), CV(95%CI): 7.10(5.40–10.4)  55%:  1st -5th:ES(95%CI): -0.09(20.71to0.53), SEM(95%CI): 0.07(0.05–0.10), CV(95%CI): 7.18(5.46–10.5)  1st-10th: ES(95%CI): 0.00(20.62-0.62), SEM(95%CI): 0.08(0.06–0.11), CV(95%CI): 9.20(7.00–13.4)  70%:  1st -5th:ES(95%CI): 0.10(20.52to0.72), SEM(95%CI): 0.08(0.06–0.12), CV(95%CI): 12.1(9.23–17.7)  1st-10th: ES(95%CI): -0.10(20.72to0.52), SEM(95%CI): 0.07(0.05–0.10), CV(95%CI): 11.4(8.66–16.6)  LVT:  25%:  1st -5th:ES(95%CI): -0.24(20.87-0.38), SEM(95%CI): 0.09(0.07–0.13), CV(95%CI): 7.02(5.34–10.3)  1st-10th: ES(95%CI):-0.16(20.78-0.46), SEM(95%CI): 0.09(0.07–0.13), CV(95%CI): 6.98(6.31–10.2)  40%:  1st -5th:ES(95%CI): -0.12(20.74t-0.51), SEM(95%CI): 0.05(0.04–0.09), CV(95%CI): 4.55(3.46–6.65)‡  1st-10th: ES(95%CI): -0.11(20.73-0.51), SEM(95%CI): 0.05(0.04–0.07), CV(95%CI): 4.67(3.55–6.82)‡  55%:  1st -5th:ES(95%CI): -0.26(20.88to0.36), SEM(95%CI): 0.04(0.03–0.05), CV(95%CI): 4.51(3.43–6.58)‡  1st-10th: ES(95%CI):-0.12(20.74to0.50), SEM(95%CI): 0.04(0.03–0.05), CV(95%CI): 4.76(3.62–6.95)‡  70%:  1st -5th:ES(95%CI): 0.00(20.62to0.62), SEM(95%CI): 0.03(0.03–0.05), CV(95%CI): 6.37(4.84–9.30)‡  1st-10th: ES(95%CI): -0.14(20.76to0.48), SEM(95%CI): 0.04(0.03–0.06), CV(95%CI): 8.26(6.28–12.1)‡  Validity of the iLOAD app with respect to the LVT for the recording of mean concentric velocity at different intensities during the back squat and bench press:  back squat  25%:  1st -5th:p=0.003, ES(95%CI):0.96 (0.30 to 1.61), r=0.83 (0.62–0.93)  1st-10th:p= 0.442, ES(95%CI):0.26 (-0.36 to 0.88), r= 0.83 (0.62–0.93)  40%:  1st -5th:p=0.016, ES(95%CI):0.80 (0.16 to 1.45), r=0.72 (0.41–0.88)  1st-10th:p=0.184, ES(95%CI):0.43 (-0.19 to 1.06), r=0.81 (0.57–0.92)  55%:  1st -5th:p=0.009, ES(95%CI):0.84 (0.19 to 1.49) , r=0.70 (0.38–0.87)  1st-10th:p= 0.308, ES(95%CI):0.31 (-0.32 to 0.93), r= 0.72 (0.42–0.88)  70%:  1st -5th:p=0.468, ES(95%CI):0.22 (-0.41 to 0.84) , r=0.79 (0.54–0.91)  1st-10th:p= 0.511, ES(95%CI):-0.20 (-0.82 to 0.43), r= 0.79 (0.53–0.91)  bench press:  25%:  1st -5th:p=0.271, ES(95%CI):0.42 (-0.20 to 1.05), r= 0.74 (0.51–0.88)  1st-10th:p= 0.914, ES(95%CI):0.24 (-0.38 to 0.87), r=0.80 (0.60–0.90)  40%:  1st -5th:p=0.008, ES(95%CI): 0.71 (0.08 to 1.35), r= 0.84 (0.64–0.94)  1st-10th:p=0.060, ES(95%CI):0.56 (-0.07 to 1.19), r=0.90 (0.75–0.96)  55%:  1st -5th:p<0.001, ES(95%CI):0.92 (0.27 to 1.57) , r=0.90 (0.76–0.96)  1st-10th:p= 0.001, ES(95%CI):0.83 (0.19 to 1.48), r= 0.86 (0.67–0.94)  70%:  1st -5th:p=0.001, ES(95%CI):1.04 (0.38 to 1.70), r= 0.83 (0.62–0.93)  1st-10th:p= 0.009, ES(95%CI):0.88 (0.23 to 1.53), r= 0.89 (0.74–0.96) | The iLOAD app could overestimate the MV of the set compared to the LVT likely because the downward phase of the exercise is performed at a faster velocity than the upward phase, and (b) the reliability and validity of the iLOAD app could be compromised when lifting heavy loads or when the sets are performed to or close to failure due to a greater difficulty to complete the set without introducing short breaks between successive repetitions. The introduction of short breaks between repetitions could explain the higher MV values for the LVT during the squat exercise when the sets were performed at a low velocity (i.e., 75% of 1RM). Therefore, the findings of this study suggest that the iLOAD app is a cost-effective, portable, and easy-to-use smartphone app that can be confidently used to quantify the MV of training sets during the squat and bench press exercises not performed to failure. |
| Picerno et al. (2016) | The proposed force–velocity-based method (FVM) was tested on 37 participants with no specific resistance-training experience, and the predicted 1RM was compared to that achieved using a direct method (DM) in chest-press (CP) and leg-press (LP) exercises. Kinematics of lifting was measured using a triaxial inertial sensor (Sensorize).  1RM at CP and LP were assessed in the same session. DM and FVM protocols were performed in two separate sessions. In a first session, a direct determination of 1RM (DM) was carried out according to the recommendations of Brown and Weir (2001). In a second session, 1RM was estimated using the proposed methodology (FVM). Specifically, after a warm-up of 20 repetitions with a moderate weight, the protocol consisted in performing three sets of 5–6, 4–5, and 3–4 repetitions at 50, 65, and 80 % of the assessed 1RM, respectively. | chest press, leg press | Chest press:  Pearson= 0.999; SEE (kg)= 1.2; MAE (kg)= 1.4; MAPE (%)= 1.5  Leg press:  Pearson= 0.999; SEE= 2.1; MAE= 1.8; MAPE= 0.8 | These findings suggest the use of the proposed methodology as a valid alternative to other indirect approaches for 1RM prediction. |
| Ruf et al. (2018) | Data from 11 resistance trained athletes were analyzed performing three 1RM assessments separated by at least 3 days. Reliability was assessed by comparing predicted 1RMs of sessions 2 and 3, whereas for validity purposes, predicted 1RMs of session 3 were compared with actual 1RMs of session 2. Mean concentric velocity at 1RM (v at 1RM) was entered in individualized linear regression equations, derived from the load-velocity relationship for 3 (20–60%, 40–80%, and 60–90% of 1RM), 4 (20–80% and 40–90% of 1RM), and 5 (20–90% of 1RM) incremental loads to predict 1RMs. Barbell displacement with respect to time was monitored by commercial linear position transducers (GymAware). | Deadlift | Reliability between sessions for predicted 1RM models, actual 1RM, and velocity at 1RM (v at 1RM).  20-60%: ICC= 0.95 (0.87-0.98); CV= 4.01 (2.95-6.47); ES= 0.04 (-0.12-0.19); TE= 7.13 (5.27-11.63)  40-80%: ICC= 0.95 (0.86-0.98); CV= 4.39 (3.22-7.08); ES= -0.13 (-0.31-0.04); TE= 7.52 (5.56-11.99)  60-90%: ICC= 0.96 (0.89-0.99); CV= 3.29 (2.42-5.29); ES= -0.01 (-0.16-0.13); TE= 6.09 (4.50-9.70)  20-80%: ICC= 0.97 (0.92-0.99); CV= 2.80 (2.06-4.49); ES= -0.05 (-0.16-0.07); TE= 5.41 (4.00-8.61)  40-90%: ICC= 0.97 (0.91-0.99); CV= 3.24 (2.38-5.20); ES= -0.05 (-0.19-0.09); TE= 5.82 (4.30-9.27)  20-90%: ICC= 0.97 (0.93-0.99); CV= 2.64 (1.95-4.24); ES= -0.01 (-0.13-0.10); TE= 5.08 (3.76-8.09)  1RM: ICC= 0.99 (0.96-1.00); CV= 1.89 (1.39-3.03); ES= 0.04 (-0.03-0.12); TE= 3.40 (2.51-5.41)  v@1RM: ICC= 0.63 (0.19-0.86); CV= 15.65 (11.34-26.07); ES= -0.30 (-0.78-0.17)  Validity of the predicted 1RM models compared with actual 1RM.  20-60%: ICC= 0.88 (0.67-0.96); CV= 5.46 (4.15-8.60) ES= 0.39 (0.19-0.59); TE= 13.74 (10.03-22.57)  40-80%: ICC= 0.92 (0.78-0.98); CV= 4.42 (3.37-6.84); ES= 0.25 (0.10-0.41); TE= 11.05 (8.07-18.10)  60-90%: ICC= 0.93 (0.81-0.98); CV= 4.01 (3.13-6.33); ES= 0.25 (0.11-0.41); TE= 10.34 (7.60-17.01)  20-80%: ICC= 0.91 (0.76-0.97); CV= 4.66 (3.55-7.22); ES= 0.30 (0.13-0.48); TE= 11.68 (8.54-19.20)  40-90%: ICC= 0.95 (0.85-0.98); CV= 3.64 (2.81-5.55); ES= 0.26 (0.12-0.39); TE= 9.09 (6.66-15.05)  20-90%: ICC= 0.94 (0.82-0.98; CV= 4.00 (3.07-6.18); ES= 0.29 (0.14-0.43); TE= 10.11 (7.37-16.61) | However, predicted 1RMs computed from all submaximal load ranges substantially overestimated the actual 1RM with considerable differences between athletes. Although 1RM predictions showed high reliability, they all overestimated the actual 1RM, which was stable between sessions. Therefore, it is not recommended to apply the prediction models used in this study to compute daily 1RMs. |
| Sandau et al. (2021) | Eight elite weightlifters carried out a 1RM snatch test followed by a snatch pull test with loads of 80% and 110% of the previously determined 1RM snatch. They were encouraged to reach their maximal snatch performance (i.e., 100% = actual 1RM) using 6–8 loads with 1–2 repetitions per load condition. After the 1RM snatch test, a 5 min rest was implemented before the snatch pull test was conducted. During the snatch pull test, weightlifters were encouraged to lift two consecutive repetitions at 80% and 110% of the previously determined 1RM snatch at maximal effort and starting with the 110% load condition. The snatch and the snatch pull lifts were video recorded (Canon) and analyzed using a custom-made real-time barbell tracking software (Realanalyzer). The vertical barbell velocity was computed as the 1st derivative of vertical barbell position.  To prove the predictive accuracy of the model, the computed snatch was compared with the actual snatch performance of the 1RM test. | Snatch pull | r=0.99 (0.99;1.0); d=0.01 (-0.02;0.4); Diff (%)=0.18; SDD (%)=1.3; SDD (kg)=1.5 (1.0;3.1); p=0.706 | This study provides a new approach to estimate 1RM snatch performance in elite weightlifters using the snatch pull force-velocity relationship. The results demonstrate that the theoretical 1RM snatch-model accurately predicts 1RM snatch performance. |
| Sayers et al. (2018) | We tested 12 healthy, regular weight trainers on two separate occasions. On the first occasion 1RM bench press strength was recorded using standard procedures and recorded to the nearest 1 kg.  During the second data collection participants performed three repetitions of BP-T at loads representing 30, 40, 50, 60 and 70% of their 1RM. We then processed these load-velocity BP-T data using the techniques proposed by Jidovtseff et al. to determine which three load range (30–50% of 1RM, 40–60% of 1RM, 50–70% of 1RM) resulted in the most accurate prediction of 1RM bench press strength.  To record the vertical position of the bar a linear position transducer (LPT) (WS17KT) was installed on the Smith Machine’s pneumatic brake. The Vmean, and Vpeak and maximum bar acceleration were calculated from the first and second differentials of the linear transducer data. These data were then used to develop a linear regression model for the prediction of 1RM. | bench press throw | ICC measured versus predicted 1RM:  30-50%: Vmean=0.868 (0.558-0.966); Vpeak=0.967 (0.890-0.990); $R^{2}$=0.87; SEE=10.4  40-60%: Vmean=0.855(0.521-0.962); Vpeak= 0.680 (0.204-0.896); $R^{2}$=0.89; SEE=10.5  50-70%: Vmean=0.849 (0.506-0.960); Vpeak= 0.867 (0.604-0.960); $R^{2}$=0.85; SEE=11.6 | These preliminary findings indicate that exercise programmers working with normal healthy populations can accurately predict Smith Machine 1RM bench press strength using relatively light load Smith Machine BP-T testing, avoiding the need to expose their clients to potentially injurious loads. |
| Sigvaldsenet al. (2023) | Twenty-eight recreationally active subjects (25 ± 2 years, 178 ± 8 cm, 78 ± 9 kg) were assessed for load-velocity (L-V) relationship, 1RM, maximal isometric force (MIF), and maximal repetitions to failure (MRF) in a Smith Machine on three separated sessions. Linear regression was used for L-V relationship, MIF, and MRF to predict 1RM. All subjects underwent three test sessions (session 1, 2 and 3), and each test session was performed over two days (test day 1 and 2) with 48–72 hours in between. Barbell velocity was assessed by an encoder (GymAware). During the isometric strength tests, two force platforms (FDMax) were placed under the bench. They were used to calculate maximal isometric force (MIF). | prone bench pull, bench press | Validity of the 1RM predictions:  Bench press:  L-V vs. 1RM: r: 0.99 (0.98, 0.99); CV: 7.8 (5.8, 9.8); ES: 0.20 (-0.18, 0.57); MAE (kg): 5.5; SEE (kg): 4.2; p: <0.01  MIF vs. 1RM: r: 0.96 (0.92, 0.98); CV: 9.7 (7.1, 12.2); ES: 0.00 (-0.37, 0.37); MAE: 5.8; SEE: 7.7; p: 0.96  MRF vs. 1RM: r: 0.94 (0.88, 0.97); CV: 11.8 (8.7, 14.8); ES: 0.00 (-0.37, 0.37); MAE: 7.2; SEE: 9.5; p: 0.99  Prone bench pull:  L-V vs. 1RM: r: 0.98 (0.96, 0.99); CV: 4.5 (3.3, 5.6); ES: 0.07 (-0.30, 0.44); MAE: 2.8; SEE: 3.2; p: 0.05  MIF vs. 1RM: r: 0.83 (0.66, 0.92); CV: 13.2(9.8,16.7); ES: 0.01 (-0.36, 0.38); MAE: 7.4; SEE: 9.7; p: 0.96  MRF vs. 1RM: r: 0.93 (0.86, 0.97); CV: 8.5(6.2,10.7); ES: 0.00 (-0.37, 0.37); MAE: 4.6; SEE: 6.2; p: 0.99 | All 1RM prediction methods were highly comparable to the traditional 1RM test. However, given the high variability associated with individual predictions for each method, they cannot be used interchangeably. |
| Simonsen, et al. (2025) | A total of 22 healthy participants were recruited. The tested exercises included ankle, knee, and hip joint flexion and extension, as well as hip abduction, hip adduction, and leg press. Velocity during the concentric phase was measured using a linear transducer, and individual linear regression models were established using incremental submaximal loads (40–80% 1RM) and velocity to estimate the 1RM. Repetition-to-failure estimations of 1 RM were assessed with eleven different regression equations, among them the Lombardi equation. | Ankle dorsiflexion, Seated ankle plantarflexion, Standing ankle plantarflexion, Hip abduction, Hip adduction, Hip extension, Hip flexion, Knee extension, Knee flexion, Leg press | Ankle dorsiflexion (MV): NMAE: 31.1±0.31; r: 0.74±0.37; ICC: 0.55±0.42 (-0.34±0.43)  Seated ankle plantarflexion (MV): NMAE: 18.6±6.9; r: 0.73±0.27; ICC: 0.52±0.23 (-0.50±0.33)  Standing ankle plantarflexion (PV): NMAE: 35.2±30.1; r: 0.72±0.52; ICC: 0.35±0.39 (-0.76±0.25)  Hip abduction (MV): NMAE: 15.9±9.4; r: 0.62±0.64; ICC: 0.66±0.42 (-0.01±0.50)  Hip adduction (PV): NMAE: 17.0±5.0; r: 0.80±0.14; ICC: 0.72±0.15 (-0.12±0.36)  Hip extension (PV): NMAE: 22.9±5.6; r: 0.28±0.64; ICC: 0.48±0.28 (-0.45±0.23)  Hip flexion (PV): NMAE: 18.3±10.2; r: 0.65±0.31; ICC: 0.56±0.29 (-0.30±0.39)  Knee extension (MV): NMAE: 11.3±6.1; r: 0.84±0.17; ICC: 0.84±0.14 (0.25±0.46)  Knee flexion (MV): NMAE: 21.7±0.10; r: 0.66±0.42; ICC: 0.52±0.33 (-0.37±0.32)  Leg press (MV): NMAE: 8.6±7.7; r: 0.88±0.24; ICC: 0.87±0.20 (0.43±0.57) | The load-velocity relationship proved inadequate for predicting 1RM in lower-extremity single-joint exercises. However, the Lombardi estimation equations showcased favorable predictive performance with a consistently low average NMAE across all exercises studied. |
| Soriano, et al. (2023) | Twenty-seven weightlifters (16 men and 11 women) participated. The first session was used to test the overhead press 1RM.  The second session consisted of an incremental loading test of three repetitions against five different loads (30, 45, 60, 75, and 90 % 1RM) during the free-weight overhead press, performed with maximum intent. The mean velocity of the barbell was measured and registered using a validated LPT (Chronojump). Three methods, which only differed in the loads used to determine the individualized load–velocity relation ships, were employed to estimate the overhead press 1RM: (I) multiple-point–fiveloads (i.e. 30, 45, 60, 75, 90 %), (II) two-point45−75 – two loads (45 and 75 % 1RM), and (III) two-point45−90 – two loads (45 and 90 % 1RM). | overhead press | Characteristics of the loads used for the modelling of the individualized load-velocity relationships:  30%: MV (m*s-1): 1.3∓0.2; ICC: 0.88 (0.76-0.94); CV: 4.5 (3.6-6.2)  45%: MV: 1.1±0.1 ; ICC: 0.86 (0.75-0.93); CV: 4.8 (3.8-6.5)  60%: MV: 0.8±0.1; ICC: 0.81 (0.62-0.91); CV: 5.9 (4.6-8.1)  75%: MV: 0.6±0.1; ICC: 0.93 (0.85-0.97); CV: 4.1 (3.2-5.6)  90%: MV: 0.4±0.1; ICC: 0.9 (0.78-0.95); CV: 9.0 (7.1-12.3)  Multiple-Point:  p=0.004; ES= -0.12; r=0.982  Two-Point45-75:  p=0.296; ES= -0.07; r=0.941  Two-Pints 45-90:  p=0.164; ES= -0.05; r=0.982 | These results suggest that the Multiple Point and Two-Point45−90 velocity-based methods might be viable tools to predict the overhead press of 1RM in weightlifters, but practitioners are encouraged to use the direct 1RM for a more accurate prescription of the training loads. |
| Warneke et al. (2024) | This article explored the random measurement error arising from repeated testing (repeatability) and the agreement between two common sensors (vMaxPro and TENDO) within one repetition, using minimal velocity thresholds as well as the velocity = 0 m/s method. Furthermore, agreement analyses were applied to the estimated and measured 1RM in 25 young elite male soccer athletes.  The test protocol was divided into 2 testing days. Test day 1 consisted of 1RMinthesquat. One week after the 1RM testing, two frequently used velocity sensors were attached to a straight bar. The participants were instructed to perform two trials of the parallel back squat as fast as possible with a prescribed percentage of their respective parallel 1RM back squat.  Following the standardized warm-up used in the 1RM session, load–velocity profiling was conducted using loads corresponding to 20%, 40%, 60%, 80%, and 90% of the previously determined 1RM. Two concentric actions were recorded for each load, and the one with the highest absolute mean velocity value was selected for further analysis. The rest interval between each trial was 2 min. | back squat | Repeatability of the testing, providing the relative reliability and the systematic and random bias resulting from repeated measurements:  MVvMaxPro T1 vs. MVvMaxPro T2:  20%: ICC: 0.68 (0.51-0.89); Concordance r: 0.67 (0.41-0.83); MAE: 0.06; MAPE: 5.3; Mean diff. (p-value): 0.04 (0.008)  40%: ICC: 0.78 (0.61-1.00); r: 0.77 (0.58-0.89); MAE: 0.04; MAPE: 3.8; Mean diff. (p-value): 0.02 (0.025)  60%: ICC: 0.79 (0.61-1.00); r: 0.78 (0.58-0.90); MAE: 0.03; MAPE: 4.1; Mean diff. (p-value): 0.01 (0.18)  80%: ICC: 0.80 (0.70-0.93); r: 0.80 (0.61-0.90); MAE: 0.22; MAPE: 3.5; Mean diff. (p-value): 0.009 (0.13)  90%: ICC: 0.81 (0.56-1.00); r: 0.80 (0.61-0.91); MAE: 0.03; MAPE: 5.3; Mean diff. (p-value): 0.01 (0.22)  MVTendo T1 vs. MVTendo T2:  20%: ICC: 0.66 (0.50-0.86); r: 0.65 (0.39-0.82); MAE: 0.07; MAPE: 6.5; Mean diff. (p-value): 0.05 (0.006)  40%: ICC: 0.74 (0.48-1.00); r: 0.73 (0.49-0.87); MAE: 0.04; MAPE: 4.2; Mean diff. (p-value): 0.02 (0.233)  60%: ICC: 0.70 (0.42-1.00); r: 0.69 (0.44-0.85); MAE: 0.04; MAPE: 4.4; Mean diff. (p-value): 0.01 (0.35)  80%: ICC: 0.77 (0.57-1.00); r: 0.76 (0.53-0.89); MAE: 0.03; MAPE: 4.7; Mean diff. (p-value): 0.002 (0.86)  90%: ICC: 0.74 (0.55-1.00); r: 0.73 (0.49-0.87); MAE: 0.05; MAPE: 7.5; Mean diff. (p-value): 0.02 (0.15)  Agreement in the velocity determination between the VMaxPro and the Tendo device for the first and second trials and the best results extracted out of both trials:  vMaxPro1 vs. Tendo1:  20%: ICC: 0.37 (0.26-0.50); r: 0.36 (0.11-0.57); MAE: 0.13; MAPE: 11.0; Mean diff. (p-value): 0.09 (<0.001)*  40%: ICC: 0.59 (0.46-0.75); r: 0.58 (0.38-0.72); MAE: 0.09; MAPE: 8.7; Mean diff. (p-value): 0.08 (<0.001)*  60%: ICC: 0.53 (0.32-0.80); r: 0.52 (0.30-0.69); MAE: 0.07; MAPE: 9.0; Mean diff. (p-value): 0.07 (<0.001)*  80%: ICC: 0.28 (-0.06-0.60); r: 0.28 (-0.67-0.58); MAE: 0.05; MAPE: 8.5; Mean diff. (p-value): 0.03 (0.033)  90%: ICC: 0.80 (0.65-1.00); r: 0.80 (0.62-0.90); MAE: 0.04; MAPE: 6.7; Mean diff. (p-value): 0.03 (<0.001)*  vMaxPro2 vs. Tendo2:  20%: ICC: 0.53 (0.40-0.70); r: 0.52 (0.32-0.68); MAE: 0.11; MAPE: 9.8; Mean diff. (p-value): 0.11 (<0.001)*  40%: ICC: 0.62 (0.48-0.80); r: 0.61 (0.39-0.76); MAE: 0.08; MAPE: 7.8; Mean diff. (p-value): 0.07 (<0.001)*  60%: ICC: 0.65 (0.49-0.86); r: 0.64 (0.43-0.78); MAE: 0.05; MAPE: 6.2; Mean diff. (p-value): 0.05 (<0.001)*  80%: ICC: 0.49 (0.27-0.73); r: 0.48 (0.18-0.69); MAE: 0.05; MAPE: 6.7; Mean diff. (p-value): 0.02 (0.079)  90%: ICC: 0.80 (0.59-1.00); r: 0.79 (0.61-0.89); MAE: 0.03; MAPE: 5.6; Mean diff. (p-value): 0.02 (0.013)  vMaxPro best vs. Tendo best:  20%: ICC: 0.52 (0.37-0.70); r: 0.51 (0.30-0.67); MAE: 0.11; MAPE: 9.4; Mean diff. (p-value): 0.10 (<0.001)*  40%: ICC: 0.60 (0.45-0.78); r: 0.59 (0.37-0.75); MAE: 0.08; MAPE: 8.3; Mean diff. (p-value): 0.07 (<0.001)*  60%: ICC: 0.50 (0.25-0.83); r: 0.49 (0.24-0.68); MAE: 0.06; MAPE: 7.3; Mean diff. (p-value): 0.06 (<0.001)*  80%: ICC: 0.40 (0.15-0.68); r: 0.39 (0.09-0.63); MAE: 0.05; MAPE: 7.3; Mean diff. (p-value): 0.03 (0.026)  90%: ICC: 0.76 (0.58-1.00); r: 0.75 (0.55-0.87); MAE: 0.04; MAPE: 7.5; Mean diff. (p-value): 0.03 (<0.001)*  Agreement between measured 1RM and estimated 1RM with and without RPE (vMaxPro):  vMaxPro: ICC: 0.82 (0.70-0.98); r: 0.81 (0.65-0.90); MAE: 7.72; MAPE: 7.15; MPE: 18.3; Systematic bias: 6.60 (<0.001)*  vMaxProRPE: ICC: 0.93 (0.87-0.99); r: 0.92 (0.84-0.97); MAE: 4.32; MAPE: 4.12; MPE: 17.0; Systematic bias: 2.24 (0.08)  Tendo V0: ICC: 0.16 (0.11-0.22); r: 0.15 (0.07-0.23); MAE: 64.55; MAPE: 60.74; MPE: 93.22; Systematic bias: 64.6 (<0.001)*  Tendo V0.3: ICC: 0.40 (0.31-0.52); r: 0.40 (0.23-0.54); MAE: 29.35; MAPE: 27.53; MPE: 46.6; Systematic bias: 29.4 (<0.001)*  Tendo V0.5: ICC: 0.85 (0.79-0.93); r: 0.84 (0.71-0.92); MAE: 8.72; MAPE: 8.30; MPE: 15.7; Systematic bias: 5.89 (0.002)*  vMaxPro V0: ICC: 0.12 (0.08-0.18); r: 0.12 (0.05-0.19); MAE: 70.31; MAPE: 66.71; MPE: 96.23; Systematic bias: 70.3 (<0.001)*  vMaxPro V0.3: ICC: 0.36 (0.63-0.48); r: 0.35 (0.19-0.49); MAE: 30.58; MAPE: 29.12; MPE: 45.01; Systematic bias: 30.6 (<0.001)*  vMaxPro V0.5: ICC: 0.89 (0.83-0.96); r: 0.88 (0.76-0.95); MAE: 6.59; MAPE: 6.50; MPE: 22.45; Systematic bias: 4.10 (0.005) | Almost all estimations systematically over/ underestimated the measured 1RM, with a random scattering between 4.12% and 71.6%, depending on the velocity threshold used. The precision of the estimation is unacceptable in free-weight exercises when compared to direct measurement of 1RM in the presented data. |
| Miller et al. (2018) | The aim of this investigation was to determine the test-retest reliability for barbell velocity during the bench press (BP) and weight velocity during the 2 leg press (2LP) for loads corresponding to 20–80% of 1 repetition maximum (1RM). Forty recreationally active individuals (22.6 6 2.5 years) with a 1RM BP and 2LP of 66.8 +- 32.4 kg and 189.5 +- 49 kg, respectively, volunteered for this study.  1 familiarization visit preceding 3 testing visits, which encompassed 1RM determination and 2 days of velocity testing. Forty-eight hours after 1RM testing, the subjects performed 1 repetition at 20, 30, 40, 50, 60, 70, and 80% of their 1RM for each exercise in randomized order. Subjects returned to the laboratory 1 week later to perform the velocity assessment again in randomized order.  Testing tool: TFSPA (Tendo Fitrodyne Sports Power Analyzer) | bench press & 2 leg press | Reliability measures of movement velocity across each percent 1RM for bench press (BP) and 2 leg press (2LP)  20%:  ICC: BP: 0.74, 2LP: 0.98  SEM (%): BP: 6.5, 2LP: 2.8  SEM (m*s-1): BP: 0.12, 2LP: 0.03  30%:  ICC: BP: 0.81, 2LP: 0.95  SEM (%): BP: 4.8, 2LP: 4.4  SEM (m*s-1): BP: 0.07, 2LP: 0.04  40%:  ICC: BP: 0.96, 2LP: 0.89  SEM (%): BP: 3.0, 2LP: 7.2  SEM (m*s-1): BP: 0.03, 2LP: 0.06  50%:  ICC: BP: 0.98, 2LP: 0.88  SEM (%): BP: 4.2, 2LP: 7.1  SEM (m*s-1): BP: 0.04, 2LP: 0.05  60%:  ICC: BP: 0.97, 2LP: 0.86  SEM (%): BP: 5.9, 2LP: 6.9  SEM (m*s-1): BP: 0.05, 2LP: 0.04  70%:  ICC: BP: 0.74, 2LP: 0.81  SEM (%): BP: 9.3, 2LP: 6.5  SEM (m*s-1): BP: 0.04, 2LP: 0.03  80%:  ICC: BP: 0.56, 2LP: 0.78  SEM (%): BP: 18.1, 2LP: 5.7  SEM (m*s-1): BP: 0.07, 2LP: 0.03 | The results of this study indicate that the TFSPA (Tendo Fitrodyne Sports Power Analyzer) (can provide reliable assessments when measuring velocity during free weight and machine-based exercise. This study demonstrated that measurement error can be reduced during machine-based exercise (2LP), across a spectrum of loads when compared with a common free weight exercise (BP).  When evaluating velocity for free weight or machine-based exercises, future research can expect reliable measures with minimal error between these intensities; however, the reliability and expected error increases at intensities outside of this range (20% and 70–80%) especially in free weight exercises. |
| van den Tillaar et al. (2024) | The aim of this study was to compare barbell velocities at different intensities and estimated 1-RM with actual 1-RM measured with different measuring tools in bench presses and squats. Fourteen resistance-trained athletes (eight men, six women, age 28.1 ± 7.5 years) performed bench presses and squats at five loads varying from 45 to 85% of one repetition maximum (1-RM), together with 1-RM testing, while measuring mean, mean propulsive, and peak barbell velocity with six different commercially used inertial measurement units (IMUs) and linear encoder software systems attached to the barbell. The 1-RM was also estimated based upon the load–velocity regression, which was compared with the actual 1-RM in the bench press and squat exercises.  Measuring tools were GymAware, Musclelab, Vmaxpro, Speed4lifts and PUSH band | bench press & back squat | back squat  Musclelab:  45%: CV/MPV: 3.7, ICC (95% CI) MPV: 0.88 (0.66-0.96), CV/MV: 1.3, ICC (95% CI) MV: 0.99 (0.98-0.99), CV/PV: 2.1, ICC (95% CI) PV: 0.98 (0.94-0.99)  55%: CV/MPV: 3.3, ICC (95% CI) MPV: 0.92(0.77–0.98), CV/MV: 2.3, ICC (95% CI) MV: 0.99(0.98–0.99), CV/PV: 2.8, ICC (95% CI) PV: 0.98(0.94–0.99)  65%: CV/MPV: 4.2, ICC (95% CI) MPV: 0.88(0.67–0.96), CV/MV: 2.0, ICC (95% CI) MV: 0.98(0.93–0.99), CV/PV: 3.3, ICC (95% CI) PV: 0.94(0.82–0.98)  75%: CV/MPV: 4.5, ICC (95% CI) MPV: 0.91(0.74–0.97), CV/MV: 3.3, ICC (95% CI) MV: 0.98(0.93–0.99), CV/PV: 1.9, ICC (95% CI) PV: 0.98(0.94–0.99)  85%: CV/MPV: 9.4, ICC (95% CI) MPV: 0.80(0.48–0.93), CV/MV: 9.2, ICC (95% CI) MV: 0.97(0.92–0.99), CV/PV: 5.2, ICC (95% CI) PV: 0.89(0.70–0.97)  100%: CV/MPV: 13.2, ICC (95% CI) MPV:0.73(0.34–0.91), CV/MV: 12.5, ICC (95% CI) MV: 0.92(0.75–0.98), CV/PV: 12.3, ICC (95% CI) PV: 0.79(0.45–0.93)  Speed4lifts:  45%: CV/MPV: 3.9, ICC (95% CI) MPV: 0.91(0.75–0.97),CV/PV: 1.3, ICC (95% CI) PV: 0.99(0.98–0.99)  55%: CV/MPV: 4.6, ICC (95% CI) MPV: 0.83(0.56–0.95),CV/PV: 7.1, ICC (95% CI) PV: 0.46(−0.09–0.80)  65%: CV/MPV: 3.3, ICC (95% CI) MPV: 0.93(0.8–0.98),CV/PV: 6.6, ICC (95% CI) PV: 0.80(0.48–0.94)  75%:CV/MPV: 6.4, ICC (95% CI) MPV: 0.83(0.56–0.95),CV/PV: 5.4, ICC (95% CI) PV: 0.76(0.37–0.92)  85%: CV/MPV: 12.9, ICC (95% CI) MPV: 0.63(0.16–0.87),CV/PV: 15.3, ICC (95% CI) PV:0.00(−0.52–0.53)  100%: CV/MPV: 7.3, ICC (95% CI) MPV: 0.94(0.81–0.98),CV/PV: 7.8, ICC (95% CI) PV: 0.92(0.77–0.98)  Vmaxpro:  45%:CV/MV: 8.2, ICC (95% CI) MV: 0.97(0.92–0.99), CV/PV: 2.3, ICC (95% CI) PV: 0.98(0.93–0.99)  55%: CV/MV: 2.6, ICC (95% CI) MV: 0.96(0.87–0.99), CV/PV: 3.4, ICC (95% CI) PV: 0.94(0.81–0.98)  65%: CV/MV: 4.6, ICC (95% CI) MV: 0.88(0.65–0.96), CV/PV: 4.8, ICC (95% CI) PV:0.89(0.69–0.97)  75%: CV/MV: 3.9, ICC (95% CI) MV: 0.98(0.94–0.99), CV/PV: 3.6, ICC (95% CI) PV: 0.93(0.78–0.97)  85%: CV/MV: 10.4, ICC (95% CI) MV: 0.89(0.67–0.96), CV/PV: 2.8, ICC (95% CI) PV: 0.97(0.91–0.99)  100%: CV/MV: 12.5, ICC (95% CI) MV: 0.71(0.29–0.90), CV/PV: 15.9, ICC (95% CI) PV: 0.69(0.25–0.89)  GymAware:  45%:CV/MV: 13.2, ICC (95% CI) MV: 0.96(0.87–0.99), CV/PV: 2.6, ICC (95% CI) PV: 0.97(0.91–0.99)  55%: CV/MV: 2.4, ICC (95% CI) MV: 0.97(0.90–0.99), CV/PV: 3.0, ICC (95% CI) PV: 0.94(0.81–0.98)  65%: CV/MV: 3.4, ICC (95% CI) MV: 0.93(0.79–0.98), CV/PV: 3.5, ICC (95% CI) PV: 0.94(0.80–0.98)  75%: CV/MV: 4.1, ICC (95% CI) MV: 0.97(0.92–0.99), CV/PV: 8.6, ICC (95% CI) PV: 0.72(0.31–0.90)  85%: CV/MV: 3.5, ICC (95% CI) MV: 0.97(0.89–0.99), CV/PV: 3.1, ICC (95% CI) PV: 0.96(0.89–0.99)  100%: CV/MV: 8.7, ICC (95% CI) MV: 0.91(0.75–0.97), CV/PV: 5.8, ICC (95% CI) PV: 0.96(0.87–0.99)  PUSH:  45%: CV/MV: 6.3, ICC (95% CI) MV: 0.80(0.48–0.94)  55%: CV/MV: 2.2, ICC (95% CI) MV: 0.98(0.93–0.99)  65%: CV/MV: 5.3, ICC (95% CI) MV: 0.87(0.64–0.96)  75%: CV/MV: 8.7, ICC (95% CI) MV: 0.92(0.75–0.97)  85%: CV/MV: 5.3, ICC (95% CI) MV: 0.93(0.79–0.98)  100%: CV/MV: 11.6, ICC (95% CI) MV: 0.80(0.47–0.94)  bench press:  Musclelab:  45%: CV/MPV: 4,6, ICC (95% CI) MPV: 0.93(0.81–0.98), CV/MV: 3.9, ICC (95% CI) MV: 0.87(0.66–0.95), CV/PV: 4.1, ICC (95% CI) PV: 0.87(0.64–0.96)  55%: CV/MPV: 5.6, ICC (95% CI) MPV: 0.93(0.81–0.98), CV/MV: 2.7, ICC (95% CI) MV: 0.96(0.89–0.99), CV/PV: 3.7, ICC (95% CI) PV: 0.95(0.84–0.98)  65%: CV/MPV: 3.6, ICC (95% CI) MPV: 0.96(0.88–0.99), CV/MV: 6.0, ICC (95% CI) MV: 0.91(0.75–0.97), CV/PV: 4.1, ICC (95% CI) PV: 0.96(0.89–0.99)  75%: CV/MPV: 4.0, ICC (95% CI) MPV: 0.96(0.89–0.99), CV/MV: 3.4, ICC (95% CI) MV: 0.97(0.91–0.99), CV/PV: 4.6, ICC (95% CI) PV: 0.95(0.85–0.98)  85%: CV/MPV: 7.8, ICC (95% CI) MPV: 0.94(0.83–0.98), CV/MV: 10.8, ICC (95% CI) MV: 0.87(0.64–0.96), CV/PV: 5.3, ICC (95% CI) PV: 0.97(0.90–0.99)  100%: CV/MPV: 15.2, ICC (95% CI) MPV: 0.81(0.52–0.93), CV/MV: 16.1, ICC (95% CI) MV: 0.65(0.2–0.87), CV/PV: 12.8, ICC (95% CI) PV: 0.74(0.36–0.91)  Speed4lifts:  45%: CV/MPV: 9.7, ICC (95% CI) MPV: 0.79(0.48–0.92),CV/PV: 11.7, ICC (95% CI) PV: 0.36(−0.19–0.74)  55%: CV/MPV: 9.7, ICC (95% CI) MPV:0.73(0.36–0.90),CV/PV: 14.1, ICC (95% CI) PV: 0.41(−0.13–0.77)  65%: CV/MPV: 4.5, ICC (95% CI) MPV: 0.95(0.85–0.98),CV/PV: 2.9, ICC (95% CI) PV: 0.95(0.84–0.98)  75%:CV/MPV: 8.2, ICC (95% CI) MPV: 0.90(0.74–0.97),CV/PV: 10.8, ICC (95% CI) PV: 0.84(0.58–0.95)  85%: CV/MPV: 6.4, ICC (95% CI) MPV: 0.96(0.88–0.99),CV/PV: 19.7, ICC (95% CI) PV:0.60(0.12–0.85)  100%: CV/MPV: 10.3, ICC (95% CI) MPV: 0.89(0.7–0.96),CV/PV: 10.3, ICC (95% CI) PV: 0.82(0.52–0.94)  Vmaxpro:  45%:CV/MV: 3.8, ICC (95% CI) MV: 0.91(0.74–0.97), CV/PV: 4.1, ICC (95% CI) PV: 0.89(0.68–0.96)  55%: CV/MV: 3.5, ICC (95% CI) MV: 0.94(0.82–0.98), CV/PV: 2.8, ICC (95% CI) PV: 0.96(0.89–0.99)  65%: CV/MV: 3.8, ICC (95% CI) MV: 0.97(0.90–0.99), CV/PV: 7.1, ICC (95% CI) PV: 0.91(0.74–0.97)  75%: CV/MV: 4.7, ICC (95% CI) MV: 0.95(0.87–0.99), CV/PV: 6.6, ICC (95% CI) PV: 0.90(0.72–0.97)  85%: CV/MV: 7.0, ICC (95% CI) MV: 0.95(0.84–0.98), CV/PV: 5.4, ICC (95% CI) PV: 0.97(0.89–0.99)  100%: CV/MV: 15.4, ICC (95% CI) MV: 0.78(0.45–0.92), CV/PV: 11.6, ICC (95% CI) PV: 0.78(0.43–0.92)  GymAware:  45%:CV/MV: 4.8 , ICC (95% CI) MV: 0.82(0.53–0.94), CV/PV: 3.2, ICC (95% CI) PV: 0.93(0.80–0.98)  55%: CV/MV: 5.2, ICC (95% CI) MV:0.89(0.69–0.96), CV/PV: 4.0, ICC (95% CI) PV: 0.95(0.84–0.98)  65%: CV/MV: 6.1, ICC (95% CI) MV: 0.92(0.76–0.97), CV/PV: 3.9, ICC (95% CI) PV:0.94(0.81–0.98)  75%: CV/MV: 5.6, ICC (95% CI) MV: 0.93(0.80–0.98), CV/PV: 6.0, ICC (95% CI) PV: 0.93(0.79–0.98)  85%: CV/MV: 8.9, ICC (95% CI) MV: 0.91(0.75–0.97), CV/PV: 5.5, ICC (95% CI) PV: 0.96(0.88–0.99)  100%: CV/MV: 18.4, ICC (95% CI) MV:0.60(0.13–0.85), CV/PV: 8.6, ICC (95% CI) PV: 0.79(0.27–0.89)  PUSH:  45%: CV/MV: 4.6, ICC (95% CI) MV: 0.85(0.59–0.95)  55%: CV/MV: 15.1, ICC (95% CI) MV:0.82(0.53–0.94)  65%: CV/MV: 9.8, ICC (95% CI) MV: 0.82(0.54–0.94)  75%: CV/MV: 11.3, ICC (95% CI) MV: 0.76(0.41–0.92)  85%: CV/MV: 35.1, ICC (95% CI) MV: 0.00(−0.51–0.51)  100%: CV/MV: 26.9, ICC (95% CI) MV: 0.53(0.03–0.82) | GymAware revealed the highest reliability along with minimal bias, while Musclelab and Vmaxpro showed moderate reliability with some variability at higher loads. Speed4lifts and Push band indicated greater variability, specifically at higher intensities.  Significant discrepancies were found between actual and estimated 1-RM values, with Speed4lifts and Musclelab notably underestimating 1-RM. |
| Jiménez-Alonso et al. (2022) | This study aimed to determine whether the verbal provision of velocity performance feedback during the free-weight bench press (BP) exercise influences (a) the within-session reliability and magnitude of mean concentric velocity (MCV) values recorded against a range of submaximal loads and (b) the accuracy of the individualized load-velocity profile to estimate the BP 1 repetition maximum (1RM). Fifteen men (BP 1RM relative to bodymass, age =20.5 +- 3.0 years [range: 18–29 years]) performed an incremental loading test until reaching the 1RM on 2 separate sessions.Subjects received verbal velocity performance feedback in 1 session (knowledge of results [KR]), and no KR was provided in another session (Control). A linear velocity transducer was used to collect the MCV against 4 loads (40-55-70-85% 1RM), and the BP 1RM was estimated from the individualized load-velocity relationship modeled through the multiple-point (40-55-70-85% 1RM) and 2-point methods (40 85%1RM). | bench press | Within-session reliability of mean concentric velocity values obtained during the bench press exercise performed with knowledge of results (KR) and without KR (control) at different loads:  40% 1RM:  KR: ICC:0.91 (0.76–0.97)  Control: ICC: 0.88 (0.69–0.96)  55% 1RM:  KR: ICC:0.79 (0.49–0.92)  Control: ICC: 0.89 (0.71–0.96)  70% 1RM:  KR: ICC:0.86 (0.64–0.95)  Control: ICC: 0.81 (0.53–0.93)  Control & KR: r=0.96  KR:  Differences (Actual 1 RM - multiple-point 1RM) (kg) vs. mean actual and multiple-point 1RM (kg): R2= 0.17  Differences (Actual 1 RM - 2-point 1RM) (kg) vs. mean actual and 2-point 1RM (kg): R^2^= 0.09  Control:  Differences (Actual 1 RM - multiple-point 1RM) (kg) vs. mean actual and multiple-point 1RM (kg): R^2^= 0.2  Differences (Actual 1 RM - 2-point 1RM) (kg) vs. mean actual and 2-point 1RM (kg): R^2^= 0.02 | These findings encourage practitioners to ver bally provide velocity performance feedback after each repe tition during BP testing procedures. Importantly, our results showforthefirst time that the recording of MCV againstonly 2 loads ( 40% 1RMand 85%1RM;i.e.,2-point method) can provide an accurate estimation of the 1RM during the free-weight BP exercise. |
| Kilgallon et al. (2022) | The purpose of this study was to examine the reliability of load-velocity profiles (LVPs) and validity of 1-repetition maximum (1-RM) prediction methods in the back-squat using the novel Vitruve linear position transducer (LPT). Twenty-five men (25.2 +- 2.8years) completed a back-squat 1-RM assessment followed by 2LVP trials using five incremental loads (20%–40%–60%–80%–90% 1-RM). Mean propulsive velocity (MPV), mean velocity (MV) and peak velocity (PV) were measured via a (LPT).  Measuring tool was the Vitruve (Vitruve encoder; Madrid, Spain). | back squat | Paired samples t test result for Velocity Measures and 1RM Predictions.  MPV, mean (SD), m*s-1:  20% 1RM: p=0.19  40% 1RM: p=0.91  60% 1RM: p=0.14  80% 1RM: p=0.82  90% 1RM: p=0.54  MV, mean (SD), m*s-1:  20% 1RM: p=0.33  40% 1RM: p=0.69  60% 1RM: p=0.02  80% 1RM: p=0.92  90% 1RM: p=0.28  PV, mean (SD), m*s-1:  20% 1RM: p=0.31  40% 1RM: p=0.73  60% 1RM: p=0.02  80% 1RM: p=0.85  90% 1RM: p=0.84  Linear regression, mean (SD), kg  MPV: p= 0.10  MV: p=0.21  PV: p=0.29  Polynominal regression, mean (SD), kg  MPV: p= 0.25  MV: p=0.52  PV: p=0.31  Smallest detectable difference of velocity measures at  20% 1RM: MPV, m*s-1: 0.07; MV, m*s-1: 0.10; PV, m*s-1: 0.10  40% 1RM: MPV, m*s-1: 0.06; MV, m*s-1: 0.08; PV, m*s-1: 0.07  60% 1RM: MPV, m*s-1: 0.05; MV, m*s-1: 0.08; PV, m*s-1: 0.05  80% 1RM: MPV, m*s-1: 0.05; MV, m*s-1: 0.08; PV, m*s-1: 0.05  90% 1RM: MPV, m*s-1: 0.05; MV, m*s-1: 0.09; PV, m*s-1: 0.05 | Prediction methods may not be sensitive enough to replace direct assessment of 1-RM. However, LVPs using linear regression and MPV or MV may still pro vide practical information regarding an individual’s performance potential. The Vitruve provides reliable LVPs for MPV, MV and PV in the back-squat using strength-trained males. Linear regression is superior for 1-RM prediction. |
| Macarilla et al. (2022) | This study examined the accuracy of predicting a free-weight back squat and a bench press one repetition maximum (1RM) using both 2- and 4-point submaximal average concentric velocity (ACV) methods. Seventeen resistance trained men (23.47±4.23) performed a warm-up and a 1RM test on the squat and bench press with ACV assessed on all repetitions. The ACVs during the warm-up closest to 1.0 and 0.5m.s-1 were used in the 2-point linear regression forecast of the 1RM and the ACVs established at loads closest to 20, 50, 70, and 80% of the 1RM were used in the 4-point 1RM prediction. | back squat & bench press | Back squat:  2-Point: R^2^= 0.88, ICC: 0.7284 (-0.0672 -0.9341), ES: 1.89, SEM: 9.1524  4-point: R^2^= 0.76, ICC: 0.6058 (-0.0891 -0.8865), ES: 1.63, SEM: 10.4215  Bench press:  2-Point: R^2^= 0.87, ICC: 0.8692 (0.3736 - 0.9624) , ES: 0.83 , SEM: 7.1427  4-point: R^2^=0.87, ICC: 0.8924 (0.6930 - 0.9624), ES: 0.47, SEM: 7.7553 | They recommend that athletes who wish to predict 1RM from submaximal velocities perform 2 single repetition sets of maximally intended velocity at the submaximal loads and use the higher velocity in the 1RM forecast. While submaximal velocity prediction may be attractive to coaches and athletes since it is both less fatiguing and time consuming than performing a true 1RM, based upon the present findings we advise against using this prediction to inform training decisions. |
| Pestana-Melero et al. (2017) | This study aimed to compare the between-session reliability of the load-velocity relationship between (1) linear vs. polynomial regression models, (2) concentric-only vs. eccentric concentric bench press variants, as well as (3) the within-participants vs. the between participants variability of the velocity attained at each percentage of the one-repetition maximum (%1RM). The load-velocity relationship of 30 men (age: 21.2±3.8 y) were evaluated by means of linear and polynomial regression models in the concentric-only and eccentric concentric bench press variants in a Smith Machine. | bench press | individual load-velocity relationship in concentric-only bench press  linear regression: R^2^= 0.988 [0.970, 0.998]  polynominal regression: R^2^=0.995 [0.984, 0.999]  in eccentric-concentric bench press:  linear regression: R^2^= 0.990 [0.964, 0.998]  polynominal regression: R^2^= 0.995 [0.985, 1.000]  Intraclass correlation coefficients with 95% confidence intervals obtained at each relative load for each pench press variant and regression model:  concentric-only bench press:  20%: linear:0.78 (0.59, 0.89); polynominal:0.69 (0.45, 0.84)  25%: linear: 0.78 (0.60, 0.89); polynominal:0.73 (0.50, 0.86)  30%: linear: 0.79 (0.60, 0.89); polynominal:0.76 (0.55, 0.88)  35%: linear: 0.79 (0.60, 0.89); polynominal: 0.78 (0.59, 0.89)  40%: linear: 0.79 (0.60, 0.89); polynominal: 0.80 (0.63, 0.90)  45%: linear: 0.79 (0.60, 0.89); polynominal: 0.81 (0.65, 0.91)  50%: linear: 0.79 (0.60, 0.89); polynominal: 0.81 (0.65, 0.91)  55%: linear: 0.78 (0.59, 0.89); polynominal: 0.81 (0.64, 0.90)  60%: linear: 0.78 (0.58, 0.89); polynominal: 0.80 (0.62, 0.90)  65%: linear: 0.77 (0.57, 0.88); polynominal: 0.78 (0.59, 0.89)  70%: linear: 0.75 (0.54, 0.87); polynominal: 0.76 (0.56, 0.88)  75%: linear:0.73 (0.50, 0.86); polynominal: 0.74 (0.52, 0.87)  80%: linear: 0.70 (0.46, 0.84); polynominal:0.71 (0.47, 0.85)  85%: linear: 0.67 (0.41, 0.83); polynominal:0.66 (0.40, 0.82)  90%: linear: 0.65 (0.38, 0.82); polynominal: 0.59 (0.30, 0.78)  95%: linear:0.64 (0.36, 0.81); polynominal:0.51 (0.19, 0.73)  100%: linear: 0.64 (0.36, 0.81); polynominal:0.47 (0.14, 0.71)  All: linear: 0.78 (0.64, 0.79); polynominal: 0.76 (0.47, 0.81)  eccentric-concentric bench press:  20%: linear: 0.76 (0.56, 0.88); polynominal: 0.64 (0.37, 0.81)  25%: linear: 0.76 (0.56, 0.88); polynominal: 0.68 (0.42, 0.83)  30%: linear: 0.76 (0.56, 0.88); polynominal: 0.72 (0.49, 0.86)  35%: linear: 0.76 (0.56, 0.88); polynominal: 0.77 (0.57, 0.88)  40%: linear: 0.76 (0.56, 0.88); polynominal: 0.80 (0.62, 0.90)  45%: linear: 0.76 (0.55, 0.88); polynominal: 0.82 (0.65, 0.91)  50%: linear: 0.75 (0.54, 0.87); polynominal: 0.82 (0.66, 0.91)  55%: linear: 0.74 (0.53, 0.87); polynominal: 0.81 (0.64, 0.91)  60%: linear: 0.73 (0.51, 0.86); polynominal: 0.80 (0.62, 0.90)  65%: linear: 0.71 (0.48, 0.85); polynominal: 0.78 (0.59, 0.89)  70%: linear: 0.69 (0.45, 0.84); polynominal: 0.76 (0.55, 0.88)  75%: linear: 0.66 (0.40, 0.82); polynominal: 0.73 (0.50, 0.86)  80%: linear: 0.63 (0.36, 0.81); polynominal: 0.68 (0.44, 0.84)  85%: linear: 0.60 (0.31, 0.79); polynominal: 0.61 (0.32, 0.79)  90%: linear: 0.57 (0.27, 0.77); polynominal: 0.45 (0.11, 0.69)  95%: linear: 0.55 (0.25, 0.76); polynominal: 0.14 (-0.23, 0.47)  100%: linear: 0.54 (0.24, 0.75); polynominal: -0.05 (-0.40, 0.31)  All%: linear: 0.73 (0.54, 0.76); polynominal: 0.73 (-0.05, 0.82) | They recommend the individual determination of the load-velocity relationship by a linear regression model to accurately monitor and prescribe the relative load (%1RM) in both variants of the Smith Machine bench press exercise. |
| Thompson et al. (2021) | The study aim was to compare different predictive models in one repetition maximum (1RM) estimation from load-velocity profile (LVP) data. Fourteen strength-trained men (26.0 ± 3.8 years) underwent initial 1RMs in the free-weight back squat, followed by two LVPs, over three sessions. Pro files were constructed via a combined method (jump squat (0 load, 30–60% 1RM) + back squat (70–100% 1RM)) or back squat only (0 load, 30–100% 1RM) in 10% increments. Measurement was conducted via 2D video analysis (iPhone 77, iOS 14.4.4, Apple, Cupertino, CA, USA) and the Gymaware Linear Position Transducer (Version 2.9.4, Kinetic, Canberra, Australia) was used to check displacement of the barbell. | back squat | Combined (quadratic 7): 95%CI (kg):120.17–192.51, r= 0.990, SEE (kg): 2.81, p= 0.391, EffectSize(g)+95%CI: 0.03(−0.74,0.81)  Combined (quadratic 4): 95%CI (kg):119.89–195.72, r= 0.997, SEE (kg): 1.62, p= 0.077, EffectSize(g)+95%CI: −0.04(−0.82,0.74)  Back squat (quadratic 7): 95%CI (kg): 119.15–193.40, r= 0.979, SEE (kg): 4.06, p= 0.502, EffectSize(g)+95%CI: 0.04(−0.74,0.81)  back squat (quadratic 4): 95%CI (kg):117.60–198.75, r= 0.996, SEE (kg): 1.82, p= 0.071, EffectSize(g)+95%CI: −0.06(−0.83,0.72)  combined (linear 7): 95%CI (kg):112.98–181.28, r= 0.990, SEE (kg): 2.82, p= <0.001, EffectSize(g)+95%CI: 0.52(−0.27,1.31)  combined (linear 4): 95%CI (kg):114.53–184.0, r=1 0.994, SEE (kg): 2.19, p= <0.001, EffectSize(g)+95%CI: 0.40(−0.38,1.19)  back squat (linear 7):95%CI (kg): 117.94–188.78, r= 0.988, SEE (kg): 3.11, p= 0.001, EffectSize(g)+95%CI: 0.19(−0.59,0.97)  back squat (linear 4): 95%CI (kg): 118.20–191.05, r= 0.987, SEE (kg): 3.26, p= 0.015, EffectSize(g)+95%CI: 0.12(−0.66,0.90) | The results of this study provide practitioners with confidence that a quadratic model that uses mean velocity of 80% 1RM and utilizes both ballistic and non-ballistic exercises is an effective method for estimating an individual’s 1RM in the free-weight back squat, ensuring load manipulation and fatigue management can be achieved on a sessional basis. It would also be feasible for a coach to employ this method at the beginning of a training session, estimate an athlete’s daily 1RM, adjust relevant working loads, and ensure parity between the loads prescribed and the intended training stimulus on that day. This would also allow coaches to utilize the integration of technology at the start of a training session, freeing up their time and attention for coaching for the remainder. |
| Pérez-Castilla et al. (2020) | This study examined the differences in the bench press one-repetition maximum obtained by three different methods (direct method, lifts-to-failure method, and two-point method). Twenty young men (age=22.5 +- 3.7years [range: 19–33years]) were tested in four different sessions. A single grip width (close, medium, wide, or self-selected) was randomly used on each session. Each session consisted of an incremental loading test until reaching the one-repetition maximum, followed by a single set of lifts-to failure against the 75% one-repetition maximum load. The last load lifted during the incremental loading test was considered the actual one-repetition maximum (direct method). The one-repetition maximum was also predicted using the Mayhew’s equation (lifts-to-failure method) and the individual load–velocity relationship modelled from two data points (two-point method). The actual one-repetition maximum was underestimated by the lifts-to-failure method (range: 1–2kg) and overestimated by the two-point method (range:–3 to–1kg), being these differences accentuated using closer grip widths. There were 48-72h rest between every session. | Bench press | Absolute differences between the actual and predicted 1RM during the bench press in a Smith machine:  Close grip width:  Difference with respect to the actual 1RM (kg) vs. Lifts-to-failure: r=0.96, SEE=4kg  Difference with respect to the actual 1RM (kg) vs. Two-point: r=0.98, SEE=3kg  Medium grip width:  Difference with respect to the actual 1RM (kg) vs. Lifts-to-failure: r=0.97, SEE=3kg  Difference with respect to the actual 1RM (kg) vs. Two-point: r=0.97, SEE=3kg  Wide grip width:  Difference with respect to the actual 1RM (kg) vs. Lifts-to-failure: r=0.95, SEE=4kg  Difference with respect to the actual 1RM (kg) vs. Two-point: r=0.98, SEE=3kg  Self-selected grip width:  Difference with respect to the actual 1RM (kg) vs. Lifts-to-failure: r=0.96, SEE=4kg  Difference with respect to the actual 1RM (kg) vs. Two-point: r=0.96, SEE=4kg  The strength of the relationship:  Close grip width:  Differences (Actual 1RM—lift-to-failure 1RM) (kg) vs. Mean actual and lifts-to-failure 1RM (kg): r^2^=0.00  Medium grip width:  Differences (Actual 1RM—lift-to-failure 1RM) (kg) vs. Mean actual and lifts-to-failure 1RM (kg): r^2^=0.12  Wide grip width:  Differences (Actual 1RM—lift-to-failure 1RM) (kg) vs. Mean actual and lifts-to-failure 1RM (kg): r^2^=0.00  Self-selected grip width:  Differences (Actual 1RM—lift-to-failure 1RM) (kg) vs. Mean actual and lifts-to-failure 1RM (kg): r^2^=0.00  Close grip width:  Differences (Actual 1RM—lift-to-failure 1RM) (kg) vs. Mean actual and Two-point 1RM (kg): r^2^=0.04  Medium grip width:  Differences (Actual 1RM—lift-to-failure 1RM) (kg) vs. Mean actual and Two-point 1RM (kg): r^2^=0.03  Wide grip width:  Differences (Actual 1RM—lift-to-failure 1RM) (kg) vs. Mean actual and Two-point 1RM (kg): r^2^=0.02  Self-selected grip width:  Differences (Actual 1RM—lift-to-failure 1RM) (kg) vs. Mean actual and Two-point 1RM (kg): r^2^=0.00  Two-point vs. direct: p=0.001, CI=-3 to -1 kg, ES=0.10-0.22  Two-point vs. lifts-to failure: p=0.001, CI=-6 to -1, ES=0.15-0.40  Medium vs close grip width: p=0.025, CI=-6 to -0kg, ES=-0.19-0.29  Medium vs wide grip width: p=0.036, CI=0-7kg, ES=0.18-0.33  Medium vs. self-selected grip width: p=1.000, CI=-2 to 5kg, ES=0.08-0.13 | The Mayhew’s lifts-to-failure equation and the two-point method reported an acceptable and comparable level of accuracy for predicting the 1RM during the Smith machine bench press exercise performed using different grip widths. It seems that the accuracy of both 1RM prediction methods could be slightly affected by the grip width (higher differences with respect to the actual 1RM using closer grip widths). |

**S17: Quality assessment part I**

| **Author** | **I1** | **I2** | **I3** | **I4** | **I5** | $\boldsymbol{\Sigma}$ |
| --- | --- | --- | --- | --- | --- | --- |
| Abbott et al. (2020) | 2 | 2 | 2 | 2 | 3 | 11 |
| Arede et al. (2019) | 1 | 3 | 2 | 2 | 2 | 10 |
| Askow et al. (2018) | 1 | 2 | 3 | 2 | 3 | 11 |
| Balsalobre-Fernández et al. (2016) | 1 | 2 | 3 | 2 | 3 | 11 |
| Bampouras et al. (2013) | 2 | 2 | 3 | 2 | 3 | 12 |
| Beckham et al. (2019) | 2 | 3 | 3 | 2 | 3 | 13 |
| Behrmann et al. (2025) | 3 | 3 | 3 | 2 | 3 | 14 |
| Boehringer & Whyte (2019) | 2 | 3 | 3 | 2 | 3 | 13 |
| Caruso et al. (2012) | 2 | 3 | 2 | 1 | 2 | 10 |
| Chavda et al. (2024) | 2 | 3 | 3 | 2 | 3 | 13 |
| Chéry & Ruf (2019) | 1 | 3 | 3 | 2 | 3 | 12 |
| Courel-Ibanez et al. (2019) | 2 | 3 | 3 | 2 | 3 | 13 |
| Elsworthy et al. (2020) | 2 | 2 | 3 | 2 | 3 | 12 |
| Ferro et al. (2019) | 1 | 2 | 2 | 3 | 3 | 11 |
| Feuerbacher et al. (2023) | 2 | 3 | 3 | 3 | 3 | 14 |
| Fritschi et al. (2021) | 1 | 3 | 2 | 2 | 2 | 10 |
| Garcia Ramos et al. (2016) | 2 | 3 | 2 | 1 | 3 | 11 |
| Garnacho-Castaño et al. (2015) | 3 | 3 | 3 | 2 | 3 | 14 |
| García-Pinillos et al. (2019) | 2 | 2 | 2 | 2 | 3 | 11 |
| Gant et al. (2023) | 2 | 2 | 2 | 2 | 3 | 11 |
| Gheller et al. (2023) | 2 | 3 | 3 | 3 | 3 | 14 |
| Goldsmith et al. (2018) | 2 | 2 | 2 | 2 | 3 | 11 |
| González-Galán et al. (2024) | 3 | 3 | 3 | 2 | 3 | 14 |
| Grgic et al. (2020) | 2 | 3 | 3 | 2 | 3 | 13 |
| Held et al. (2021) | 2 | 3 | 3 | 2 | 3 | 13 |
| Illera-Domínguez et al. (2022) | 2 | 2 | 2 | 2 | 2 | 10 |
| Janicijevic et al. (2021) | 2 | 3 | 3 | 2 | 2 | 12 |
| Jovanovic & Jukic (2020) | 1 | 3 | 3 | 2 | 3 | 12 |
| Jukic et al. (2023) | 3 | 3 | 3 | 2 | 3 | 14 |
| Justo-Álvarez et al. (2025) | 2 | 3 | 1 | 3 | 3 | 12 |
| Lake et al. (2018) | 2 | 2 | 3 | 2 | 3 | 12 |
| Lake et al. (2019) | 1 | 2 | 3 | 2 | 2 | 10 |
| Lawson et al. (2024) | 2 | 3 | 3 | 3 | 3 | 14 |
| Lopez-Torres et al. (2022) | 2 | 3 | 3 | 2 | 3 | 13 |
| Lorenzetti et al. (2017) | 1 | 2 | 2 | 2 | 3 | 10 |
| Maroto-Izquierdo et al. (2023) | 2 | 3 | 3 | 2 | 2 | 12 |
| Martin-San Augustin et al. (2020) | 2 | 3 | 1 | 3 | 3 | 12 |
| Martinez-Cava et al. (2020) | 2 | 3 | 2 | 2 | 3 | 12 |
| Mc Grath et al. (2018) | 1 | 3 | 2 | 2 | 3 | 11 |
| Miller et al. (2018) ( | 3 | 3 | 2 | 1 | 3 | 12 |
| Moreno-Villanueva et al. (2022) | 1 | 3 | 3 | 2 | 3 | 12 |
| Muyor et al. (2017) | 2 | 3 | 3 | 2 | 3 | 13 |
| Orange et al. (2020) | 2 | 3 | 3 | 2 | 3 | 13 |
| Olaya-Cuartero et al. (2022) | 2 | 2 | 3 | 3 | 2 | 12 |
| Oleksy et al. (2023) | 2 | 3 | 2 | 2 | 3 | 12 |
| Perez-Castilla et al. (2018) | 2 | 2 | 3 | 2 | 3 | 12 |
| Pérez-Castilla et al. (2019) | 1 | 3 | 3 | 2 | 2 | 11 |
| Perez-Castillia et al. (2021) | 1 | 3 | 3 | 2 | 3 | 12 |
| Perez-Castillia et al. (2021) | 2 | 3 | 3 | 2 | 3 | 13 |
| Pelka et al. (2023) | 2 | 3 | 2 | 2 | 3 | 12 |
| Pinzone et al. (2024) | 2 | 3 | 3 | 3 | 3 | 14 |
| Pueo et al. (2021) | 2 | 3 | 3 | 2 | 3 | 13 |
| Qu et al. (2024) | 1 | 3 | 3 | 2 | 3 | 12 |
| Ruiz-Alias et al. (2024) | 1 | 3 | 3 | 2 | 3 | 12 |
| Sato et al. (2009) | 1 | 2 | 2 | 2 | 2 | 9 |
| Sousa et al. (2024) | 2 | 3 | 3 | 2 | 2 | 12 |
| Stock et al. (2011) | 2 | 3 | 3 | 1 | 3 | 12 |
| Suchomel et al. (2023) | 2 | 2 | 2 | 2 | 3 | 11 |
| Van den Tillaar & Ball (2019) | 2 | 3 | 3 | 2 | 3 | 13 |
| Van den Tillaar et al. (2024) | 2 | 3 | 3 | 3 | 3 | 14 |
| Wagner et al. (2025) | 3 | 3 | 3 | 2 | 3 | 14 |
| Warneke et al. (2024) | 2 | 2 | 3 | 2 | 3 | 12 |
| Zhang et al. (2024) | 1 | 3 | 2 | 2 | 2 | 10 |

**S18: Quality assessment part II**

| **Author** | **I1** | **I2** | **I3** | **I4** | **I5** | **Σ** |
| --- | --- | --- | --- | --- | --- | --- |
| Balsalobre-Fernández et al. (2019) | 2 | 2 | 2 | 2 | 2 | 10 |
| Balsalobre-Fernández & Kipp (2021) | 3 | 2 | 2 | 2 | 3 | 12 |
| Banyard et al. (2017) | 2 | 3 | 3 | 3 | 3 | 14 |
| Bazuelo-Ruiz et al. (2015) | 3 | 3 | 2 | 3 | 2 | 13 |
| Benavides-Ubric et al. (2020) | 3 | 3 | 3 | 3 | 3 | 15 |
| Berton et al. (2021) | 2 | 3 | 3 | 3 | 2 | 13 |
| Bosquet et al. (2010) | 2 | 2 | 2 | 3 | 2 | 11 |
| Caven et al. (2020) | 2 | 3 | 2 | 2 | 3 | 12 |
| Cetin et al. (2022) | 2 | 3 | 3 | 2 | 3 | 13 |
| da Silva et al. (2023) | 1 | 3 | 3 | 3 | 2 | 12 |
| dos Santos et al. (2023) | 2 | 3 | 2 | 3 | 3 | 13 |
| Fitas et al. (2024) | 2 | 2 | 2 | 3 | 2 | 11 |
| Fernandes et al. (2021) | 2 | 3 | 2 | 2 | 2 | 11 |
| Gantois et al. (2022) | 2 | 2 | 2 | 2 | 2 | 10 |
| García-Ramos et al. (2019) | 2 | 2 | 3 | 3 | 2 | 12 |
| García-Ramos et al. (2018) | 3 | 3 | 3 | 3 | 3 | 15 |
| Hughes et al. (2019) | 2 | 3 | 3 | 3 | 3 | 14 |
| Jukic et al. (2022) | 2 | 3 | 2 | 3 | 2 | 12 |
| Janicijevic et al. (2021) | 3 | 3 | 2 | 3 | 2 | 13 |
| Janicijevic et al. (2024) | 2 | 3 | 2 | 2 | 3 | 12 |
| Jiménez-Alonso et al. (2022) | 2 | 3 | 2 | 3 | 2 | 12 |
| Kilgallon et al. (2022) | 2 | 3 | 2 | 3 | 3 | 13 |
| Larsen et al. (2023) | 2 | 2 | 3 | 3 | 2 | 12 |
| Lake et al. (2017) | 1 | 2 | 2 | 3 | 2 | 10 |
| Macarilla et al. (2022) | 2 | 2 | 1 | 3 | 2 | 10 |
| Oberhofer et al. (2021) | 3 | 3 | 2 | 2 | 3 | 13 |
| Pérez-Castilla et al. (2019) | 1 | 2 | 2 | 3 | 3 | 11 |
| Pérez-Castilla et al. (2020) | 2 | 3 | 2 | 3 | 3 | 13 |
| Pérez-Castilla et al. (2021) - Study A | 2 | 3 | 3 | 3 | 3 | 14 |
| Pérez-Castilla et al. (2021) – Study B | 2 | 3 | 1 | 3 | 3 | 12 |
| Pestaña-Melero et al. (2018) | 3 | 3 | 2 | 2 | 2 | 12 |
| Ruf et al. (2018) | 1 | 3 | 3 | 3 | 3 | 13 |
| Sayers et al. (2018) | 1 | 3 | 2 | 3 | 3 | 12 |
| Sandau et al. (2021) | 1 | 2 | 2 | 3 | 2 | 10 |
| Sigvaldsen et al. (2023) | 2 | 3 | 3 | 3 | 3 | 14 |
| Simonsen et al. (2025) | 2 | 3 | 3 | 3 | 2 | 13 |
| Soriano et al. (2023) | 2 | 3 | 3 | 3 | 2 | 13 |
| Thompson et al. (2021) | 1 | 3 | 1 | 3 | 2 | 10 |

**S19: Results part I**

| **Device agreement** | **Moderator** | **Effekt Size (95% CI)** | **Test of Moderators (p-value)** | **Residual Heterogeneity (p-value)** | **Beta-value (p-value)** |
| --- | --- | --- | --- | --- | --- |
| **Device agreement ICC** | (k=439) | ICC=0.92 (0.83 – 0.97) |  | Q(438) = 1317.5317 (p<0.0001) |  |
|  | Exercise category (k=439) |  | QM(3) = 12.9107 (p=0.0048) | Q(435) = 1313.4649 (p<0.0001) | Hip thrust: -0.6091 (p=0.6598)  Squat: 0.3693 (p=0.0005)  Push up: 1.3774 (p=0.4858) |
|  | Variable (k=439) |  | QM(2) = 0.9243 (p=0.6299) | Q(436) = 1301.4248 (p<0.0001) | MV: -0.1590 (p=0.4028)  PV: -0.0732 (p=0.6826) |
|  | Intensity (k=439) |  | QM(2) = 10.2854 (p=0.0058) | Q(436) = 1280.4945 (p<0.0001) | Moderate: 0.4166 (p=0.0032)  High: 0.4345 (p=0.0061) |
|  | Sensor type (k=439) |  | QM(17) = 49.1230 (p<0.0001) | Q(421) = 584.3495 (p<0.0001) | Chronojump: 5.0217 (p=0.00839  EnodePro: 3.3031 (p=0.0683)  ET-Enc-02: 3.3554 (p=0.0836)  Gyko: 0.4331 (p=0.8573)  GymAware: 3.3835 (p=0.0637)  HUMAC: 0.09754 (p=0.6388)  MuscleLab: 3.4435 (p=0.0585)  PowerLift: 3.1576 (p=0.1159)  PUSH: 2.6658 (p=0.1441)  RepOne: 5.0322 (p=0.0334)  Speed4Lifts: 2.6271 (p=0.1493)  T-force: 4.4155 (p=0.0678)  Velowin: 5.2009 (p=0.0063)  Vitruve1: 3.2069 (p=0.1770)  Vitruve2: 3.4404 (p=0.1475) |
| **Device agreement Lin** | (k=64) | CCC=0.90 (0.52-0.98) |  | Q(63) = 1083.5052 (p<0.0001) |  |
|  | Exercise category (k=64) |  | QM(1) = 21.5331 (p<0.0001) | Q(62) = 1046.4502 (p<0.0001) | Squat: -0.2140 (p<0.0001) |
|  | Variable (k=64) |  | QM(2) = 42.3680 (p<0.0001) | Q(61) = 899.9288 (p<0.0001) | MV: -0.2412 (p=0.0728)  PV: -0.2858 (p<0.0001) |
|  | Intensity (k=64) |  | QM(2) = 1.4669 (p=0.4803) | Q(61) = 911.7790 (p<0.0001) | Moderate: 0.0294 (p=0.8456)  High: 0.1437 (p=0.2418) |
|  | Sensor type (k=64) |  | QM(3) = 17.9214 (p=0.0005) | Q(60) = 705.1011 (p<0.0001) | IMS: 1.5817 (p<0.0001)  Vitruve1: 0.8621 (p=0.0274)  Vitruve2: 0.9231 (p=0.0182) |
| **Device agreement Pearson** | (k=227) | r=0.91 (0.84-0.95) |  | Q(226) = 1550.4836 (p<0.0001) |  |
|  | Exercise category (k=227) |  | QM(7) = 56.2392 (p<0.0001) | Q(219) = 1330.4131 (p<0.0001) | Deadlift: -0.3033 (p=0.4819)  High-pull: 0.5915 (p=0.3972)  Hip extension: -0.5471 (p=0.2360)  Hip thrust: -0.6917 (p=0.0457)  Pushup: -0.0112 (p=0.9627)  Squat: -0.3437 (p<0.0001)  Unilateral straight knee bridge: -0.5078 (p=0.2714) |
|  | Variable (k=227) |  | QM(3) = 64.5709 (p<0.0001) | Q(223) = 1334.0368 (p<0.0001) | MPV: -0.8597 (p=0.1587)  MV: -0.9651 (p=0.1101)  PV: -0.6423 (p=0.2879) |
|  | Intensity (k=222) |  | QM(2) = 13.9214 (p=0.0009) | Q(219) = 1488.9526 (p<0.0001) | Moderate: 0.1651 (p=0.0002)  High: 0.1127 (p=0.0469) |
|  | Sensor type (k=227) |  | QM(11) = 27.3609 (p=0.0041) | Q(215) = 776.4803 (p<0.0001) | IMS: -0.3073 (p=0.6487)  EnodePro: -0.6035 (p=0.2759)  Gyko: -1.4310 (p=0.0366)  iLoad: -0.7256 (p=0.1858)  Jueying: 0.5175 (p=4465)  Pasco: 0.3034 (p=0.6567)  PUSH: -0.9592 (p=0.0763)  RepOne: 0.2043 (p=0.7606)  Vitruve: -0.1494 (p=0.8015)  WIMU: -1.0352 (p=0.1229) |
| **VALIDITY** |  | **Effekt Size (95% CI)** | **Test of Moderators (p-value)** | **Residual Heterogeneity (p-value)** | **Beta-value (p-value)** |
| **Device validity ICC** | (k=28) | ICC=0.91 (0.87 – 0.94) |  | Q(27) = 15.3945 (p=0.9635) |  |
|  | Exercise category (k=28) |  | QM(1) = 0.1661 (p=0.6836) | Q(26) = 15.2763 (p<0.0001) | Squat: -0.1708 (p=0.6836) |
|  | Variable (k=26) |  | QM(1) = 1.2399 (p=0.2655) | Q(26) = 14.1546 (p=0.9709) | PV: 0.5884 (p=0.2655) |
|  | Intensity* |  | - | - | - |
|  | Sensor type (k=28) |  | QM(4) = 2.9026 (p=0.5743) | Q(23) = 11.1483 (p=0.9816) | GymAware: 1.6281 (p=0.1532)  OBS: 0.7239 (p=0.5154)  PUSH: 0.0756 (p=0.9308)  Tendo: 0.4129 (p=0.6798) |
|  | Goldstandard (k=28) |  | QM(3) = 5.4282 (p=0.1430) | Q(24) = 9.9663 (p=0.9947) | Optotrak: -0.9487 (p=0.2961)  Qualysis: 0.5534 (p=0.6022)  Vicon: -1.0408 (p=0.1860) |
| **Device validity Pearson** | (k=55) | r=0.95 (0.82 – 0.98) |  | Q(54) = 689.2566 (p<0.0001) |  |
|  | Exercise category (k=55) |  | QM(3) = 4.6849 (p=0.1964) | Q(51) = 469.3721 (p<0.0001) | Bench press: -1.4438 (p=0.0347)  Jump: -1.4644 (p=0.1023)  Squat: -1.4248 (p=0.0336) |
|  | Variable (k=55) |  | QM(2) = 20.5089  (p=<0.0001) | Q(52) = 612.4912 (p<0.0001) | PV: 0.1836 (p=0.5380)  MV: -0.3790 (p=0.2010) |
|  | Intensity (k=50) |  | QM(2) = 4.2270 (p=0.1208) | Q(47) = 648.5209  (p<0.0001) | Moderate: 0.4305 (p=0.1556)  High: 0.7102 (p=0.0409) |
|  | Sensor type (k=55) |  | QM(10) = 163.8618 (p<0.0001) | Q(44) = 354.6417  (p<0.0001) | EnodePro: 0.3446 (p=0.7076)  Ergonauta: -0.0471 (p=0.9684)  Flex: -0.3160 (p=0.7386)  GymAware: 0.0173 (p=0.9884)  Myotest: -0.8543 (p=0.4783)  PUSH: -0.0767 (p=0.9342)  Quantum: 2.9871 (p=0.0016)  T-force: -0.0653 (p=0.9562)  Tendo: -0.0262 (p=0.9824) |
|  | Goldstandard (k=55) |  | QM(3) = 5.6511  (p=0.1299) | Q(51) = 548.2449 (p<0.0001) | Kistler FP: -0.6869 (p=0.3877)  OptiTrack: 0.5996 (p=0.4540)  Vicon: -0.9106 (p=0.0979) |

| **RELIABILITY** | **Moderator** | **Effekt Size fisher´s z (95% CI)** | **Test of Moderators (p-value)** | **Residual Heterogeneity (p-value)** | **Beta-value (p-value)** |
| --- | --- | --- | --- | --- | --- |
| **Interday reliability ICC** | (k=608) | ICC=0.90 (0.85 – 0.93) |  | Q(607) = 950.1343 (p<0.0001) |  |
|  | Exercise category (k=608) |  | QM(6) = 7.7425 (p=0.2576) | Q(601) = 868.8877 (p<0.0001) | Bench press: -0.2268 (p=0.8616)  Deadlift: -0.6610 (p=0.6212)  Hip thrust: -1.4236 (p=0.3010)  Jump: -0.3456 (p=0.8181)  LPT: -0.6813 (p=0.7041)  Squat: -0.2776 (p=0.8308) |
|  | Variable (k=600) |  | QM(26) = 33.6457 (p=0.1442) | Q(573) = 871.5969 (p<0.0001) | MPV: -0.2841 (p=0.8646)  MV: -0.3181 (p=0.8469)  PV: -0.1733 (p=0.9162) |
|  | Intensity (k=591) |  | QM(2) = 1.5759 (p=0.4548) | Q(588) = 762.5801 (p<0.0001) | Moderate: -0.1455 (p=0.2094)  High: -0.0732 (p=0.5697) |
|  | Sensor type (k=608) |  | QM(20) = 47.2795 (p=0.0005) | Q(587) = 602.7533 (p=0.3173) | ADR: 4.1144 (p=0.3338)  Bar Sensei: -1.8709 (p=0.2838)  FP BioWare: 1.3122 (p=0.4396)  Chronjump: 1.4391 (p=0.2749)  EnodePro: 0.8369 (p=0.5093)  GymAware: 0.9677 (p=0.4368)  HUMAC: 0.8608 (p=0.5600)  Jueying: 0.8253 (p=0.6311)  Myotest: 0.5235 (p=0.7372)  Powerlift: 0.4022 (p=0.7832)  PowerTool: -0.0426 (p=0.7832)  PUSH: 0.0041 (p=0.9974)  RepOne: 0.7589 (p=0.5466)  SmartCoach: 0.2970 (p=0.8617)  Speed4Lifts: 0.5538 (p=0.6797)  T-force: 1.6202 (p=0.1941)  Tendo: 0.9531 (p=0.4455)  Velowin: 1.3435 (p=0.3060)  Vicon: 1.4095 (p=0.2930)  WIMU: 1.6258 (p=0.2372) |
| **Interday reliability LIN** | (k=6) | CCC=0.99 (0.99-0.99) |  | Q(5) = 30.6065 (p<0.0001) |  |
|  | Exercise category* |  | - | - | - |
|  | Variable (k=6) |  | QM(1) = 14.2139 (p=0.0002) | Q(4) = 16.3926 (p=0.0025) | PV: -0.4443 (p=0.0002) |
|  | Intensity* |  | - | - | - |
|  | Sensor type (k=6) |  | QM(2) = 13.8386 (p=0.0010) | Q(3) = 16.7679 (p=0.0008) | GymAware: -0.1061 (p=0.4624)  PUSH: -0.5089 (p=0.0004) |
| **Interday reliability Pearson** | (k=44) | r=0.91 (0.38 – 0.99) |  | Q(43) = 232.0732 (p<0.0001) |  |
|  | Exercise category (k=44) |  | QM(3) = 45.2707 (p<0.0001) | Q(40) = 75.1626 (p=0.0006) | Hip thrust: -2.0193 (p<0.0001)  LPT: -1.5798 (p<0.0001)  Squat: -1.5949 (p<0.0001) |
|  | Variable (k=44) |  | QM(1) = 3.1099 (p=0.0778) | Q(42) = 231.2427 (p<0.0001) | PV: 0.1329 (p=0.0778) |
|  | Intensity* |  | - | - | - |
|  | Sensor type (k=44) |  | QM(3) = 41.2350 (p<0.0001) | Q(40) = 77.9662 (p=0.0003) | EnodePro: -1.7722(p<0.0001)  GymAware: -1.5798 (p<0.0001)  Speed4Lifts: -1.8420 (p<0.0001) |
| **Intraday reliability ICC** | (k=228) | ICC=0.91 (0.87 – 0.95) |  | Q(227) = 268.2198 (p=0.0315) |  |
|  | Exercise category (k=228) |  | QM(6) = 12.1060 (p=0.0596) | Q(221) = 227.4519 (p=0.3686) | Bench press: 1.0677 (p=0.2685)  Bench pull: 0.0658 (p=0.9481)  Hip thrust: 1.0399 (p=0.3275)  Jump: 1.2855 (p=0.2926)  LPT: 0.3082 (p=0.8101)  Squat: 1.2653 (p=0.1958) |
|  | Variable (k=228) |  | QM(24) = 20.2217 (p=0.6841) | Q(203) = 229.4752 (p=0.0979) | MV: 0.7018 (p=0.6023)  MPV: 2.9736 (p=0.1081)  PV: 0.8284 (p=0.5404) |
|  | Intensity (k=180) |  | QM(2) = 2.4379 (p=0.2955) | Q(177) = 216.1111 (p=0.0240) | Moderate: 0.2316 (p=0.1633)  High: 0.3076 (p=0.1856) |
|  | Sensor type (k=228) |  | QM(14) = 35.8681 (p=0.0011) | Q(213) = 161.8462 (p=0.9963) | Beast: -2.1296 (p=0.0038)  Chronojump: 0.1452 (p=0.7624)  EnodePro: -0.6280 (p=0.1463)  Ergonauta: 1.9759 (p=0.1656)  GymAware: -0.8839 (p=0.3808)  HUMAC: 0.4249 (p=0.6752)  PowerLift: -0.0849 (p=0.8954)  PUSH: -0.9205 (p=0.0804)  Speed4lifts: 0.0801 (p=0.8354)  T-force: 0.2816 (p=0.4621)  Tendo: -0.9087 (p=0.3662)  Velowin: -0.4868 (p=0.4530)  Vicon: -0.1669 (p=0.7114) |
| **Intraday reliability Pearson** | (k=4) | r=0.89 (0.83 – 0.93) |  | Q(3) = 2.8576 (p=0.4141) |  |
|  | Exercise category (k=4) |  | QM(1) = 0.0747 (p=0.7846) | Q(2) = 2.7828 (p=0.2487) | Squat: 0.0683 (p=0.7846) |
|  | Variable* |  | - | - | - |
|  | Intensity* |  | - | - | - |
|  | Sensor type (k=4) |  | QM(1) = 2.4794 (p=0.1153) | Q(2) = 0.3781 (p=0.8277) | Speed4Lifts: 0.3937 (p=0.1153) |

OBS – Open barbell System, *analysis has less than two unique values

**S20: Results part II**

| **Interday Reliability ICC** | (k=124) | ICC=0.90 (0.83-0.94) |  | Q(123) = 7136.7079 (p<0.0001) |  |
| --- | --- | --- | --- | --- | --- |
|  | Exercise category (k=124) |  | QM(4) = 312.9288 (p<0.0001) | Q(119) = 5912.4016 (p<0.0001) | Deadlift: 0.2841 (p=0.0786) Power Clean: -0.9417 (0.4162)  Bench pull: 0.3410 (p=0.7677)  Squat: -1.2756 (p<0.001) |
|  | Prediction Method (k=124) |  | QM(1) = 0.1015 (p=0.7501) | Q(122) = 6764.6418 (p<0.0001) | Polynomial reg.: -0.0088 (p=0.7501) |
|  | Variable (k=124) |  | QM(3) = 4.5848 (p=0.2048) | Q(120) = 6472.9515 (p<0.0001) | MPV: -0.2566 (p=0.7409)  MV: 0.1955 (p=0.8004)  PV: -0.4445 (p=0.5668) |
| **Validity ICC** | (k=9) | ICC=0.91 (0.72-0.98) |  | Q(8) = 914.1355 (p<0.0001) |  |
|  | Exercise category (k=9) |  | QM(2) = 19.6636 (p<0.0001) | Q(6) = 182.9708 (p<0.0001) | Deadlift: 1.7708 (p=0.0013)  Squat: -0.5701 (p=0.3015) |
|  | Prediction Method* |  | - | - | - |
|  | Variable (k=9) |  | QM(1) = 3.9884 (p=0.0458) | Q(7) = 873.0812 (p<0.0001) | PV: 0.2353 (p=0.0458) |
| **Validity Pearson** | (k=188) | r=0.96 (0.94-0.97) |  | Q(187) = 1472.4957 (p<0.0001) |  |
|  | Exercise category (k=188) |  | QM(10) = 83.4746 (p<0.0001) | Q(177) = 982.4312 (p<0.0001) | Squat: -0.9806 (p<0.0001)  Bench press: 0.0346 (p=0.6850)  Deadlift: -0.9393 (p<0.0001)  Horizontal pull: -0.3028 (p=0.0075)  Knee extension: -0.3399 (p=0.5891)  Lat pull: 0.5091 (p=0.0025)  Overhead press: 0.1972 (p=0.5120)  Bench pull: -0.2927 (p=0.2683)  Cable row: 0.4718 (p=0.0051)  Snatch pull: 0.5509 (p=0.4648) |
|  | Prediction Method (k=188) |  | QM(6) = 46.2857 (p<0.0001) | Q(181) = 1333.5553 (p<0.0001) | Lin. F-V reg.: -0.3565 (p=0.5161)  Lin. L-V reg.: -0.0918 (p=0.2591)  Mulitlin. reg.: 0.0078 (p=0.9338)  Polynomial reg.: -0.3941 (p<0.0001)  Quadratic: 0.1348 (p=0.5475)  Regularized reg.: 0.0078 (p=0.9338) |
|  | Variable (k=188) |  | QM(7) = 6.4760 (p=0.4854) | Q(180) = 1035.1192 (p<0.0001) | MCV: 0.3735 (p=0.3453)  MPV: 0.1102 (p=0.7295)  MV: 0.0690 (p=0.8320)  PV: -0.0212 (p=0.9489) |

*analysis has less than two unique values

**S21: Prisma Checklist**

| **Section and Topic** | **Item #** | **Checklist item** | **Location where item is reported** |
| --- | --- | --- | --- |
| **TITLE** | | |  |
| Title | 1 | Identify the report as a systematic review | Title page |
| **ABSTRACT** | | |  |
| Abstract | 2 | See the PRISMA 2020 for Abstracts checklist. | Abstract |
| **INTRODUCTION** | | |  |
| Rationale | 3 | Describe the rationale for the review in the context of existing knowledge | Introduction |
| Objectives | 4 | Provide an explicit statement of the objective(s) or question(s) the review addresses | Methods 2.1 Literature search; 2.2 Quality Assessment |
| **METHODS** | | |  |
| Eligibility criteria | 5 | Specify the inclusion and exclusion criteria for the review and how studies were grouped for the syntheses. | Methods 2.1 Literature search |
| Information sources | 6 | Specify all databases, registers, websites, organisations, reference lists and other sources searched or consulted to identify studies. Specify the date when each source was last searched or consulted. | Methods  2.1 Literature search |
| Search strategy | 7 | Present the full search strategies for all databases, registers and websites, including any filters and limits used. | Methods  2.1 Literature search, Supplementary (SX) |
| Selection process | 8 | Specify the methods used to decide whether a study met the inclusion criteria of the review, including how many reviewers screened each record and each report retrieved, whether they worked independently, and if applicable, details of automation tools used in the process. | Methods  2.1 Literature search |
| Data collection process | 9 | Specify the methods used to collect data from reports, including how many reviewers collected data from each report, whether they worked independently, any processes for obtaining or confirming data from study investigators, and if applicable, details of automation tools used in the process. | Methods  2.3 Data processing and Statistics |
| Data items | 10a | List and define all outcomes for which data were sought. Specify whether all results that were compatible with each outcome domain in each study were sought (e.g. for all measures, time points, analyses), and if not, the methods used to decide which results to collect. | Methods 2.2 Quality Assessment |
|  | 10b | List and define all other variables for which data were sought (e.g. participant and intervention characteristics, funding sources). Describe any assumptions made about any missing or unclear information. | Methods 2.2 Quality Assessment, Methods 2.3 Data processing and Statistics |
| Study risk of bias assessment | 11 | Specify the methods used to assess risk of bias in the included studies, including details of the tool(s) used, how many reviewers assessed each study and whether they worked independently, and if applicable, details of automation tools used in the process. | Methods 2.2 Quality Assessment |
| Effect measures | 12 | Specify for each outcome the effect measure(s) (e.g. risk ratio, mean difference) used in the synthesis or presentation of results. | Methods 2.3 Data processing and Statistics |
| Synthesis methods | 13a | Describe the processes used to decide which studies were eligible for each synthesis (e.g. tabulating the study intervention characteristics and comparing against the planned groups for each synthesis (item #5)). | Methods 2.3 Data processing and Statistics; Supplementary |
|  | 13b | Describe any methods required to prepare the data for presentation or synthesis, such as handling of missing summary statistics, or data conversions. | Methods 2.3 Data processing and Statistics |
|  | 13c | Describe any methods used to tabulate or visually display results of individual studies and syntheses. | Methods 2.3 Data processing and Statistics |
|  | 13d | Describe any methods used to synthesize results and provide a rationale for the choice(s). If meta-analysis was performed, describe the model(s), method(s) to identify the presence and extent of statistical heterogeneity, and software package(s) used. | Methods 2.3 Data processing and Statistics |
|  | 13e | Describe any methods used to explore possible causes of heterogeneity among study results (e.g. subgroup analysis, meta-regression). | Methods 2.3 Data processing and Statistics |
|  | 13f | Describe any sensitivity analyses conducted to assess robustness of the synthesized results. | Methods 2.3 Data processing and Statistics |
| Reporting bias assessment | 14 | Describe any methods used to assess risk of bias due to missing results in a synthesis (arising from reporting biases). | Methods 2.3 Data processing and Statistics |
| Certainty assessment | 15 | Describe any methods used to assess certainty (or confidence) in the body of evidence for an outcome. | Methods 2.2 Quality Assessment |
| **RESULTS** | | |  |
| Study selection | 16a | Describe the results of the search and selection process, from the number of records identified in the search to the number of studies included in the review, ideally using a flow diagram. | Results 3.1.2; Results 3.2.2; Fig. 1; Fig. 3 |
|  | 16b | Cite studies that might appear to meet the inclusion criteria, but which were excluded, and explain why they were excluded. | Figure 1 and Figure 3 |
| Study characteristics | 17 | Cite each included study and present its characteristics. | Results 3.1.2; Results 3.2.2; Supplementary Table S15 |
| Risk of bias in studies | 18 | Present assessments of risk of bias for each included study. | Results 3.1.3; Results 3.2.3; Supplementary Table S16 & S17 |
| Results of individual studies | 19 | For all outcomes, present, for each study: (a) summary statistics for each group (where appropriate) and (b) an effect estimate and its precision (e.g. confidence/credible interval), ideally using structured tables or plots. | Results 3.1.4.1; Results 3.1.5.1; Results 3.1.6.1; Results 3.2.4.1; Results 3.2.5.1; Supplementary Fig S2-S14 |
| Results of syntheses | 20a | For each synthesis, briefly summarise the characteristics and risk of bias among contributing studies. | Results 3.1.2; Results 3.1.3; Results 3.2.2; Results 3.2.3; Supplementary Table S15-S17 |
|  | 20b | Present results of all statistical syntheses conducted. If meta-analysis was done, present for each the summary estimate and its precision (e.g. confidence/credible interval) and measures of statistical heterogeneity. If comparing groups, describe the direction of the effect. | Results 3.1.4.1; 3.1.5.1; 3.1.6.1; 3.2.4.1; 3.2.5.1; Figure 2, 4, 5; Supplementary Fig. S2-S14 |
|  | 20c | Present results of all investigations of possible causes of heterogeneity among study results. | Results 3.1.4.2; 3.1.5.2; 3.1.6.2; 3.2.4.2; 3.2.5.2; Table 1 |
|  | 20d | Present results of all sensitivity analyses conducted to assess the robustness of the synthesized results. | Not applicable |
| Reporting biases | 21 | Present assessments of risk of bias due to missing results (arising from reporting biases) for each synthesis assessed. | Results 3.1.1; 3.2.1; Supplementary Fig. S2-S14 |
| Certainty of evidence | 22 | Present assessments of certainty (or confidence) in the body of evidence for each outcome assessed. | Not applicable |
| **DISCUSSION** | | |  |
| Discussion | 23a | Provide a general interpretation of the results in the context of other evidence. | Discussion 4.1; 4.2; 4.3.1; 4.3.2; 4.4.1; 4.4.2 |
|  | 23b | Discuss any limitations of the evidence included in the review. | Discussion 4.1; 4.2; 4.3.1; 4.3.2; 4.4.1; 4.4.2 |
|  | 23c | Discuss any limitations of the review processes used. | Limitations 5 |
|  | 23d | Discuss implications of the results for practice, policy, and future research. | Not applicable |
| **OTHER INFORMATION** | | |  |
| Registration and protocol | 24a | Provide registration information for the review, including register name and registration number, or state that the review was not registered. | Abstract; Methods 2 |
|  | 24b | Indicate where the review protocol can be accessed, or state that a protocol was not prepared. | Abstract; Methods 2 |
|  | 24c | Describe and explain any amendments to information provided at registration or in the protocol. | No amendments were made to the registered protocol |
| Support | 25 | Describe sources of financial or non-financial support for the review, and the role of the funders or sponsors in the review. | end |
| Competing interests | 26 | Declare any competing interests of review authors. | end |
| Availability of data, code and other materials | 27 | Report which of the following are publicly available and where they can be found: template data collection forms; data extracted from included studies; data used for all analyses; analytic code; any other materials used in the review. | Supplementary materials |

*From:* Page MJ, McKenzie JE, Bossuyt PM, Boutron I, Hoffmann TC, Mulrow CD, et al. The PRISMA 2020 statement: an updated guideline for reporting systematic reviews. BMJ 2021;372:n71. doi: 10.1136/bmj.n71. This work is licensed under CC BY 4.0. To view a copy of this license, visit <https://creativecommons.org/licenses/by/4.0/>
